# Maackiain Modulates miR-374a/GADD45A Axis to Inhibit Triple-Negative Breast Cancer Initiation and Progression

**DOI:** 10.3389/fphar.2022.806869

**Published:** 2022-03-04

**Authors:** Fu Peng, Li Wang, Liang Xiong, Hailin Tang, Junrong Du, Cheng Peng

**Affiliations:** ^1^ Key Laboratory of Drug-Targeting and Drug Delivery System of the Education Ministry and Sichuan Province, Sichuan Engineering Laboratory for Plant-Sourced Drug and Sichuan Research Center for Drug Precision Industrial Technology, West China School of Pharmacy, Sichuan University, Chengdu, China; ^2^ State Key Laboratory of Southwestern Chinese Medicine Resources, Chengdu University of Traditional Chinese Medicine, Chengdu, China; ^3^ Department of Breast Oncology, Sun Yat-sen University Cancer Center, Guangzhou, China

**Keywords:** triple negative breast cancer, maackiain, miR-374a, GADD45α, EMT—epithelial to mesenchymal transformation

## Abstract

Breast cancer ranks as the leading cause of death in lethal malignancies among women worldwide, with a sharp increase of incidence since 2008. Triple negative breast cancer (TNBC) gives rise to the largest proportion in breast cancer-related deaths because of its aggressive growth and rapid metastasis. Hence, searching for promising targets and innovative approaches is indispensable for the TNBC treatment. Maackiain (MA), a natural compound with multiple biological activities, could be isolated from different Chinese herbs, such as *Spatholobus suberectus* and *Sophora flavescens*. It was the first time to report the anti-cancer effect of MA in TNBC. MA could suppress TNBC cell proliferation, foci formation, migration, and invasion. MA also exerted a significant inhibitory effect on tumor growth of TNBC. Furthermore, MA could induce apoptosis with an increase of GADD45α and a decrease of miR-374a. In contrast, overexpressing miR-374a would result in at least partly affecting the proapoptotic effect of MA and suppressing GADD45α stimulated by MA. These results reveal the anti-TNBC effect of MA *in vitro* and *in vivo*, providing evidence for its potential as a drug candidate utilized in TNBC therapy.

## Introduction

In 2020, breast cancer still ranked as the leading cause of death in cancer-related female patients worldwide, with the highest incidence (47.8 per 100,000 population) and the second highest mortality (13.6 per 100,000 population) based on the data from the Global Cancer Observatory database (An et al., 2021). According to molecular profiling, breast cancer is a complicated disease including various molecular subtypes, namely, luminal A, luminal B, human epidermal growth factor receptor 2 (HER2)-enriched, and triple-negative (Sinha et al., 2013). Approximately 15% of diagnosed breast cancers are triple negative breast cancer (TNBC), exerting higher recurrence, more aggressive growth, and more rapid metastasis (Tang et al., 2019). TNBC patients do not respond to hormone therapy or HER2 targeted agents because TNBC is the absence of hormone-receptor and HER2. Thus, it is urgent to search for novel targets and approaches to rise to the TNBC clinical challenge.

**FIGURE 1:**
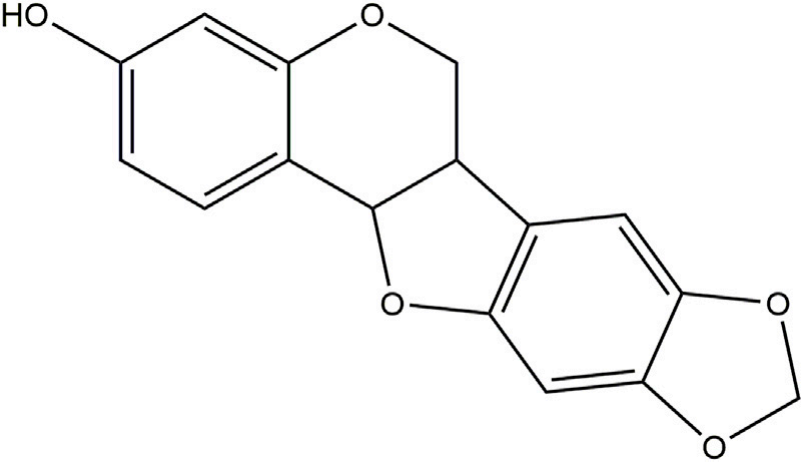
The chemical structure of MA constructed using ChemBioDraw.

MicroRNAs (miRNAs) are classified as a class of small non-coding RNA molecules of 20-22 nucleotides negatively regulating their targets through cleavage or translational repression (Mendell et al., 2012). MiRNAs modulate diverse cellular processes relying on the impressive regulatory function even in cancer. The first study exploring the role of miRNAs in breast cancer was reported in 2005. The findings indicated overexpressed miR-21 and miR-155 in breast cancer patients as oncogenic gene (Iorio et al., 2005). Since then, a substantial number of studies started to focus on this novel conserved non-coding RNA in breast cancer management. Recently, it was pointed out that miR-374a could induce tumorigenicity and progression of breast cancer through the deregulation of *β*-lactamases, which was detected as part of the mitochondrial ribosomal complex (Zhang et al., 2018). Additionally, in TNBC, miR-374a promoted cancer development by directly targeting arrestin *β* 1, a member of the arrestin/β-arrestin family (Son et al., 2019). These reports suggested miR-374a as an oncogene in breast cancer. Therefore, it is worthwhile to expand the underlying mechanism of miR-374a regulation and discover approaches to suppress miR-374a in TNBC. *Spatholobus suberectus* Dunn (*S. suberectus*), a traditional Chinese medicine (TCM), could promote blood circulation and remove blood stasis according to TCM theory, implying its function to treat blood-stasis related diseases. *S. suberectus* was commonly used to treat rheumatism, anemia, and menoxenia in clinic (Qin et al., 2019). *S. suberectus* has been shown to have diverse pharmacological properties including anti-inflammatory, anti-bacterial, antioxidant, and antidiabetic effects (Fu et al., 2017; Do et al., 2018; Guo et al., 2018). Especially, *S. suberectus* treatment displayed cytotoxicity in myeloma cells and leukemia cells by promoting reactive oxygen species stress and inducing apoptosis (Lim et al., 2019). Recent studies declared that *S. suberectus* manifested an inhibitory effect on breast cancer *via* arresting the cell cycle and declining lactate dehydrogenase (Wang et al., 2013). *S. suberectus* could also inhibit TNBC *in vitro* and *in vivo* through ROS-induced pyroptosis (Zhang et al., 2021). Interestingly, pure compounds isolated from it also possessed potent anti-cancer effects in breast cancer according to our previous study (Peng et al., 2019). Maackiain (MA, Figure 1) could be extracted from *S. suberectus*, *Sophora flavescens,* and many other Chinese herbal medicines (Tsai et al., 2020). MA exerted multiple pharmacologic activities, such as anti-allergic, anti-cancer, and anti-inflammatory effects (Aratanechemuge et al., 2004; Mizuguchi et al., 2015). Accordingly, it is interesting to explore the inhibitory capacity of MA on TNBC as a promising drug candidate.

In this study, it is the first report to declare the anti-cancer propriety of MA in TNBC. MA exhibits a significant repressive impact on TNBC cell proliferation, epithelial-mesenchymal transition (EMT), migration, and invasion. MA also blocks foci formation and tumor growth of TNBC. In addition, MA could promote growth arrest and DNA damage-inducible 45α (GADD45α) protein as well as mRNA expression through the downregulation of miR-374a, resulting in a proapoptotic consequence. Conversely, overexpressed miR-374a could at least partly reverse the apoptosis-inducing capacity of MA and decrease GADD45A expression induced by MA. Taken together, our work indicates MA is a promising anti-TNBC agent owing to its ability to modulate miR-374a.

## Materials and Methods

### Chemicals and Reagents

Reagents utilized in this study were obtained from typical companies. Specifically, the purity of MA reached 98% and was purchased from Chem Faces (Wuhan, China). The stocking solution of MA was dissolved in DMSO. When using MA to treat TNBC cells, the stocking solution was diluted until the percentage of DMSO was below 0.1%. Xylene, Eosin Y, Hematoxylin, and others were also purchased from typical companies such as Sigma (St. Louis, MO).

### Cell Culture

TNBC cell line MDA-MB-231 and BT549 from American Type Culture Collection (ATCC, United States) were incubated in a 5% CO_2_ 37°C incubator. A total of 293 T cells was also obtained from ATCC. FBS was obtained from Gibco (Life Technologies, United States). All the mediums, penicillin, and trypsin were obtained from Hyclone (Cytiva, United States). MDA-MB-231, BT549, and 293 T were cultured in DMEM medium. When culturing cells, 10% FBS, 1% penicillin, and 1% streptomycin were added in cell medium.

### CCK-8 Assay

TNBC cells were seeded in the 96 well plates at a concentration of 5×10^4^ cells/ml treated with different doses (0, 2.5, 5, 10, 20, 40, 80, 100 μM) of MA for 24 and 48 h. After MA treatment, 10 μL CCK-8 reagents were added to the 96 well plates according to the manufacturer’s instructions (MedChemExpress, United States). After incubation, the absorbance would be read by an ELISA plate reader at 450 nm. Triplicate experiments were performed independently.

### Foci Formation Assay

Foci formation assay would be conducted as previously (Peng et al., 2020). Briefly, clones were stained with 0.5% crystal violet for 15 min. Foci numbers were counted after staining. Triplicate experiments were performed independently.

### Flow Cytometry Analysis

Flow cytometry analysis would be conducted as previously (Peng et al., 2020). Briefly, FITC-conjugated Annexin V and PI staining were conducted through apoptosis analysis kit (BD Company, CA). After washing TNBC cells with cold PBS, the cells were resuspended in 1 × binding buffer at the concentration of 1 × 10 ^6^ cells/ml. Then, we gently mixed cells with Annexin V and PI for 15 min at room temperature in the dark. Finally, stained cells were analyzed by flow cytometry within 1 h. Triplicate experiments were performed independently.

### Chamber Invasion Assays

To determine the effect of MA on TNBC cell invasion, 6-well transwell chambers with Matrigel were utilized (Coring, United States). TNBC cells were planted at the upper compartment of the transwell plates at a concentration of 2.5×10^5^ cells/well. After being treated with MA (0, 1 μM, 2.5 μM, 5 μM) for 24 h, invading cells were stained through 0.5% crystal violet. The remaining cells were detected through microscope (ZEISS, Germany).

### Wound Scratch Assay

Wound scratch assays were performed as previously (Gong et al., 2018). Briefly, 3×10^5^ cells/ml of TNBC cells were plated into Culture-Insert (ibidi GmbH, Martinsried, Germany). After 24 h cell attachment, Culture-Insert was gently removed. After MA treatment (0, 1, 2.5 and 5 μM) for another 24 h, scratches were recorded at the beginning and the end point of the experiment through microscope (ZEISS, Germany).

### Western Blot

The protein in TNBC cells was extracted using cell lysis buffer (St. Louis, MO). There was 20-30 μg protein resolved on 10% SDS-PAGE gels and transferred onto PVDF membrane (Millipore, Merck, United States). We incubated sliced membranes and primary antibody against E-cadherin, N-cadherin, Vimentin, Bax, Bcl-2, GADD45α, GAPDH, and *β*-actin (Cell Signaling, Danvers, MA) overnight at 4°C. *β*-actin or GAPDH was selected as the loading control. After incubating sliced membranes and secondary antibody for 1 h, the detection was conducted *via* ECL Advance Reagent (Millipore, Merck, United States) and Image Lab Software (Bio-rad, Kidlington, UK).

### Quantitative Real-Time PCR

We extracted total RNA *via* RNAiso Plus (Takara, Shiga, Japan) reagent after exposure to MA for 24 h. The RNA transcription into cDNA was performed *via* PrimeScript RT Reagent Kit with gDNA Eraser (Takara, Shiga, Japan). The real-time PCR for amplification was conducted through TB Green (Takara, Shiga, Japan). MiRNA was extracted through miRNeasy Mini Kit from Qiagen (Germany) after treatment. The miRNA transcription into cDNA was performed *via* TaqMan MicroRNA Reverse Transcription Kit (Ambion, Life Technologies, United States). The real-time PCR for amplification was conducted through ExiLENT SYBR Green master mix (QIAGEN, United States). GAPDH and U6 were selected as the loading control, and 2^−ΔΔCT^ values were calculated. The following primers utilized for real time PCR were listed in Supplementary Table S1.

### PCR Array

PCR assay was performed *via* microRNA PCR Panel (Qiagen, Germany) as previously described (Peng et al., 2020). Briefly, miRNA in TNBC cells was extracted using miRNeasy Mini Kit from Qiagen (Germany) in different groups. We used TaqMan MicroRNA Reverse Transcription Kit from Ambion (Life Technologies, United States) to conduct miRNA transcription. We conducted real-time PCR for amplification corresponding cDNA using ExiLENT SYBR Green master mix.

### Cell Transfection

MirVana miRNA-374a mimic and mirVana miRNA mimic Negative Control were purchased from Ambion (Life Technologies, United States). We chose Lipofectamine RNAiMAX Transfection Reagent (Invitrogen, Life Technologies, United States) as transfection reagent. TNBC cells were plated on 6 well plates at a concentration of 2.5×10^5^ cells/ml. The complexes of miRNA and transfection reagent were added to TNBC cells. Then, the plates were incubated at 37°C in the cell incubator for at least 24 h.

### Luciferase Reporter Assay

The pMIR-REPOR miRNA Expression Reporter Vector System and Dual-Glo luciferase Assay System (Promega, Madison, WI) were utilized to verify the interaction between miR-374a and 3′UTR region of GADD45A. There are five groups, including the control group, miRNA mimic negative control + GADD45A 3′UTR wild type, miRNA-374a mimic + GADD45A 3′UTR wild type, miRNA mimic negative control + GADD45A 3′UTR mutated, and miRNA-374a mimic + GADD45A 3′UTR mutated, aiming at mimicking the modulation of miR-374a on GADD45A 3′UTR. The reagents were added based on the manufacturer’s instruction. The luciferase activity was recorded by SpectraMax M5. The primers for amplification are shown in Supplementary Table S2.

### Xenograft Tumor Growth Assays

Concisely, we conducted cell injection with 2×10^6^ MDA-MB-231 cells into the dorsal flanks to establish a TNBC xenograft tumor model. After model establishment, 4-week-old nude mice were separated into the control group, low dose group (MA, 25 mg/kg/d), and high dose group (MA, 50 mg/kg/d) randomly (*n* = 10). We administered MA to mice through intraperitoneal injection for 28 days with a record of tumor volumes. After 28-days administration, mice were sacrificed and tumor weights were assessed. The animal experiments were conducted complying with the institutional guidelines of Sichuan University and Chengdu University of Traditional Chinese Medicine.

### Immunohistochemistry

Immunohistochemistry (IHC) was utilized to detect GADD45α protein distribution and expression in tumor tissues. Tumor bulks were collected after nude mice were sacrificed at the end point of the animal study. The tumor tissues were deparaffinized and rehydrated, before antigen retrieval with sodium citrate 10 mM, pH 6.0 at a high temperature. Before slides were incubated with the primary antibody against GADD45α (Cell Signaling, Danvers, MA), the section was blocked in 5% goat serum for 1 h at room temperature. After rinsing with TBST three times, we incubated the slides with HRP secondary conjugates for 30 min at room temperature. SignalStain DAB Substrate Kit was adopted to determine GADD45α protein in tumor tissues. We used hematoxylin to stain the nuclei. The results were recorded through a Zeiss microscope Axio Lab A1.

### Data Analysis

We utilized GraphPad Prism 7.0 (Graph Pad Software, San Diego, CA) to conduct analyzing and visualizing data. The corresponding data were present as means ± standard deviations (SD). We used two-tailed student’s test and one-way ANOVA to assess the statistical significance of data (*p < 0.05, **p < 0.01, ^##^ *p* < 0.01).

## Results

### MA Exerts Cytotoxic Effects on TNBC Cells

**FIGURE 2:**
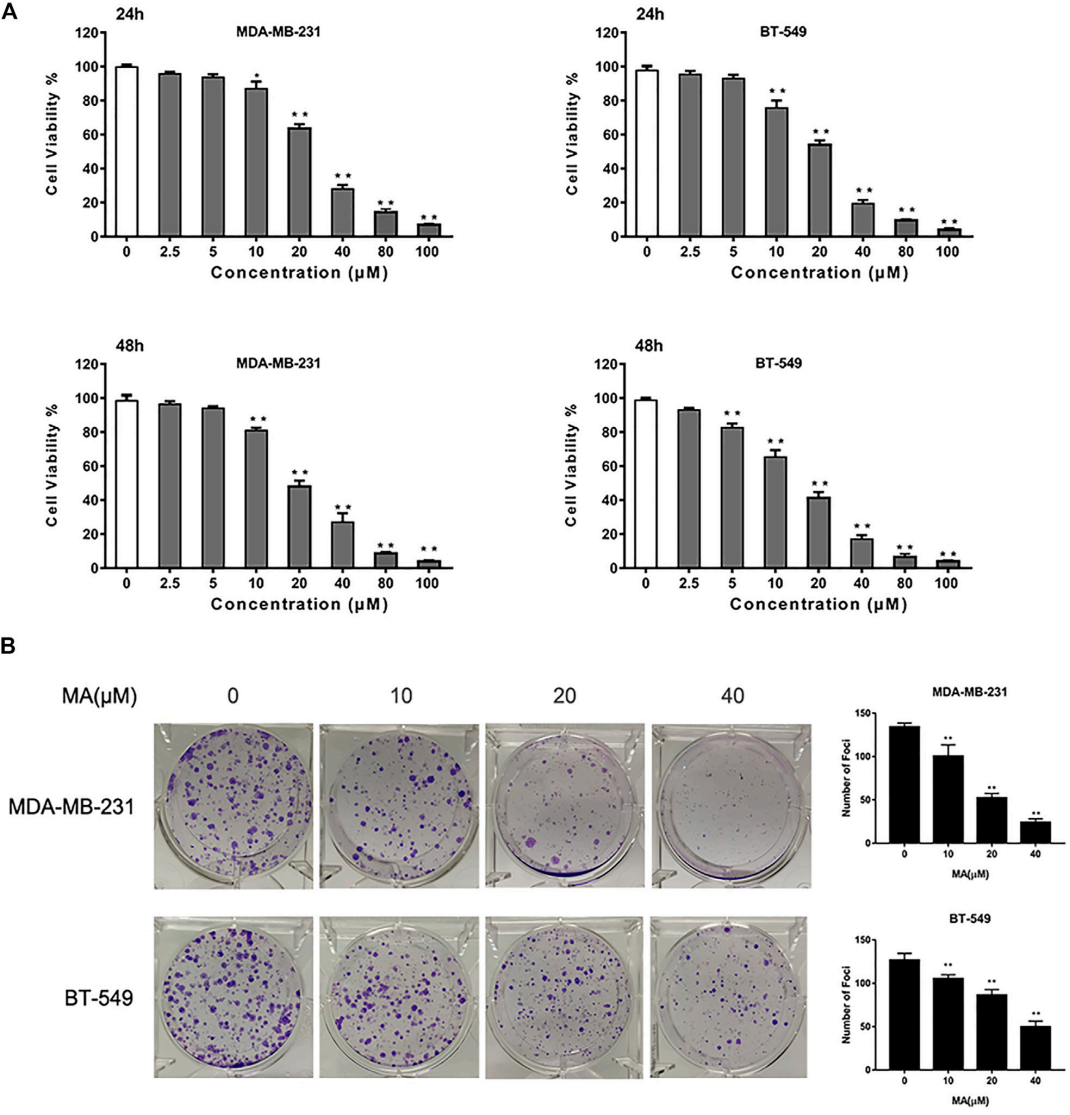
MA attenuates TNBC cell growth. **(A)** Cell viability of TNBC cells after MA treatment. **(B)** Representative images and number of foci formation in TNBC cells after exposure to MA for 24 h. Compared with the control group (0 μM), *p < 0.05, **p < 0.01.

To assess the inhibitory effect of MA on TNBC proliferation, we used a CCK-8 assay. After 24 h MA treatment, the cell viability was notably repressed above the concentration of 10 μM in BT549 and MDA-MB-231 cells. The IC_50_ values of MDA-MB-231 and BT549 cells were 25.24 and 20.99 μM, respectively (Supplementary Figure S1). MA could inhibit TNBC cell proliferation remarkedly at the concentration of 5 μM for 48 h treatment (*p* < 0.01). In the meantime, MA demonstrated a dose-dependent suppression of cell viability in TNBC cells (*p* < 0.01) (Figure 2A). The cytotoxic effect of MA on TNBC cells was further determined *via* foci formation assay. As shown in Figure 2B, MA could reduce the number of foci in MDA-MB-231 and BT549 cells after 24 h inference. Taken conjointly, MA has a repressive effect on TNBC cell growth and foci formation.

### MA Promotes Apoptosis in TNBC Cells

**FIGURE 3:**
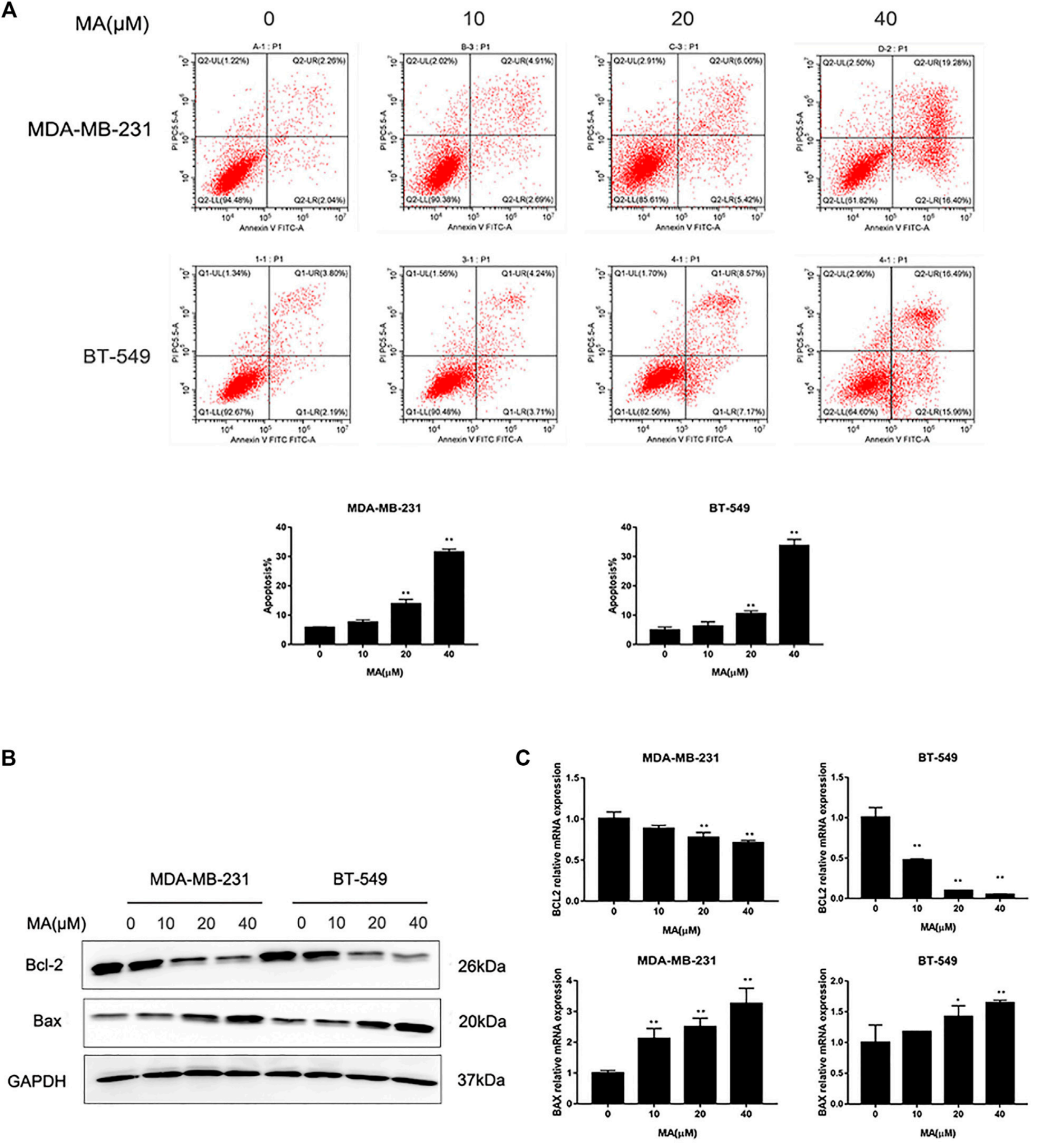
MA promotes TNBC cell apoptosis. **(A)** Representative images of flow cytometry and percentages of apoptotic cells in TNBC cells determined after MA treatment for 24 h. **(B)** Western blot analysis of Bax and Bcl-2 protein expression after 24 h MA treatment. **(C)** Real-time PCR analysis of BAX and BCL-2 mRNA expression after 24 h MA treatment. Compared with the control group (0 μM), **p < 0.01.

The results of Annexin V/PI staining apoptosis analysis showed that MA could induce an increase of a percentage of apoptotic cells after 24 h interference in TNBC cells (*p* < 0.01) (Figure 2A). The increasing percentage of apoptotic cells was consistent with the increasing of MA concentration. Additionally, MA could enhance Bax protein expression and reduce Bcl-2 protein expression (Figure 3B). Bax promotes mitochondrial apoptosis as a proapoptotic factor, while Bcl-2 restrains apoptosis progress as an anti-apoptotic factor. Meanwhile, MA could upregulate Bax mRNA expression and downregulate Bcl-2 mRNA expression (Figure 3C). These data signify that MA might inhibit TNBC cell proliferation by triggering mitochondrial-based apoptosis.

### MA Inhibits EMT in TNBC Cells

**FIGURE 4:**
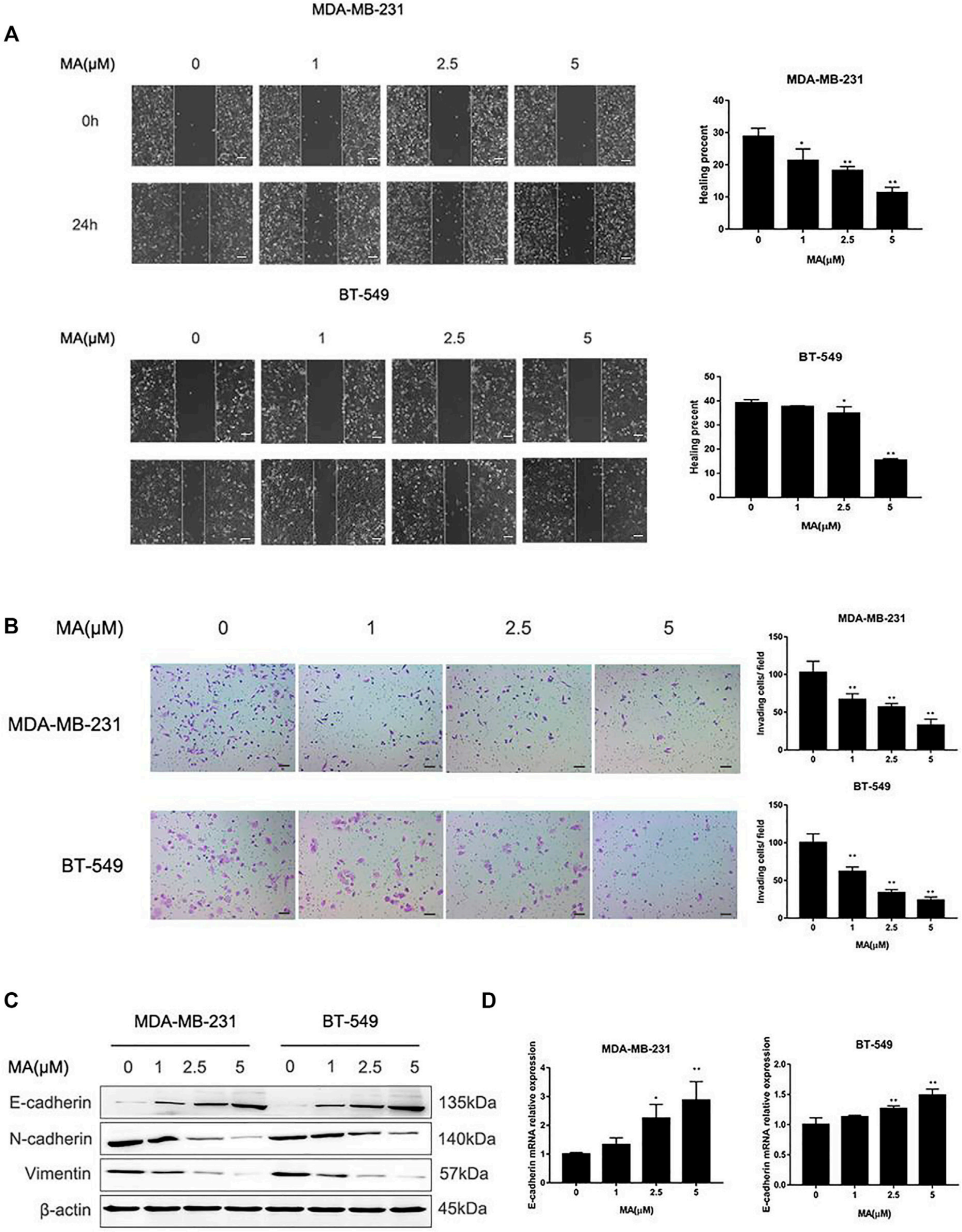
MA alleviates migration and invasion of TNBC cells. **(A)** Representative images of wound healing assay and healing percentage of TNBC cells after 24 h MA treatment. **(B)** Representative images of chamber invasion assay and number of invading cells after 24 h MA treatment. **(C)** Western blot analysis of EMT-related protein levels after 24 h MA intervention. **(D)** Real-time PCR analysis of E-cadherin after exposure to MA for 24 h. Compared with the control group (0 μM), *p < 0.05, **p < 0.01.

Based on the data from the CCK-8 assay, MA could not affect cell proliferation of BT549 and MDA-MB-231 cells under the concentration of 5 μM for 24 h treatment. Thus, we adopted 0, 1, 2.5, and 5 μM MA to conduct the study further about the suppressive effect of MA on TNBC cell mobile capacity. Wound healing assay exerted that after MA treatment for 24 h, the percentages of BT549 and MDA-MB-231 cells to moving into the wound area were significantly repressed, compared with the control group (Figure 4A). Similarly, invading cell numbers were dramatically abated after MA treatment for 24 h according to the results of a chamber invasion assay (Figure 4B). The EMT process plays an essential role in cancer cell migration and invasion. Mesenchymal phenotype markers (N-cadherin, Vimentin) protein expression was remarkably decreased, while epithelial phenotype marker (E-cadherin) protein expression was noticeably increased after MA interference in TNBC cells (Figure 4C). Meanwhile, MA-treated cells manifested a significant increase of E-cadherin mRNA (Figure 4D). These findings thus imply that MA hinders EMT progression to successively hamper the migratory and invasive abilities of TNBC cells.

### MA Suppresses TNBC Tumor Growth

**FIGURE 5:**
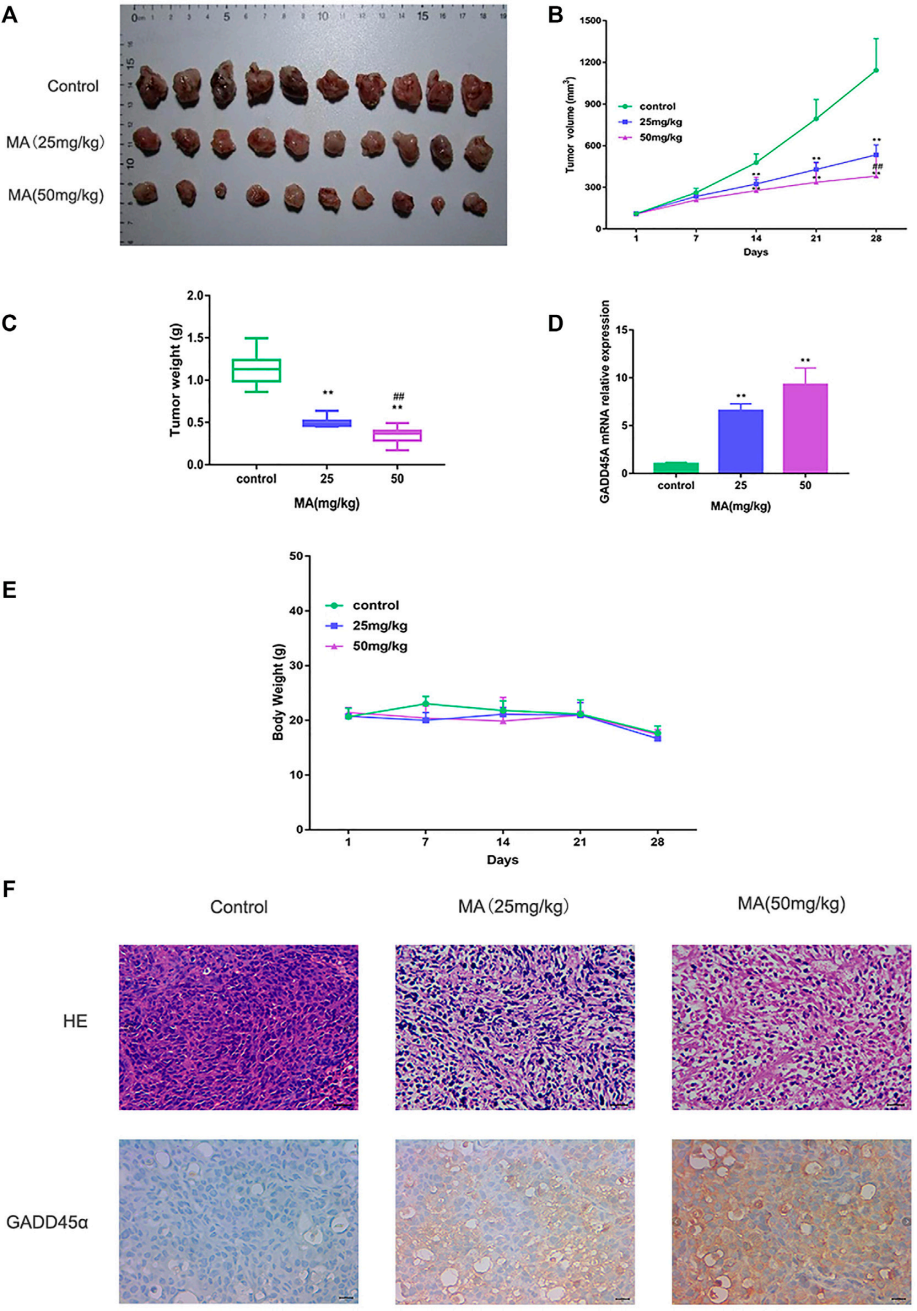
MA restrains TNBC tumor growth. **(A)** Collected tumor tissues after MA administration. **(B)** Tumor volumes in different groups during the experiment. **(C)** Tumor weights in different groups after MA administration. **(D)** Real-time PCR analysis of GADD45α mRNA levels in xenograft models at the end point. **(E)** Body weights of mice in different groups during the experiment. **(F)** Representative images of HE staining and IHC analysis of GADD45α after MA administration. Compared to the control group, **p < 0.01; compared to the low-dose group, ^##^ *p* < 0.01.

To investigate the suppressive effect of MA *in vivo*, MDA-MB-231 cells were injected into mammary fat pads of 4-week-old nude mice. After 28 days MA intraperitoneal injection, the mice were sacrificed, and the tumors were collected. The volumes and weights of tumors were recorded and compared. The results displayed that MA could reduce the volume and weights of tumors compared to the control group, and the high-dose group had a significantly more inhibitory effect on tumor growth than the low-dose group (Figures 5A–C). Interestingly, even the high dose of MA did not affect the body weights significantly compared to the control group (Figure 5E). HE staining demonstrated that in the control group, there were a large number of agglomerated and closely arranged tumor cells with deeply stained nuclei. In MA-treated groups, especially the high-dose group, the number of tumor cells decreased dramatically and the arrangement was relatively loose (Figure 5F). Taken together, MA also has a critical inhibitory effect on TNBC tumor growth *in vivo*.

### MA Downregulates Overexpressed miR-374a in TNBC Cells

**FIGURE 6:**
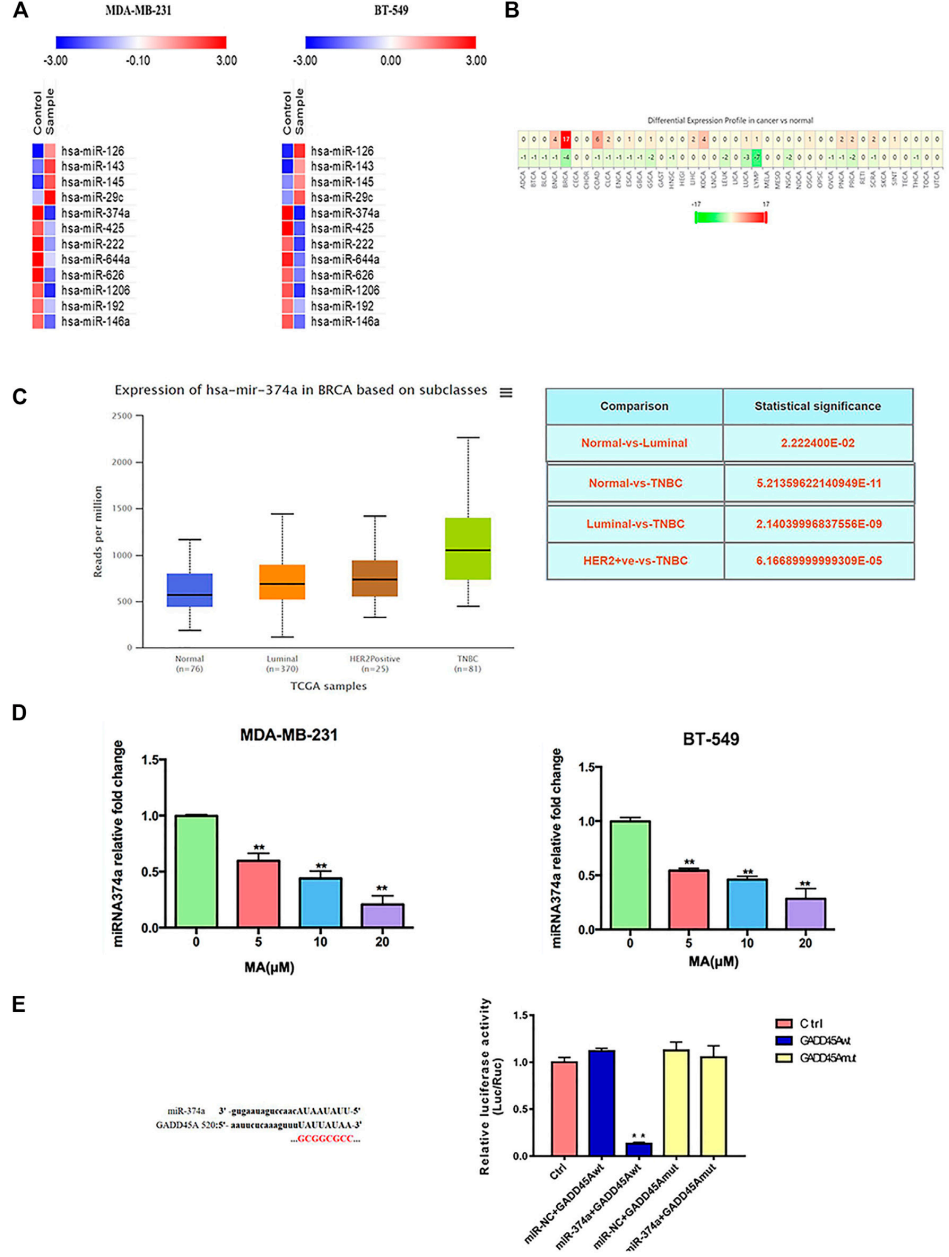
Overexpressed miR-374a is inhibited by MA in TNBC cells. **(A)** PCR array analysis of miRNAs variation in TNBC cells after 24 h MA intervention. **(B)** MiR-374a expression in different types of cancer tissues and the corresponding normal tissues. **(C)** MiR-374a expression in different types of breast cancer tissues and normal tissues. **(D)** Real-time PCR analysis of miR-374a expression in TNBC cells after 24 h MA intervention. **(E)** Relative luciferase activity determined through dual luciferase reporter assay in 293 T cells. Compared to the control group, **p < 0.01.

Accumulating evidence indicates that miRNAs play an essential role in cell proliferation and tumor growth in breast cancer. A PCR array was performed to screen the varied miRNA with at least 1.5-fold change after MA treatment. According to the results, miR-374a was notably abated in both cell lines after exposure to 20 μM MA for 24 h (Figure 6A). According to the clinical data from the dbDEMC database (https://www.biosino.org/dbDEMC/index), miR-374a was highly expressed in various cancers compared to their corresponding normal tissues, suggesting miR-374a as an oncogene (Figure 6B) (Yang et al., 2017). Besides, miR-374a was confirmed to express at a high level in basal-like breast cancer tissues (Supplementary Figure S2). Also, data from the TCGA database using UALCAN analysis (http://ualcan.path.uab.edu/analysis.html) showed that miR-374a was dramatically overexpressed in TNBC tissues compared to other molecular types of breast cancer (Figure 6C) (Chandrashekar et al., 2017). Utilizing real-time PCR, we confirmed that MA had an inhibitory effect on miR-374a of TNBC cells in a dose-dependent manner (Figure 6D). Collectively, these data manifest that MA inhibits oncogenic miR-374a in TNBC cells.

### MA Modulates miR-374a/GADD45α Axis

**FIGURE 7:**
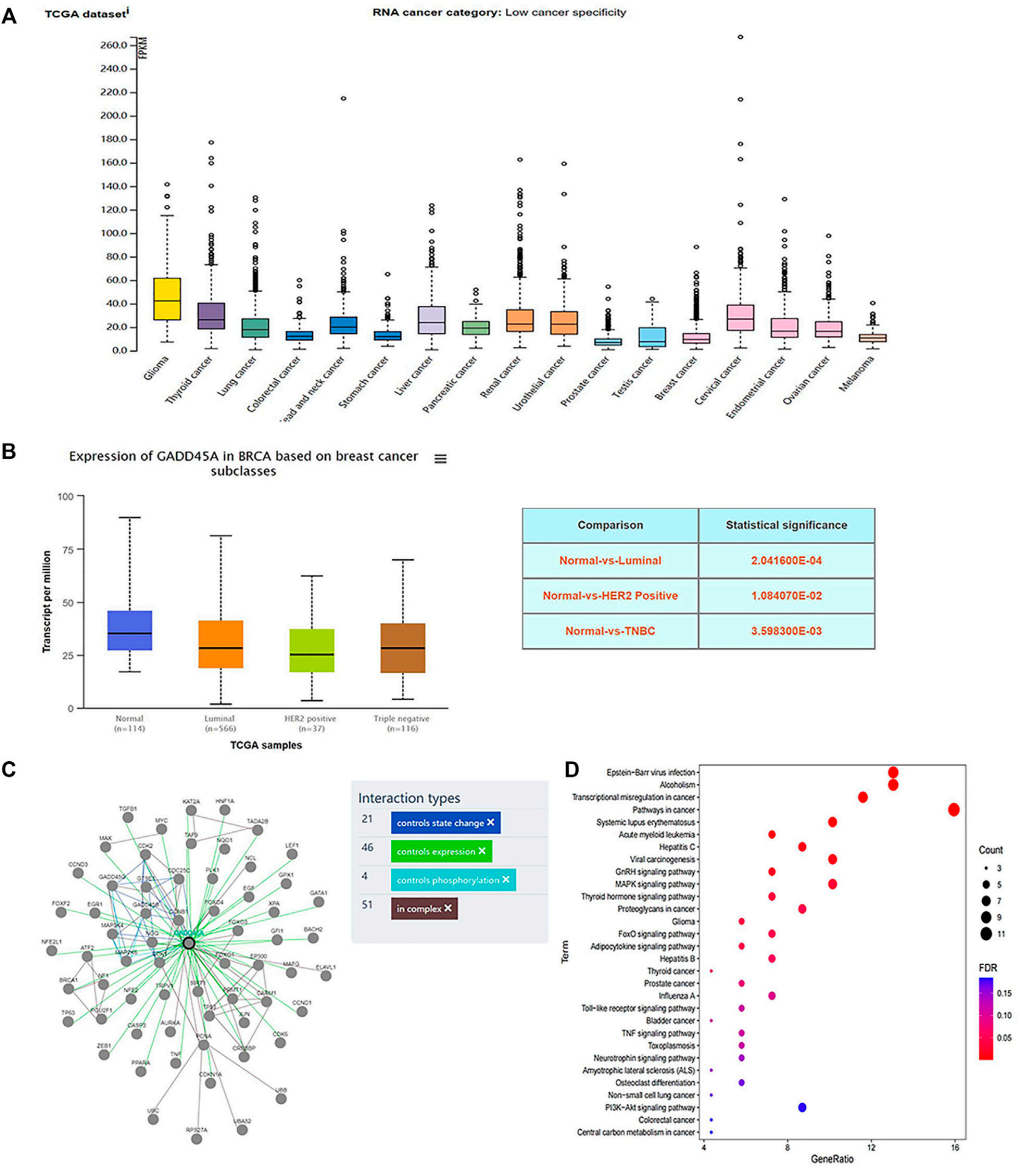
GADD45A is expressed at a low level in TNBC. **(A)** GADD45A expression in different types of cancer tissues. **(B)** GADD45A expression in different types of breast cancer tissues and normal tissues. **(C)** GADD45A is interacted with multiple genes. **(D)** KEGG annotation classification statistics of GADD45A-related genes.

**FIGURE 8:**
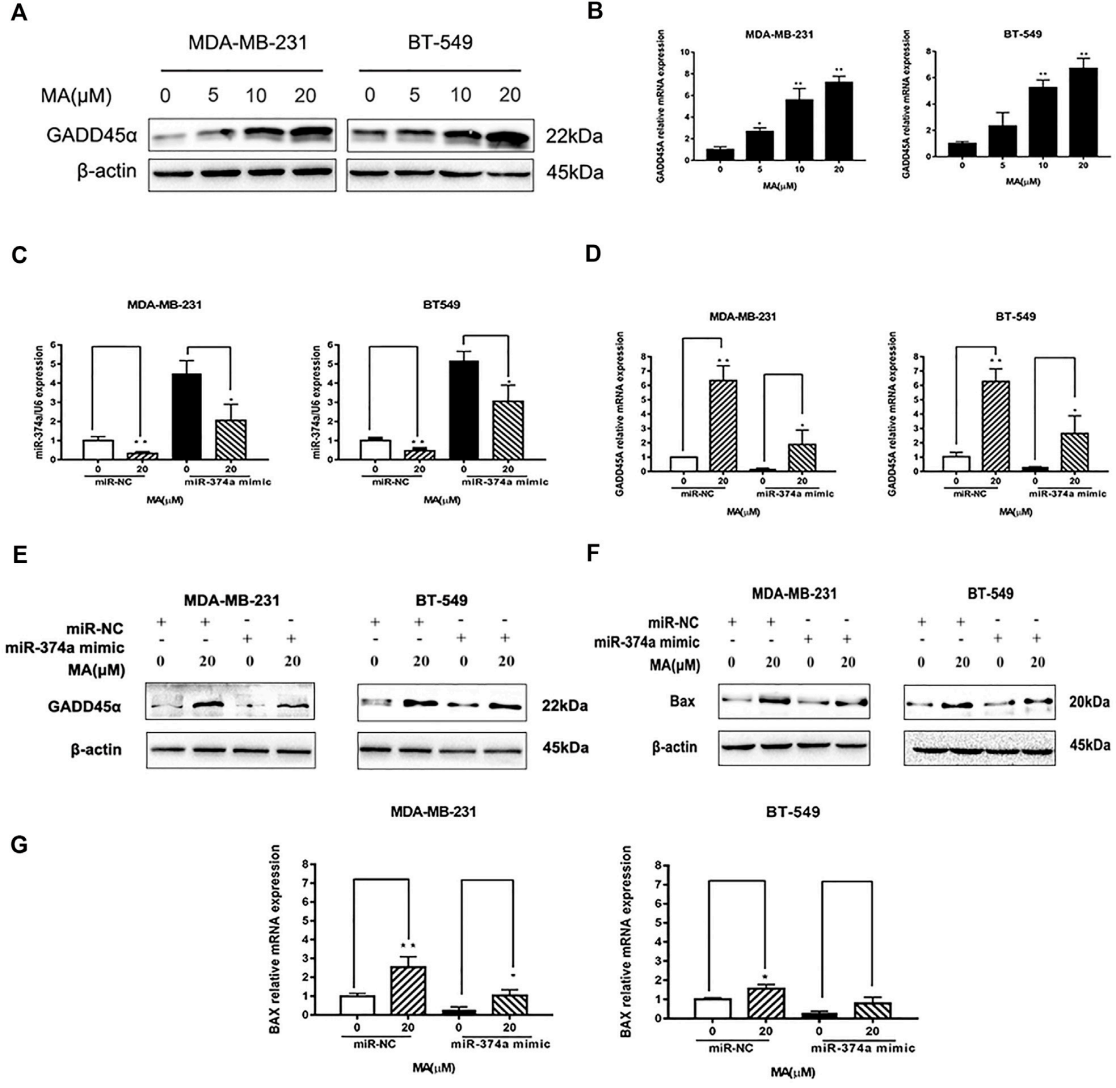
MA regulates miR-374a/GADD45A axis. **(A)** Western blot analysis of GADD45α after 24 h MA treatment. **(B)** Real-time PCR analysis of GADD45α mRNA expression after 24 h MA treatment. **(C)** Real-time PCR analysis of miR-374a expression in different groups of TNBC cell **(D)** Real-time PCR analysis of GADD45A mRNA expression in different groups of TNBC cells. **(E)** Western blot analysis of GADD45α protein expression in different groups of TNBC cells. **(F)** Western blot analysis of Bax protein expression in different groups of TNBC cells. **(G)** Real-time PCR analysis of BAX in different groups of TNBC cells. Compared with the control group (0 μM), *p < 0.05, **p < 0.01.

DIANA TOOLS (http://diana.imis.athena-innovation.gr/DianaTools/) predicted that GADD45A was one of the most potential targets of miR-374a (Supplementary Figure S3), and a present study suggested GADD45α as the downstream of miR-374a in a pheochromocytoma cell (Gong et al., 2018). Dual luciferase reporter assay confirmed that miR-374a could directly bind to the 3′UTR region of GADD45A (Figure 6E). Based on the data from the TCGA database using UALCAN analysis, GADD45A was lowly expressed in various types of cancer, including breast cancer. Moreover, GADD45A was dramatically expressed at a low level in TNBC, compared to normal tissues (Figures 7A,B). Pathway common (https://www.pathwaycommons.org/) exerted genes interacting with GADD45A (Supplementary Table S3), and KEGG annotation classification statistics of these genes indicated their role in cancer as well as MAPK pathway (Figures 7C,D). Further study displayed that MA could facilitate GADD45α mRNA and protein levels dose-dependently after 24 h treatment (Figures 8A,B). The *in vivo* results were inconsistent with *in vitro* findings. Determined by IHC staining, GADD45α was almost absent in tumor tissue and significantly increased in the MA high-dose group (Figure 5F). Results from real-time PCR exerted that GADD45A was lowly expressed in tumor tissues, and MA treatment could enhance GADD45A expression dose-dependently *in vivo* (Figure 5D). To evaluate the effect of miR-374a downregulated by MA on GADD45A, we transfected BT-549 and MDA-MB-231 cells with miRNA mimic negative control and miR-374a mimic, and the successful transfection was confirmed by real-time PCR (Figure 8C). GADD45α mRNA and protein expression were detected after miR-374a mimic transfection and MA treatment. As shown in Figures 8D,E, MA could significantly promote GADD45α mRNA and protein levels at the concentration of 20 μM, and the effect was at least partly blocked after the miR-374a mimic pretreatment. Besides, Western blot results demonstrated that MA could promote Bax expression, and the upregulation of miR-374a would at least partly reverse the effect of MA (Figure 8F). As for BAX mRNA expression, the miR-374a mimic exerted a similar effect even after MA treatment (Figure 8G). Altogether, MA might activate GADD45α and related genes through downregulation of miR-374a in TNBC cells.

## Discussion

Breast cancer is the most common cancer occurring in women worldwide, with increasing incidence. TNBC belongs to the most aggressive subtype of breast cancer, always accompanying worse clinical outcome (Feng Wang et al., 2021). Despite improvement in breast cancer therapeutic measures, the treatment for TNBC clinically without obvious side effects is still a huge challenge (Ghosh et al., 2021). Searching for complementary and alternative medicine is critical for the advancement of current TNBC therapies. Presently, TCM, with multiple biological capacities, gains credibility and reputation as a source of potential anti-cancer reagents (Yao et al., 2021). According to our previous work, several pure compounds isolated from *S. suberectus* could inhibit breast cancer (Peng et al., 2020; Peng et al., 2021b). We found that MA, extracted from *S. suberectus*, could also suppress the proliferative, migratory, and invasive abilities of TNBC cells (Figures 2, 3, 4). Apart from *in vitro* study, results from animal experiments further confirmed its anti-TNBC effect (Figure 5). Consequently, our study first clarified the anti-breast cancer property of MA. Meanwhile, it is interesting that we found that MA could downregulate miR-374a (Figure 6), an oncogenic regulator in TNBC cells promoting cell proliferation, migration, and invasion (Cai et al., 2013; Du et al., 2019; Son et al., 2019).

Triggering apoptosis relied on the balance between prosurvival and proapoptotic BCL-2 protein family members (Diepstraten et al., 2021). A recent study pointed out that DNA damage and oncogene deregulation could contribute to the activation of BCL-2-family-regulated pathway (Singh et al., 2019). Interestingly, miR-374a could act as an oncogenic factor in various types of cancer, and the down-regulation of miR-374a frequently led to stimulating apoptosis (Peng et al., 2021a). Our study exerted the proapoptotic effect of MA with a decrease of miR-374a, and miR-374a could directly bind to the 3′UTR region of GADD45A (Figures 8, 5, 6). GADD45A belongs to the growth arrest and DNA damage-inducible 45 family, mainly affecting cell cycle and interacting with cell growth modulators (Sytnikova et al., 2011). Furthermore, GADD45A had an inhibitory effect on cell cycle and cell proliferation through the regulation of P53 in bladder cancer (Han et al., 2019). More recent studies indicated GADD45A could even act as a tumor suppressive gene by facilitating apoptosis, maintaining cell-to-cell adhesion, inhibiting angiogenesis, modulating drug resistance, suppressing metastasis, promoting DNA repair, and stabilizing genomics (Yang et al., 2013; Bartoszewski et al., 2020; Runtian Wang et al., 2021). GADD45A was gradually considered as a promising target for tumor malignancy (Lin et al., 2022). More recent studies made an effort to prove that natural compounds could promote apoptosis through the activation of the cellular function of GADD45A (Jang et al., 2021). Meanwhile, MA could upregulate both GADD45α protein and mRNA expression, and overexpressing miR-374a would at least partly block the effect (Figure 8). These findings suggested the modulatory effect of MA in the mitochondria apoptotic signaling with the function to promote GADD45A through the inhibition of miR-374a in TNBC.

In addition to accelerating tumor growth, miR-374a is attributed to epithelial cells shifting into mesenchymal stem cells, namely, EMT process in cancer (Ma et al., 2019). In cancer cells, EMT initiates migration and invasion with the increase of more aggressive capacities (Bakir et al., 2020). EMT initiation is based on E-cadherin to N-cadherin switch, resulting in lessening cell-cell adhesion and gaining mobile ability (Biswas, 2020). Intriguingly, MA could upregulate E-cadherin and downregulate N-cadherin, accompanying a decrease of migratory and invasive TNBC cell numbers (Figure 4). In the meantime, MA could diminish miR-374a expression (Figure 6). These data first implied the role of MA as the mediator of the EMT process in TNBC cells.

## Conclusion

TNBC cells tend to be more aggressive and EMT-activated than other molecular types of breast cancer. Our study demonstrated that MA had an inhibitory effect on TNBC proliferation, migration, invasion, and tumor growth as a promising drug candidate. Interestingly, MA could significantly repress miR-374a, which was usually considered as an oncogene regulating cancer initiation and development. Moreover, MA could promote GADD45α protein and mRNA expression through the downregulation of miR-374a. Further study confirmed that miR-374a directly targeted the 3′UTR region of GADD45A. Furthermore, the proapoptotic effect of MA was also negatively correlated to the expression of miR-374a. These findings first reported the anti-breast cancer effect of MA, pointed out GADD45A as the direct target of miR-374a in breast cancer, and partly offered evidence for *S. suberectus* as a resource of novel drug candidates in TNBC therapy.

## Data Availability

Datasets generated for this article can be found in [TCGA] using the accession number TCGA-BRCA. The data are analyzed using UALCAN (http://ualcan.path.uab.edu/analysis.html).

## References

[B1] An J. Peng C. Tang H. Liu X. Peng F. (2021). New Advances in the Research of Resistance to Neoadjuvant Chemotherapy in Breast Cancer. Ijms 22, 9644. 10.3390/ijms22179644 34502549 PMC8431789

[B2] Aratanechemuge Y. Hibasami H. Katsuzaki H. Imai K. Komiya T. (2004). Induction of Apoptosis by Maackiain and Trifolirhizin (Maackiain Glycoside) Isolated from Sanzukon (Sophora Subprostrate Chen et T. Chen) in Human Promyelotic Leukemia HL-60 cells. Oncol. Rep. 12, 1183–1188. 10.3892/or.12.6.1183 15547735

[B3] Bakir B. Chiarella A. M. Pitarresi J. R. Rustgi A. K. (2020). EMT, MET, Plasticity, and Tumor Metastasis. Trends Cel. Biol. 30, 764–776. 10.1016/j.tcb.2020.07.003 PMC7647095 32800658

[B16] Bartoszewski R. Gebert M. Janaszak-Jasiecka A. Cabaj A. Króliczewski J. Bartoszewska S. (2020). Genome-Wide mRNA Profiling Identifies RCAN1 and GADD45A as Regulators of the Transitional Switch from Survival to Apoptosis During ER Stress. FEBS J. 287, 2923–2947. 10.1111/febs.15195 31880863

[B4] Biswas K. H. (2020). Molecular Mobility-Mediated Regulation of E-Cadherin Adhesion. Trends Biochem. Sci. 45, 163–173. 10.1016/j.tibs.2019.10.012 31810601

[B5] Cai J. Guan H. Fang L. Yang Y. Zhu X. Yuan J. (2013). MicroRNA-374a Activates Wnt/β-Catenin Signaling to Promote Breast Cancer Metastasis. J. Clin. Invest. 123, 566–579. 10.1172/jci65871 23321667 PMC3561816

[B6] Chandrashekar D. S. Bashel B. Balasubramanya S. A. H. Creighton C. J. Ponce-Rodriguez I. Chakravarthi B. V. S. K. (2017). UALCAN: A Portal for Facilitating Tumor Subgroup Gene Expression and Survival Analyses. Neoplasia 19, 649–658. 10.1016/j.neo.2017.05.002 28732212 PMC5516091

[B7] Diepstraten S. T. Anderson M. A. Czabotar P. E. Lessene G. Strasser A. Kelly G. L. (2021). The Manipulation of Apoptosis for Cancer Therapy Using BH3-Mimetic Drugs. Nat. Rev. Cancer 22, 45–64. 10.1038/s41568-021-00407-4 34663943

[B8] Do M. Hur J. Choi J. Kim Y. Park H.-Y. Ha S. (2018). Spatholobus Suberectus Ameliorates Diabetes-Induced Renal Damage by Suppressing Advanced Glycation End Products in Db/db Mice. Ijms 19, 2774. 10.3390/ijms19092774 PMC6163801 30223524

[B9] Du S. Hu W. Zhao Y. Zhou H. Wen W. Xu M. (2019). Long Non-coding RNA MAGI2-AS3 Inhibits Breast Cancer Cell Migration and Invasion *via* Sponging microRNA-374a. Cancer Biomark. 24, 269–277. 10.3233/cbm-182216 30883342 PMC13082510

[B10] Wang F. Kong L. Pu Y. Chao F. Zang C. Qin W. (2021). Long Noncoding RNA DICER1-AS1 Functions in Methylation Regulation on the Multi-Drugresistance of Osteosarcoma Cells *via* miR-34a-5p and GADD45A. Front. Oncol. 11, 685881. 10.3389/fonc.2021.685881 34307152 PMC8299526

[B11] Fu Y. F. Jiang L. H. Zhao W. D. Xi-Nan M. Huang S. Q. Yang J. (2017). Immunomodulatory and Antioxidant Effects of Total Flavonoids of Spatholobus Suberectus Dunn on PCV2 Infected Mice. Sci. Rep. 7, 8676. 10.1038/s41598-017-09340-9 28819143 PMC5561176

[B12] Ghosh S. Javia A. Shetty S. Bardoliwala D. Maiti K. Banerjee S. (2021). Triple Negative Breast Cancer and Non-small Cell Lung Cancer: Clinical Challenges and Nano-Formulation Approaches. J. Control. Release 337, 27–58. 10.1016/j.jconrel.2021.07.014 34273417

[B13] Gong W. Qie S. Huang P. Xi J. (2018). Protective Effect of miR-374a on Chemical Hypoxia-Induced Damage of PC12 Cells *In Vitro* via the GADD45α/JNK Signaling Pathway. Neurochem. Res. 43, 581–590. 10.1007/s11064-017-2452-0 29247275

[B14] Guo W.-L. Wang W.-H. Hu W.-T. Xie Z.-Y. Wang S.-F. Sun Y. (2018). Antibacterial Synergisms of Ji Xue Teng, Spatholobus Suberectus , Extract and Selected Antibiotics against Streptococcus Agalactiae from Nile Tilapia, *Oreochromis niloticus* (L.), *In Vitro* and *In Vivo* . J. World Aquacult Soc. 49, 1002–1013. 10.1111/jwas.12516

[B15] Han N. Yuan F. Xian P. Liu N. Liu J. Zhang H. (2019). GADD45a Mediated Cell Cycle Inhibition Is Regulated by P53 in Bladder Cancer. Onco Targets Ther. 12, 7591–7599. 10.2147/ott.S222223 31571910 PMC6754676

[B17] Iorio M. V. Ferracin M. Liu C. G. Veronese A. Spizzo R. Sabbioni S. (2005). MicroRNA Gene Expression Deregulation in Human Breast Cancer. Cancer Res. 65, 7065–7070. 10.1158/0008-5472.Can-05-1783 16103053

[B18] Jang H.-J. Yang J. H. Hong E. Jo E. Lee S. Lee S. (2021). Chelidonine Induces Apoptosis via GADD45a-P53 Regulation in Human Pancreatic Cancer Cells. Integr. Cancer Ther. 20, 153473542110061. 10.1177/15347354211006191 PMC8077490 33884928

[B19] Lim H. Park M. Kim C. Kang B. Song H.-S. Lee H. (2019). MiR-657/ATF2 Signaling Pathway Has a Critical Role in Spatholobus Suberectus Dunn Extract-Induced Apoptosis in U266 and U937 Cells. Cancers 11, 150. 10.3390/cancers11020150 PMC6406694 30696076

[B20] Lin H.-Y. Wu H.-J. Chen S.-Y. Hou M.-F. Lin C.-S. Chu P.-Y. (2022). Epigenetic Therapy Combination of UNC0638 and CI-994 Suppresses Breast Cancer via Epigenetic Remodeling of BIRC5 and GADD45A. Biomed. Pharmacother. 145, 112431. 10.1016/j.biopha.2021.112431 34798471

[B21] Ma L. Shao Z. Zhao Y. (2019). MicroRNA-374a Promotes Pancreatic Cancer Cell Proliferation and Epithelial to Mesenchymal Transition by Targeting SRCIN1. Pathol. - Res. Pract. 215, 152382. 10.1016/j.prp.2019.03.011 30890278

[B23] Mendell J. Olson E. (2012). MicroRNAs in Stress Signaling and Human Disease. Cell 148, 1172–1187. 10.1016/j.cell.2012.02.005 22424228 PMC3308137

[B22] Mizuguchi H. Nariai Y. Kato S. Nakano T. Kanayama T. Kashiwada Y. (2015). Maackiain Is a Novel Antiallergic Compound that Suppresses Transcriptional Upregulation of the Histamine H 1 Receptor and Interleukin‐4 Genes. Pharmacol. Res. Perspect. 3, e00166. 10.1002/prp2.166 26516579 PMC4618638

[B24] Peng F. Zhu H. Meng C.-W. Ren Y.-R. Dai O. Xiong L. (2019). New Isoflavanes from Spatholobus Suberectus and Their Cytotoxicity against Human Breast Cancer Cell Lines. Molecules 24, 3218. 10.3390/molecules24183218 PMC6766798 31487934

[B25] Peng F. Xiong L. Peng C. (2020). (-)-Sativan Inhibits Tumor Development and Regulates miR-200c/PD-L1 in Triple Negative Breast Cancer Cells. Front. Pharmacol. 11, 251. 10.3389/fphar.2020.00251 32231566 PMC7082844

[B26] Peng F. Tang H. Du J. Chen J. Peng C. (2021b). Isoliquiritigenin Suppresses EMT-Induced Metastasis in Triple-Negative Breast Cancer through miR-200c/C-JUN/β-Catenin. Am. J. Chin. Med. 49, 505–523. 10.1142/s0192415x21500233 33641651

[B27] Peng F. Fan H. Li S. Peng C. Pan X. (2021a). MicroRNAs in Epithelial-Mesenchymal Transition Process of Cancer: Potential Targets for Chemotherapy. Ijms 22, 7526. 10.3390/ijms22147526 34299149 PMC8305963

[B28] Qin S. Wu L. Wei K. Liang Y. Song Z. Zhou X. (2019). A Draft Genome for Spatholobus Suberectus. Sci. Data 6, 113. 10.1038/s41597-019-0110-x 31273216 PMC6609623

[B29] Wang R. Xu K. Gao F. Huang J. Guan X. (2021). Clinical Considerations of CDK4/6 Inhibitors in Triple-Negative Breast Cancer. Biochim. Biophys. Acta (Bba) - Rev. Cancer 1876, 188590. 10.1016/j.bbcan.2021.188590 34271137

[B30] Singh R. Letai A. Sarosiek K. (2019). Regulation of Apoptosis in Health and Disease: the Balancing Act of BCL-2 Family Proteins. Nat. Rev. Mol. Cel. Biol. 20, 175–193. 10.1038/s41580-018-0089-8 PMC7325303 30655609

[B31] Sinha A. Agarwal S. Parashar D. Verma A. Saini S. Jagadish N. (2013). Down Regulation of SPAG9 Reduces Growth and Invasive Potential of Triple-Negative Breast Cancer Cells: Possible Implications in Targeted Therapy. J. Exp. Clin. Cancer Res. 32, 69. 10.1186/1756-9966-32-69 24330581 PMC3848771

[B32] Son D. Kim Y. Lim S. Kang H. G. Kim D. H. Park J. W. (2019). miR-374a-5p Promotes Tumor Progression by Targeting ARRB1 in Triple Negative Breast Cancer. Cancer Lett. 454, 224–233. 10.1016/j.canlet.2019.04.006 31004703

[B33] Sytnikova Y. A. Kubarenko A. V. Schäfer A. Weber A. N. R. Niehrs C. (2011). Gadd45a Is an RNA Binding Protein and Is Localized in Nuclear Speckles. PLoS One 6, e14500. 10.1371/journal.pone.0014500 21249130 PMC3017548

[B34] Tang Q. Ouyang H. He D. Yu C. Tang G. (2019). MicroRNA-Based Potential Diagnostic, Prognostic and Therapeutic Applications in Triple-Negative Breast Cancer. Artif. Cell Nanomed. Biotechnol. 47, 2800–2809. 10.1080/21691401.2019.1638791 31284781

[B35] Tsai R.-T. Tsai C.-W. Liu S.-P. Gao J.-X. Kuo Y.-H. Chao P.-M. (2020). Maackiain Ameliorates 6-Hydroxydopamine and SNCA Pathologies by Modulating the PINK1/Parkin Pathway in Models of Parkinson's Disease in *Caenorhabditis elegans* and the SH-SY5Y Cell Line. Ijms 21, 4455. 10.3390/ijms21124455 PMC7352553 32585871

[B36] Wang Z. Wang D. Han S. Wang N. Mo F. Loo T. Y. (2013). Bioactivity-Guided Identification and Cell Signaling Technology to Delineate the Lactate Dehydrogenase A Inhibition Effects of Spatholobus Suberectus on Breast Cancer. PLoS One 8, e56631. 10.1371/journal.pone.0056631 23457597 PMC3572989

[B37] Yang F. Zhang W. Li D. Zhan Q. (2013). Gadd45a Suppresses Tumor Angiogenesis via Inhibition of the mTOR/STAT3 Protein Pathway. J. Biol. Chem. 288, 6552–6560. 10.1074/jbc.M112.418335 23329839 PMC3585088

[B38] Yang Z. Wu L. Wang A. Tang W. Zhao Y. Zhao H. (2017). dbDEMC 2.0: Updated Database of Differentially Expressed miRNAs in Human Cancers. Nucleic Acids Res. 45, D812–D818. 10.1093/nar/gkw1079 27899556 PMC5210560

[B39] Yao C. L. Zhang J. Q. Li J. Y. Wei W. L. Wu S. F. Guo D. A. (2021). Traditional Chinese Medicine (TCM) as a Source of New Anticancer Drugs. Nat. Prod. Rep. 38, 1618–1633. 10.1039/d0np00057d 33511969

[B40] Zhang J. He Y. Yu Y. Chen X. Cui G. Wang W. (2018). Upregulation of miR-374a Promotes Tumor Metastasis and Progression by Downregulating LACTB and Predicts Unfavorable Prognosis in Breast Cancer. Cancer Med. 7, 3351–3362. 10.1002/cam4.1576 PMC6051141 29790671

[B41] Zhang F. Liu Q. Ganesan K. Kewu Z. Shen J. Gang F. (2021). The Antitriple Negative Breast Cancer Efficacy of *Spatholobus Suberectus* Dunn on ROS-Induced Noncanonical Inflammasome Pyroptotic Pathway. Oxid Med. Cel Longev 2021, 5187569. 10.1155/2021/5187569 PMC8514942 34659633

