# Supplementary figures and images for: Maackiain Modulates miR-374a/GADD45A Axis to Inhibit Triple-Negative Breast Cancer Initiation and Progression (part 1 of 2)
Source: Front Pharmacol. 2022 Mar 4;13:806869. doi: 10.3389/fphar.2022.806869 (PMC8930825; doi:10.3389/fphar.2022.806869)

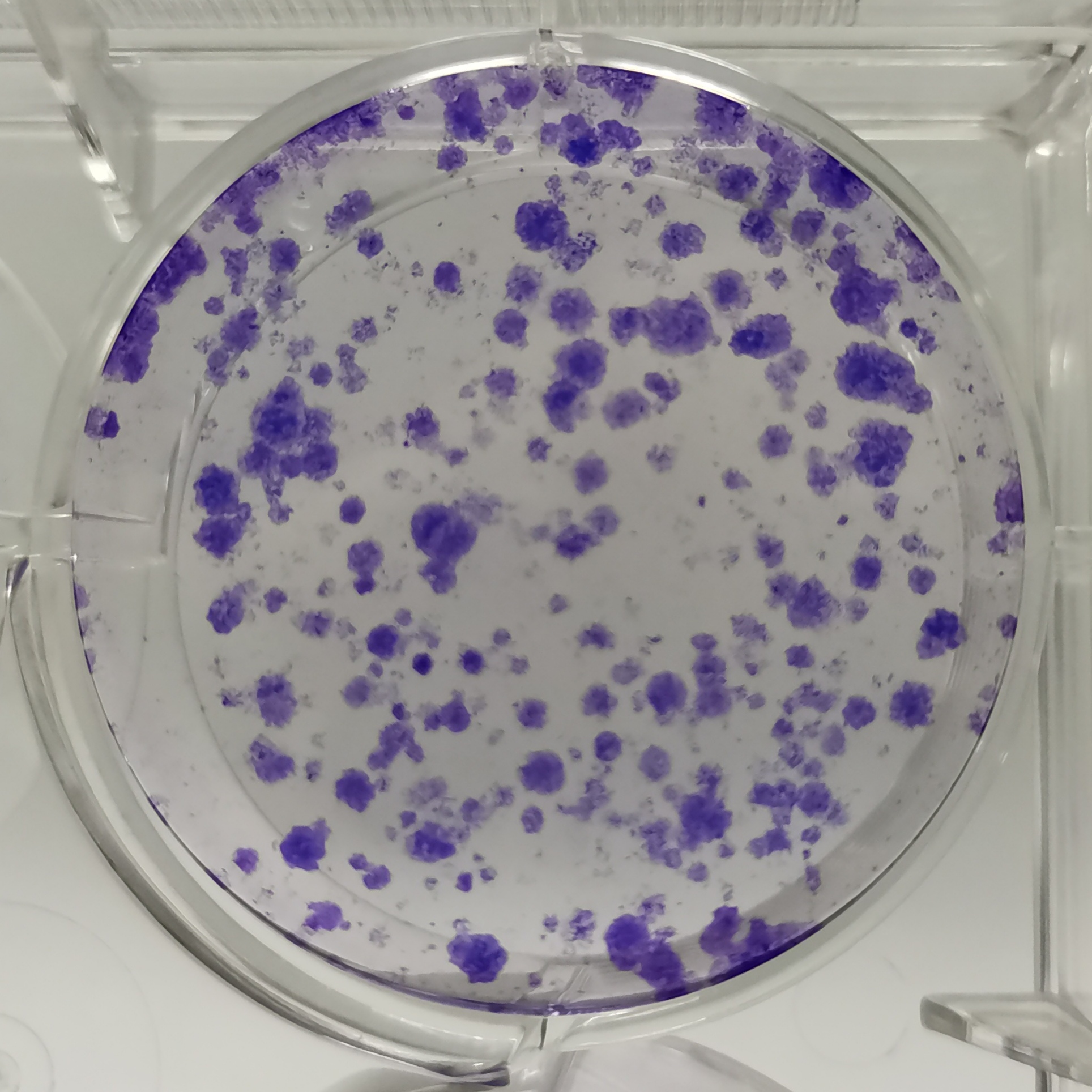

Supplement: Supplementary file 1 [file DataSheet3.ZIP › Clone formation assay/BT-549/5 (1).jpg]

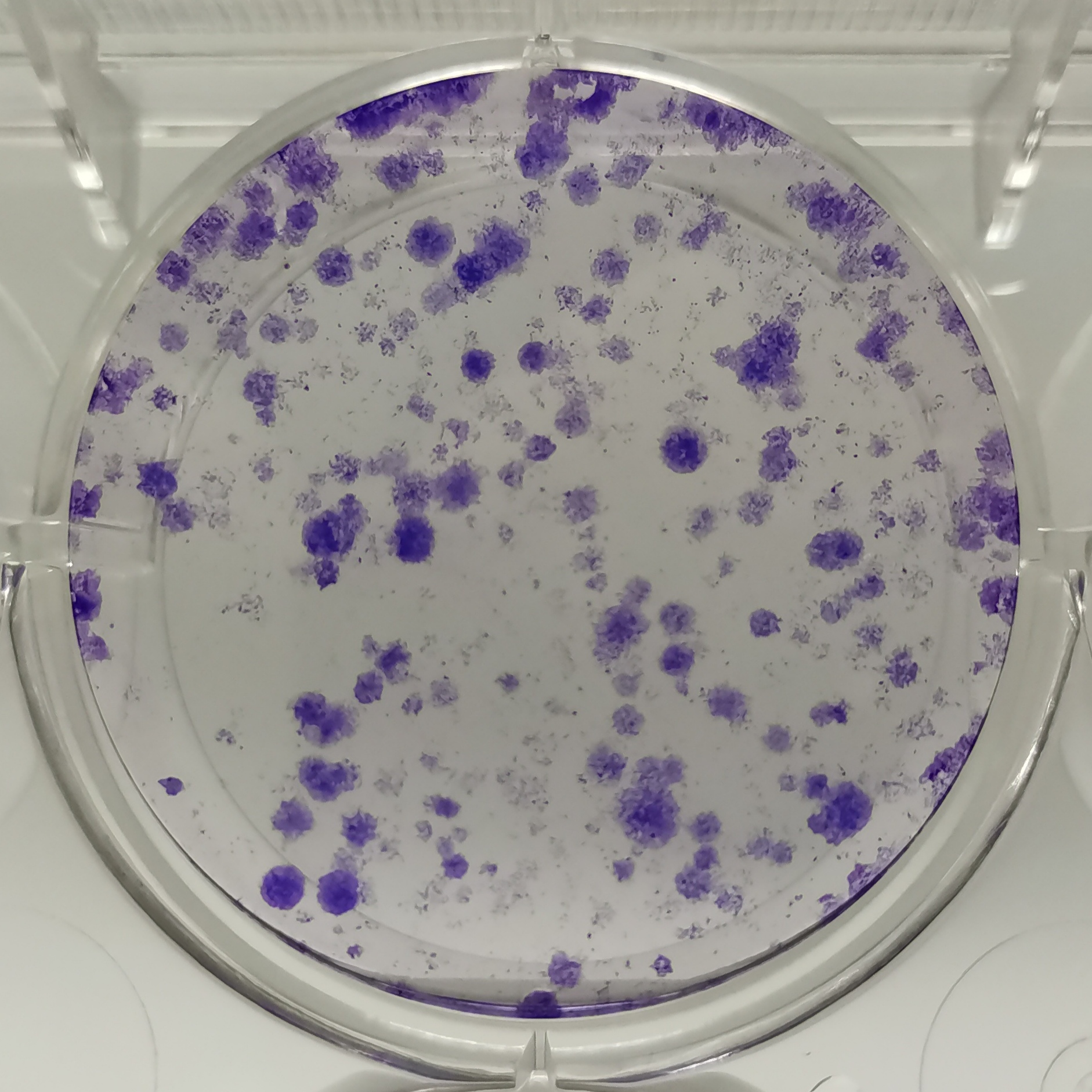

Supplement: Supplementary file 1 [file DataSheet3.ZIP › Clone formation assay/BT-549/5 (2).jpg]

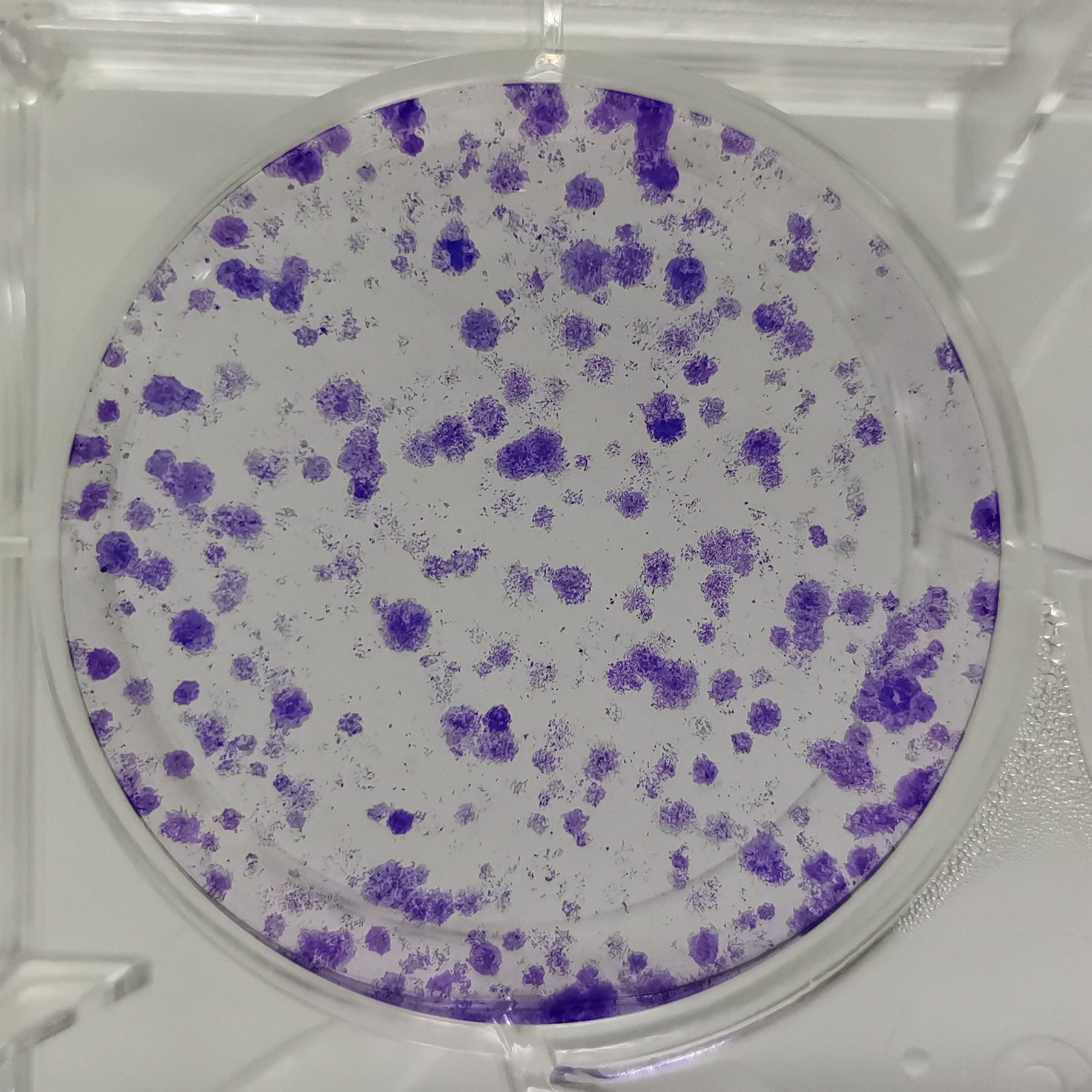

Supplement: Supplementary file 1 [file DataSheet3.ZIP › Clone formation assay/BT-549/5 (3)-1.jpg]

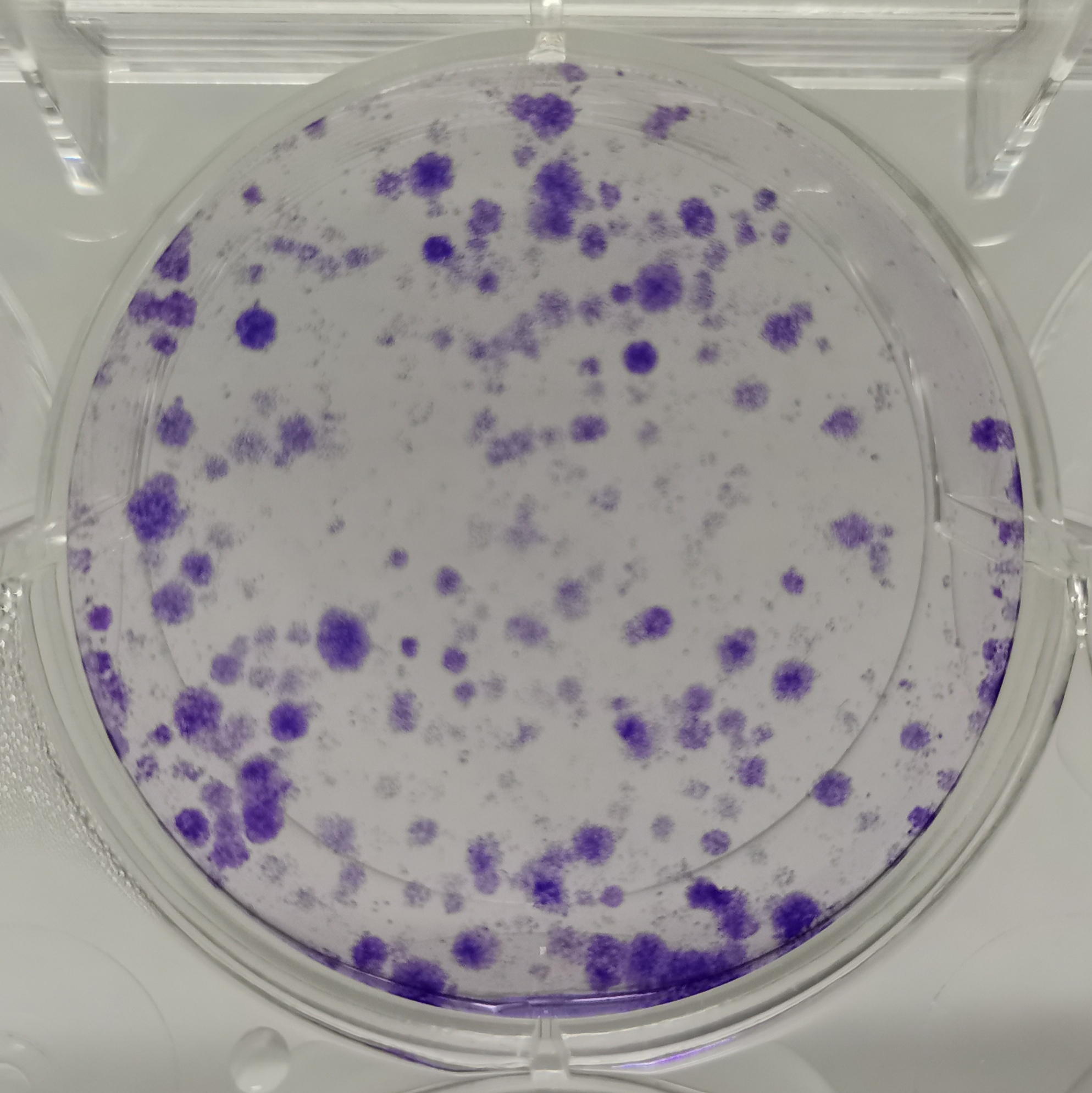

Supplement: Supplementary file 1 [file DataSheet3.ZIP › Clone formation assay/BT-549/6 (1).jpg]

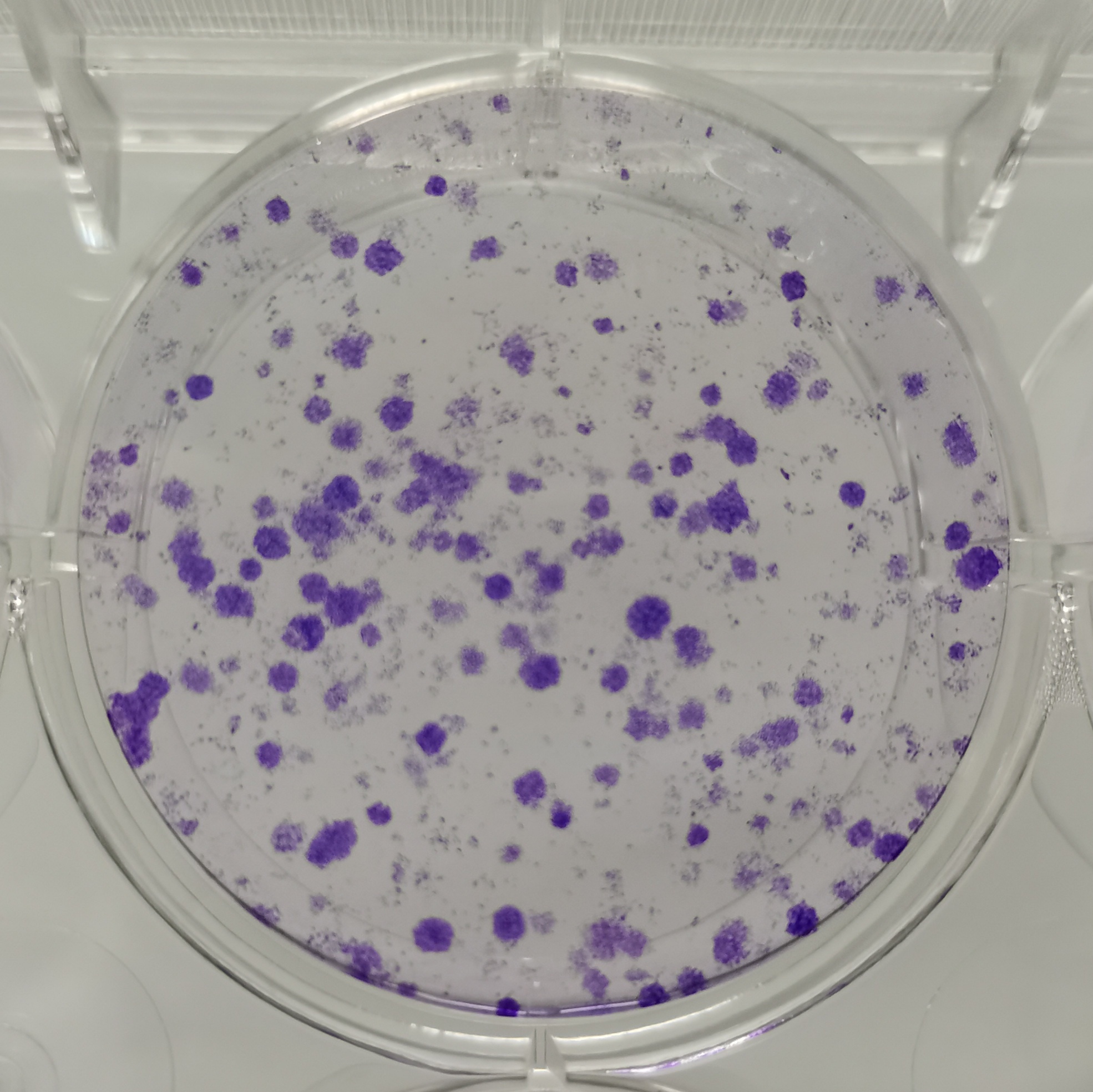

Supplement: Supplementary file 1 [file DataSheet3.ZIP › Clone formation assay/BT-549/6 (2)-1.jpg]

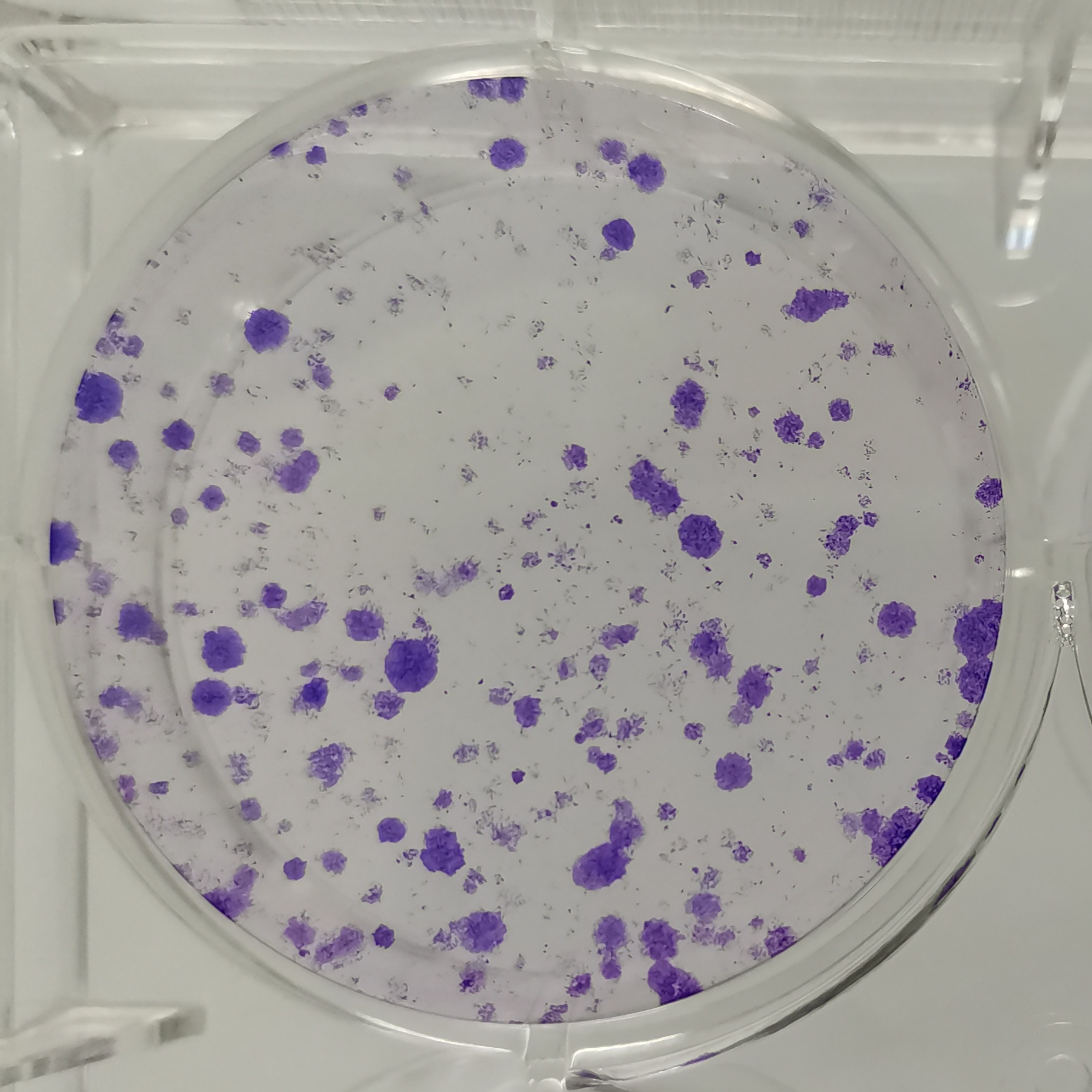

Supplement: Supplementary file 1 [file DataSheet3.ZIP › Clone formation assay/BT-549/6 (3).jpg]

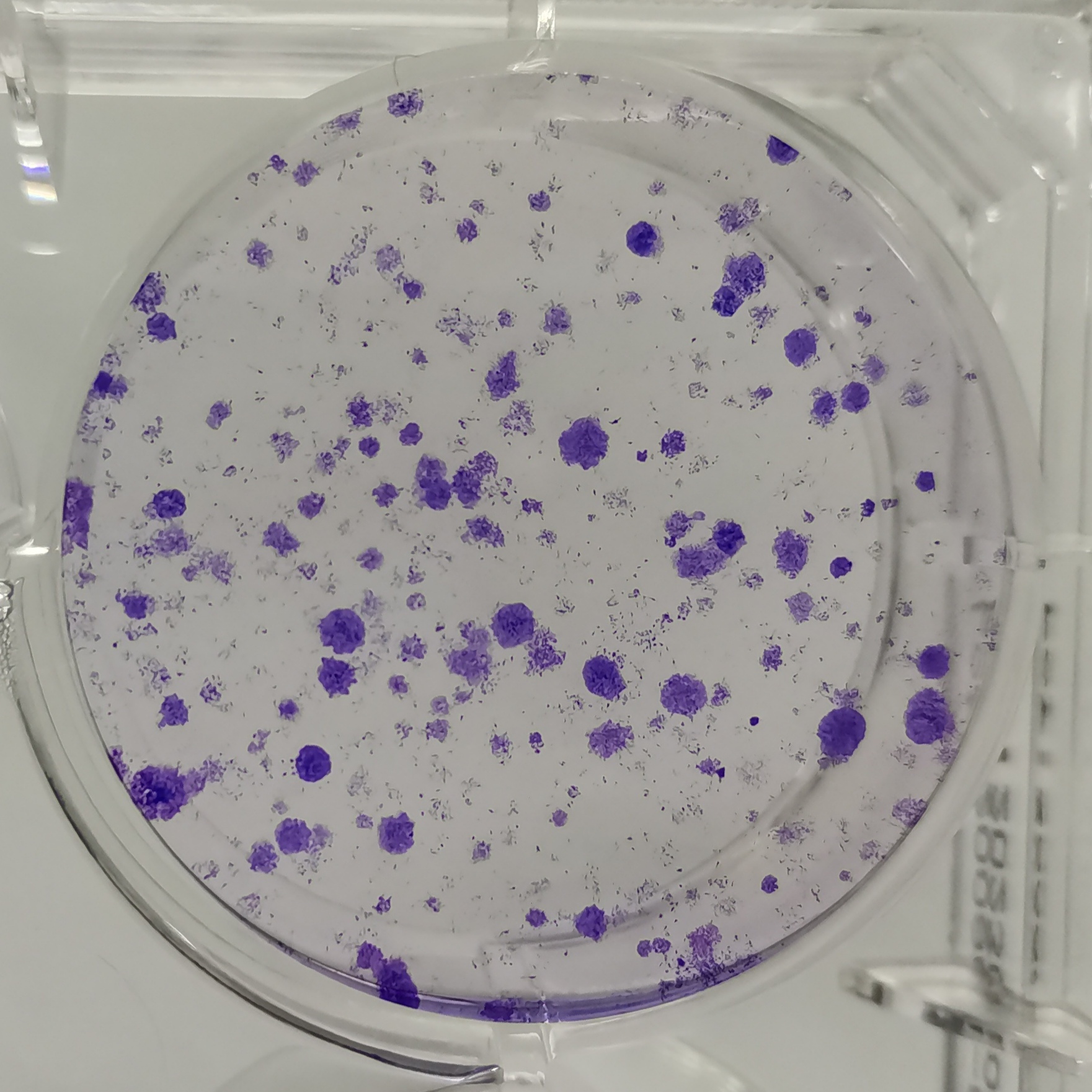

Supplement: Supplementary file 1 [file DataSheet3.ZIP › Clone formation assay/BT-549/7 (1).jpg]

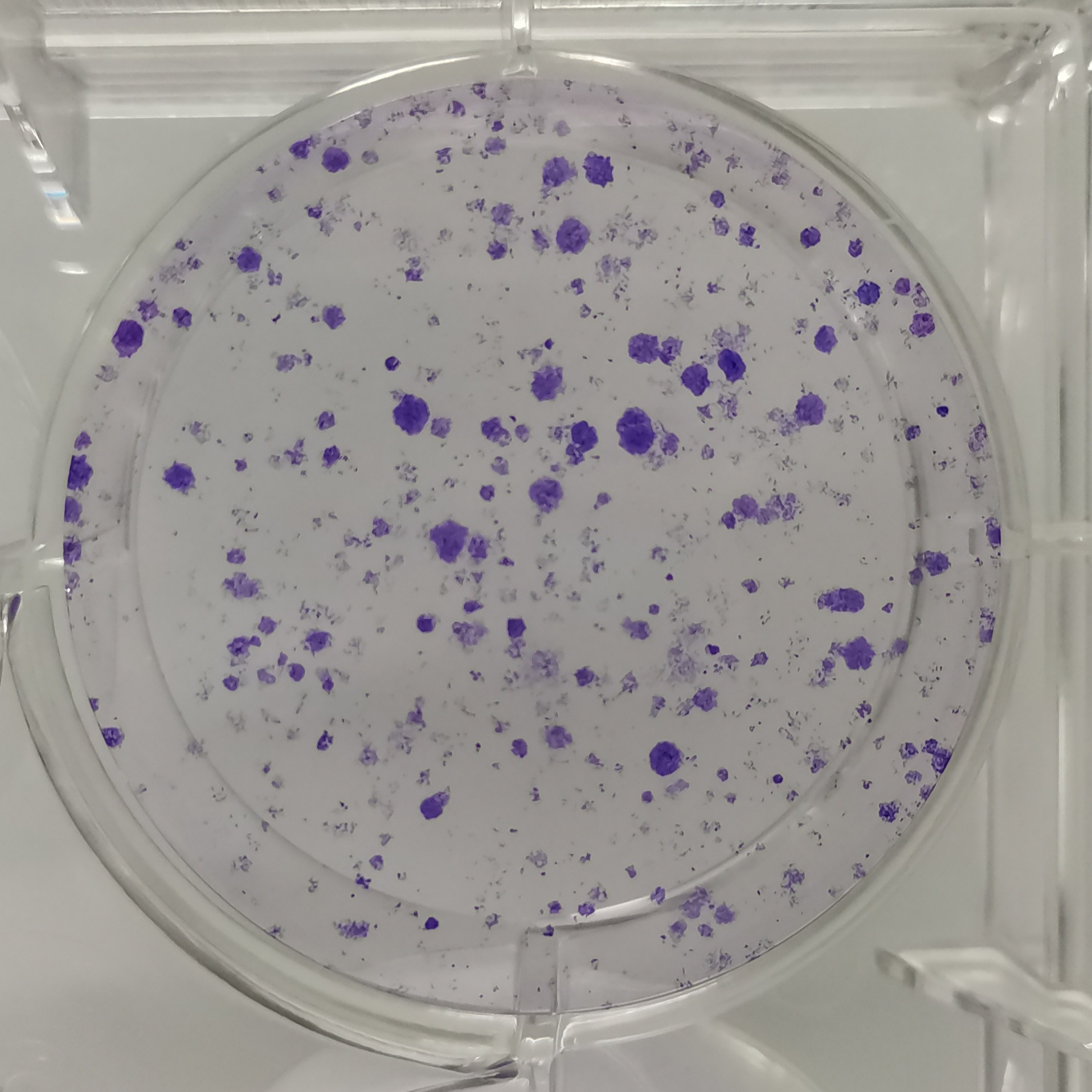

Supplement: Supplementary file 1 [file DataSheet3.ZIP › Clone formation assay/BT-549/7 (2)-1.jpg]

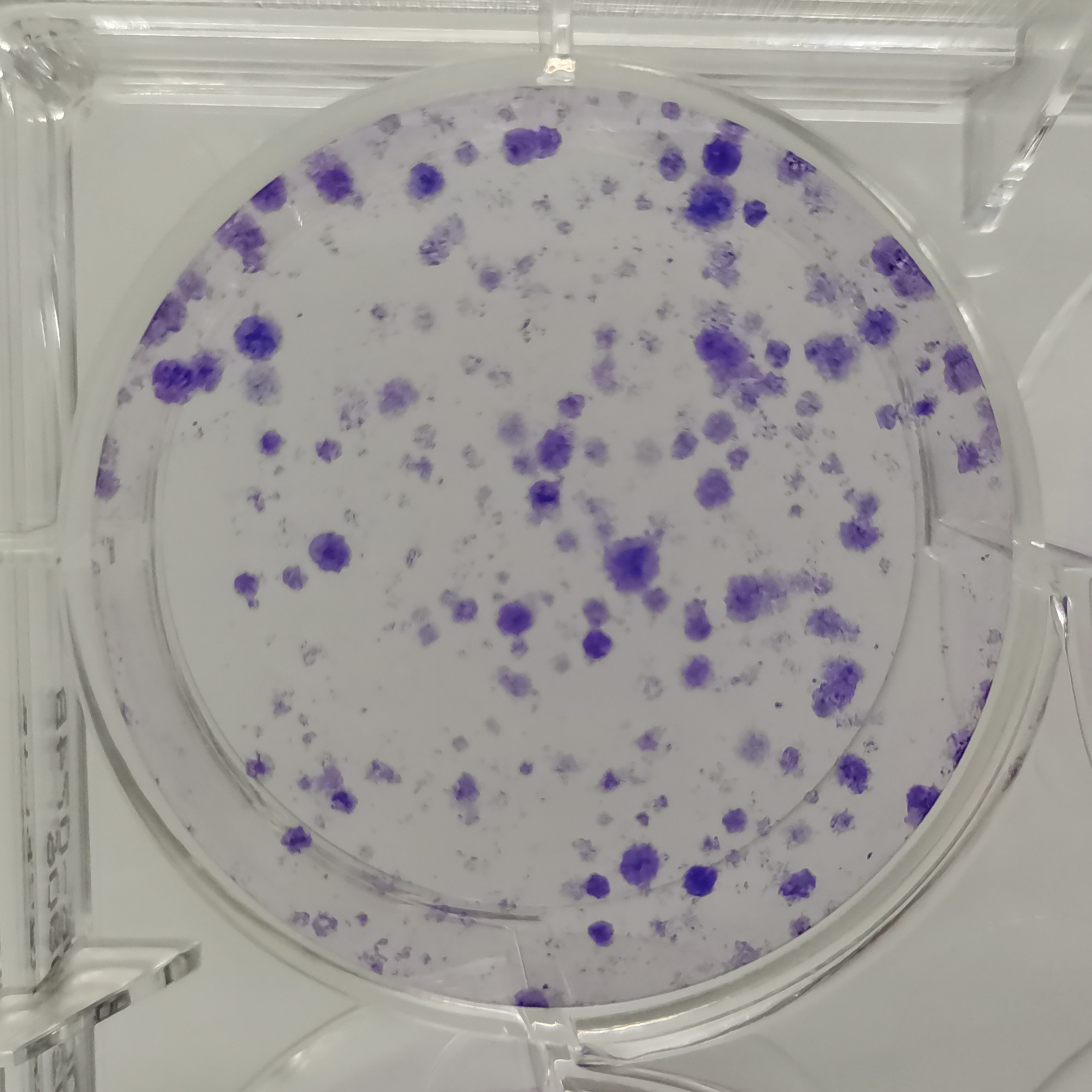

Supplement: Supplementary file 1 [file DataSheet3.ZIP › Clone formation assay/BT-549/7 (3).jpg]

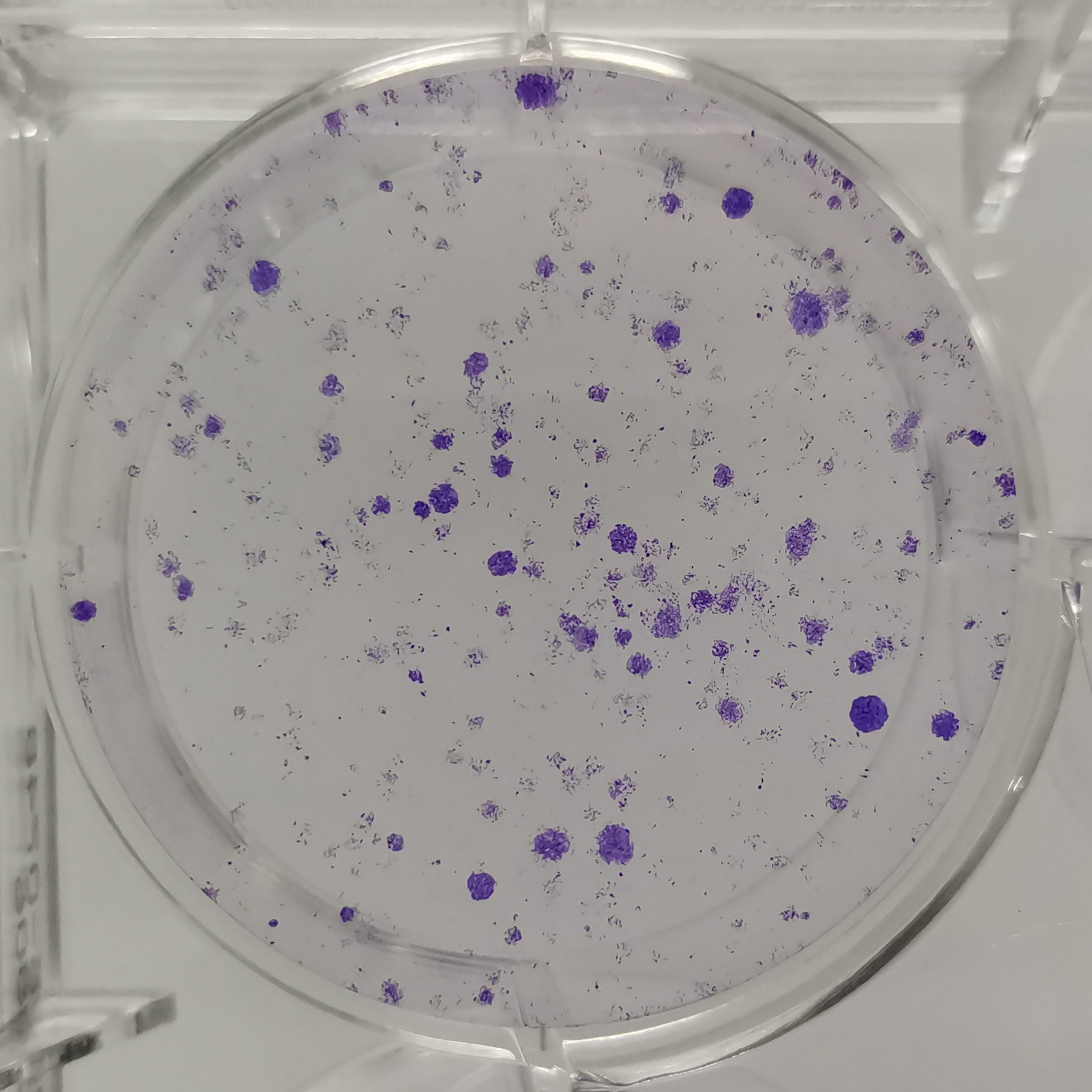

Supplement: Supplementary file 1 [file DataSheet3.ZIP › Clone formation assay/BT-549/8 (1)-1.jpg]

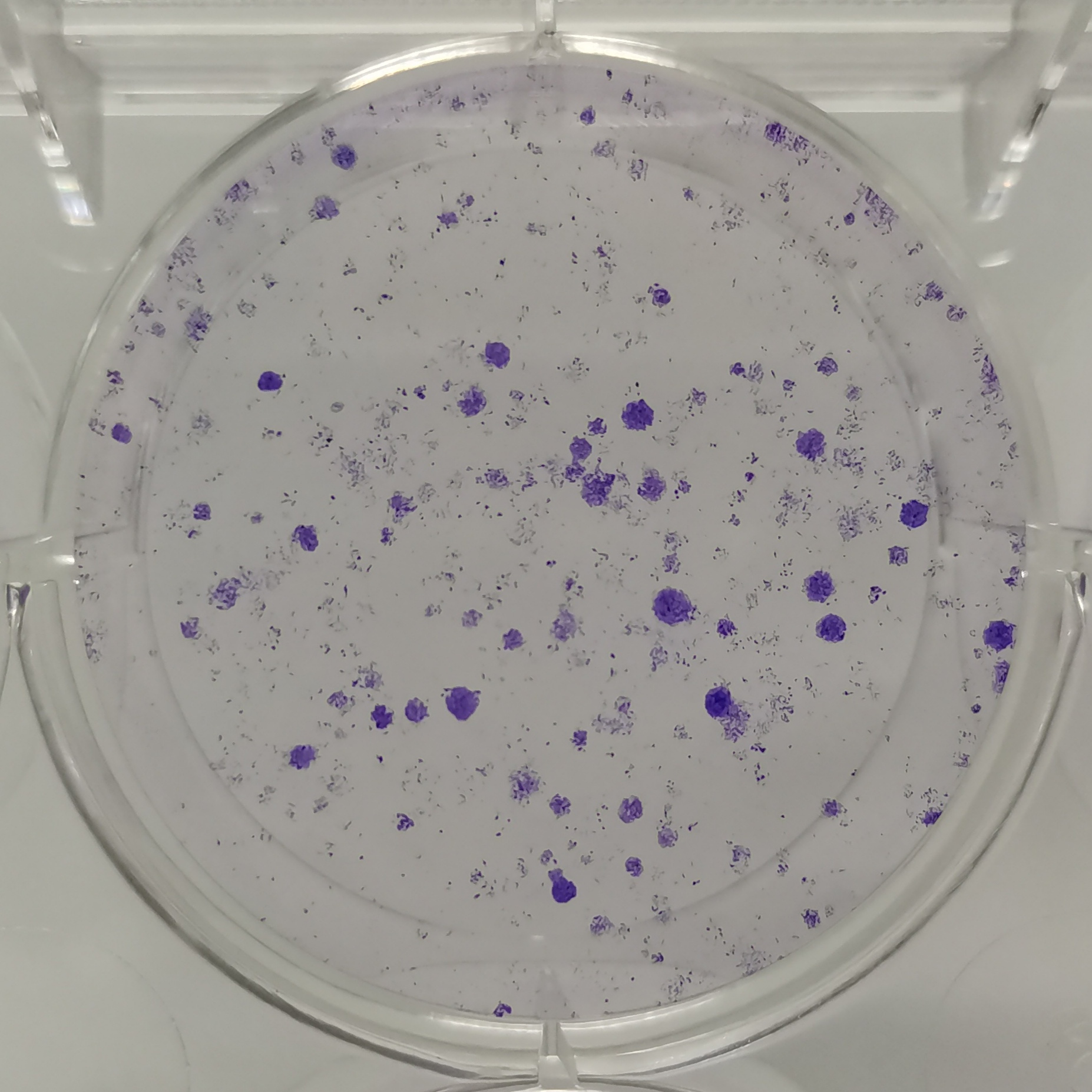

Supplement: Supplementary file 1 [file DataSheet3.ZIP › Clone formation assay/BT-549/8 (2).jpg]

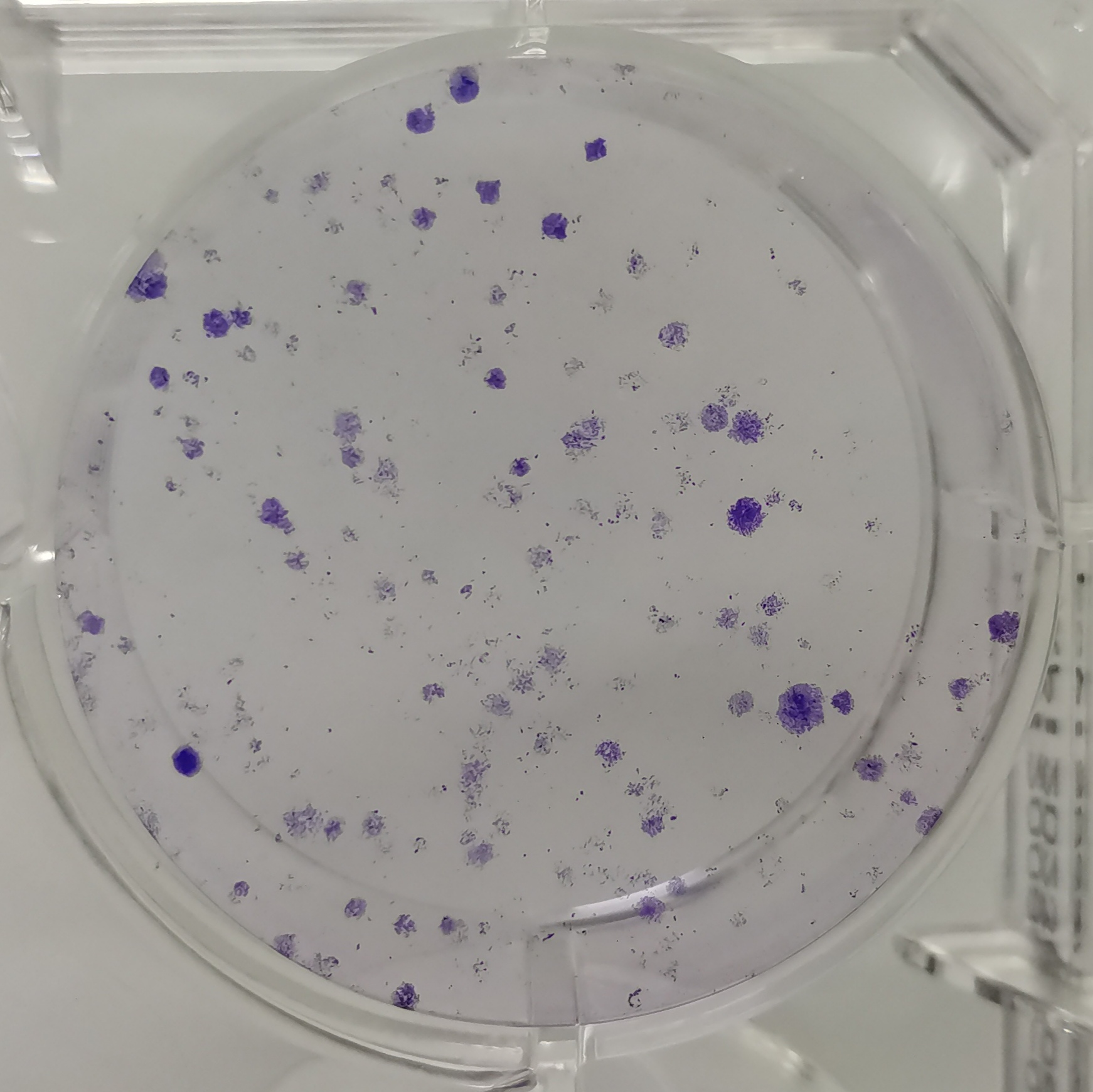

Supplement: Supplementary file 1 [file DataSheet3.ZIP › Clone formation assay/BT-549/8 (3).jpg]

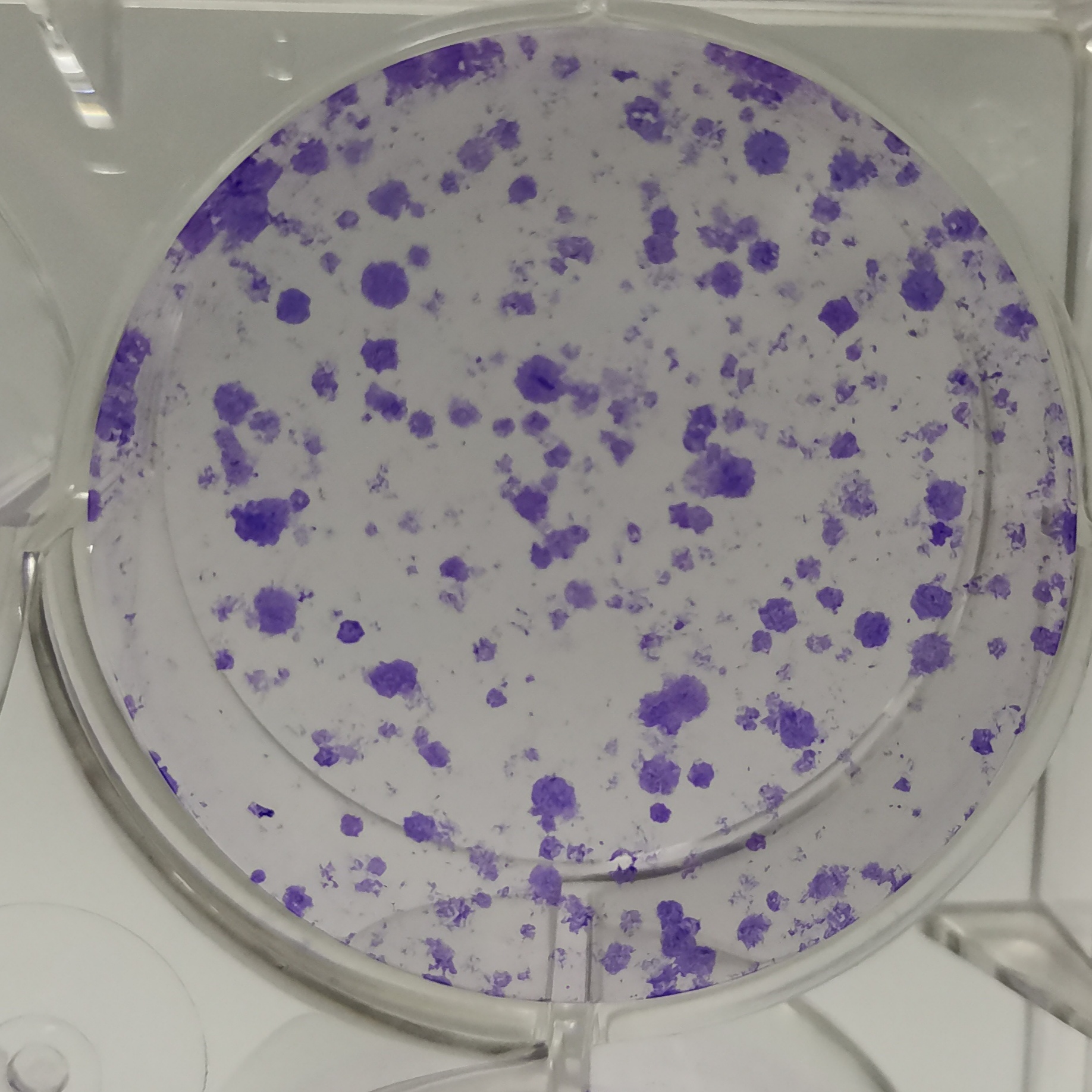

Supplement: Supplementary file 1 [file DataSheet3.ZIP › Clone formation assay/MDA-MB-231/1 (1)-1.jpg]

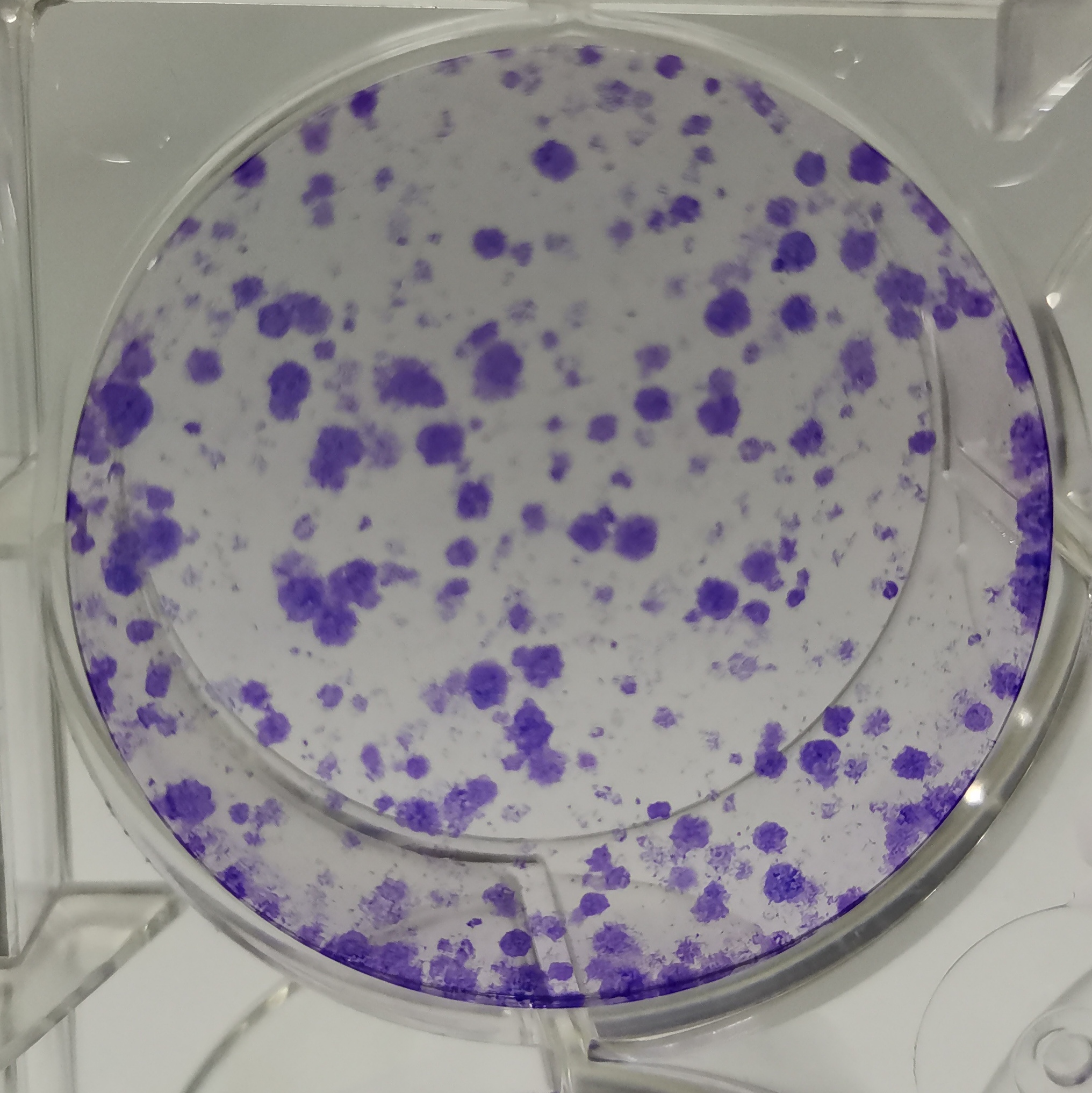

Supplement: Supplementary file 1 [file DataSheet3.ZIP › Clone formation assay/MDA-MB-231/1 (2).jpg]

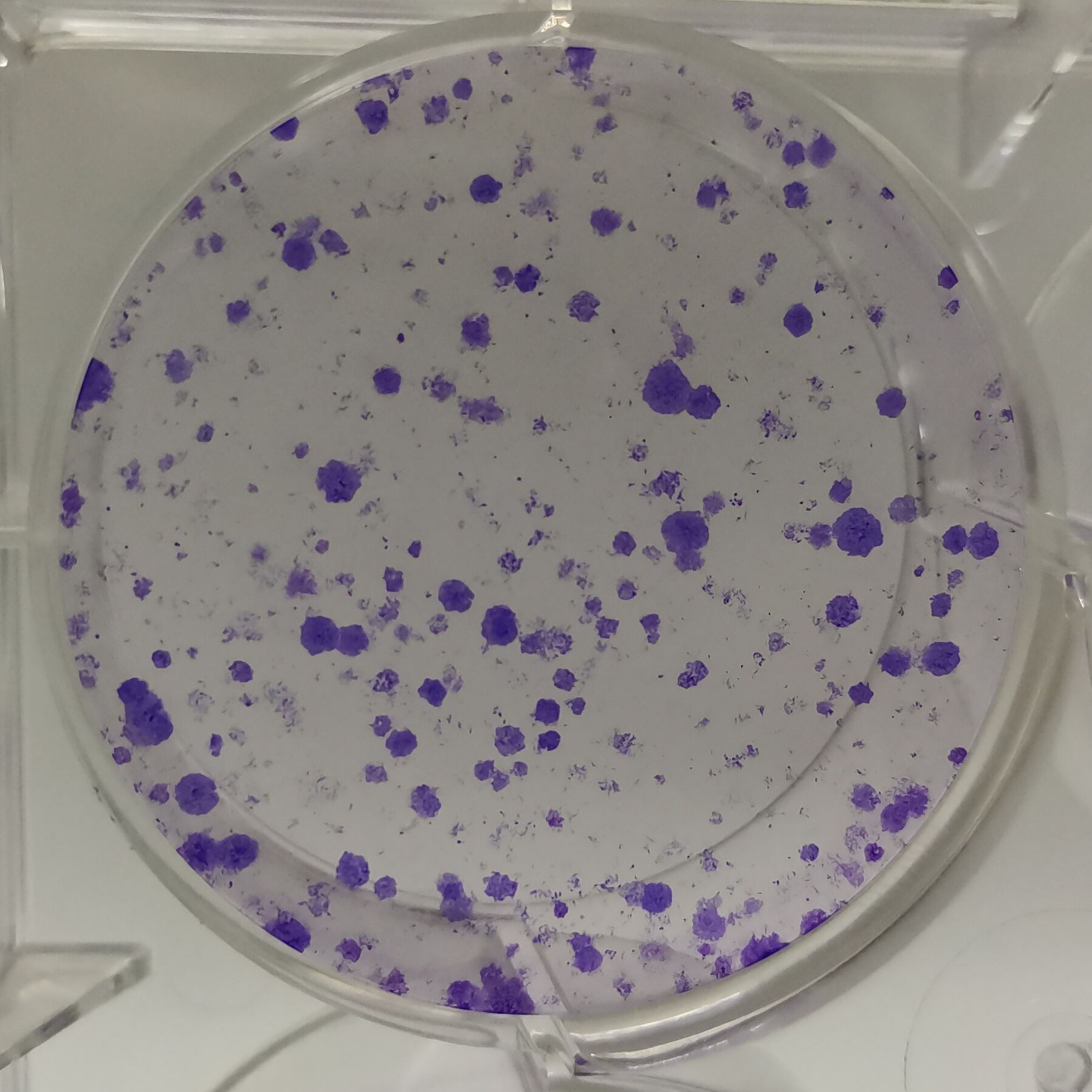

Supplement: Supplementary file 1 [file DataSheet3.ZIP › Clone formation assay/MDA-MB-231/1 (3).jpg]

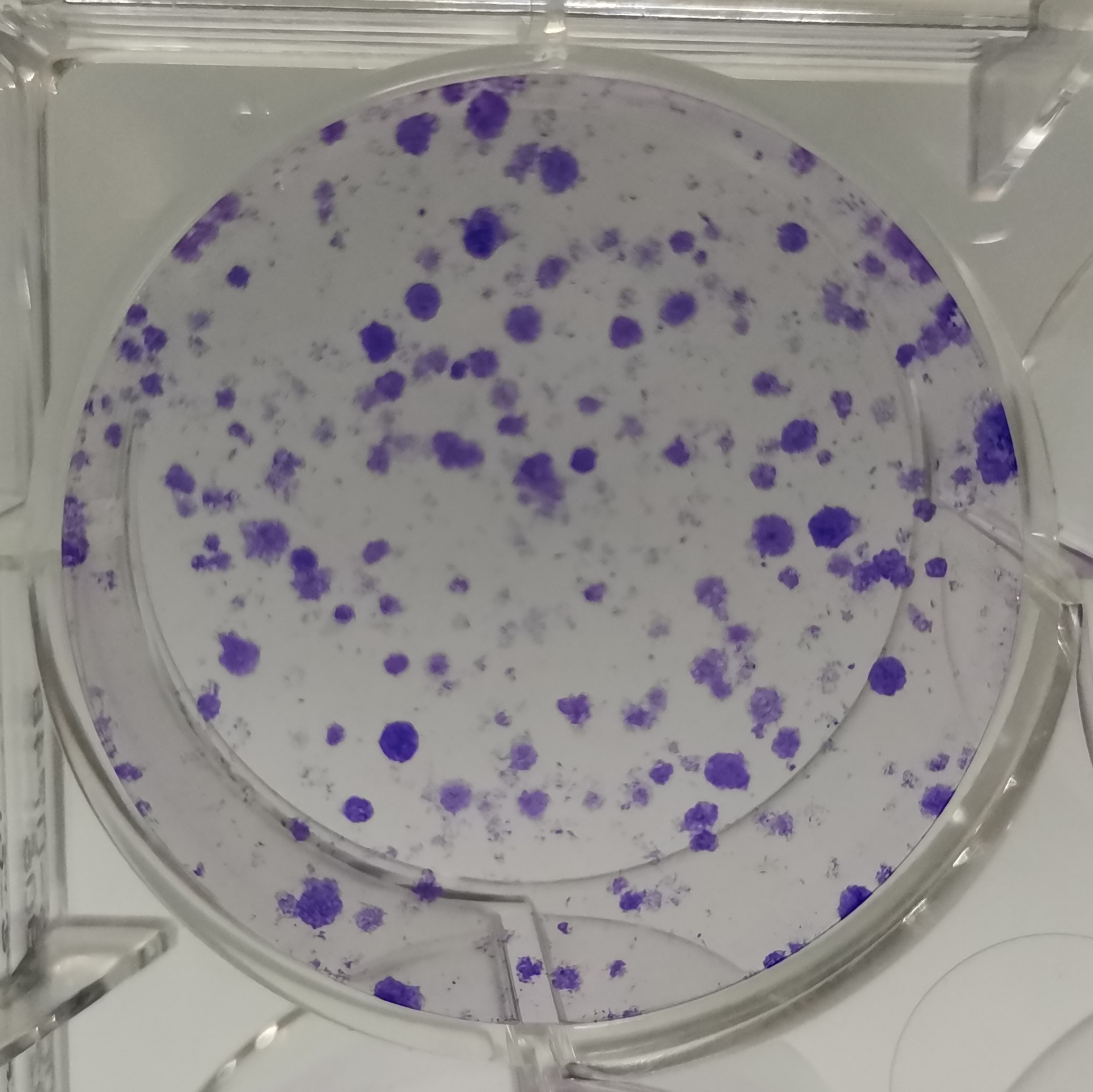

Supplement: Supplementary file 1 [file DataSheet3.ZIP › Clone formation assay/MDA-MB-231/2 (1).jpg]

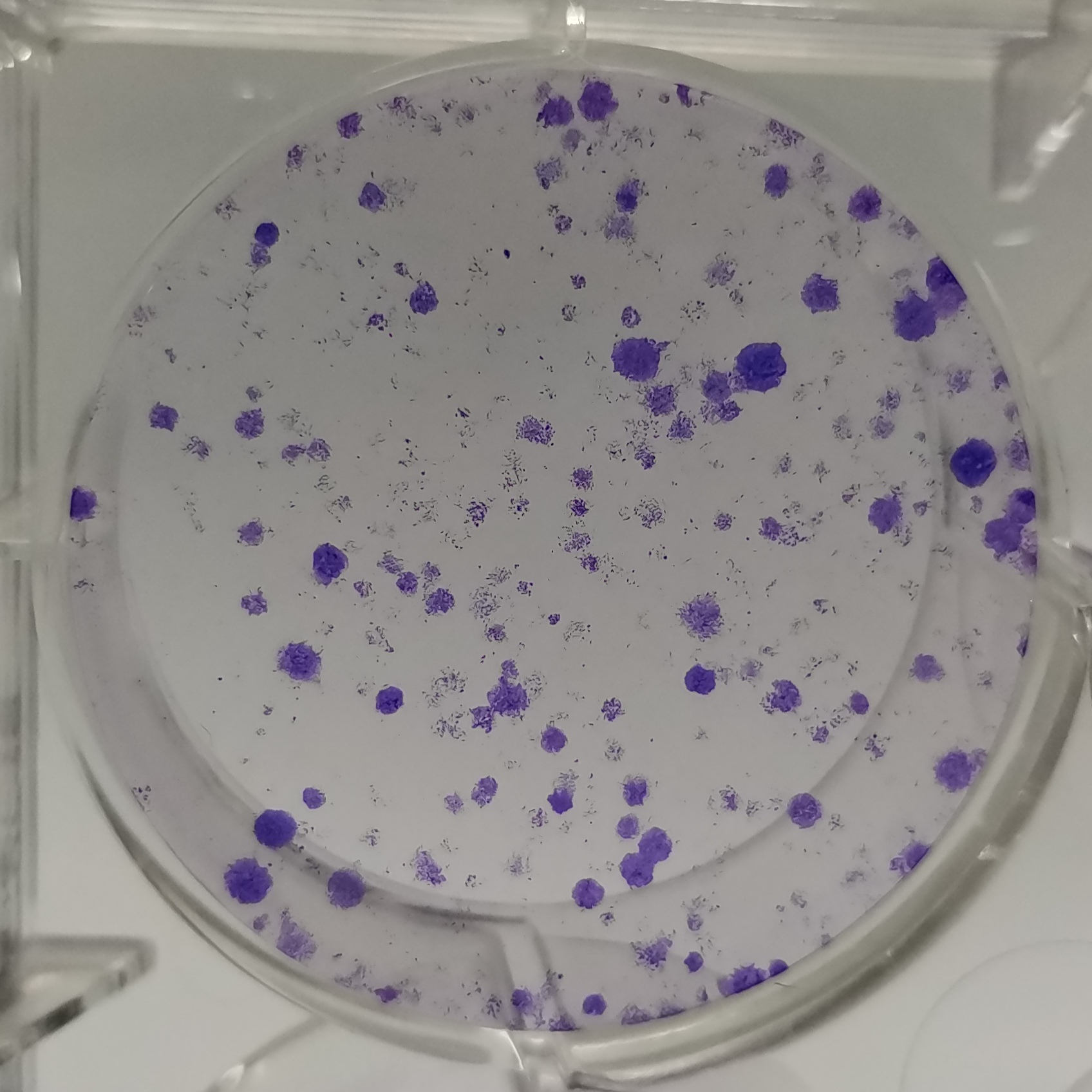

Supplement: Supplementary file 1 [file DataSheet3.ZIP › Clone formation assay/MDA-MB-231/2 (2)-1.jpg]

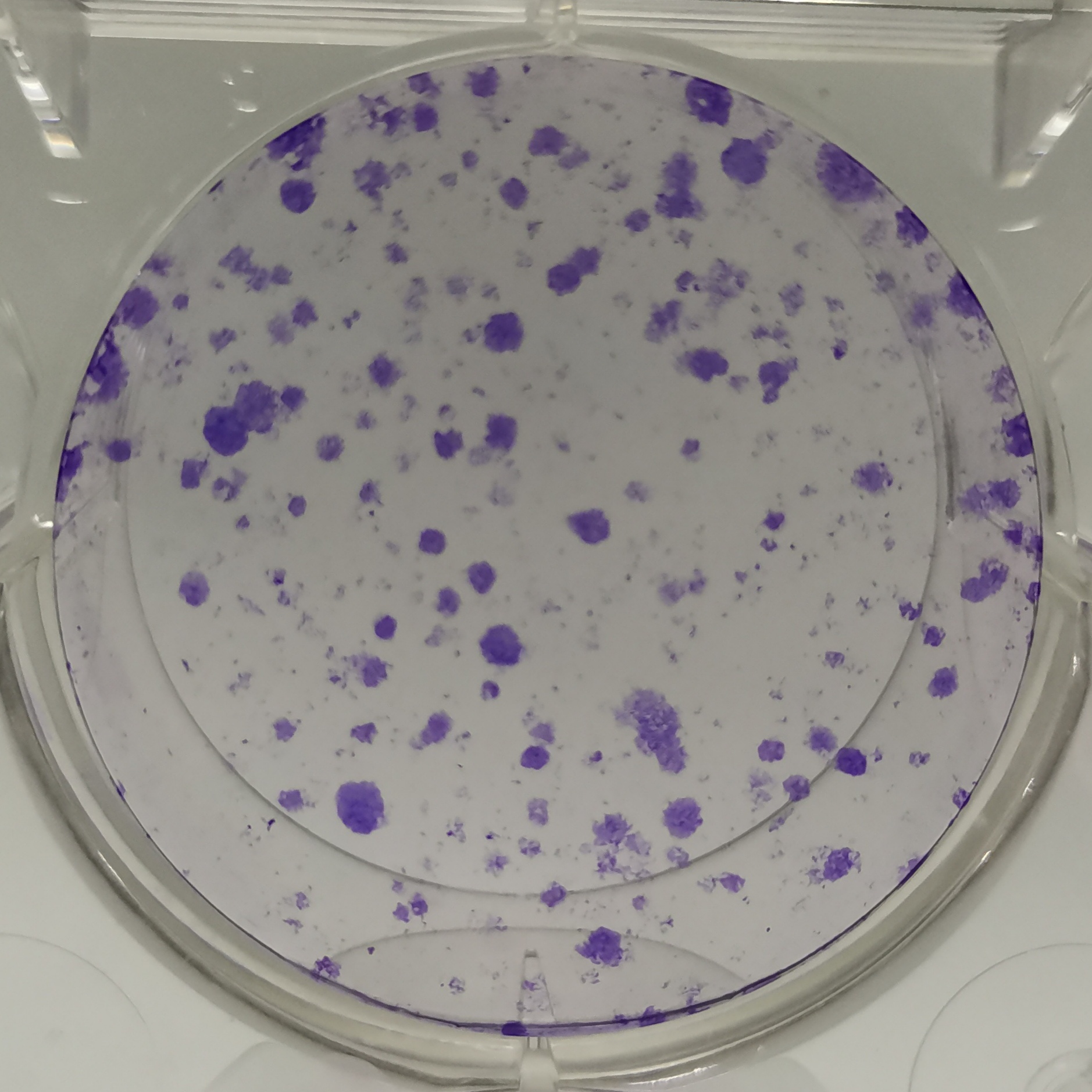

Supplement: Supplementary file 1 [file DataSheet3.ZIP › Clone formation assay/MDA-MB-231/2 (3).jpg]

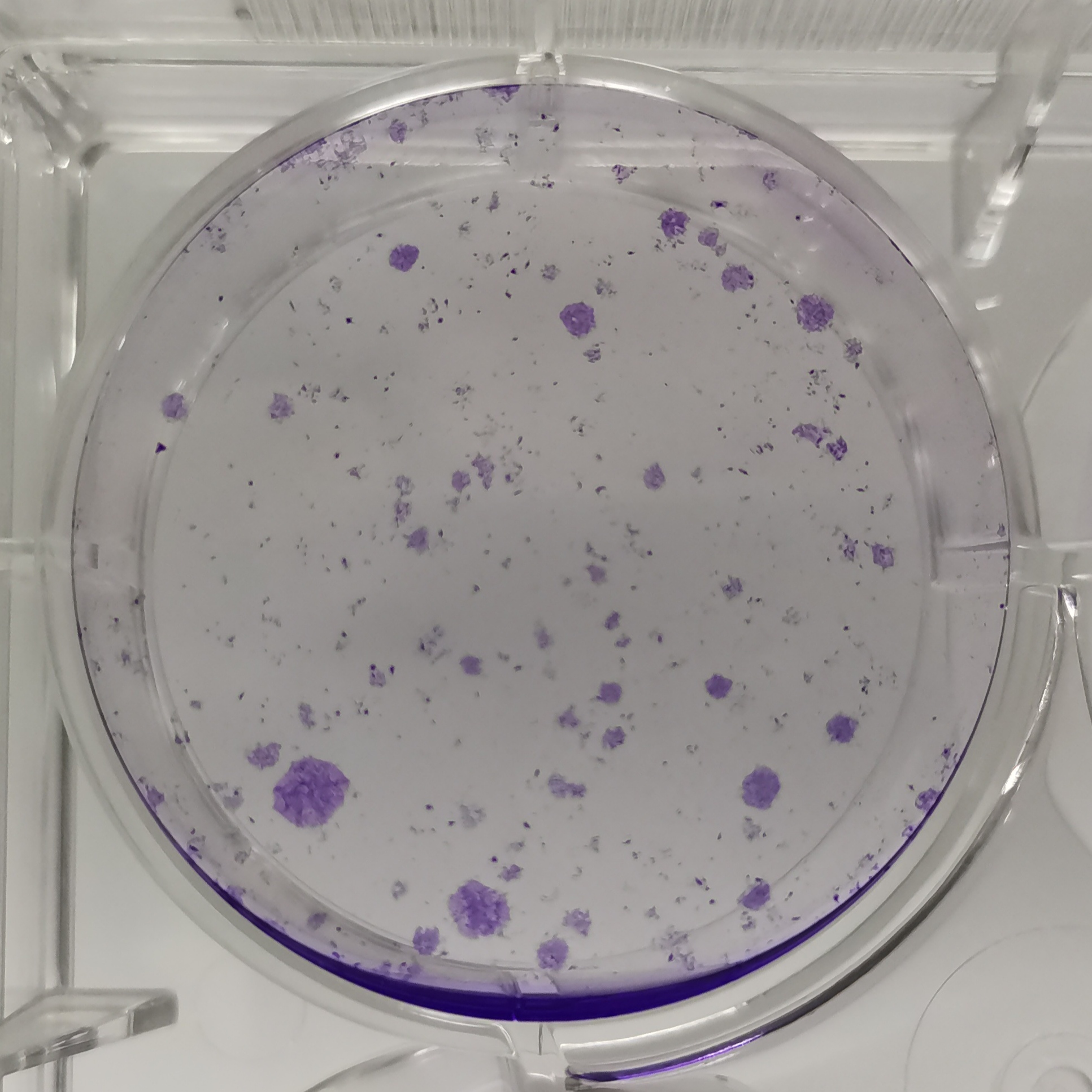

Supplement: Supplementary file 1 [file DataSheet3.ZIP › Clone formation assay/MDA-MB-231/3 (1)-1.jpg]

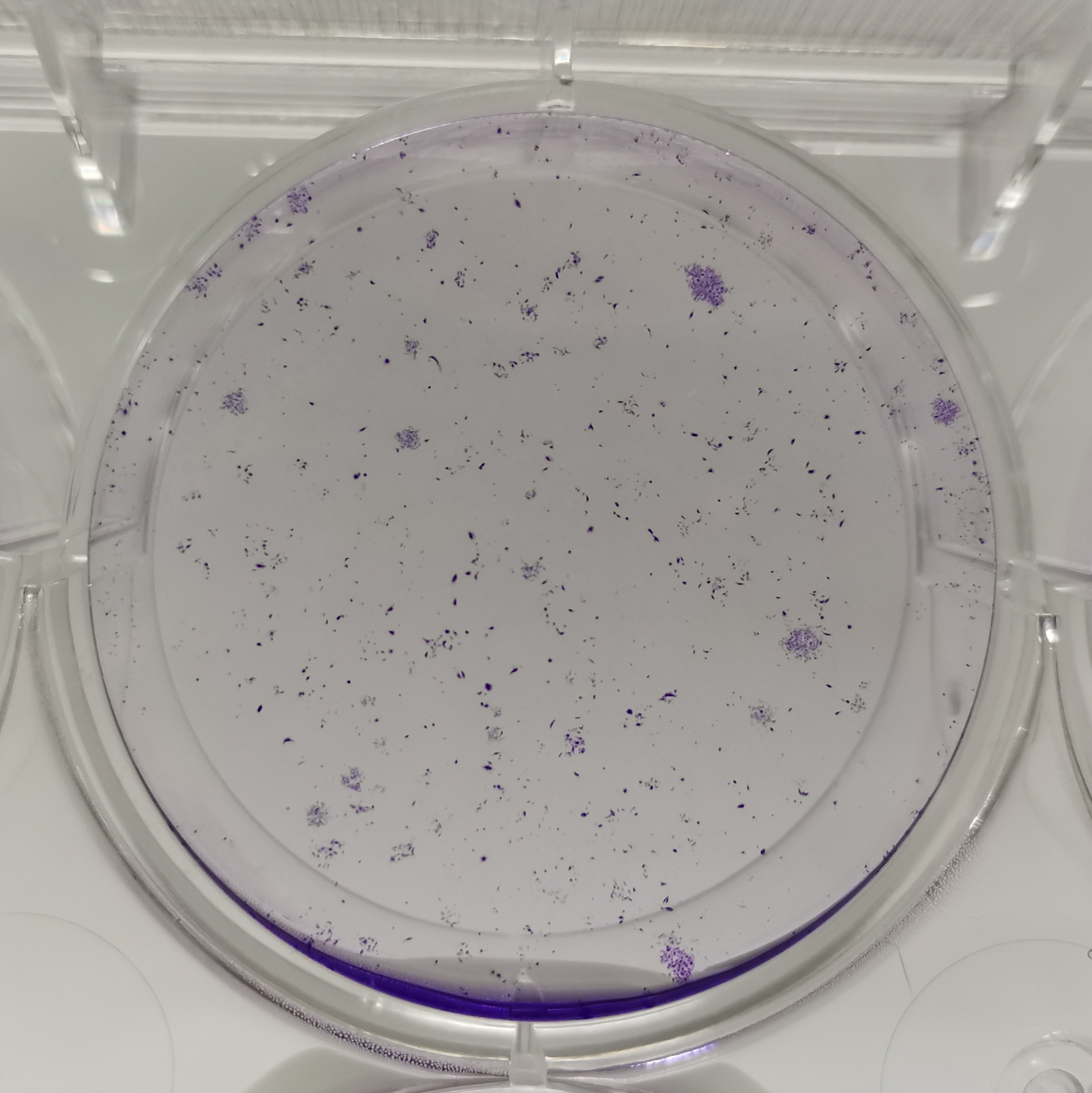

Supplement: Supplementary file 1 [file DataSheet3.ZIP › Clone formation assay/MDA-MB-231/3 (2).jpg]

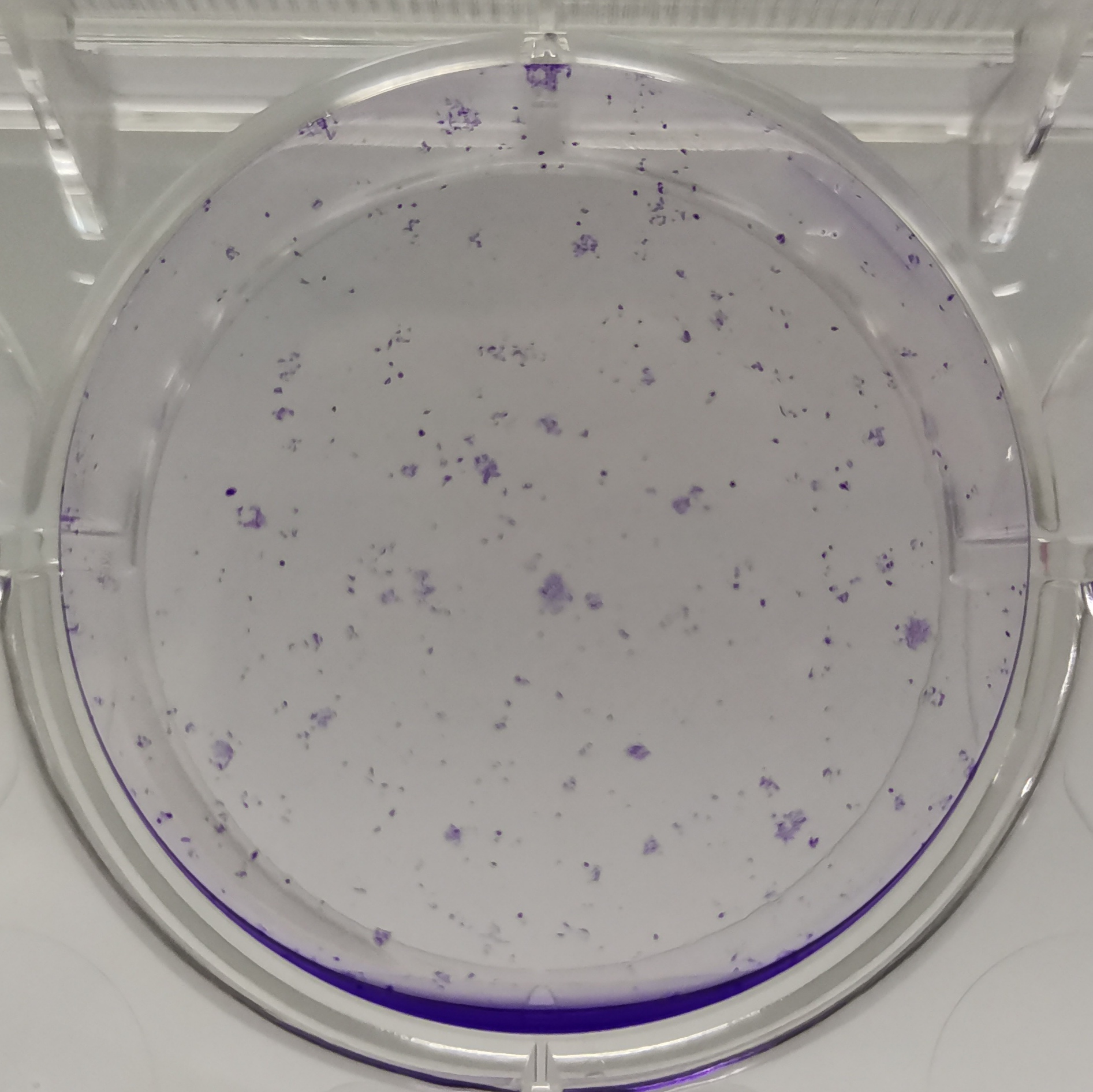

Supplement: Supplementary file 1 [file DataSheet3.ZIP › Clone formation assay/MDA-MB-231/3 (3).jpg]

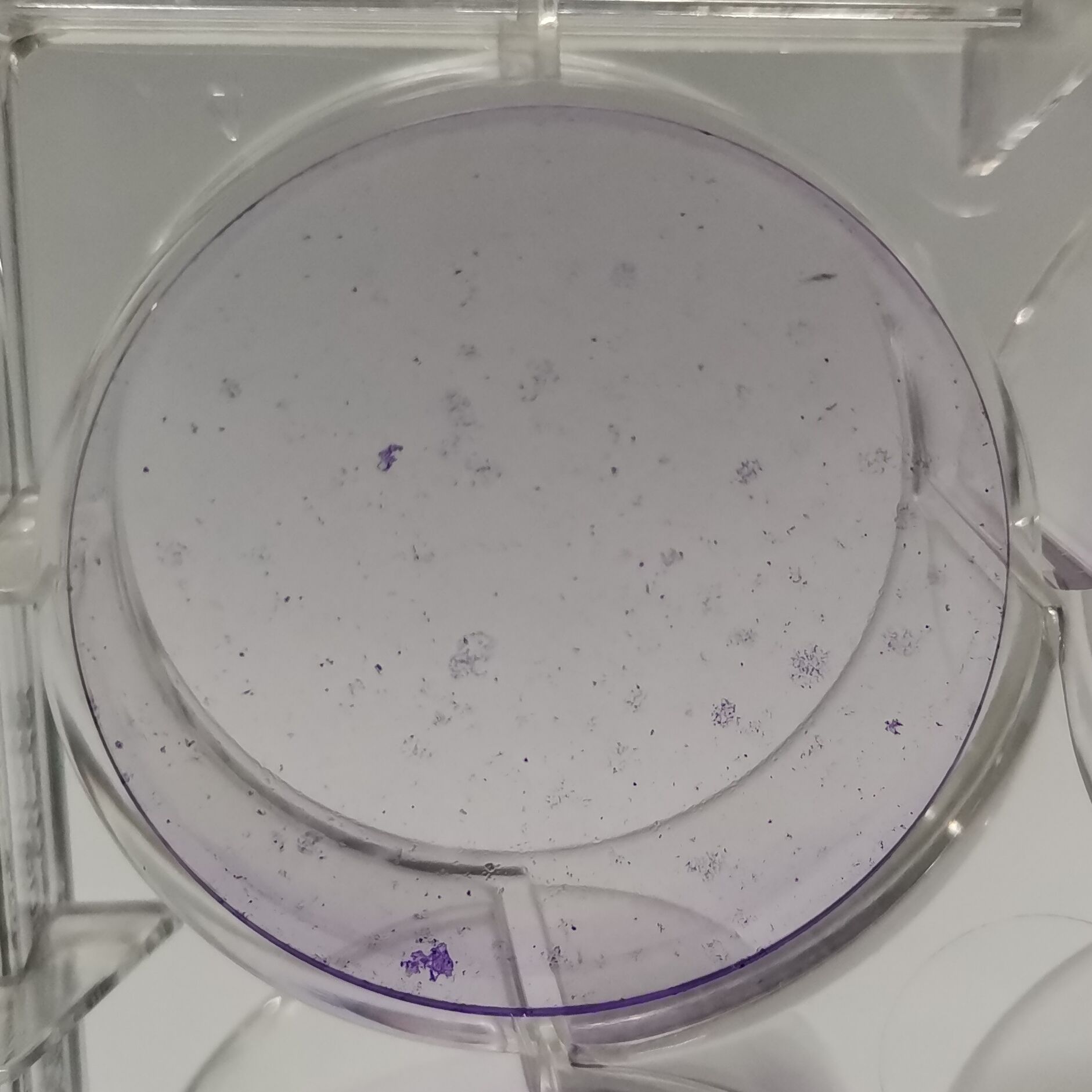

Supplement: Supplementary file 1 [file DataSheet3.ZIP › Clone formation assay/MDA-MB-231/4 (1).jpg]

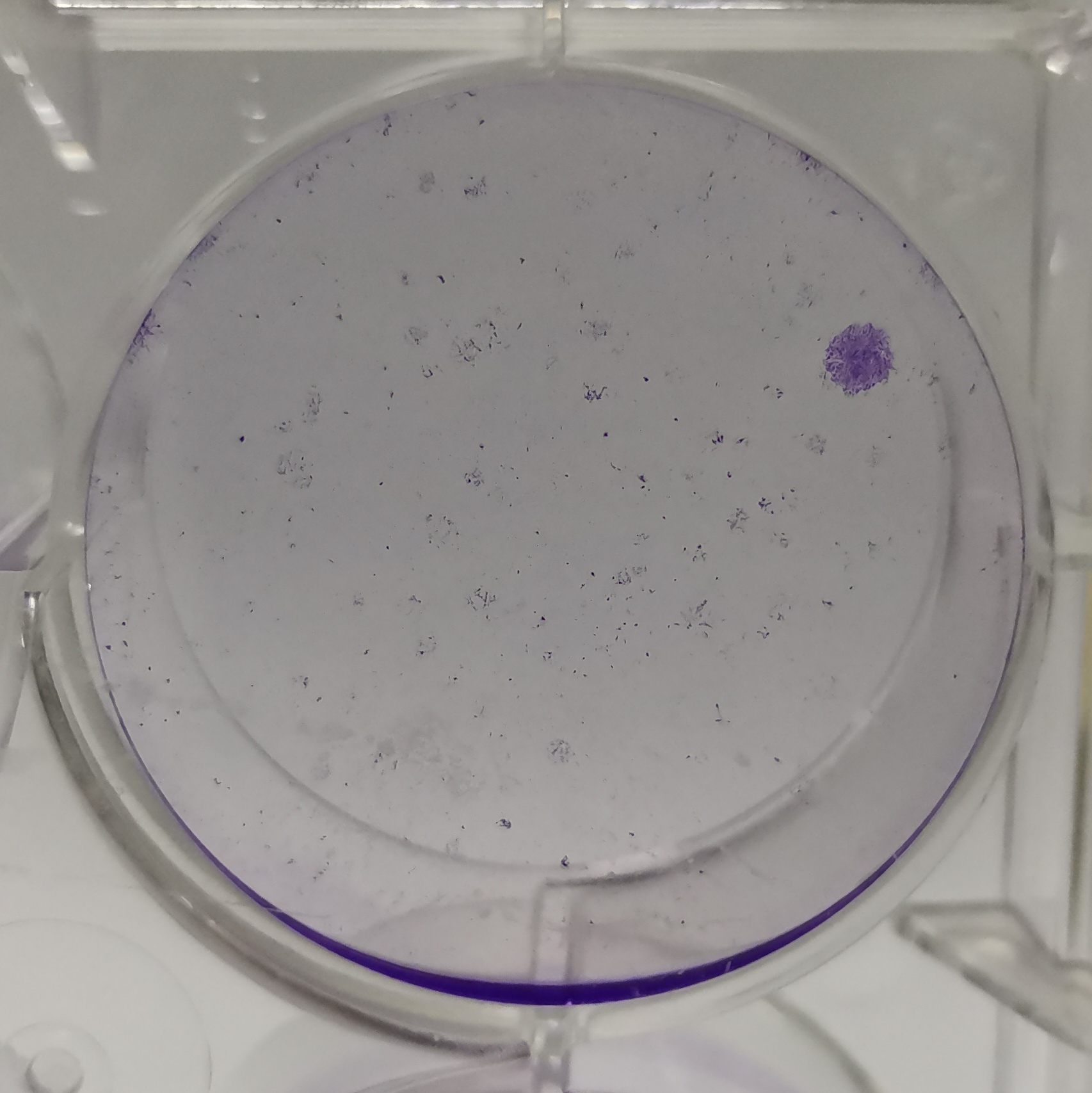

Supplement: Supplementary file 1 [file DataSheet3.ZIP › Clone formation assay/MDA-MB-231/4 (2).jpg]

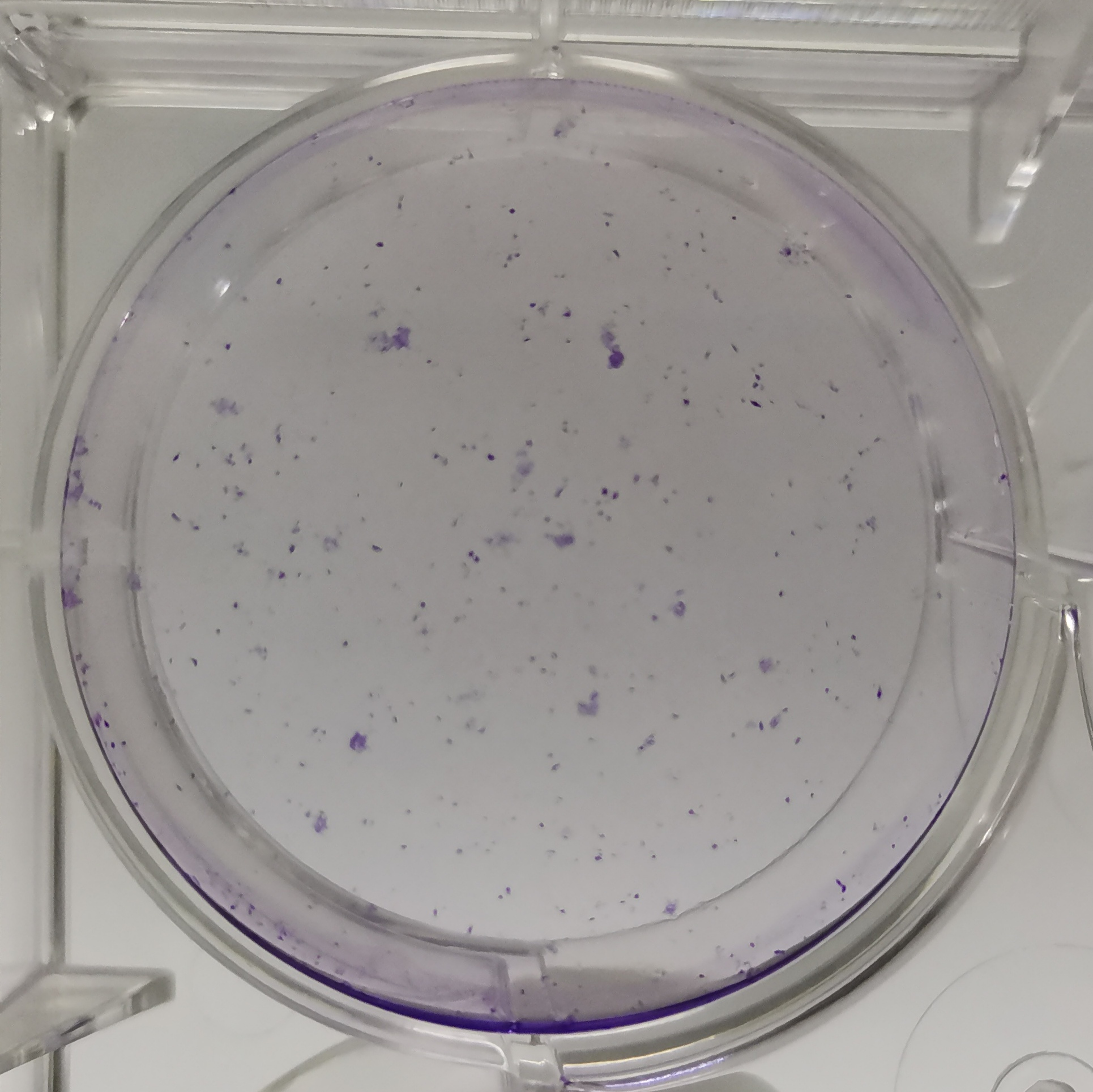

Supplement: Supplementary file 1 [file DataSheet3.ZIP › Clone formation assay/MDA-MB-231/4 (3)-1.jpg]

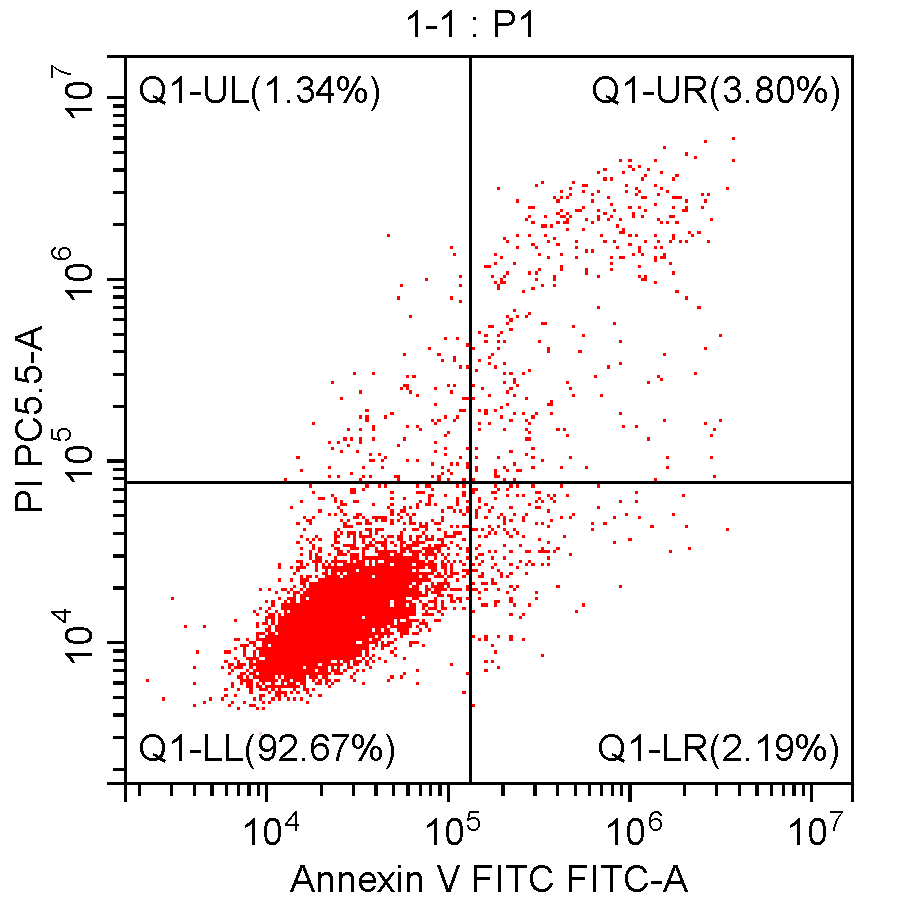

Supplement: Supplementary file 3 [file DataSheet4.ZIP › BT-549/1-1_Plot1.bmp]

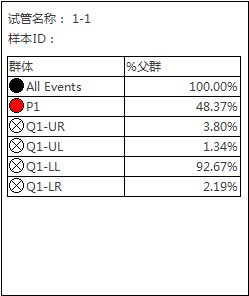

Supplement: Supplementary file 3 [file DataSheet4.ZIP › BT-549/1-1_Statistics1.bmp]

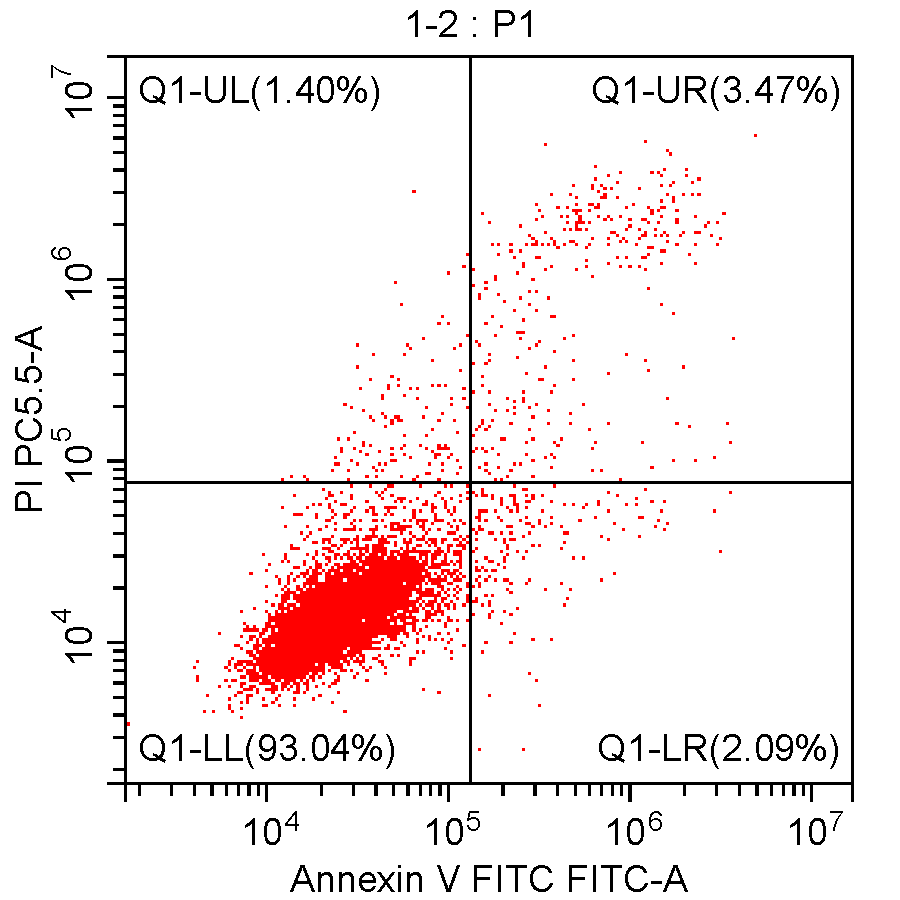

Supplement: Supplementary file 3 [file DataSheet4.ZIP › BT-549/1-2_Plot1.bmp]

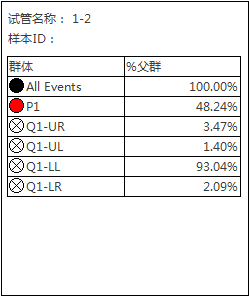

Supplement: Supplementary file 3 [file DataSheet4.ZIP › BT-549/1-2_Statistics1.bmp]

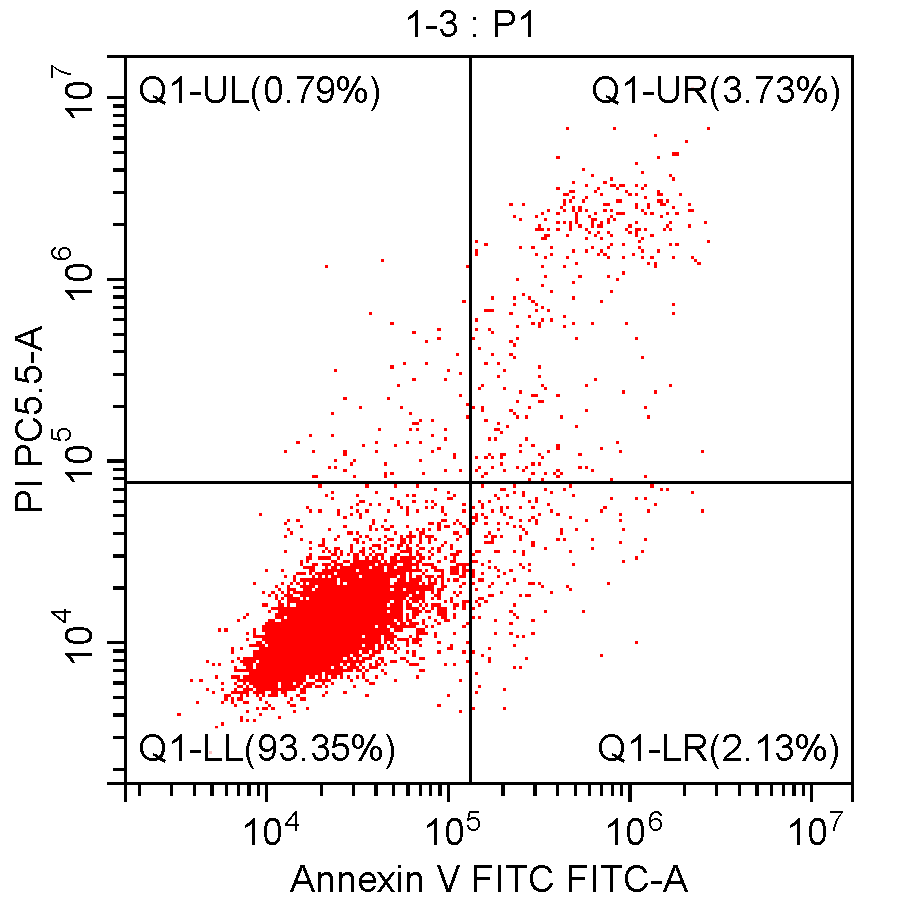

Supplement: Supplementary file 3 [file DataSheet4.ZIP › BT-549/1-3_Plot1.bmp]

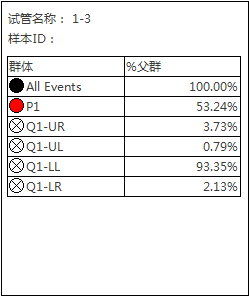

Supplement: Supplementary file 3 [file DataSheet4.ZIP › BT-549/1-3_Statistics1.bmp]

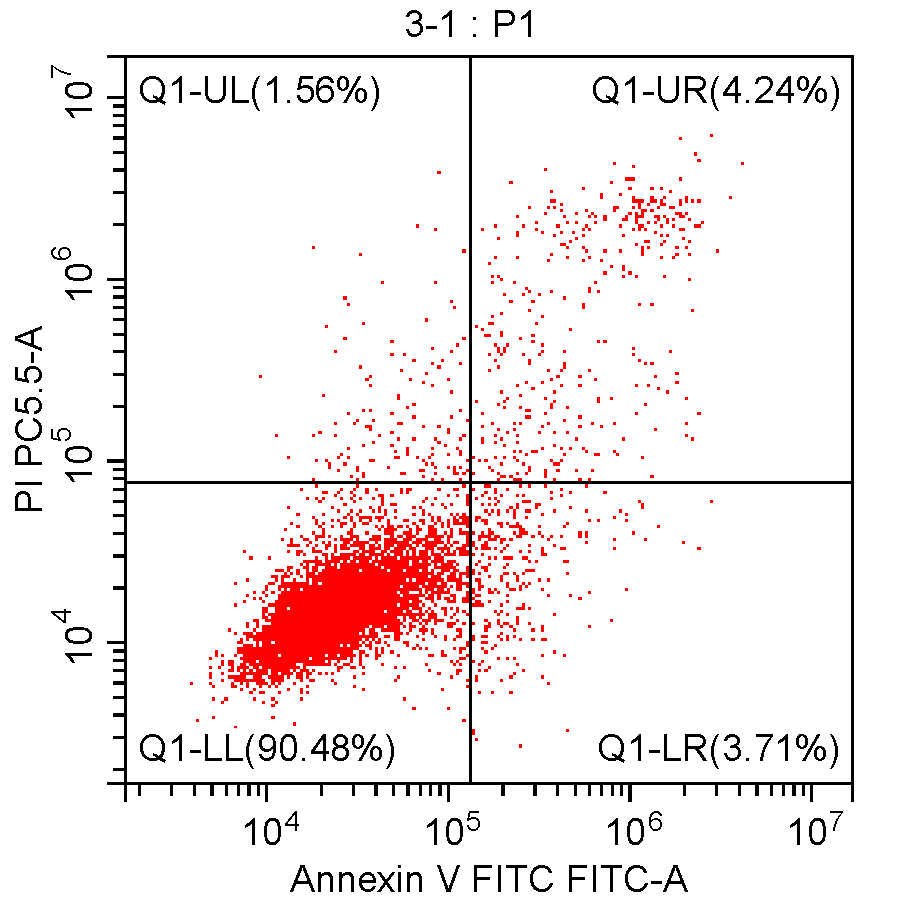

Supplement: Supplementary file 3 [file DataSheet4.ZIP › BT-549/2-1_Plot1.bmp]

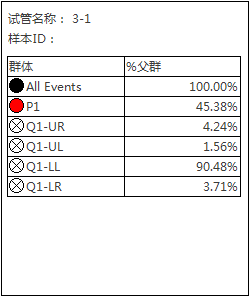

Supplement: Supplementary file 3 [file DataSheet4.ZIP › BT-549/2-1_Statistics1.bmp]

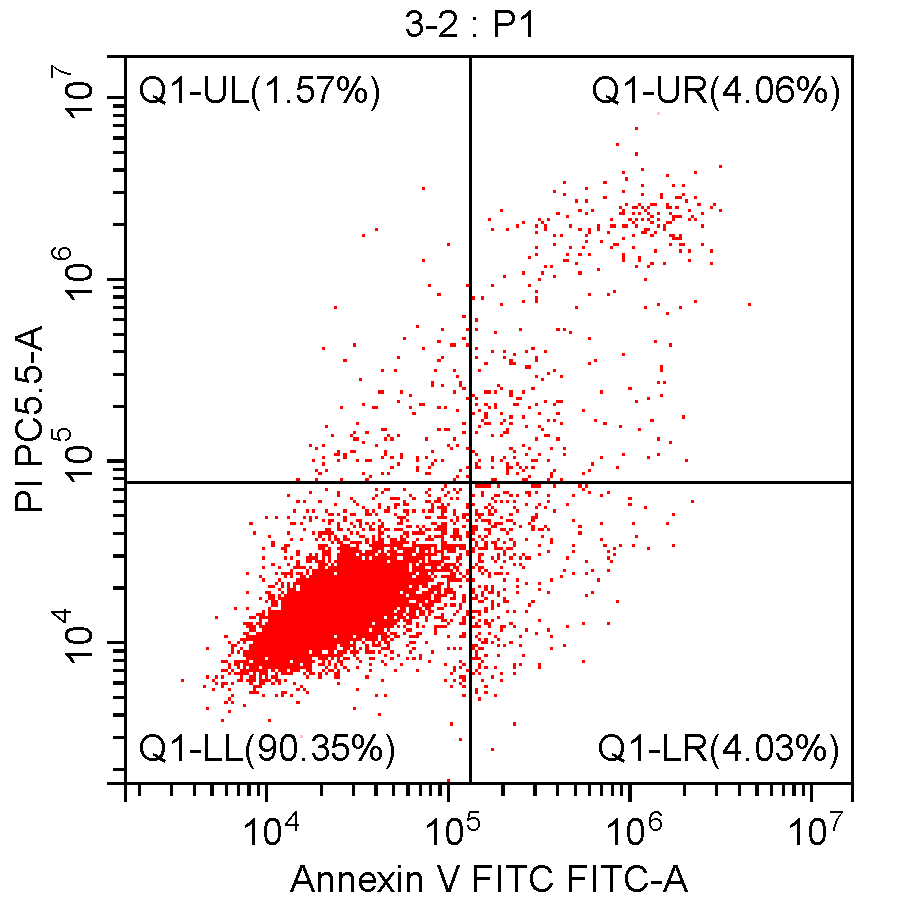

Supplement: Supplementary file 3 [file DataSheet4.ZIP › BT-549/2-2_Plot1.bmp]

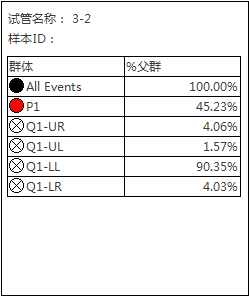

Supplement: Supplementary file 3 [file DataSheet4.ZIP › BT-549/2-2_Statistics1.bmp]

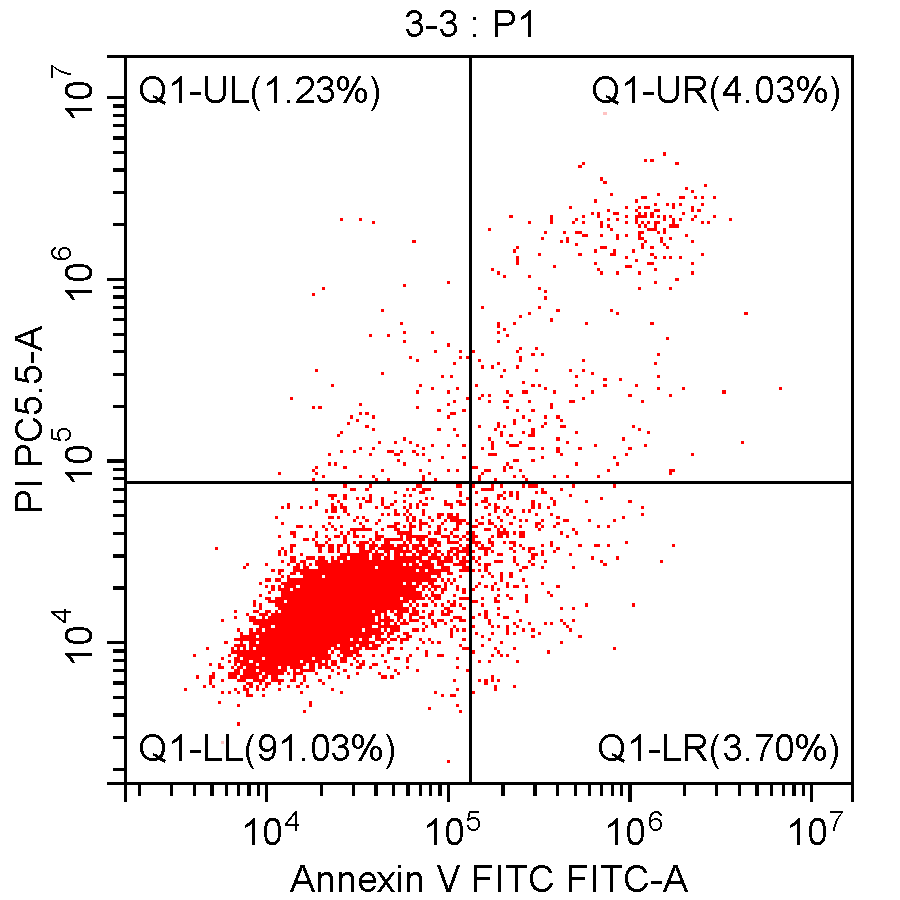

Supplement: Supplementary file 3 [file DataSheet4.ZIP › BT-549/2-3_Plot1.bmp]

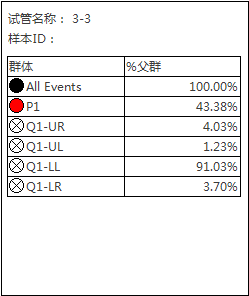

Supplement: Supplementary file 3 [file DataSheet4.ZIP › BT-549/2-3_Statistics1.bmp]

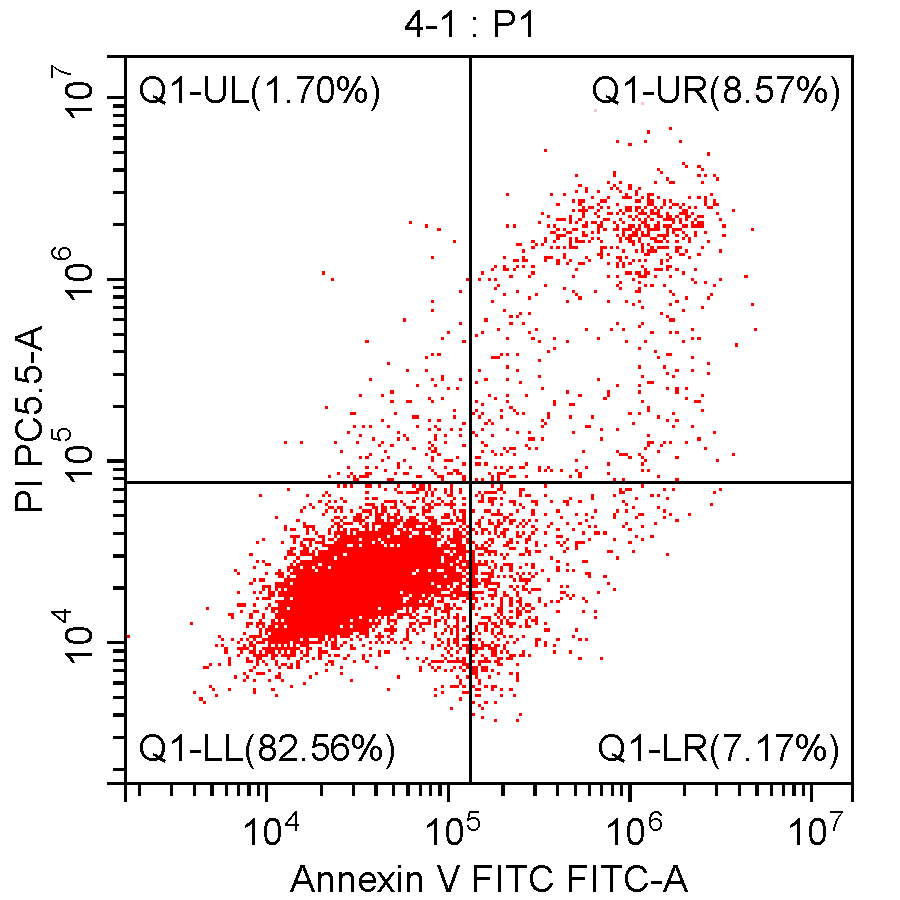

Supplement: Supplementary file 3 [file DataSheet4.ZIP › BT-549/3-1_Plot1.bmp]

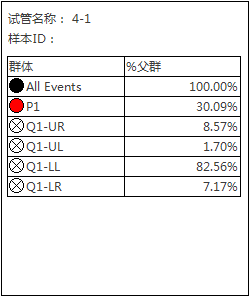

Supplement: Supplementary file 3 [file DataSheet4.ZIP › BT-549/3-1_Statistics1.bmp]

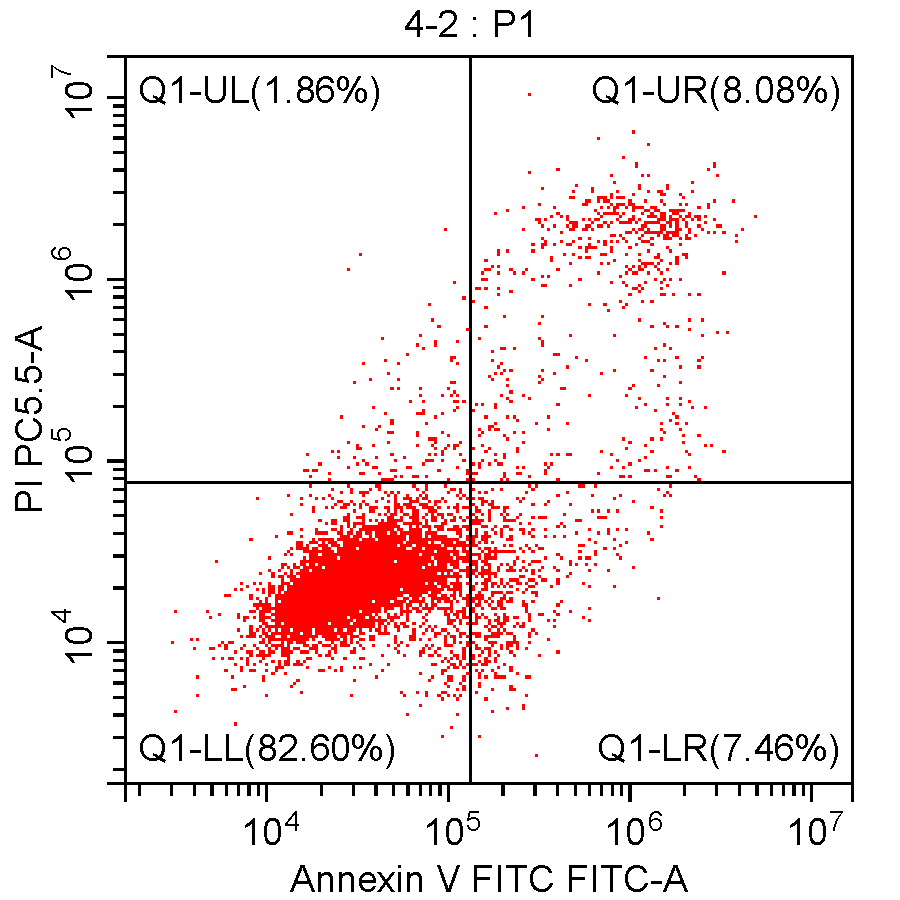

Supplement: Supplementary file 3 [file DataSheet4.ZIP › BT-549/3-2_Plot1.bmp]

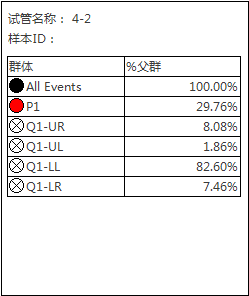

Supplement: Supplementary file 3 [file DataSheet4.ZIP › BT-549/3-2_Statistics1.bmp]

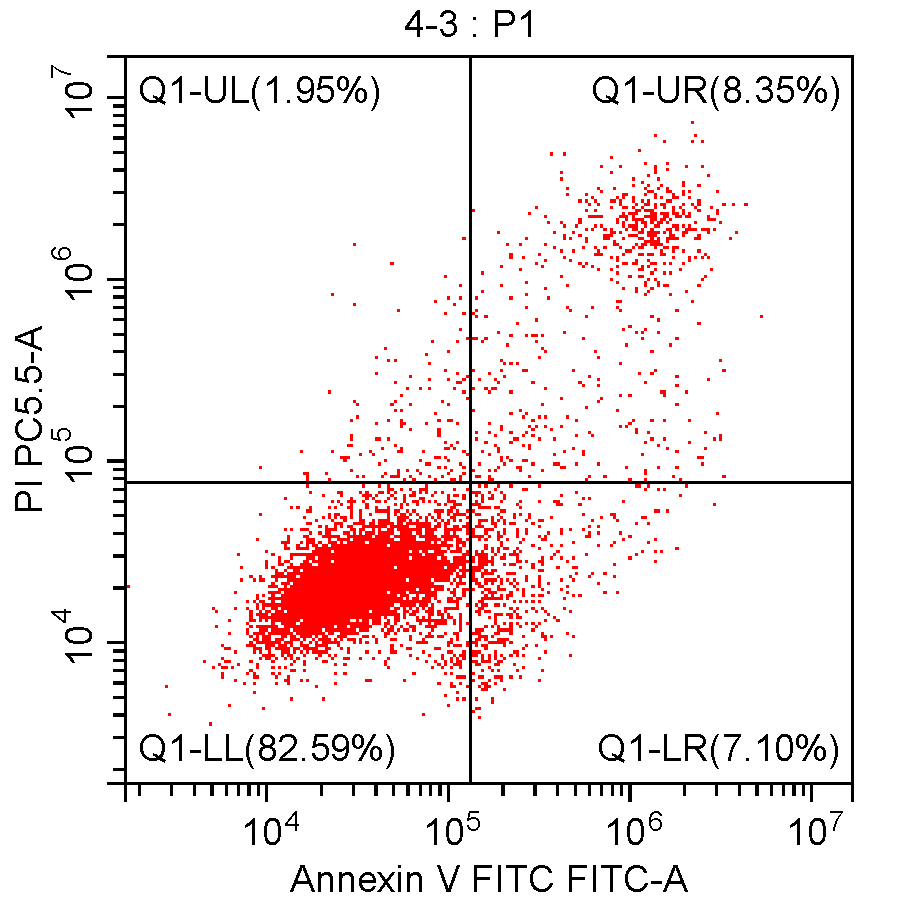

Supplement: Supplementary file 3 [file DataSheet4.ZIP › BT-549/3-3_Plot1.bmp]

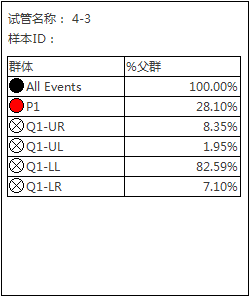

Supplement: Supplementary file 3 [file DataSheet4.ZIP › BT-549/3-3_Statistics1.bmp]

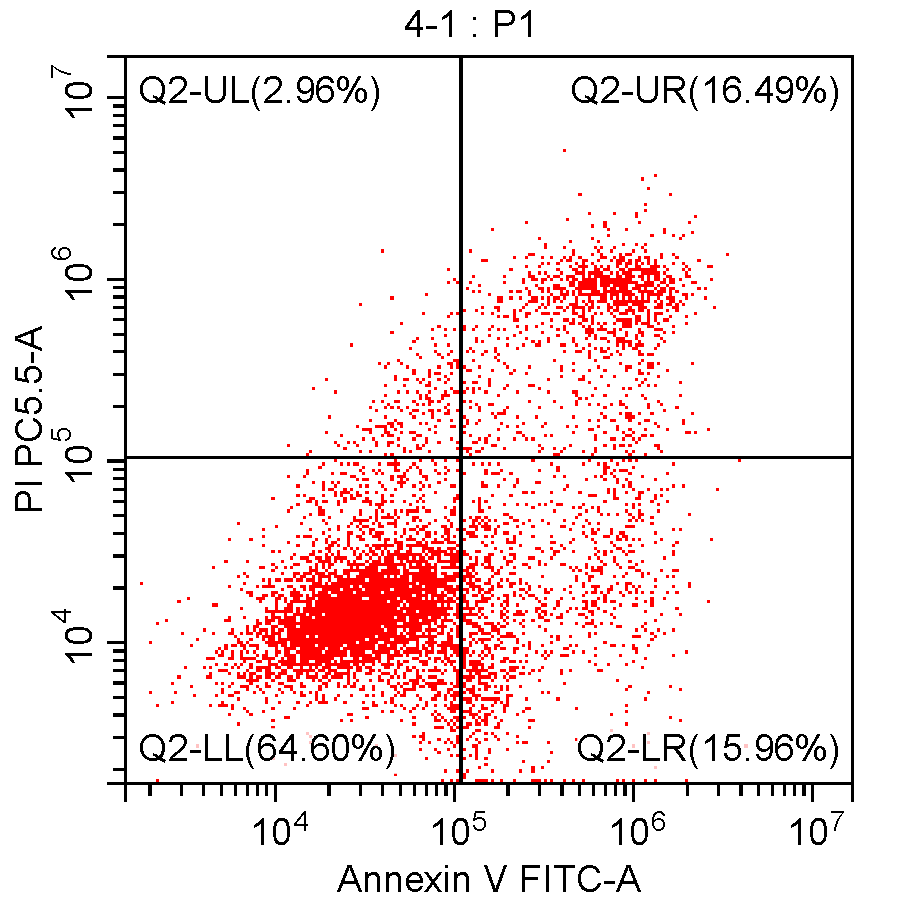

Supplement: Supplementary file 3 [file DataSheet4.ZIP › BT-549/4-1_Plot1.bmp]

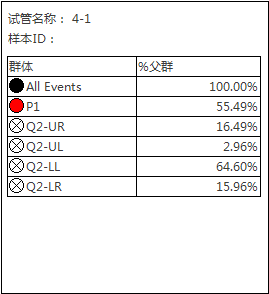

Supplement: Supplementary file 3 [file DataSheet4.ZIP › BT-549/4-1_Statistics1.bmp]

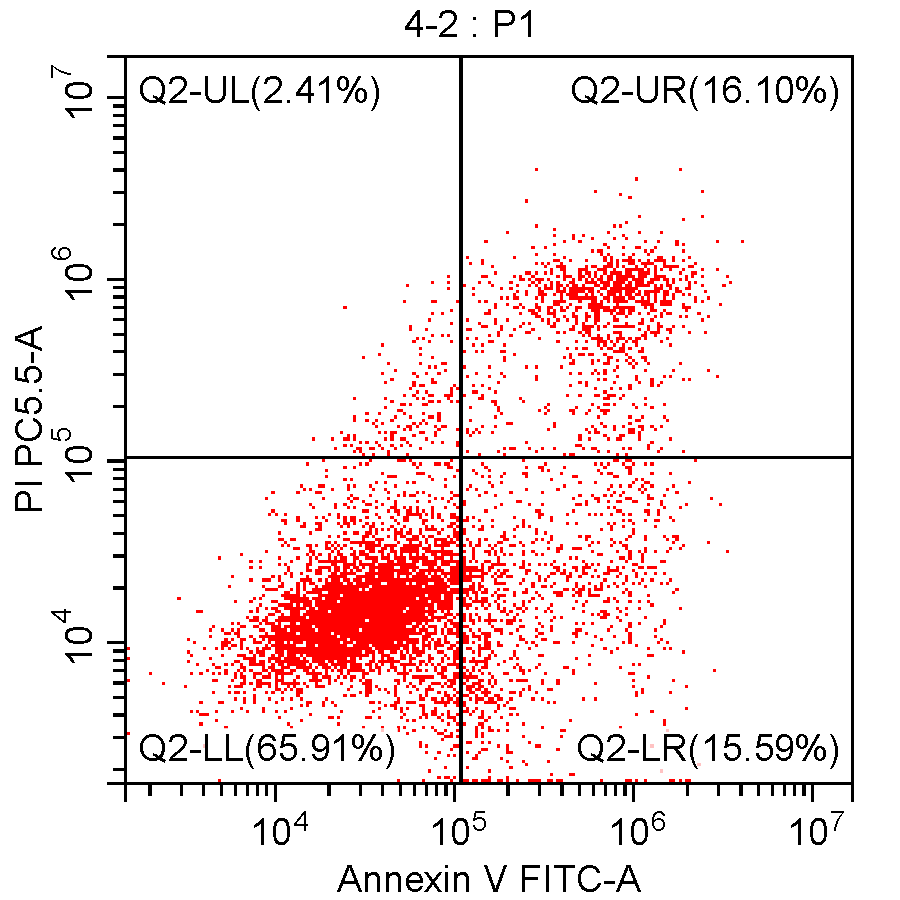

Supplement: Supplementary file 3 [file DataSheet4.ZIP › BT-549/4-2_Plot1.bmp]

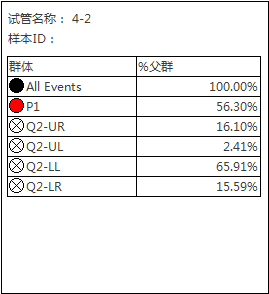

Supplement: Supplementary file 3 [file DataSheet4.ZIP › BT-549/4-2_Statistics1.bmp]

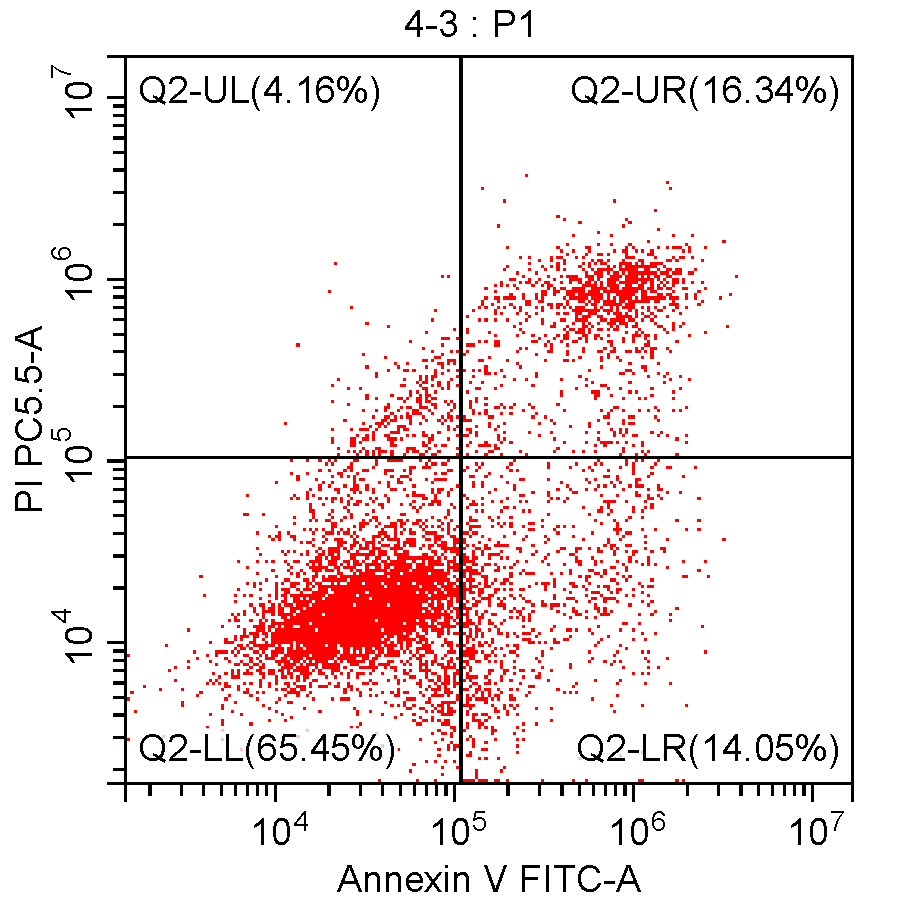

Supplement: Supplementary file 3 [file DataSheet4.ZIP › BT-549/4-3_Plot1.bmp]

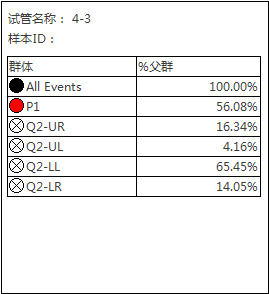

Supplement: Supplementary file 3 [file DataSheet4.ZIP › BT-549/4-3_Statistics1.bmp]

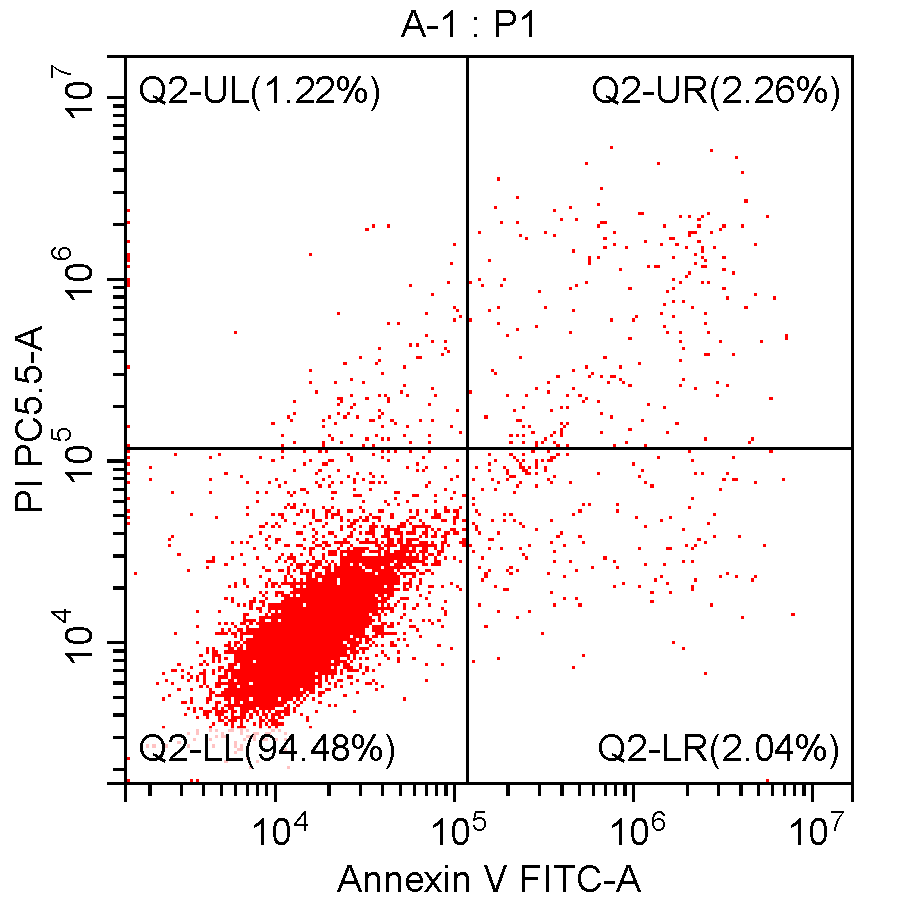

Supplement: Supplementary file 3 [file DataSheet4.ZIP › MDA-MB-231/A-1_Plot1.bmp]

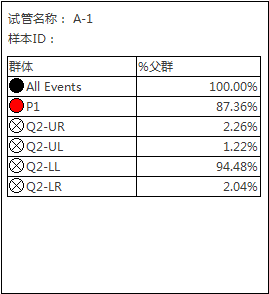

Supplement: Supplementary file 3 [file DataSheet4.ZIP › MDA-MB-231/A-1_Statistics1.bmp]

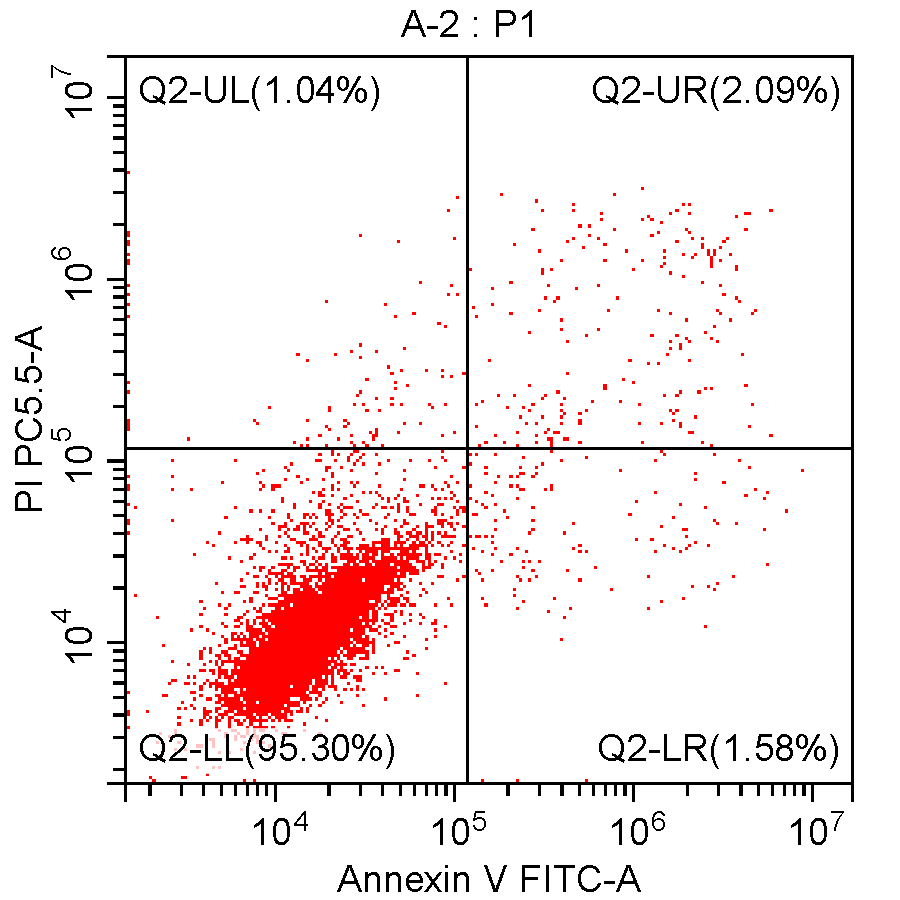

Supplement: Supplementary file 3 [file DataSheet4.ZIP › MDA-MB-231/A-2_Plot1.bmp]

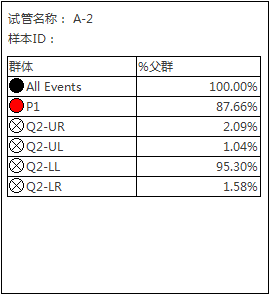

Supplement: Supplementary file 3 [file DataSheet4.ZIP › MDA-MB-231/A-2_Statistics1.bmp]

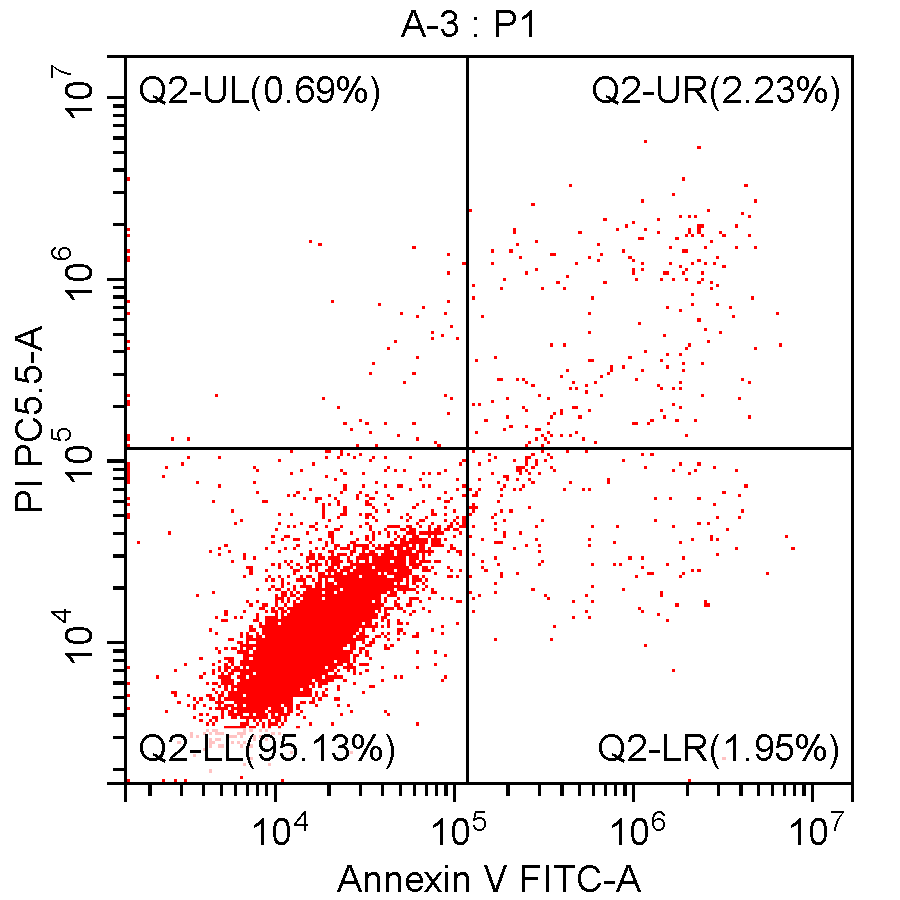

Supplement: Supplementary file 3 [file DataSheet4.ZIP › MDA-MB-231/A-3_Plot1.bmp]

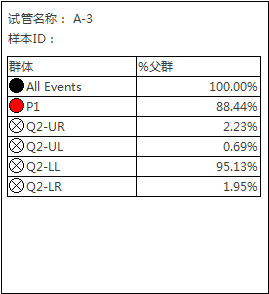

Supplement: Supplementary file 3 [file DataSheet4.ZIP › MDA-MB-231/A-3_Statistics1.bmp]

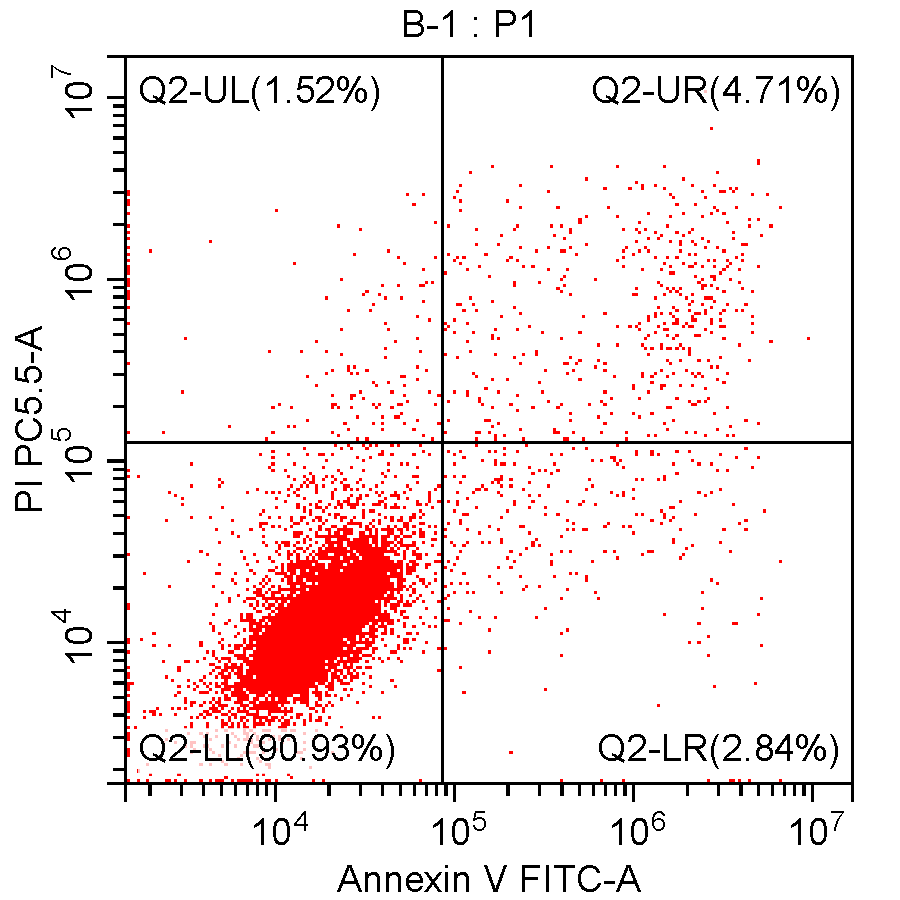

Supplement: Supplementary file 3 [file DataSheet4.ZIP › MDA-MB-231/B-1_Plot1.bmp]

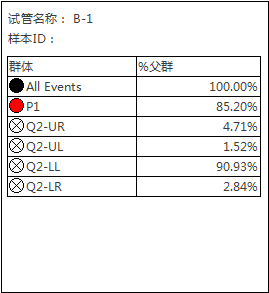

Supplement: Supplementary file 3 [file DataSheet4.ZIP › MDA-MB-231/B-1_Statistics1.bmp]

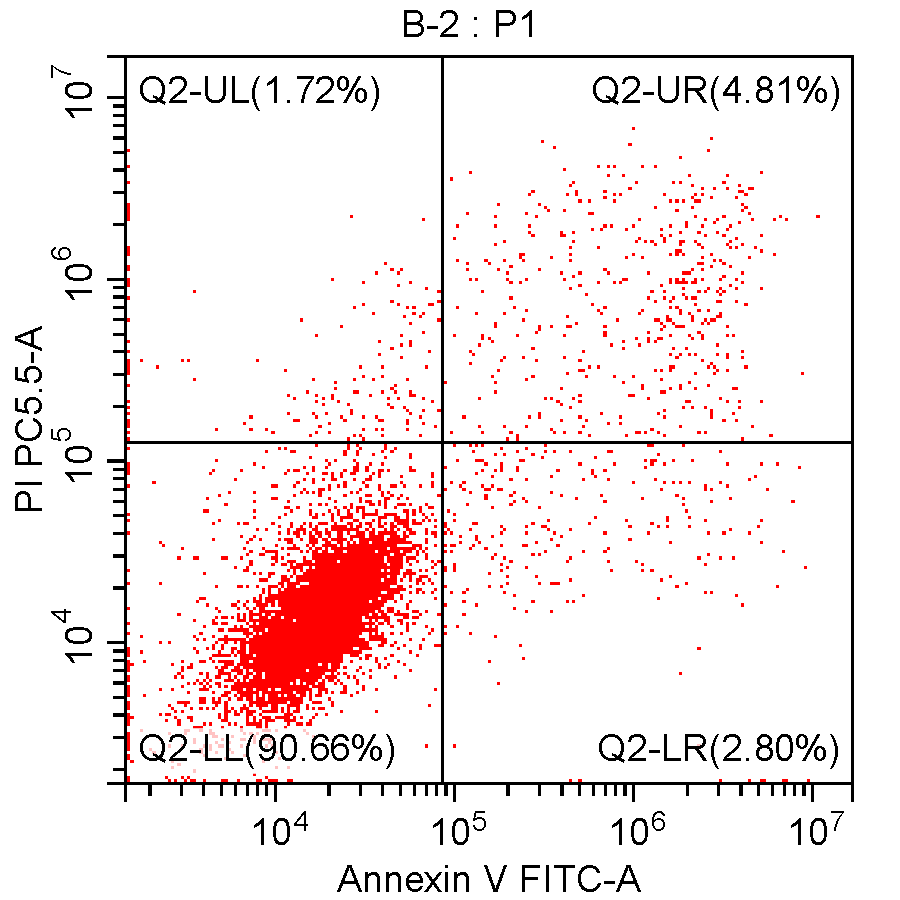

Supplement: Supplementary file 3 [file DataSheet4.ZIP › MDA-MB-231/B-2_Plot1.bmp]

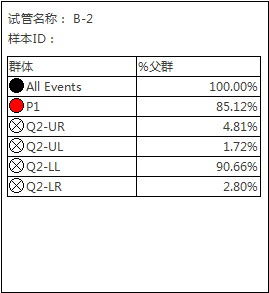

Supplement: Supplementary file 3 [file DataSheet4.ZIP › MDA-MB-231/B-2_Statistics1.bmp]

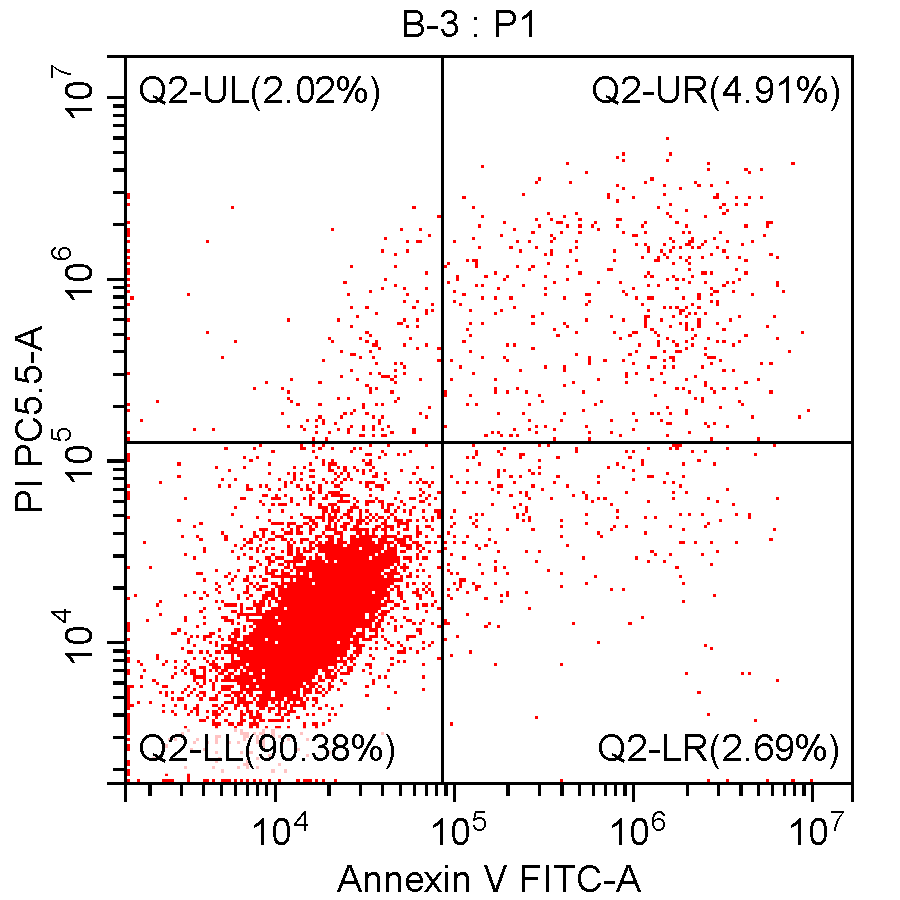

Supplement: Supplementary file 3 [file DataSheet4.ZIP › MDA-MB-231/B-3_Plot1.bmp]

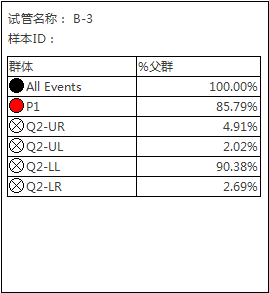

Supplement: Supplementary file 3 [file DataSheet4.ZIP › MDA-MB-231/B-3_Statistics1.bmp]

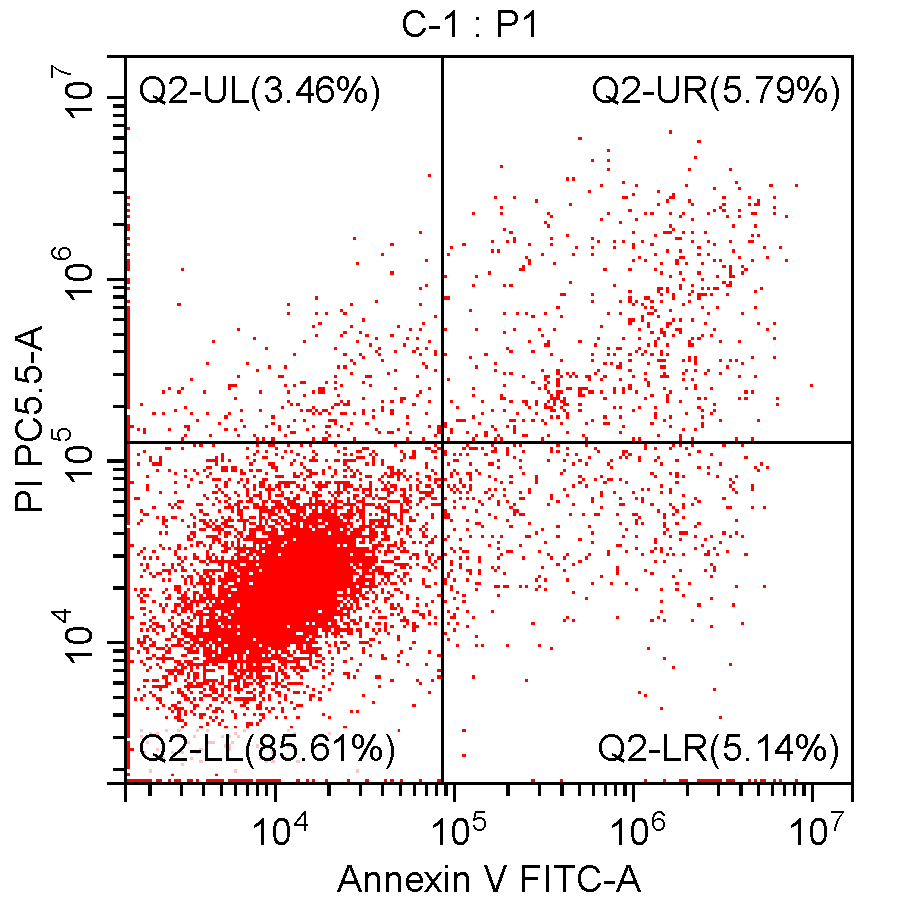

Supplement: Supplementary file 3 [file DataSheet4.ZIP › MDA-MB-231/C-1_Plot1.bmp]

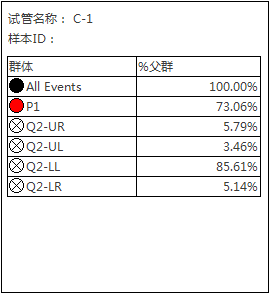

Supplement: Supplementary file 3 [file DataSheet4.ZIP › MDA-MB-231/C-1_Statistics1.bmp]

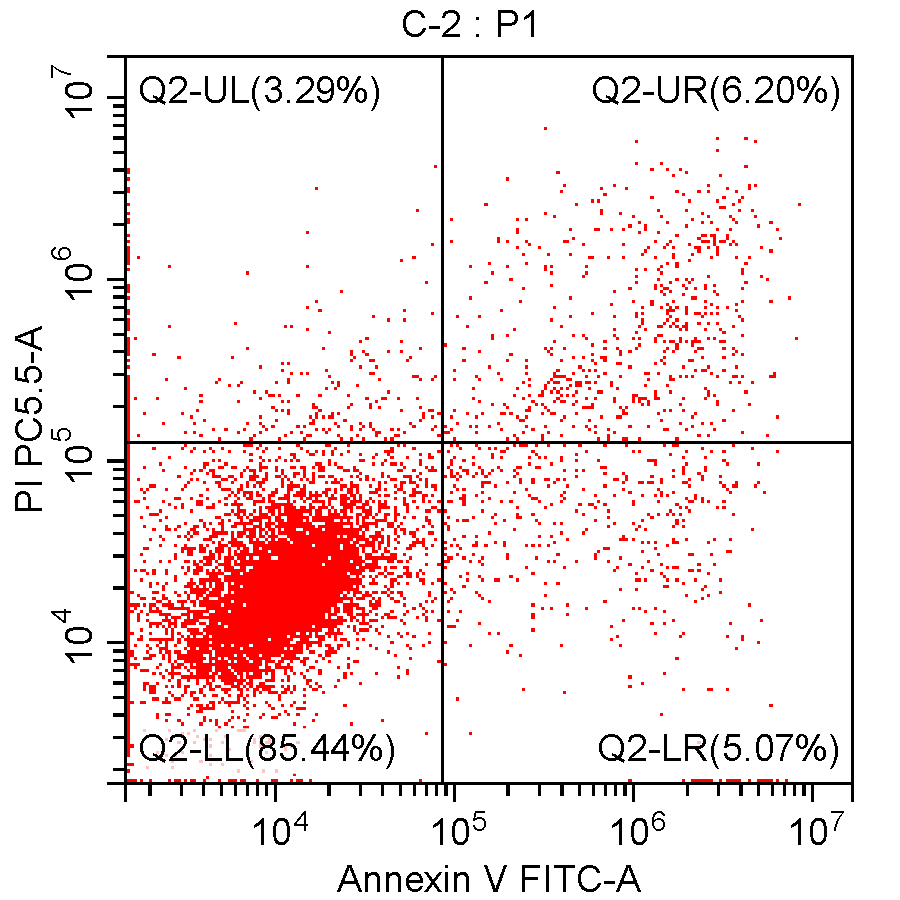

Supplement: Supplementary file 3 [file DataSheet4.ZIP › MDA-MB-231/C-2_Plot1.bmp]

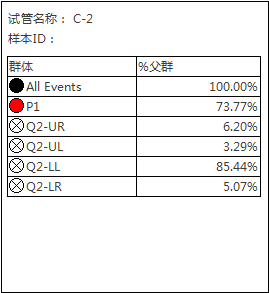

Supplement: Supplementary file 3 [file DataSheet4.ZIP › MDA-MB-231/C-2_Statistics1.bmp]

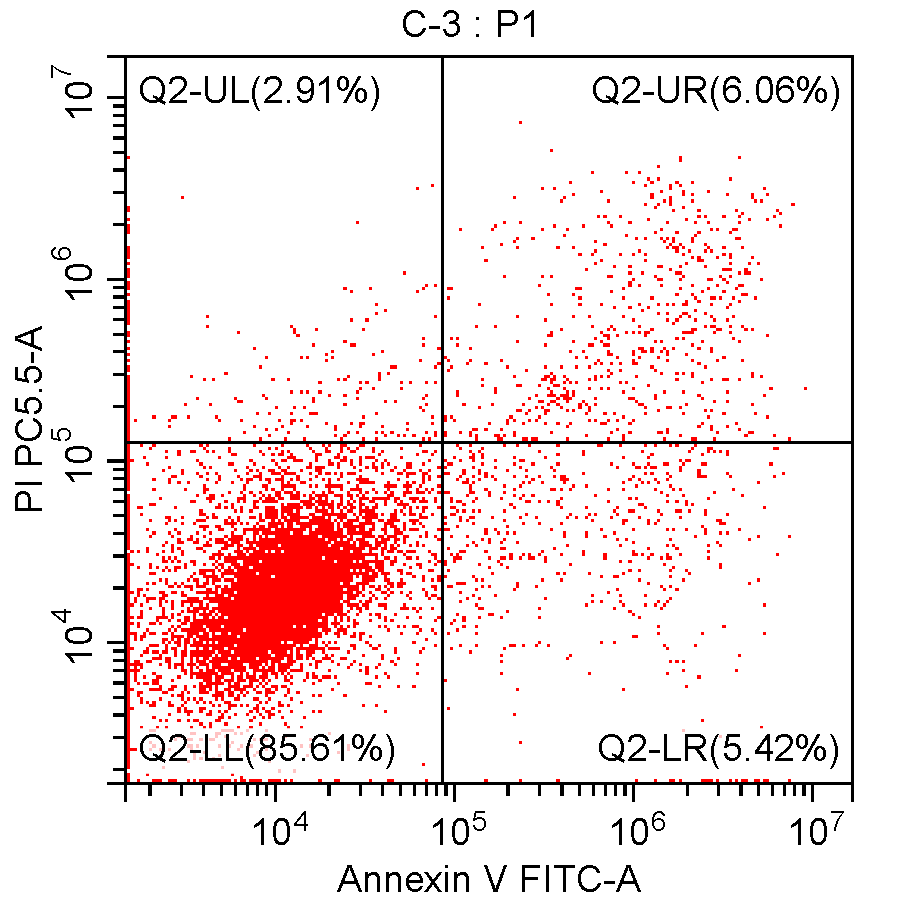

Supplement: Supplementary file 3 [file DataSheet4.ZIP › MDA-MB-231/C-3_Plot1.bmp]

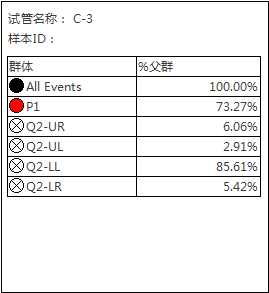

Supplement: Supplementary file 3 [file DataSheet4.ZIP › MDA-MB-231/C-3_Statistics1.bmp]

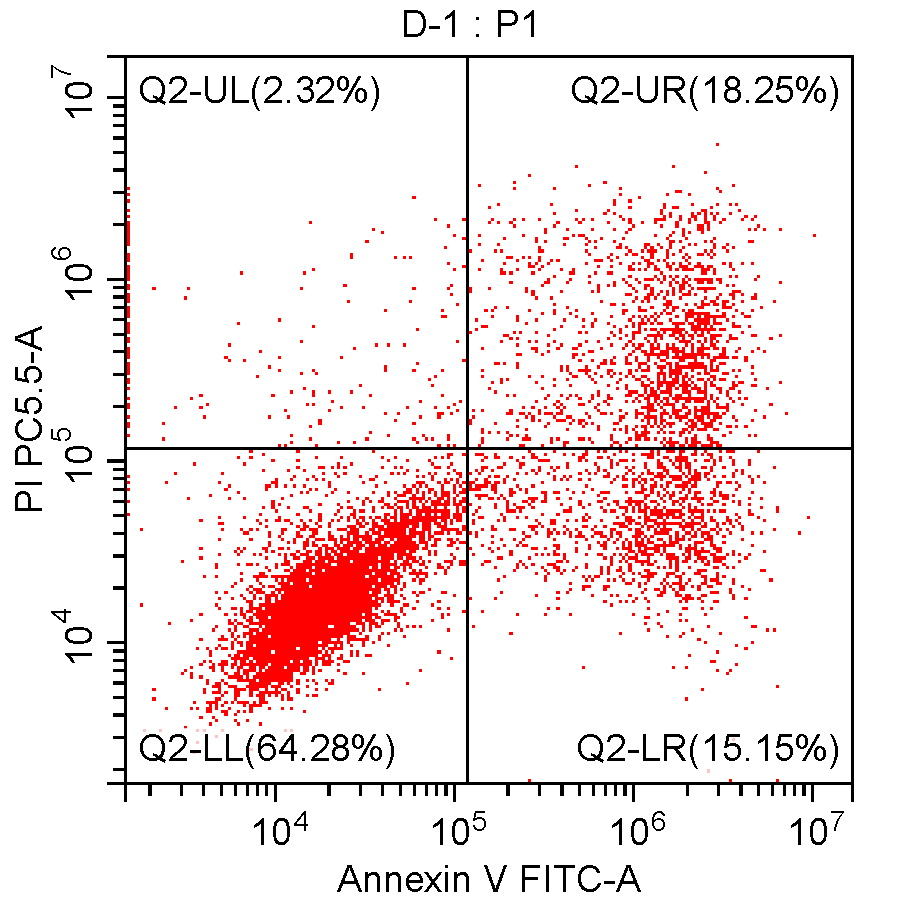

Supplement: Supplementary file 3 [file DataSheet4.ZIP › MDA-MB-231/D-1_Plot1.bmp]

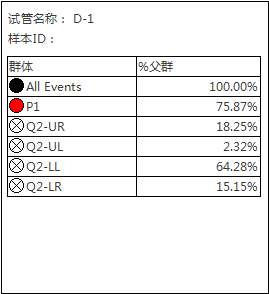

Supplement: Supplementary file 3 [file DataSheet4.ZIP › MDA-MB-231/D-1_Statistics1.bmp]

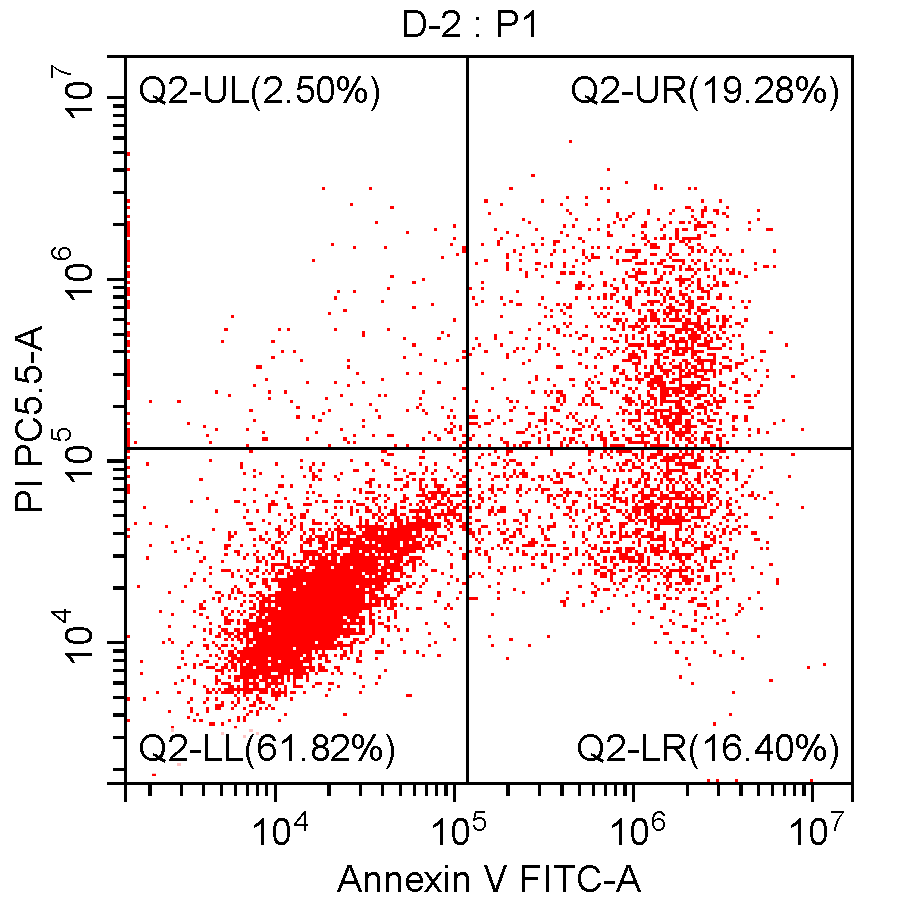

Supplement: Supplementary file 3 [file DataSheet4.ZIP › MDA-MB-231/D-2_Plot1.bmp]

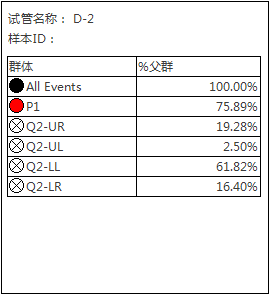

Supplement: Supplementary file 3 [file DataSheet4.ZIP › MDA-MB-231/D-2_Statistics1.bmp]

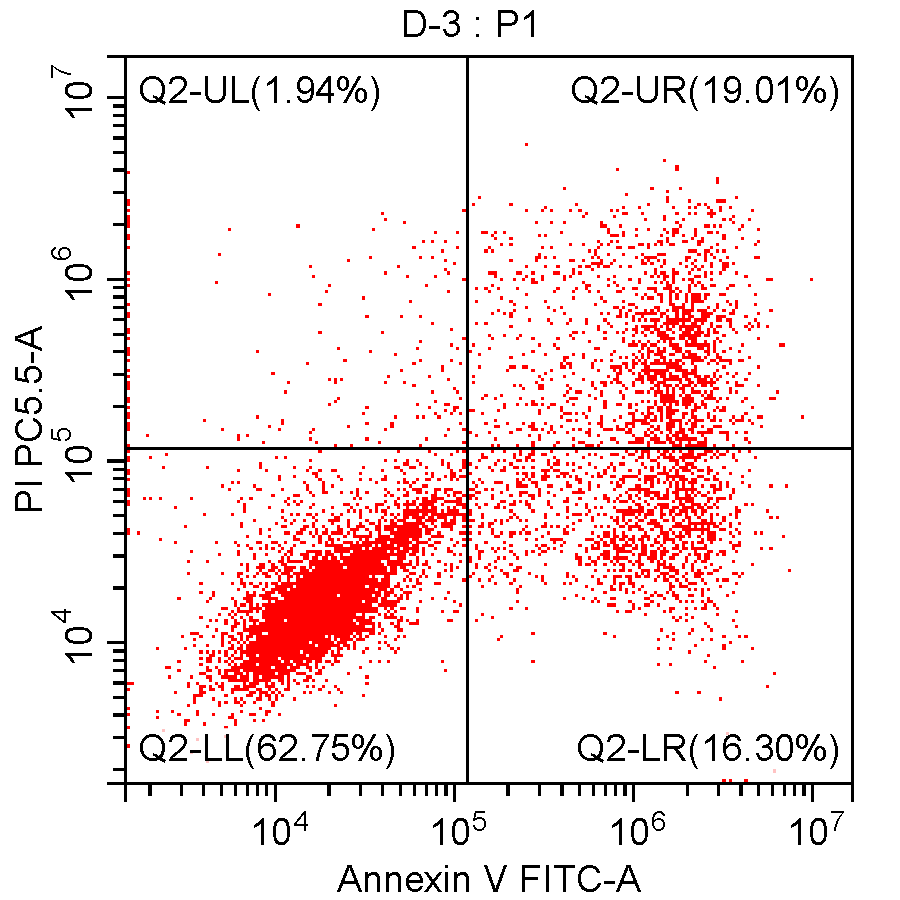

Supplement: Supplementary file 3 [file DataSheet4.ZIP › MDA-MB-231/D-3_Plot1.bmp]

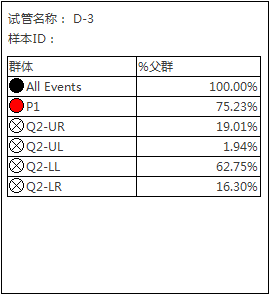

Supplement: Supplementary file 3 [file DataSheet4.ZIP › MDA-MB-231/D-3_Statistics1.bmp]

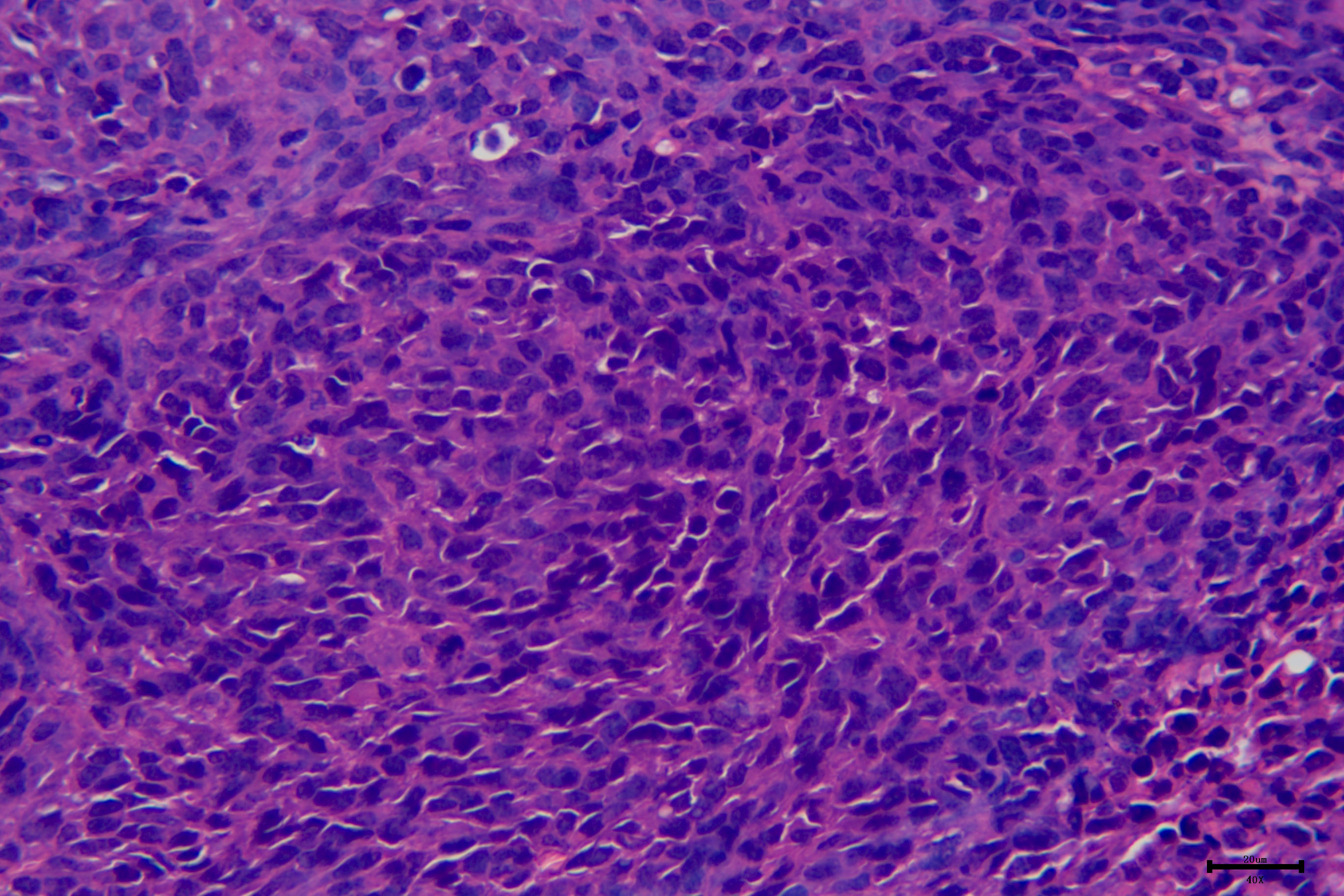

Supplement: Supplementary file 4 [file DataSheet6.ZIP › HE/A-400X.jpg]

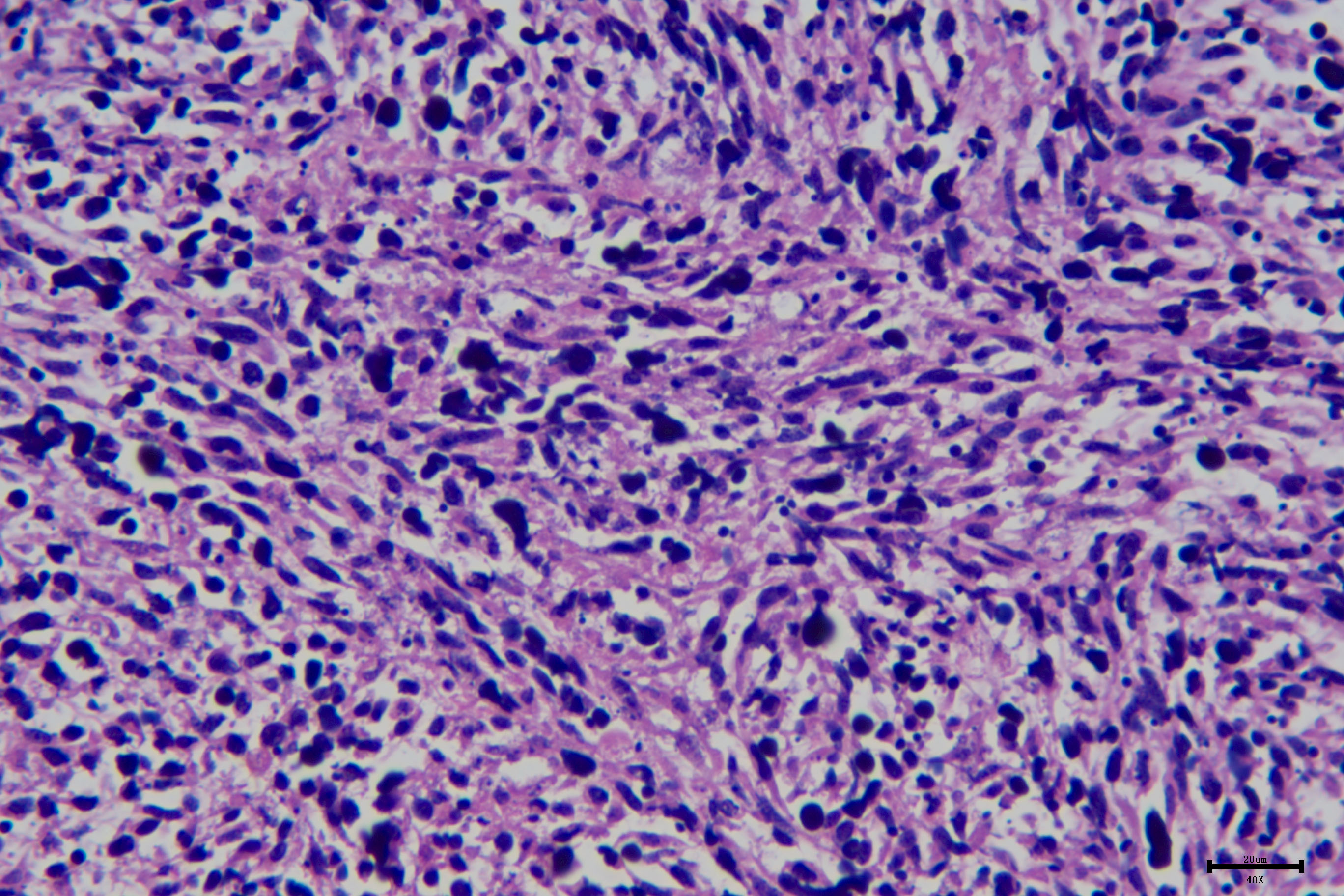

Supplement: Supplementary file 4 [file DataSheet6.ZIP › HE/B-400X.jpg]

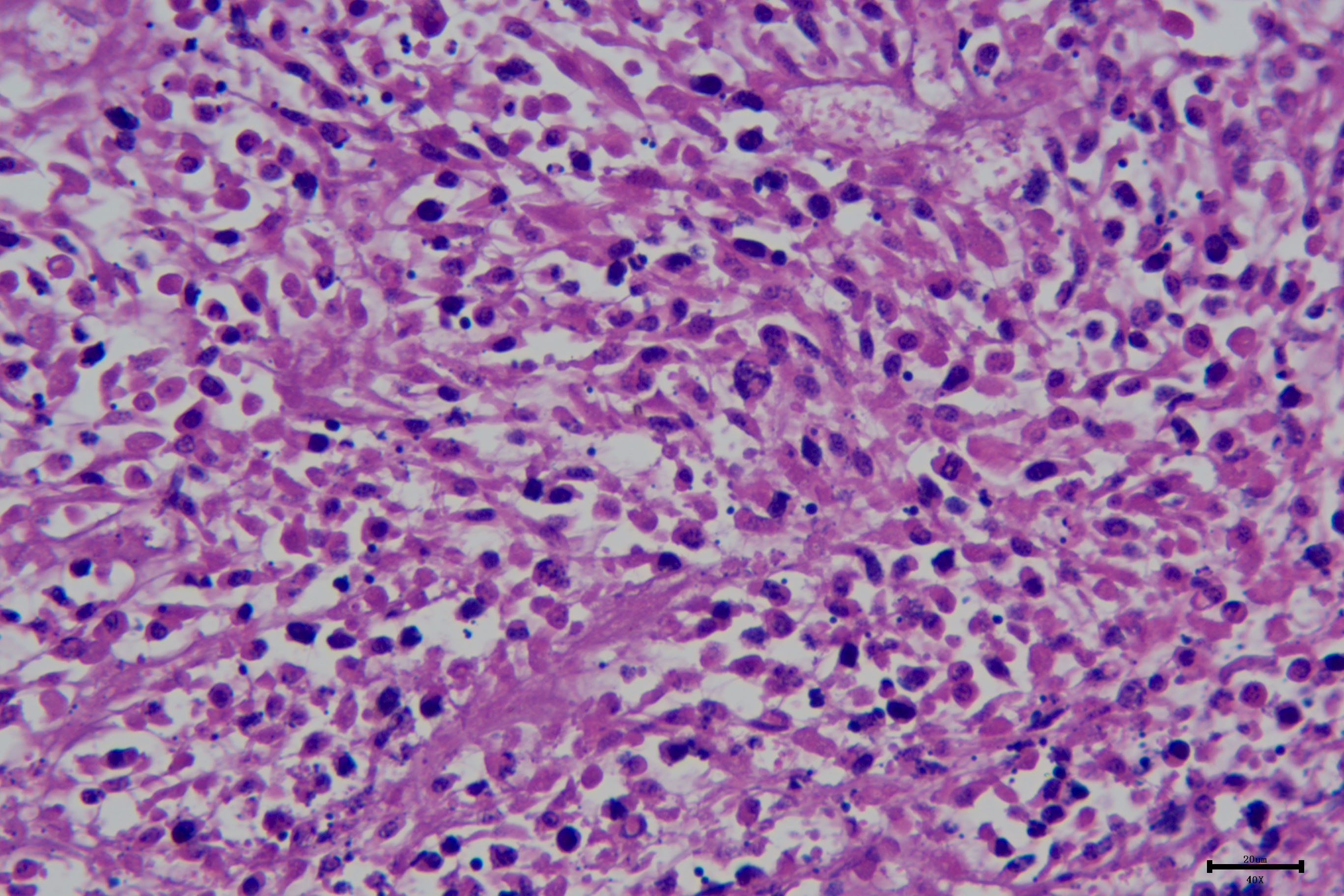

Supplement: Supplementary file 4 [file DataSheet6.ZIP › HE/C-400X.jpg]

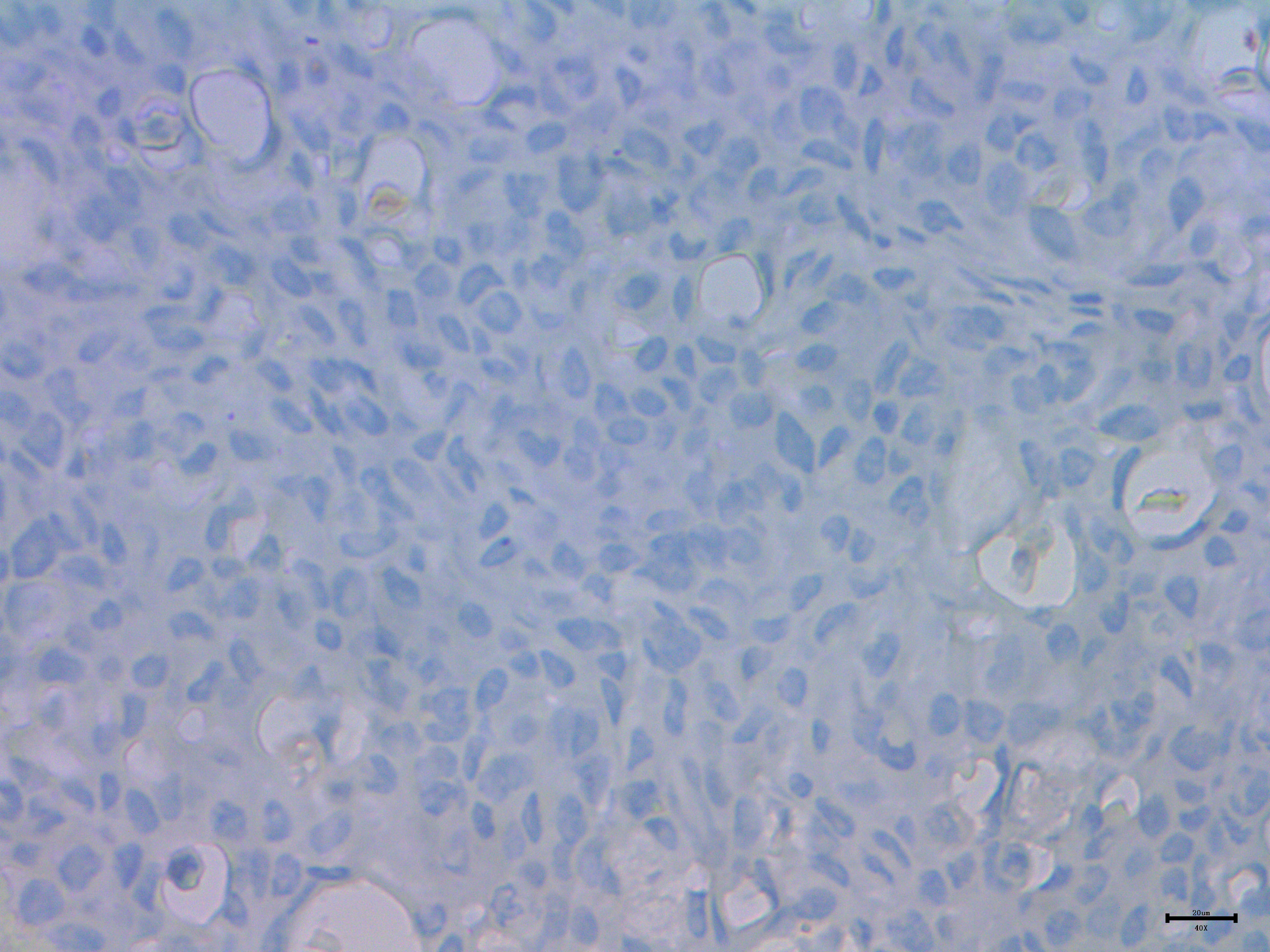

Supplement: Supplementary file 4 [file DataSheet6.ZIP › IHC/A-400X.jpg]

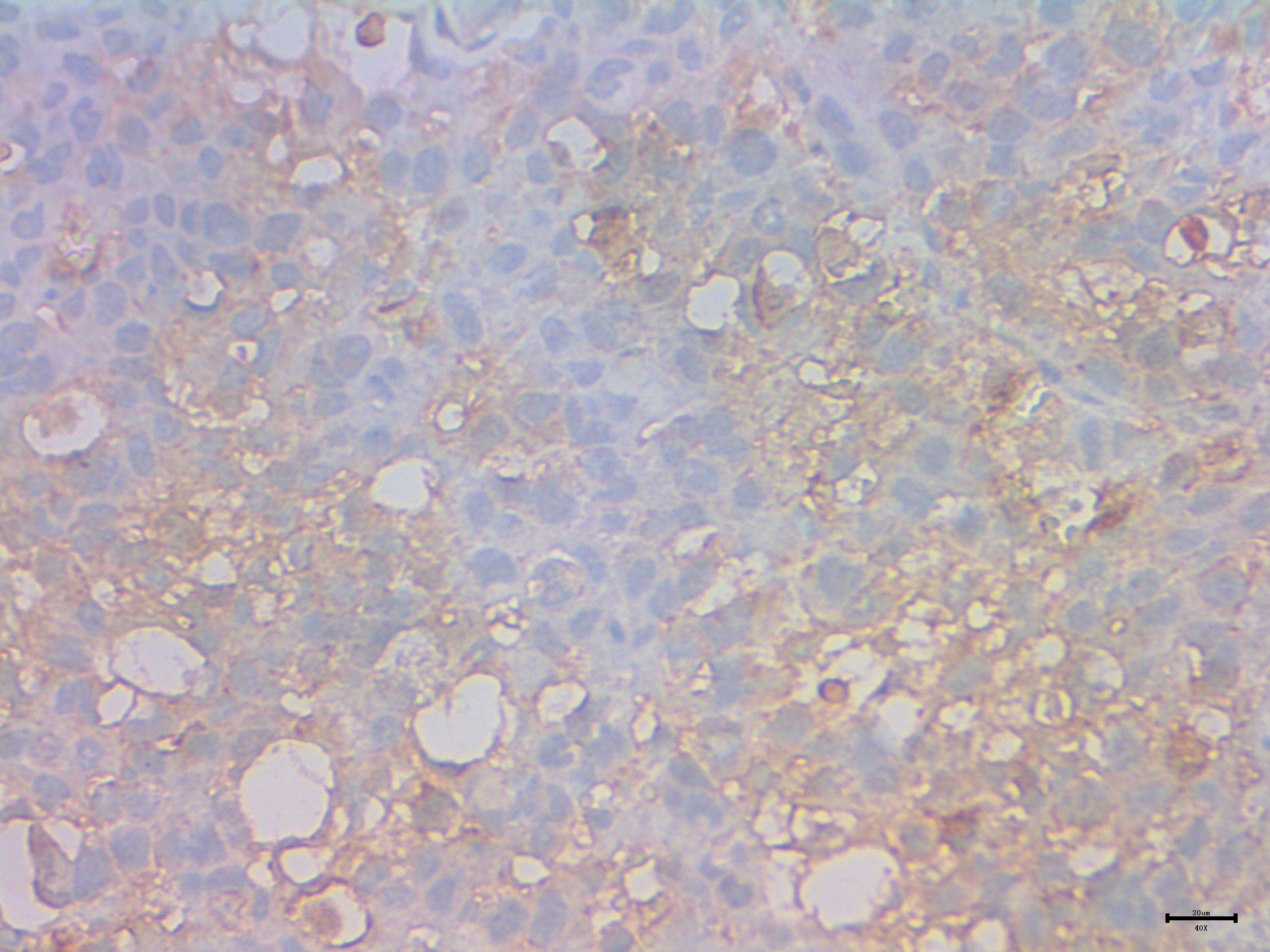

Supplement: Supplementary file 4 [file DataSheet6.ZIP › IHC/B-400X.jpg]

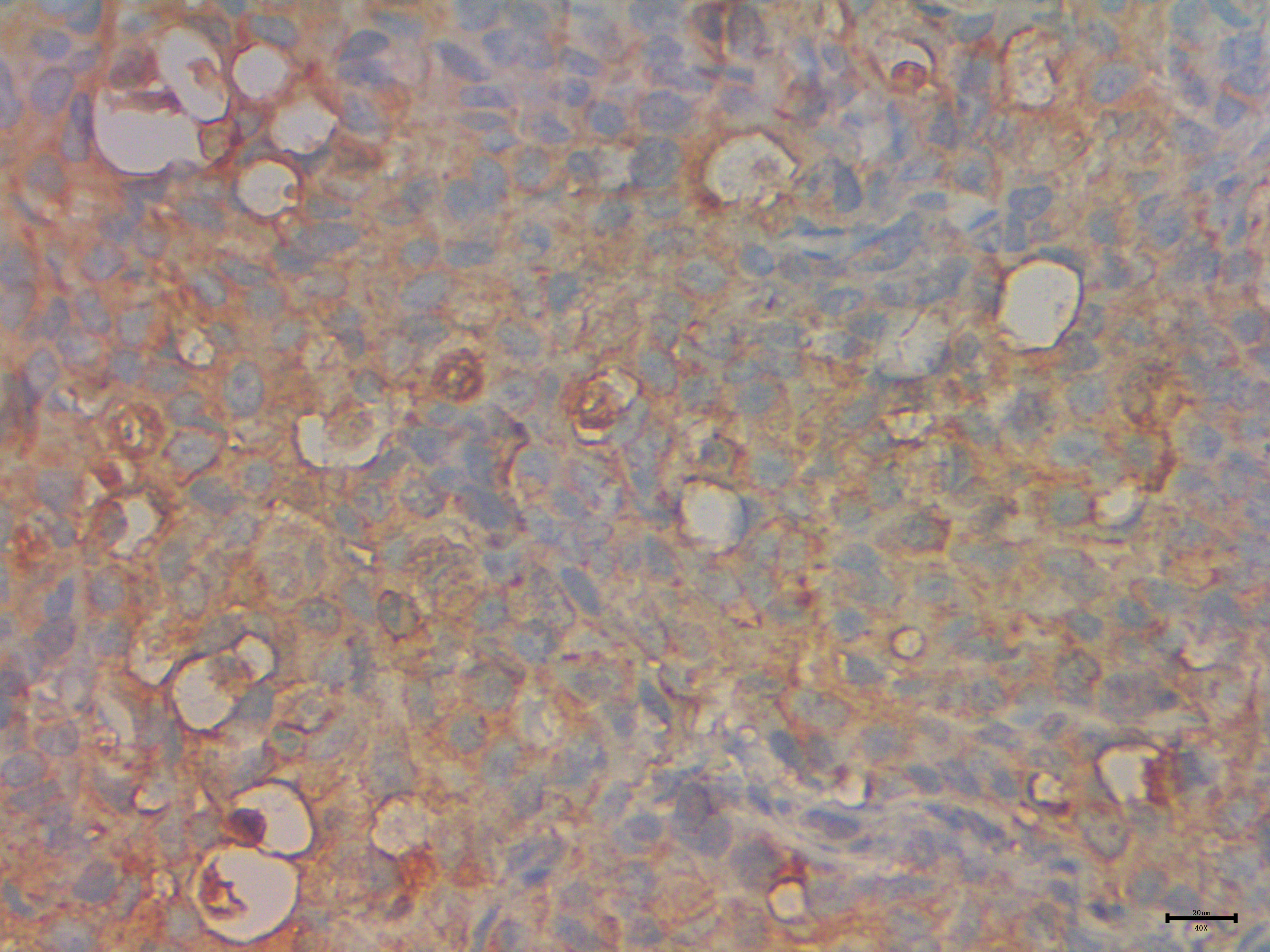

Supplement: Supplementary file 4 [file DataSheet6.ZIP › IHC/C-400X.jpg]

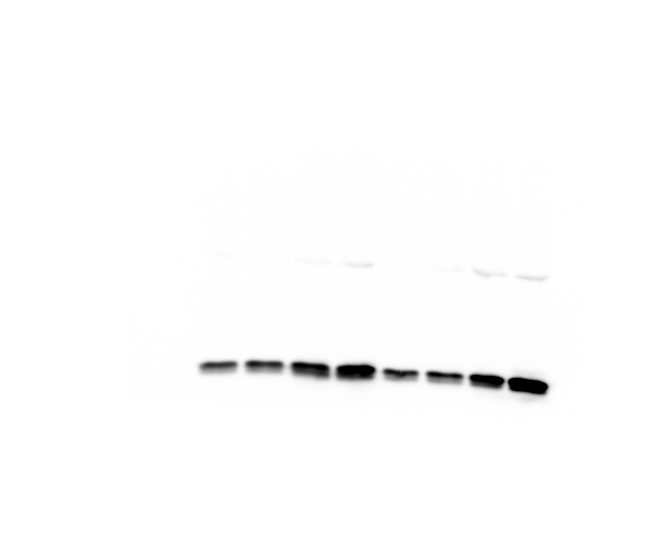

Supplement: Supplementary file 6 [file DataSheet2.ZIP › Western blot/Figure 3B/BAX.tif]

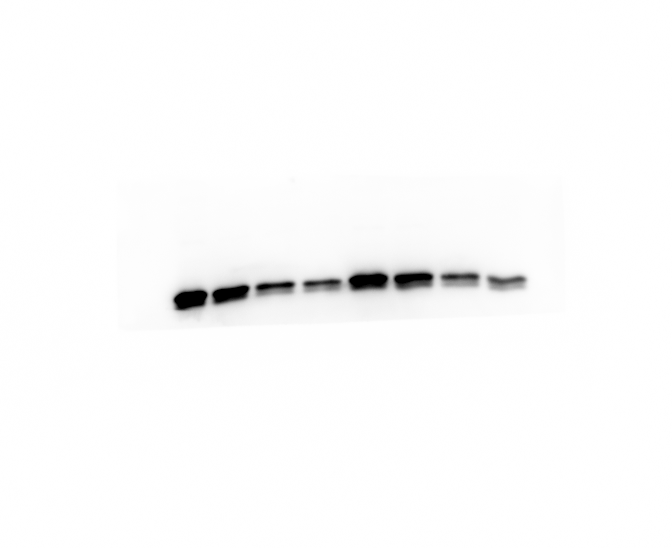

Supplement: Supplementary file 6 [file DataSheet2.ZIP › Western blot/Figure 3B/Bcl2.tif]

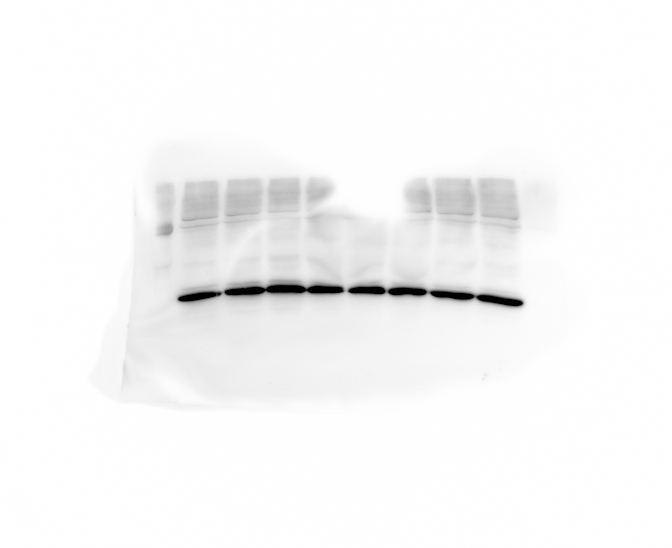

Supplement: Supplementary file 6 [file DataSheet2.ZIP › Western blot/Figure 3B/GAPDH.tif]

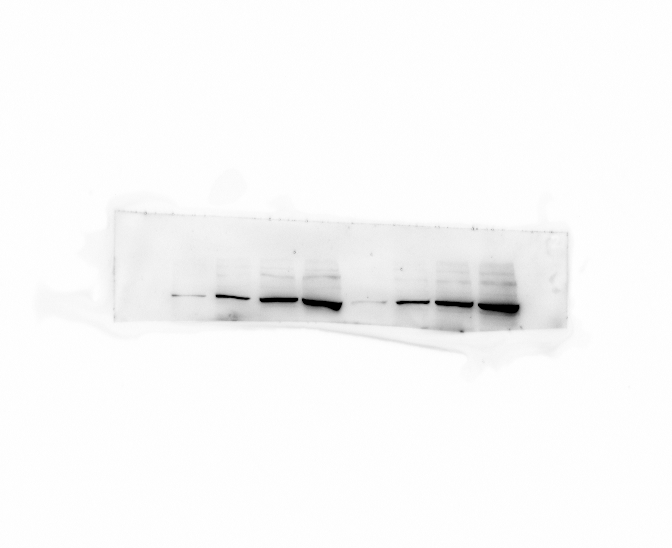

Supplement: Supplementary file 6 [file DataSheet2.ZIP › Western blot/Figure 4C/E-cadherin.tif]

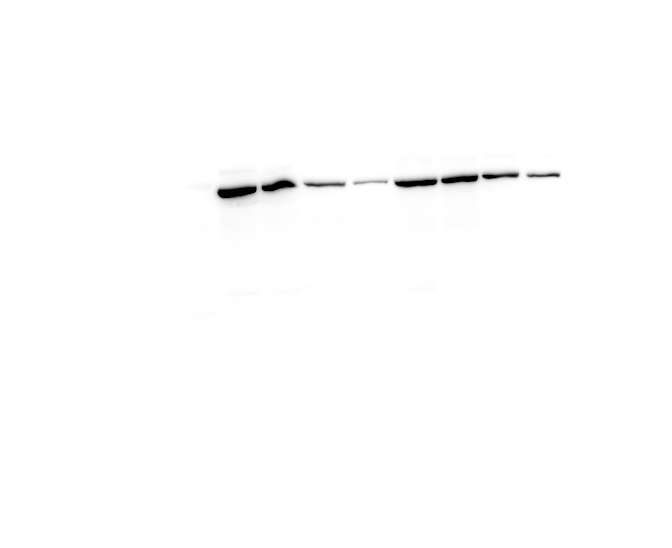

Supplement: Supplementary file 6 [file DataSheet2.ZIP › Western blot/Figure 4C/N-cadherin.tif]

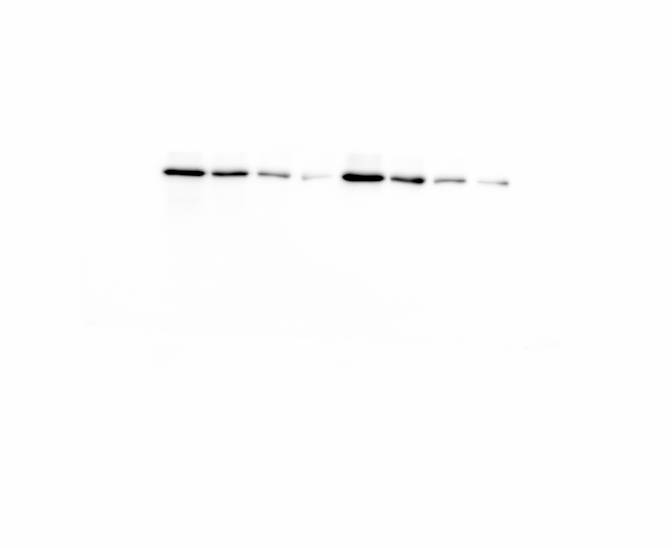

Supplement: Supplementary file 6 [file DataSheet2.ZIP › Western blot/Figure 4C/vimentin.tif]

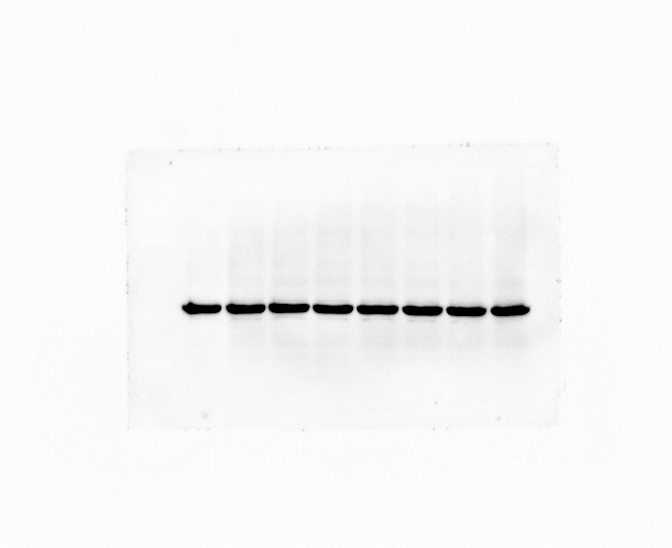

Supplement: Supplementary file 6 [file DataSheet2.ZIP › Western blot/Figure 4C/a┬-actin.tif]

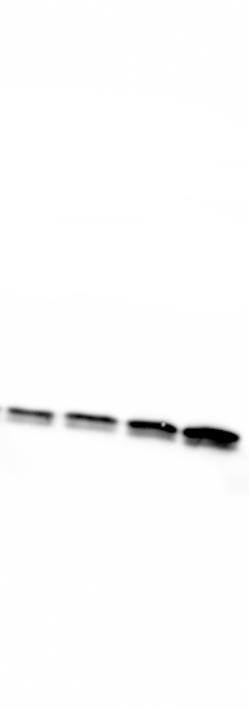

Supplement: Supplementary file 6 [file DataSheet2.ZIP › Western blot/Figure 8A/GADD45a-BT-549.jpg]

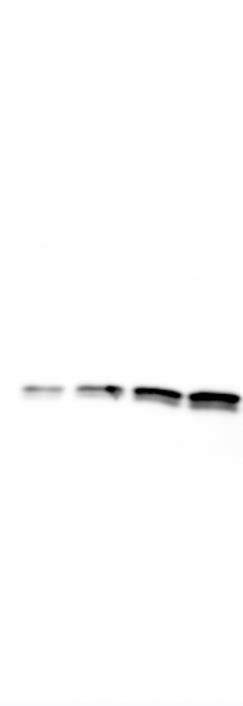

Supplement: Supplementary file 6 [file DataSheet2.ZIP › Western blot/Figure 8A/GADD45a-MDA-MB-231.jpg]

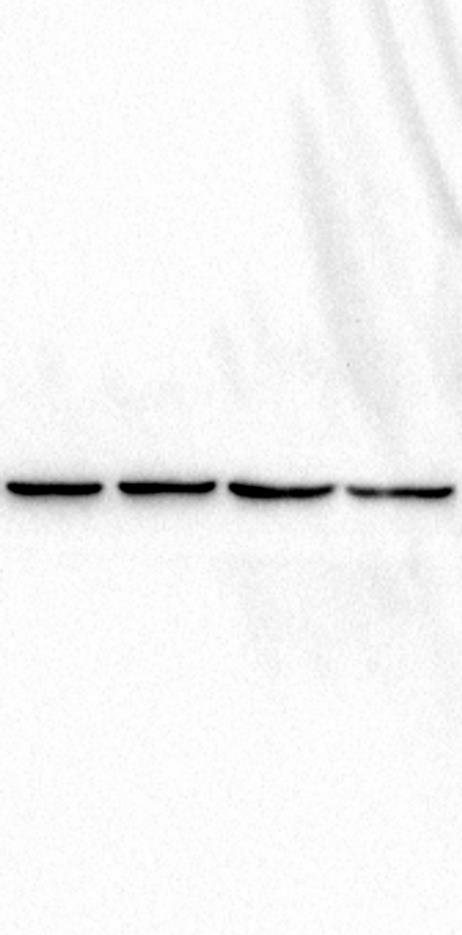

Supplement: Supplementary file 6 [file DataSheet2.ZIP › Western blot/Figure 8A/beta-actin-BT-549.jpg]

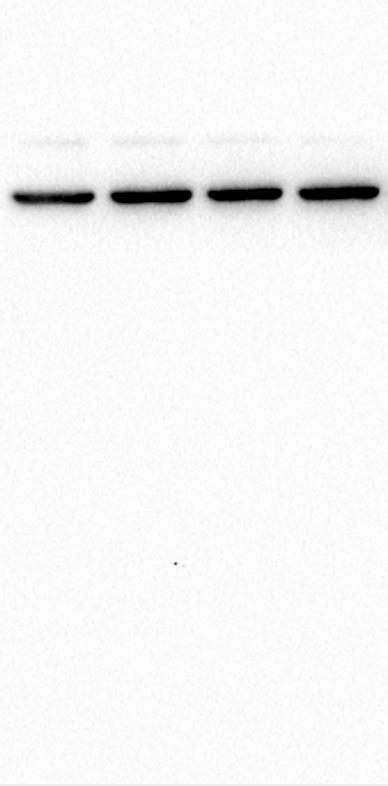

Supplement: Supplementary file 6 [file DataSheet2.ZIP › Western blot/Figure 8A/beta-actin-MDA-MB-231.jpg]

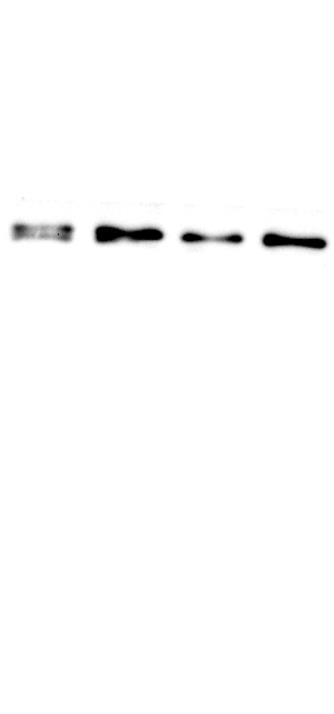

Supplement: Supplementary file 6 [file DataSheet2.ZIP › Western blot/Figure 8E/GADD45A-BT-549.jpg]

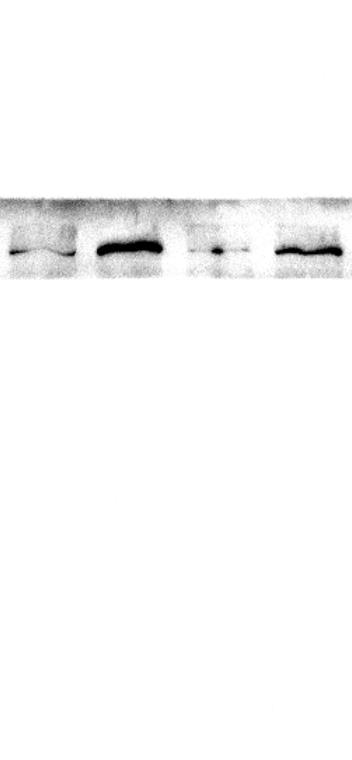

Supplement: Supplementary file 6 [file DataSheet2.ZIP › Western blot/Figure 8E/GADD45A-MDA-MB-231.jpg]

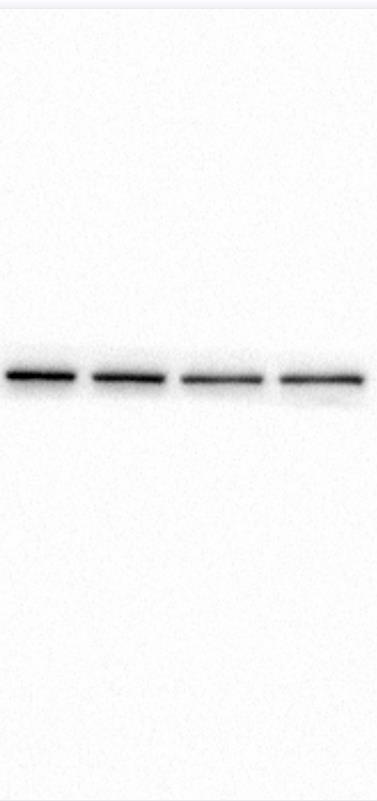

Supplement: Supplementary file 6 [file DataSheet2.ZIP › Western blot/Figure 8E/beta-actin-BT-549.jpg]

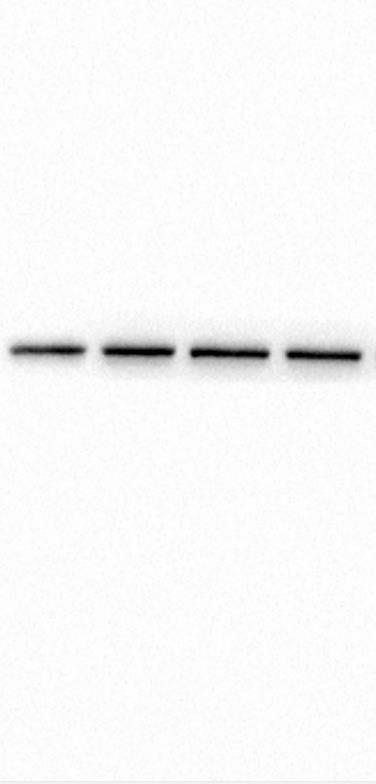

Supplement: Supplementary file 6 [file DataSheet2.ZIP › Western blot/Figure 8E/beta-actin-MDA-MB-231.jpg]

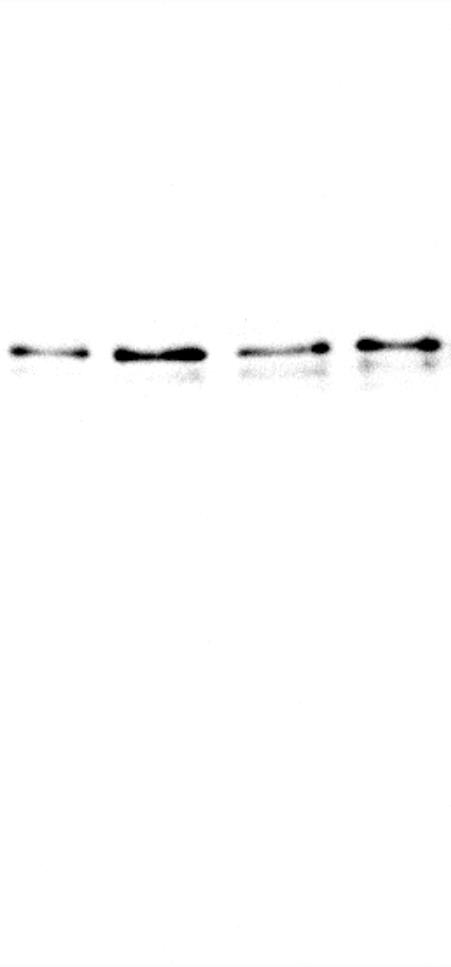

Supplement: Supplementary file 6 [file DataSheet2.ZIP › Western blot/Figure 8F/Bax-BT-549.jpg]

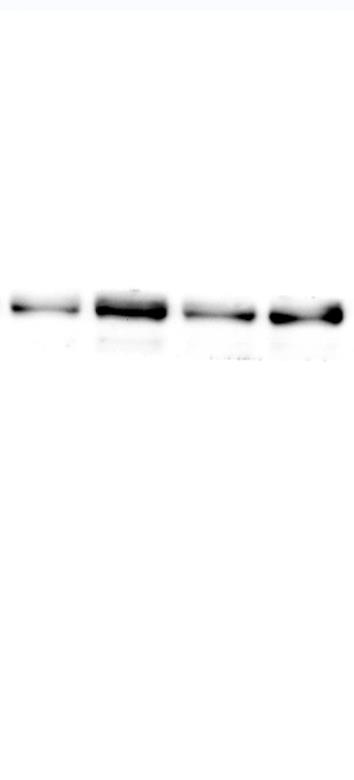

Supplement: Supplementary file 6 [file DataSheet2.ZIP › Western blot/Figure 8F/Bax-MDA-MB-231.jpg]

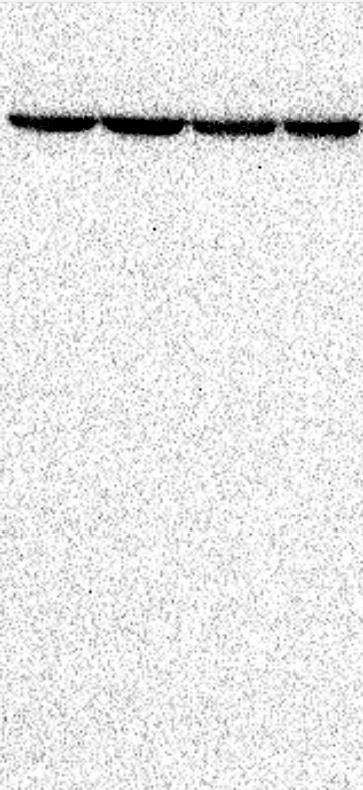

Supplement: Supplementary file 6 [file DataSheet2.ZIP › Western blot/Figure 8F/bata-actin-BT-549.jpg]

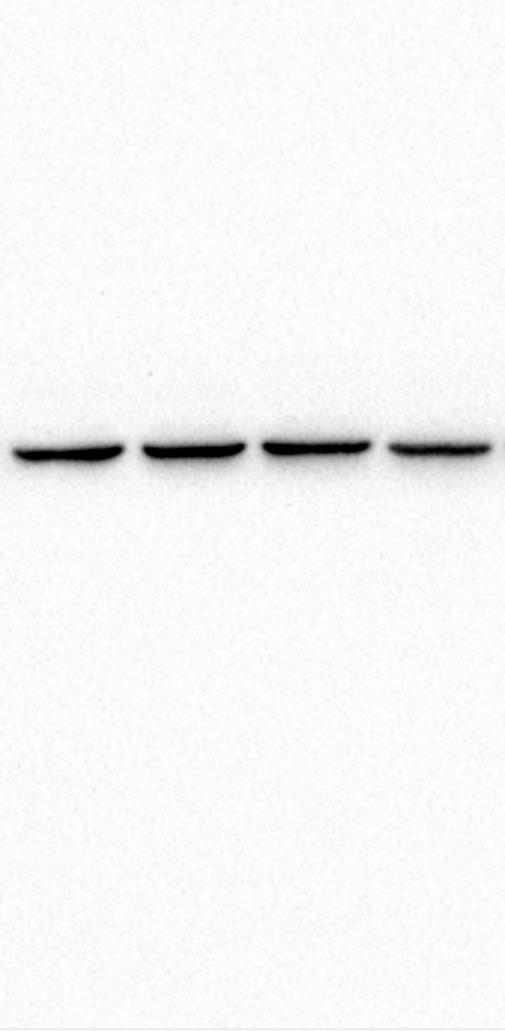

Supplement: Supplementary file 6 [file DataSheet2.ZIP › Western blot/Figure 8F/bata-actin-MDA-MB-231.jpg]

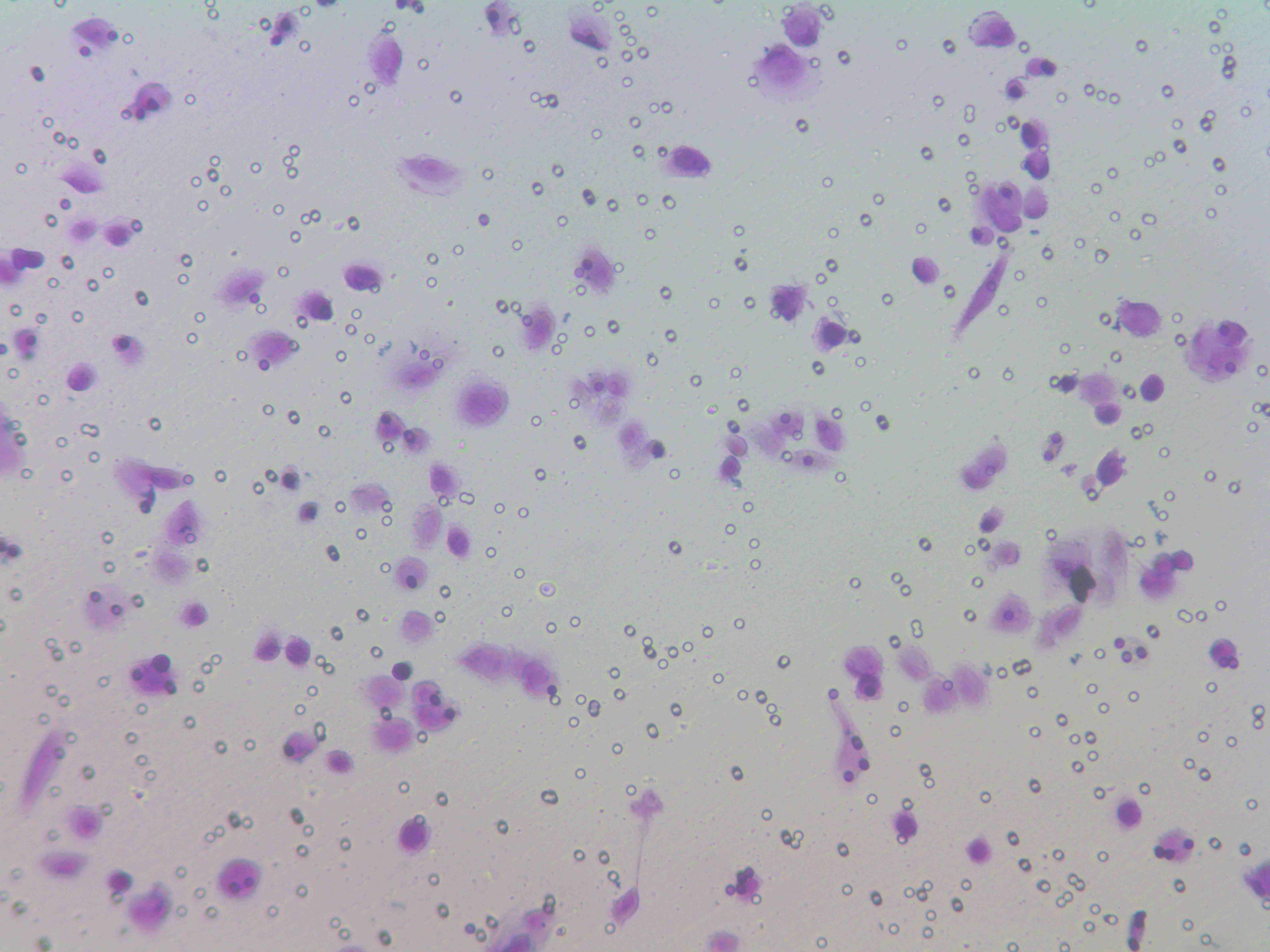

Supplement: Supplementary file 7 [file DataSheet5.ZIP › BT-549/5-1-100X (1)-1_new.jpg]

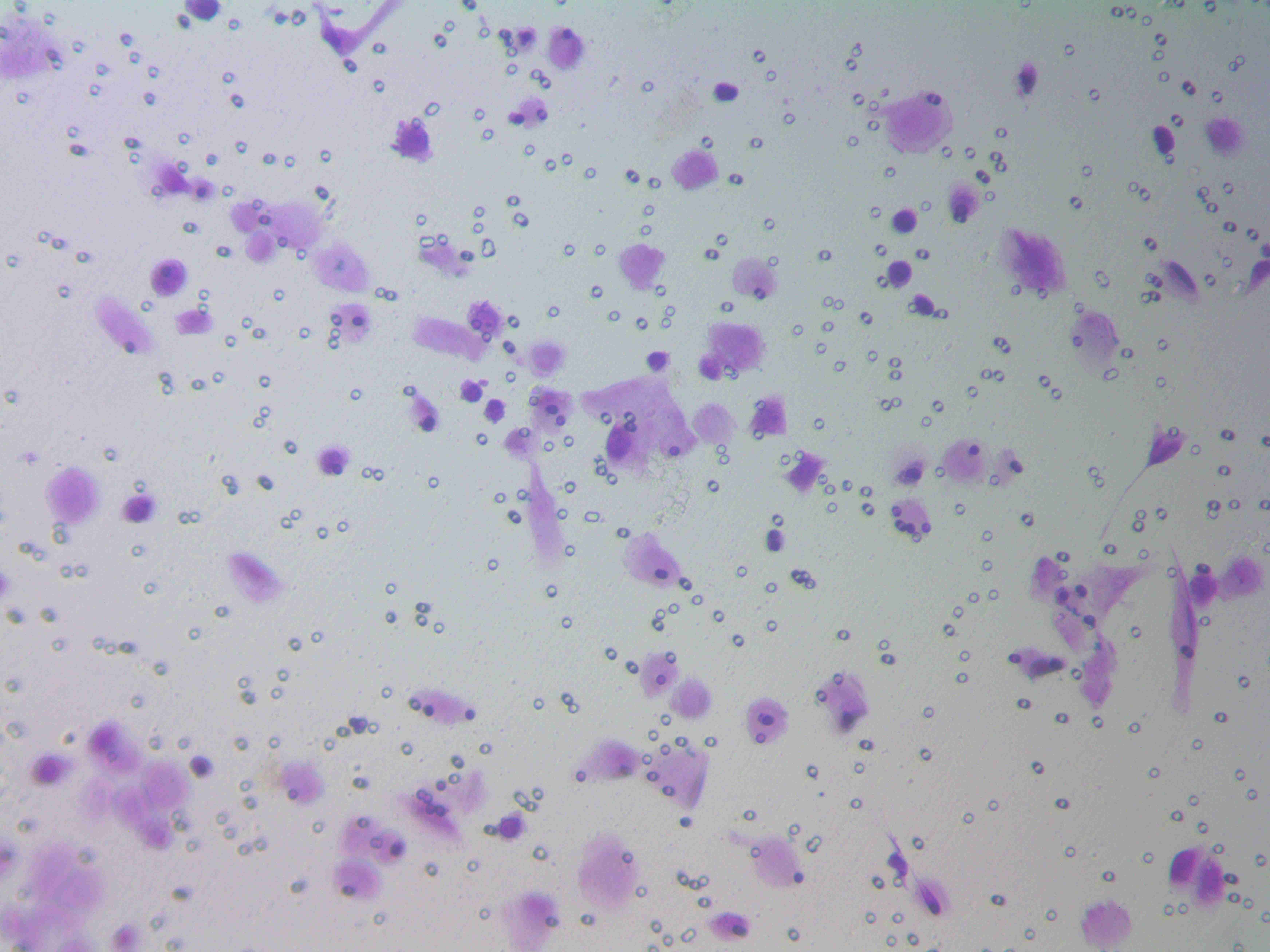

Supplement: Supplementary file 7 [file DataSheet5.ZIP › BT-549/5-3-100X (2)-1_new.jpg]

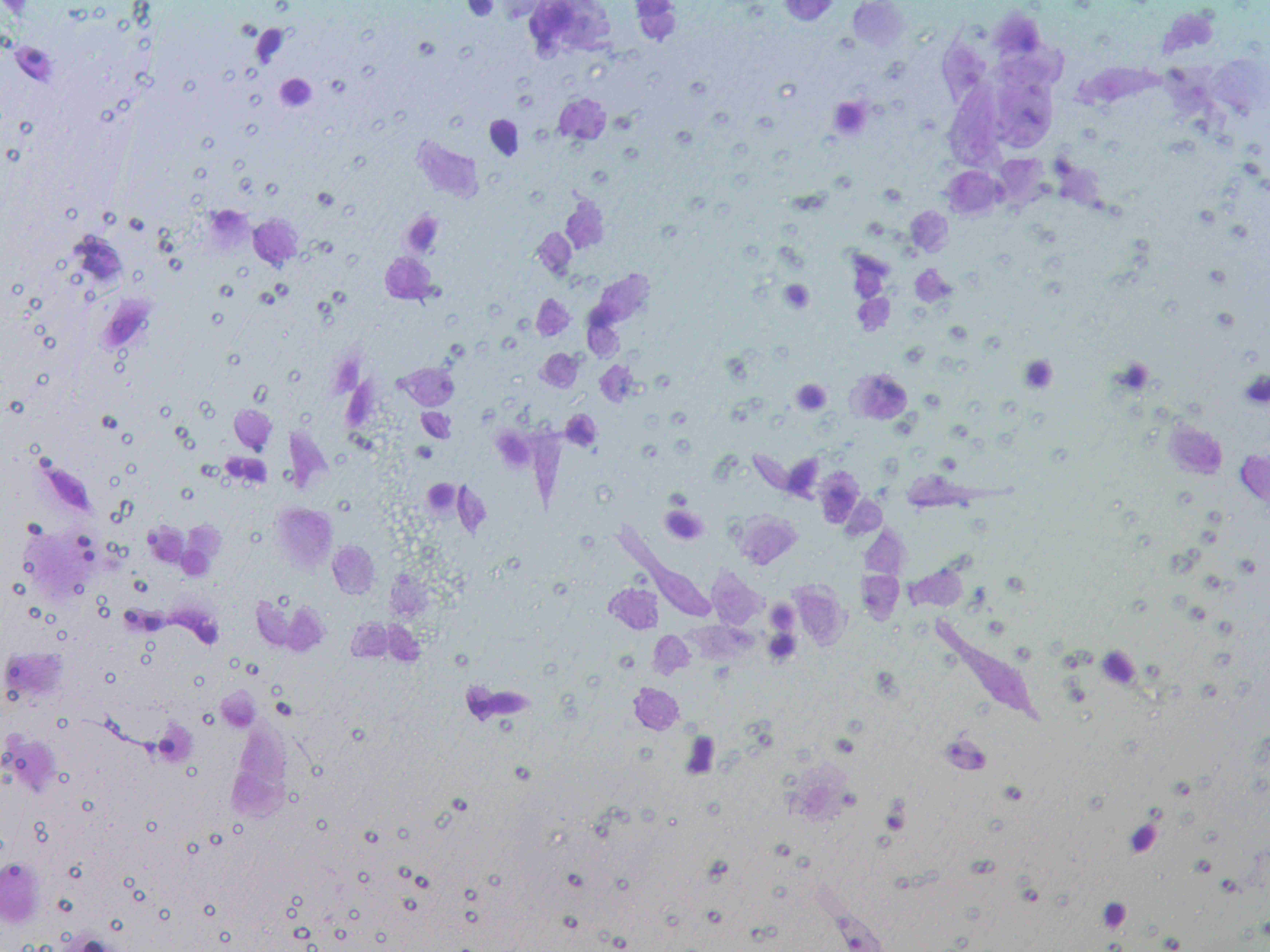

Supplement: Supplementary file 7 [file DataSheet5.ZIP › BT-549/5-3-100X (4)-1_new.jpg]
