# Supplementary material for: Maackiain Modulates miR-374a/GADD45A Axis to Inhibit Triple-Negative Breast Cancer Initiation and Progression
Source: Front Pharmacol. 2022 Mar 4;13:806869. doi: 10.3389/fphar.2022.806869 (PMC8930825; doi:10.3389/fphar.2022.806869)
Supplement: Supplementary file 5 [file DataSheet1.PDF]

Maackiain Modulates miR-374a/GADD45A Axis to inhibit Triple-Negative Breast Cancer Initiation and Progression

Fu Peng [1], Li Wang[1], Liang Xiong[2], Hailin Tang[3], Junrong Du[1]*, Cheng Peng [2]*

[1] Department of Pharmacology, Key Laboratory of Drug-Targeting and Drug Delivery System of the Education Ministry, Sichuan Engineering Laboratory for Plant-Sourced Drug and Sichuan Research Center for Drug Precision Industrial Technology, West China School of Pharmacy, Sichuan University, Chengdu, 610041, China

[2] State Key Laboratory of Southwestern Chinese Medicine Resources, Chengdu University of Traditional Chinese Medicine, Chengdu, 611137, China

[3] Department of Breast Oncology, Sun Yat-sen University Cancer Center, Guangzhou, 510060, China.

Correspondence to: Prof. Cheng Peng, Chengdu University of Traditional Chinese Medicine, No. 1166 Liutai Avenue, Wenjiang District, Chengdu, Sichuan 611137, China. Tel: +86-028-6180-0018, Fax: +86-28-8778-4606, Email: pengchengchengdu@126.com (C.P.); Prof. Junrong Du, No. 17, Section 3, Southern Renmin Rd. Chengdu 610041 China, Tel/fax: +86-28-85501628, Email: dujr_1@163.com (R.D.).

Table S1. The primers used for Real-time PCR

**BAX**	(F) CCGCCGTGGACACAGACT
	(R) TTGAAGTTGCCGTCAGAAAACA
**BCL-2**	(F) CGAGATGTCCAGCCAGCTG
	(R)GATCCAGGTGTGCAGGTGC
**CDH1**	(F) CGACCCAACCCAAGAATCTA
	(R) CTCCAAGAATCCCCAGAATG
**GADD45A**	(F) CCCCGATAACGTGGTGTTGT
	(R) GATGTCGTTCTCGCAGCAAA
**GAPDH**	(F) GACTCATGACCACAGTCCATGC
	(R) AGAGGCAGGGATGATGTTCTG
**U6**	(F) CTCGCTTCGGCAGCACA
	(R) AACGCTTCACGAATTTGCGT

Table S2. The primers used for colony PCR

**GADD45A wide-type**	(F) cgctcgagcaagcagttactccctacactgat
	(R) cgaagcttgatgcaattattcataccagtttattg
**GADD45A mut**	(F) tattaaattctcaaagtttttaatataattctactaagttattttatgacatgaaa
	(R) aaaactttgagaatttaatatctcatcaaa

Table S3. GADD45A-related genes summarized by Pathway Common

GADD45A	controls-expression-of	CASP3
GADD45A	controls-state-change-of	CCNB1
GADD45A	controls-expression-of	CCND1
GADD45A	controls-expression-of	CCND3
GADD45A	controls-state-change-of	CDC25C
GADD45A	controls-state-change-of	CDK1
GADD45A	controls-state-change-of	CDK2
GADD45A	controls-expression-of	CDK6
GADD45A	interacts-with	GADD45B
GADD45A	interacts-with	GADD45GIP1
GADD45A	interacts-with	GADD45G
GADD45A	interacts-with	GAPDH
GADD45A	interacts-with	GBP2
GADD45A	interacts-with	GNB2
GADD45A	in-complex-with	GTSE1
GADD45A	interacts-with	HIST1H1A
GADD45A	interacts-with	HIST1H2AG
GADD45A	interacts-with	HIST1H2AI
GADD45A	interacts-with	HIST1H2AK
GADD45A	interacts-with	HIST1H2AL
GADD45A	interacts-with	HIST1H2AM
GADD45A	interacts-with	HIST2H2BE
GADD45A	interacts-with	HIST3H3
GADD45A	interacts-with	HNRNPA0
GADD45A	interacts-with	HNRNPA1
GADD45A	interacts-with	IGHM
GADD45A	interacts-with	IGSF21
GADD45A	interacts-with	ING4
GADD45A	interacts-with	JMJD1C
GADD45A	in-complex-with	JUN
GADD45A	interacts-with	KHSRP
GADD45A	interacts-with	LMO4
GADD45A	interacts-with	LNX2
GADD45A	controls-phosphorylation-of	MAP2K6
GADD45A	controls-state-change-of	MAP2K6

GADD45A	interacts-with	MAP2K6
GADD45A	controls-state-change-of	MAP3K4
GADD45A	in-complex-with	MAP3K4
GADD45A	interacts-with	MAP3K4
GADD45A	interacts-with	MAPK14
GADD45A	interacts-with	MAPK1
GADD45A	interacts-with	MASP1
GADD45A	interacts-with	MAX
GADD45A	interacts-with	MDM2
GADD45A	interacts-with	MICAL1
GADD45A	interacts-with	MIDN
GADD45A	interacts-with	MPP1
GADD45A	interacts-with	MTOR
GADD45A	in-complex-with	NCL
GADD45A	interacts-with	NPM1
GADD45A	interacts-with	NRBP1
GADD45A	interacts-with	NUCB2
GADD45A	interacts-with	PARVG
GADD45A	in-complex-with	PCNA
GADD45A	interacts-with	PCNA
GADD45A	interacts-with	PFKFB4
GADD45A	interacts-with	PML
GADD45A	interacts-with	PPARA
GADD45A	in-complex-with	PRMT1
GADD45A	interacts-with	PSMC3
GADD45A	interacts-with	PTN
GADD45A	interacts-with	PTPRK
GADD45A	interacts-with	QARS
GADD45A	interacts-with	RAI1
GADD45A	interacts-with	RARA
GADD45A	interacts-with	RBPJ
GADD45A	interacts-with	RPS2
GADD45A	interacts-with	RPS7
GADD45A	interacts-with	RXRA
GADD45A	interacts-with	SH3GLB1
GADD45A	interacts-with	SMARCB1
GADD45A	interacts-with	SMARCC1
GADD45A	interacts-with	SPERT
GADD45A	interacts-with	SPTBN4
GADD45A	interacts-with	STAT3
GADD45A	interacts-with	SYNE4
GADD45A	interacts-with	TAF1
GADD45A	interacts-with	TDG
GADD45A	interacts-with	TIAL1
GADD45A	interacts-with	TLE1
GADD45A	in-complex-with	TP53
GADD45A	interacts-with	TP53

GADD45A	interacts-with	UBE2D1
GADD45A	interacts-with	UBR1
GADD45A	interacts-with	VIM
GADD45A	interacts-with	ZHX1
GADD45A	interacts-with	ZNF135

IC_{50} Regression Results [Data 1]

Parameter	Value
IC_{50}	25.2385
Equation	$Y = 4.8167 + \frac{97.4712 - 4.8167}{1 + (\frac{X}{25.2385})^{2.2177}}$
Equation Form	$Y = Min + \frac{Max - Min}{1 + (\frac{X}{IC_{50}})^{Hill\ coefficient}}$

IC_{50} Regression Results [Data 1]

Parameter	Value
IC_{50}	20.9887
Equation	$Y = 0.5424 + \frac{97.0944 - 0.5424}{1 + (\frac{X}{20.9887})^{1.9348}}$
Equation Form	$Y = Min + \frac{Max - Min}{1 + (\frac{X}{IC_{50}})^{Hill\ coefficient}}$

Figure S1. The IC_{50} values of MA on MDA-MB-231 and BT-549, respectively.

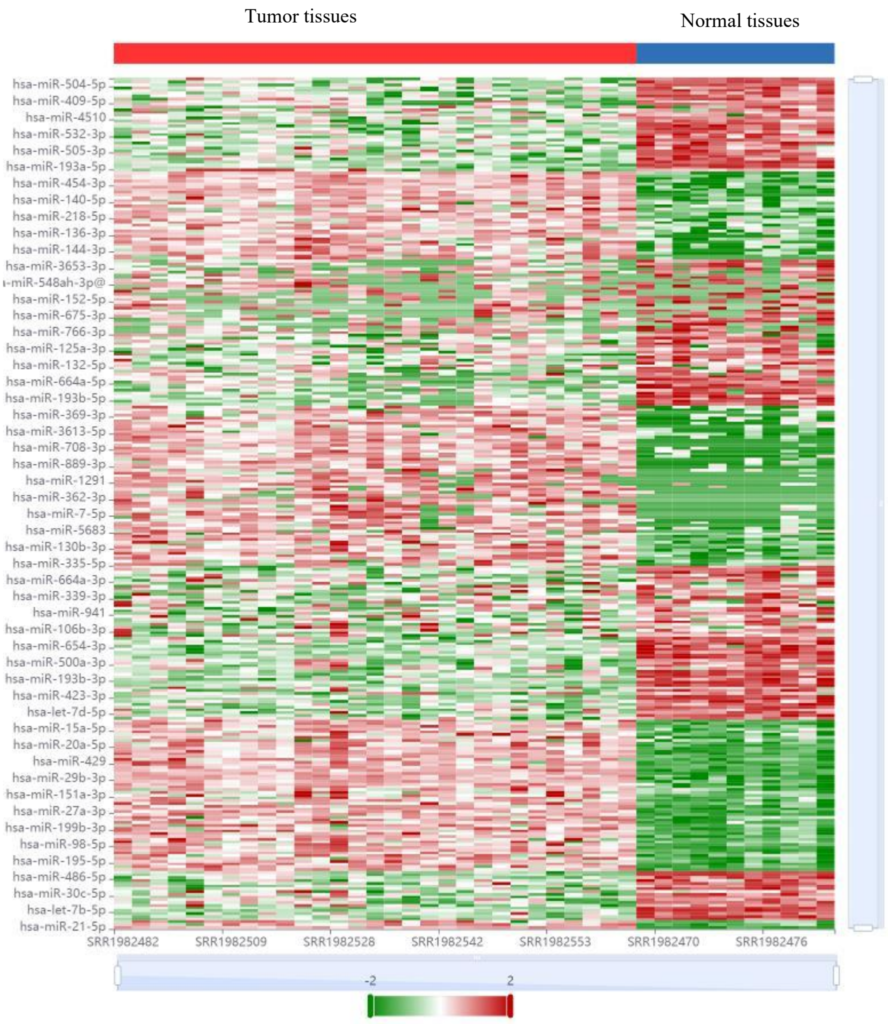

Figure S3. miR-374a expressed at a high level in basal-like breast cancer tissues compared to normal tissues in dbDEMC database.

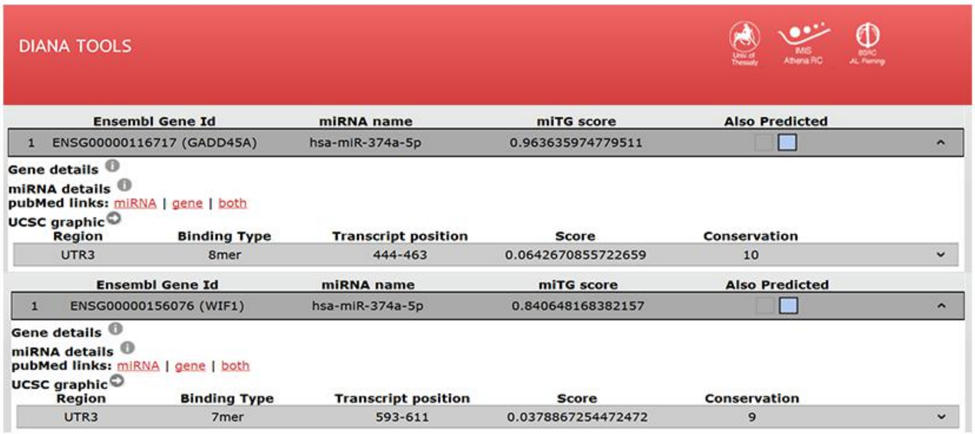

Figure S3. DIANA TOOLS (http://diana.imis.athena-innovation.gr/DianaTools/) predicted that miR-374a could bind to the 3'UTR region of GADD45A.
