# Supplementary figures and images for: Maackiain Modulates miR-374a/GADD45A Axis to Inhibit Triple-Negative Breast Cancer Initiation and Progression (part 2 of 2)
Source: Front Pharmacol. 2022 Mar 4;13:806869. doi: 10.3389/fphar.2022.806869 (PMC8930825; doi:10.3389/fphar.2022.806869)

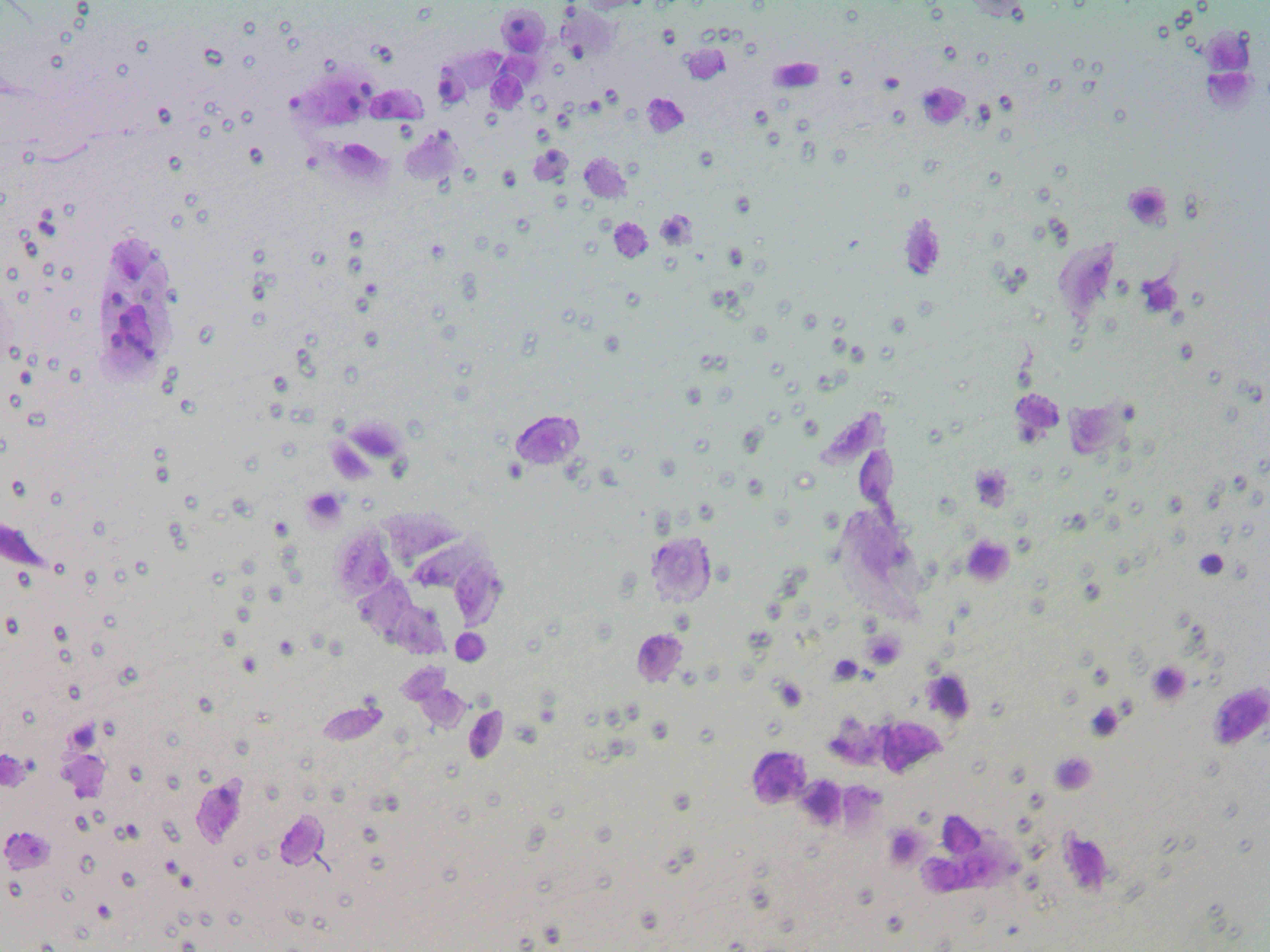

Supplement: Supplementary file 7 [file DataSheet5.ZIP › BT-549/6-1-100X (3)-1_new.jpg]

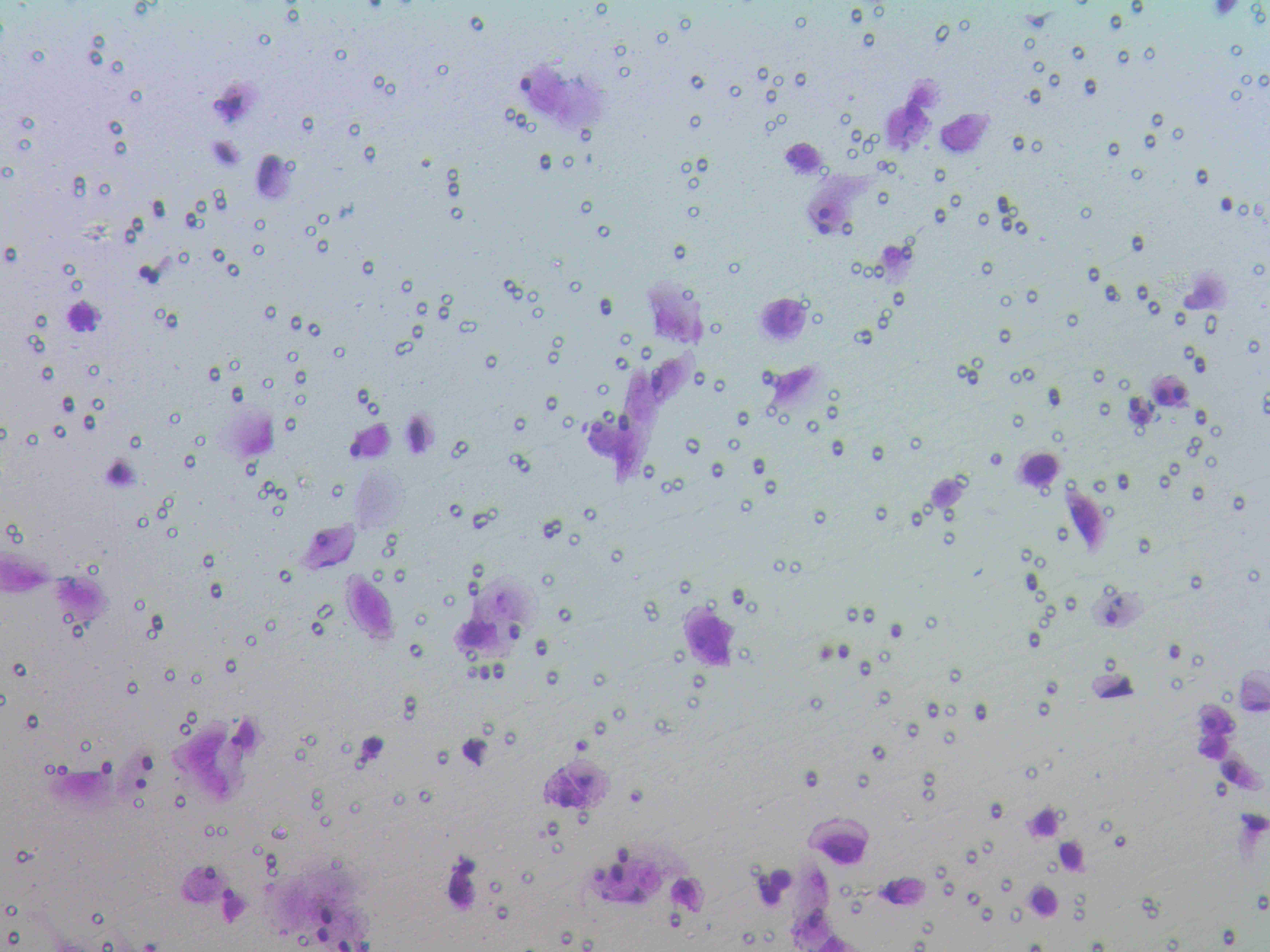

Supplement: Supplementary file 7 [file DataSheet5.ZIP › BT-549/6-2-100X (4)-1_new.jpg]

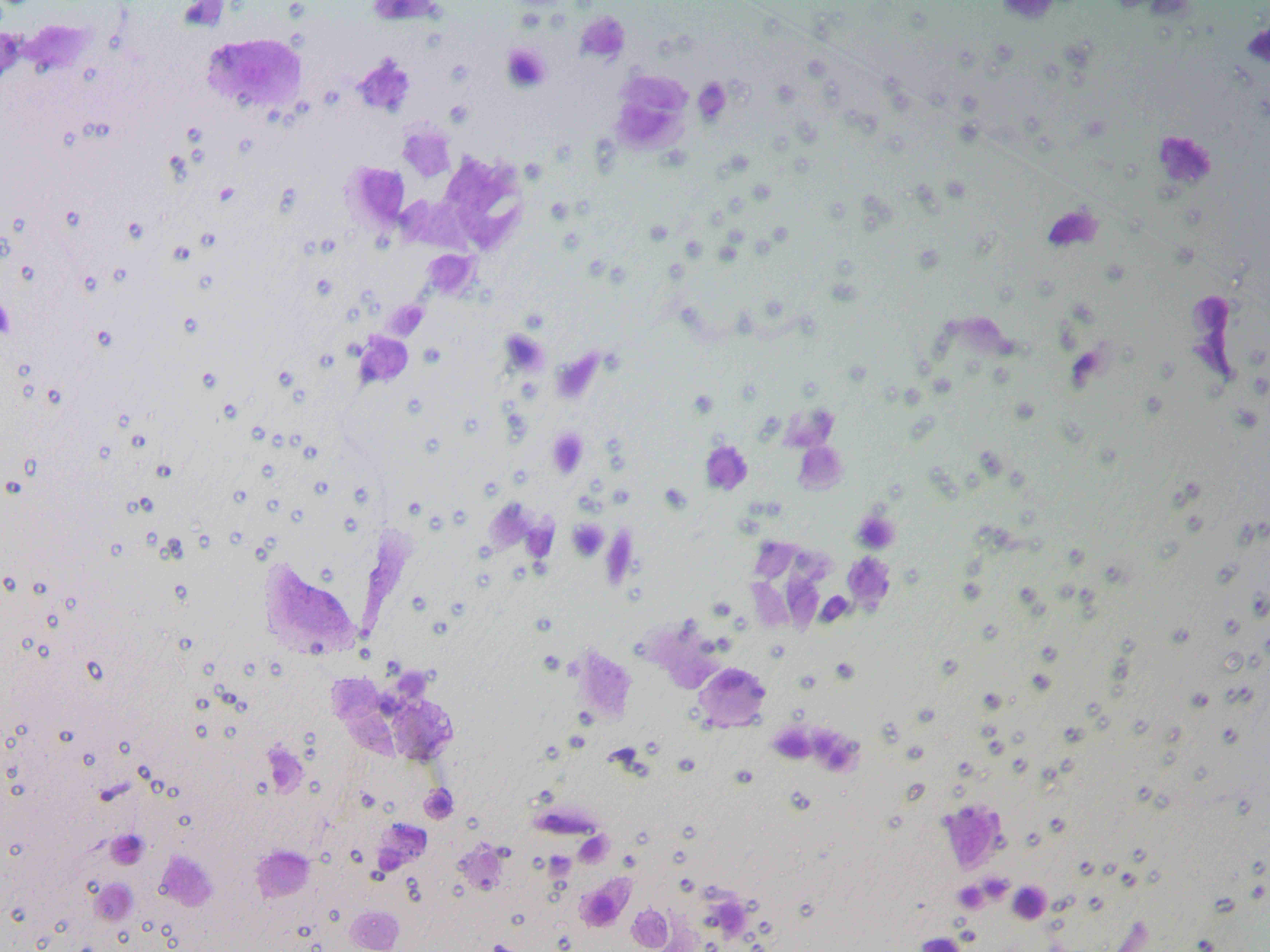

Supplement: Supplementary file 7 [file DataSheet5.ZIP › BT-549/6-3-100X (5)-1_new.jpg]

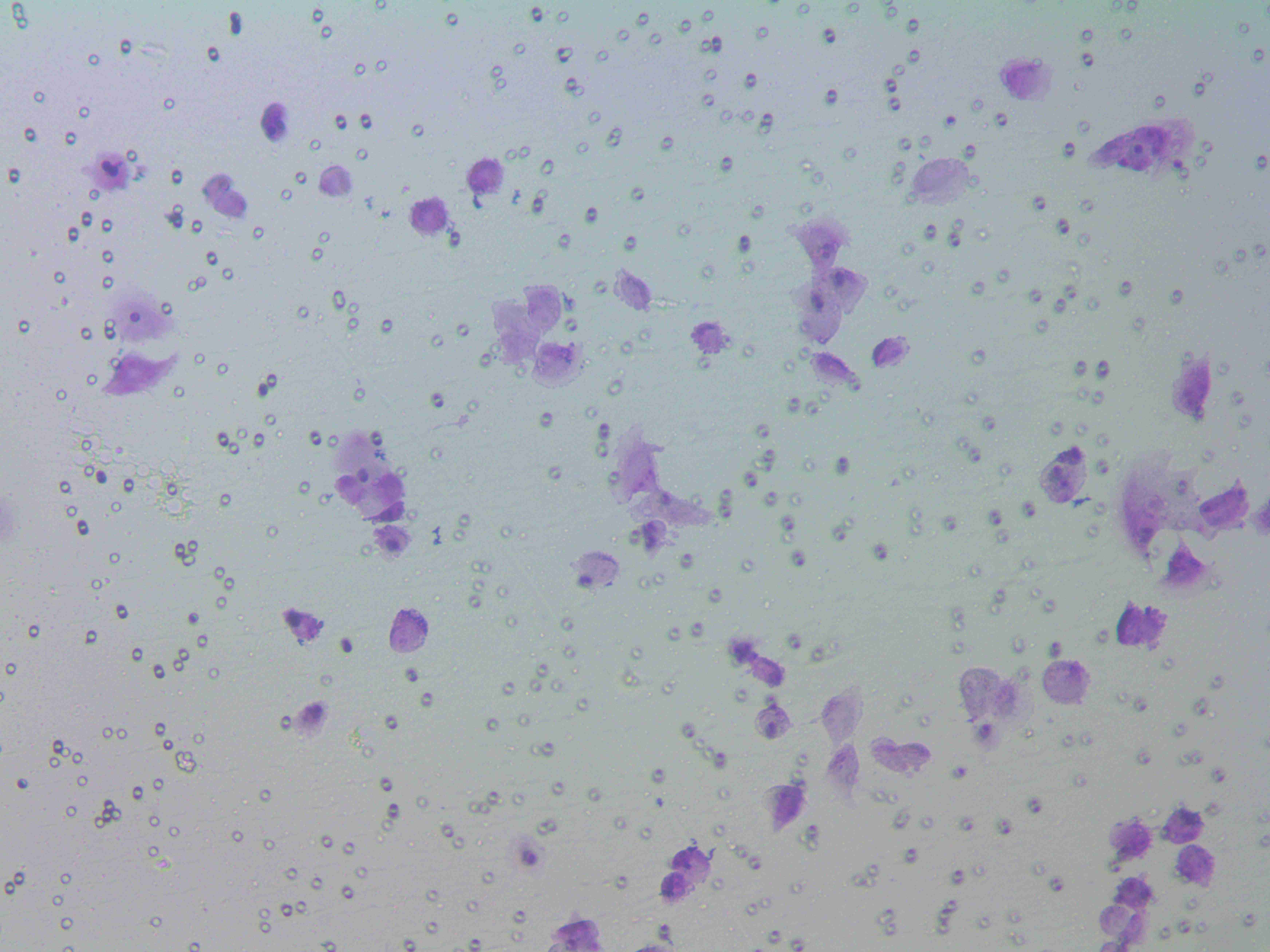

Supplement: Supplementary file 7 [file DataSheet5.ZIP › BT-549/7-1-100X (4)-1_new.jpg]

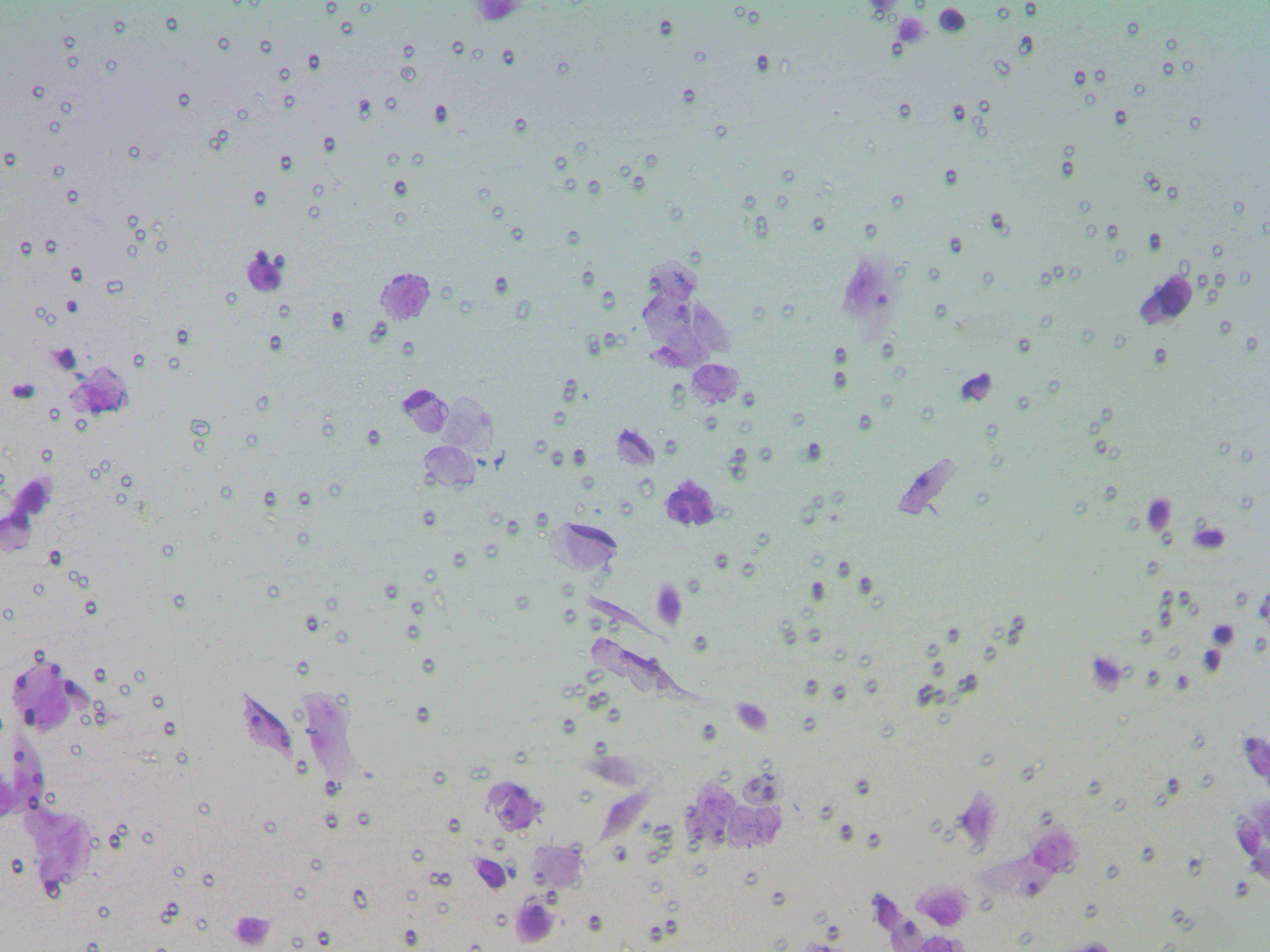

Supplement: Supplementary file 7 [file DataSheet5.ZIP › BT-549/7-2-100X (4)-1_new.jpg]

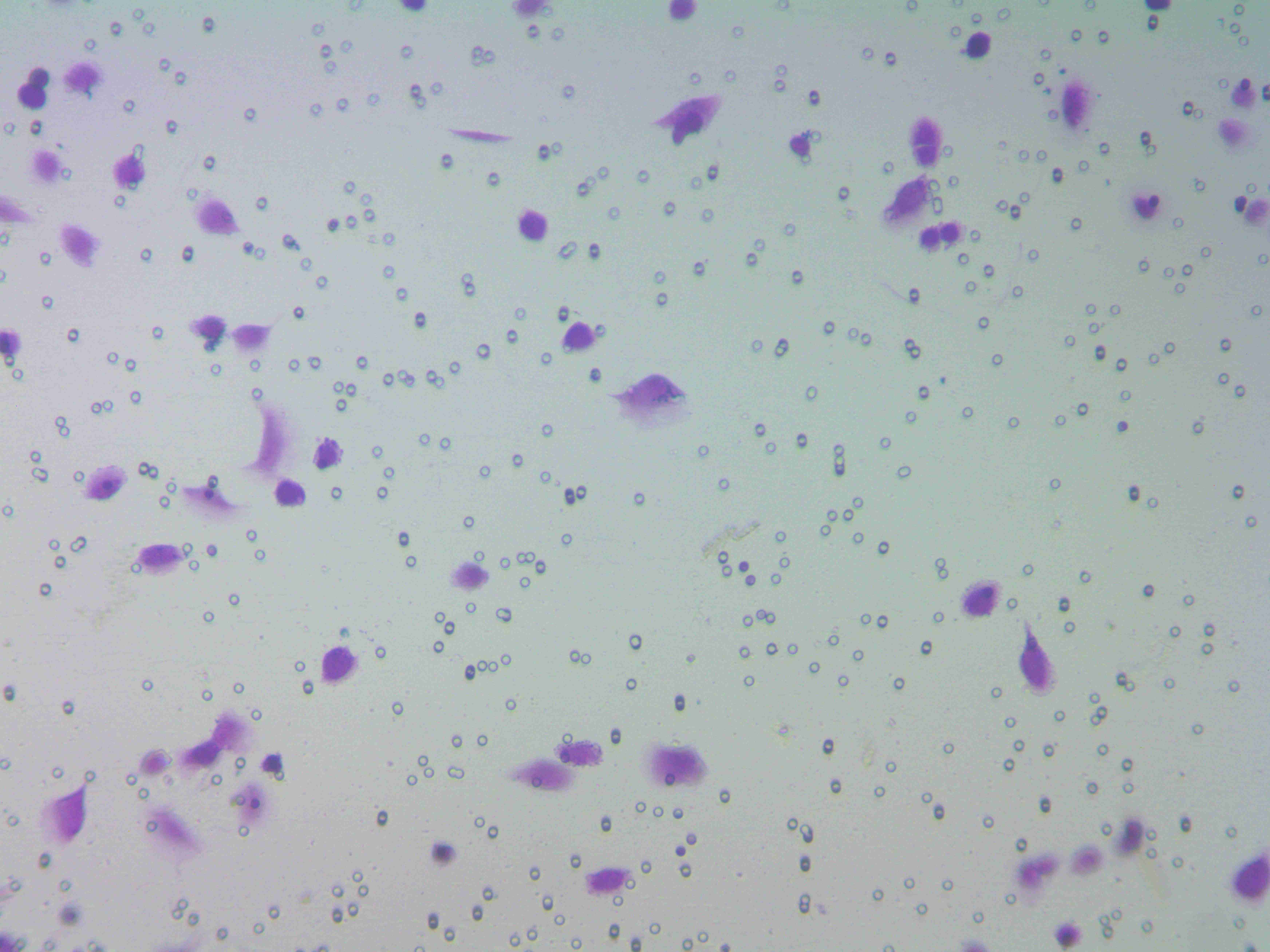

Supplement: Supplementary file 7 [file DataSheet5.ZIP › BT-549/7-3-100X (2)-1_new.jpg]

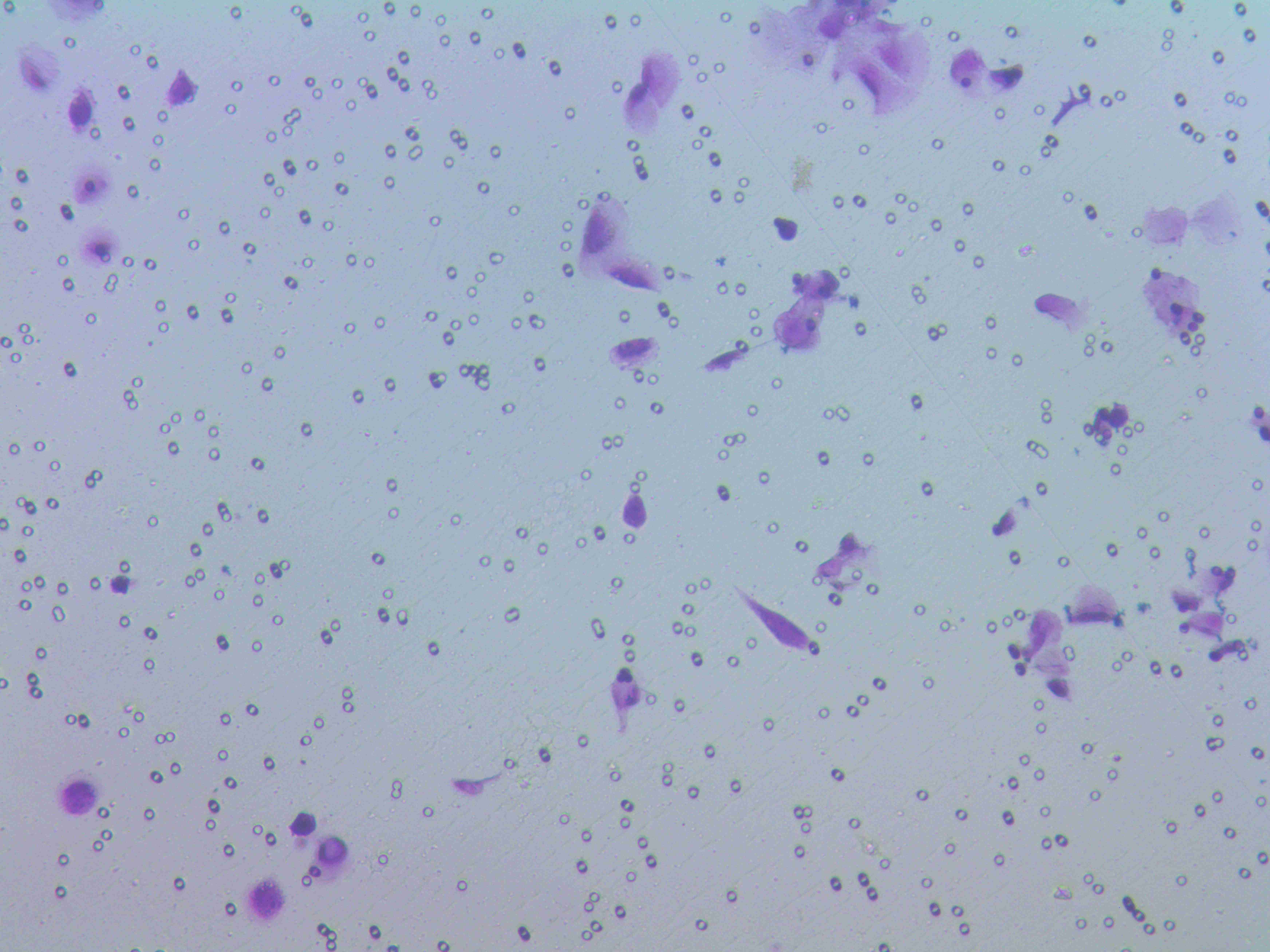

Supplement: Supplementary file 7 [file DataSheet5.ZIP › BT-549/8-1-100X (5)-1_new.jpg]

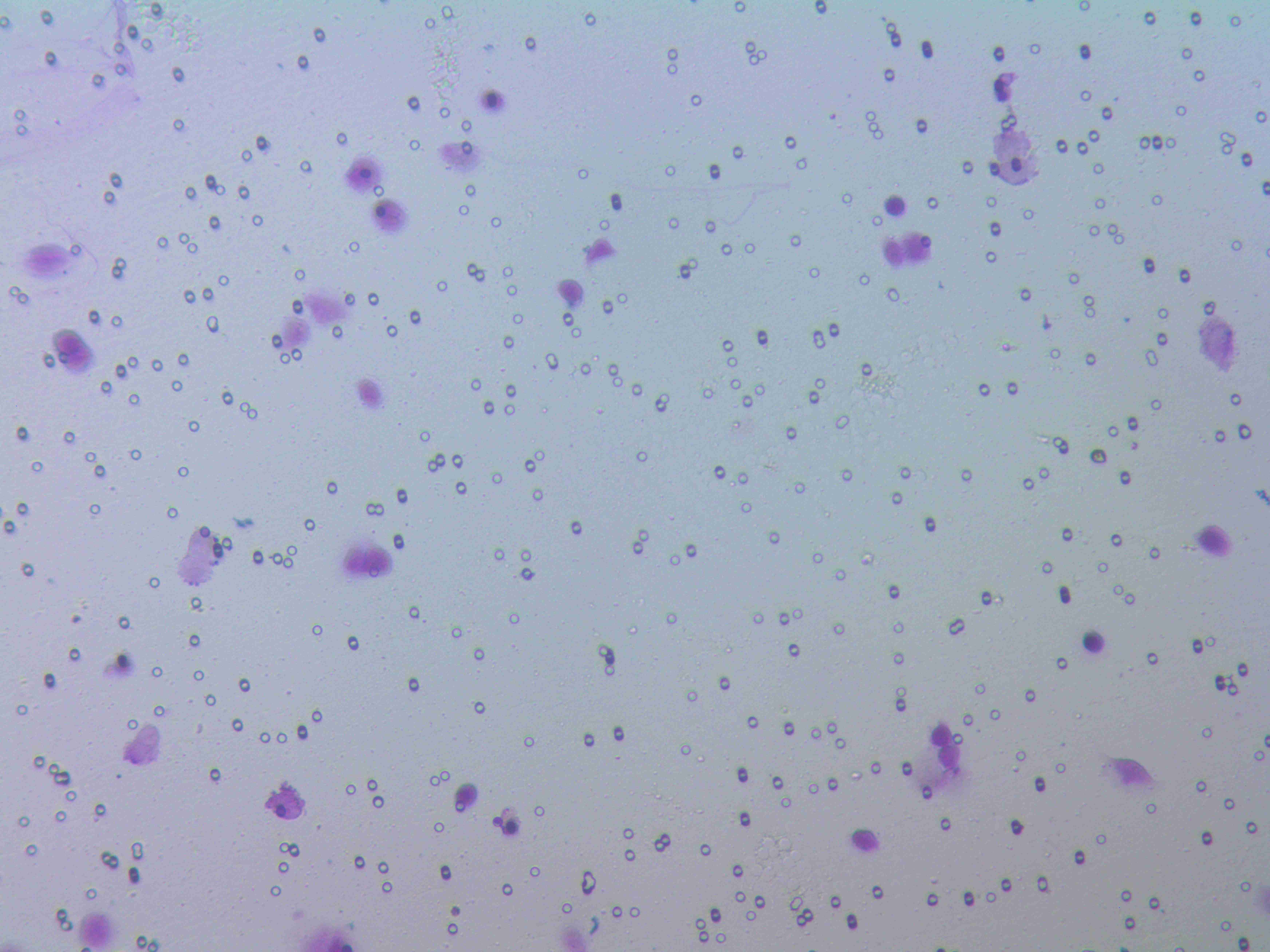

Supplement: Supplementary file 7 [file DataSheet5.ZIP › BT-549/8-2-100X (3)-1_new.jpg]

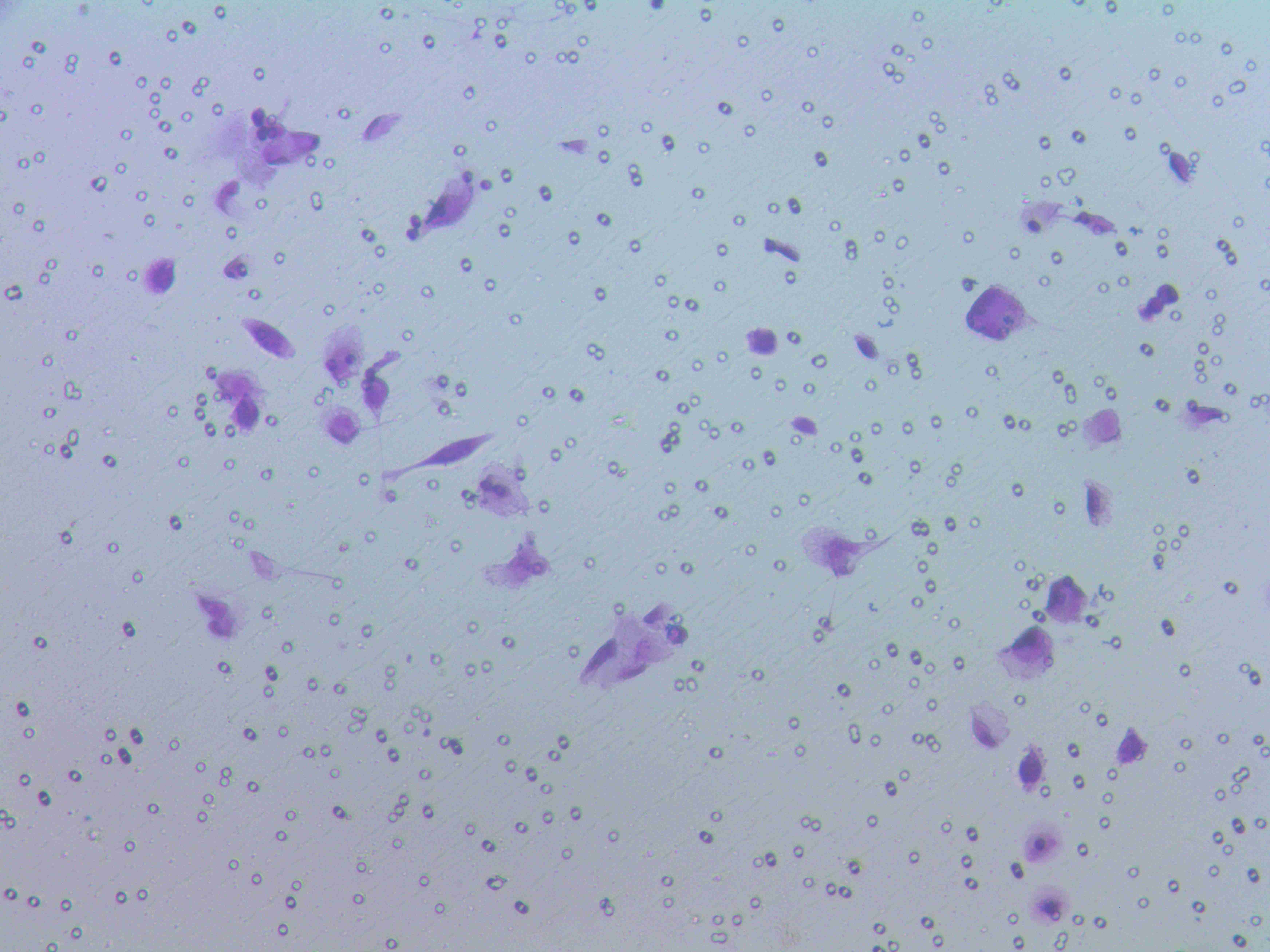

Supplement: Supplementary file 7 [file DataSheet5.ZIP › BT-549/8-2-100X (4)-1_new.jpg]

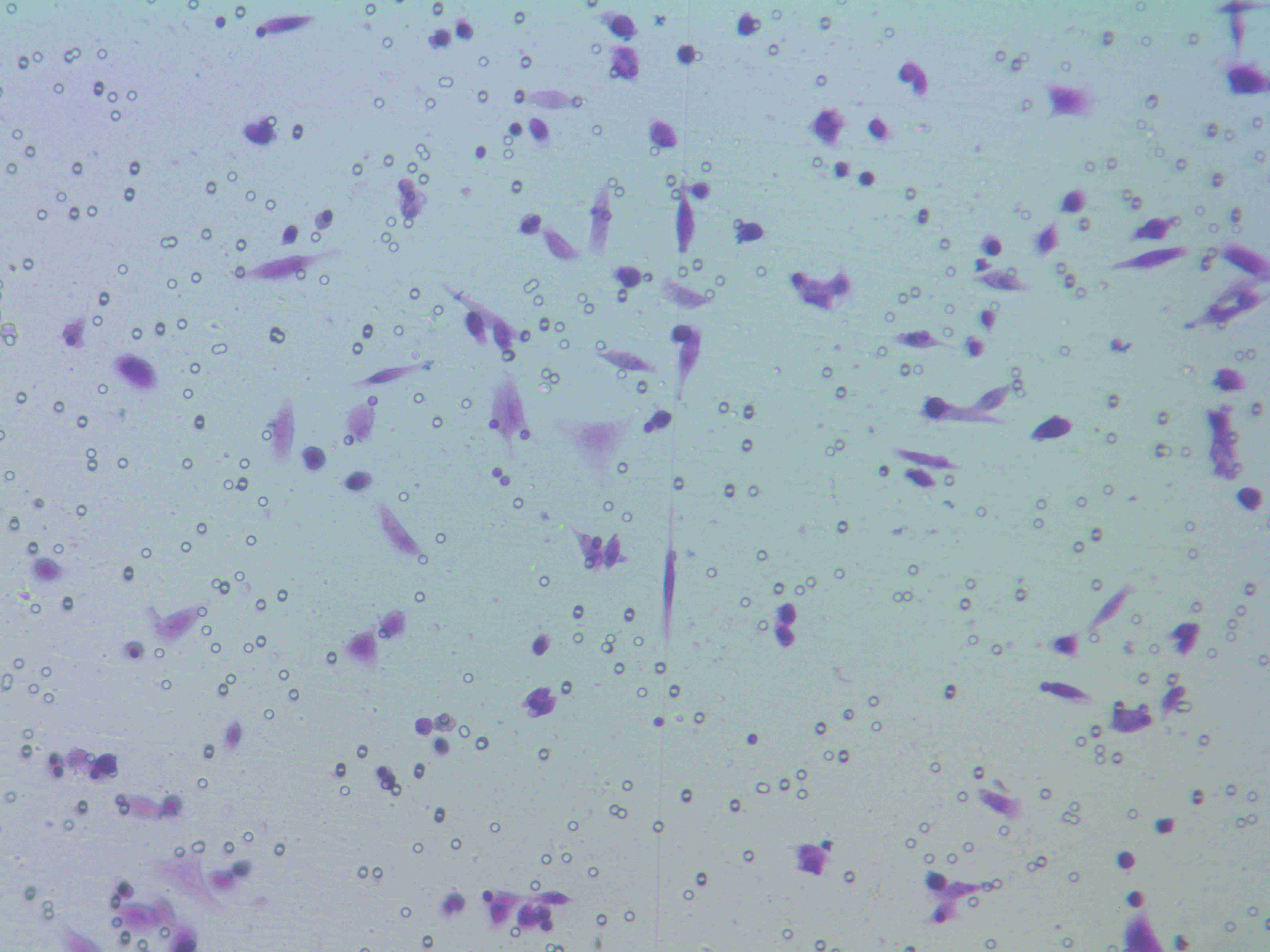

Supplement: Supplementary file 7 [file DataSheet5.ZIP › MDA-MB-231/1-1-100X (3)-1_new.jpg]

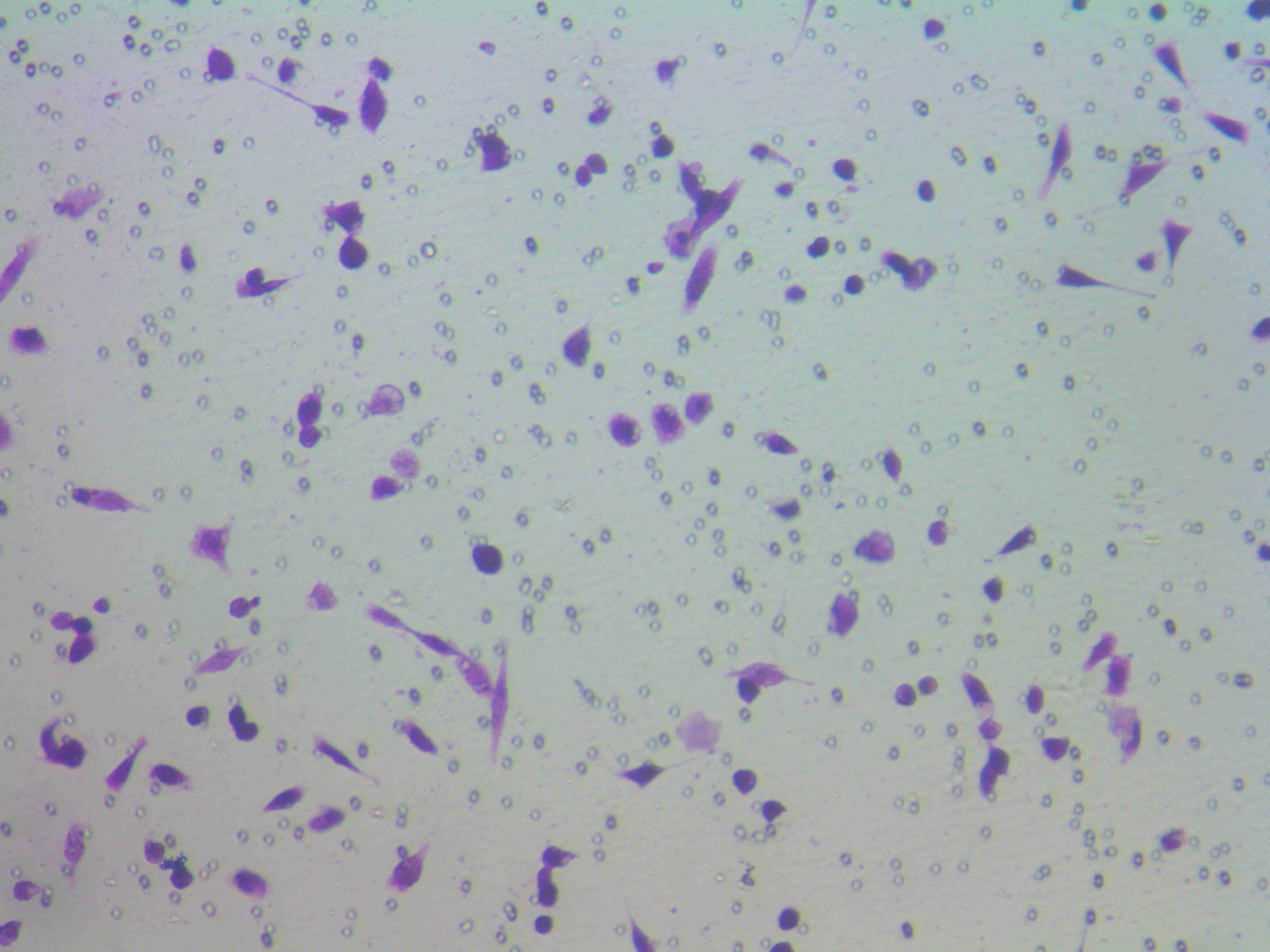

Supplement: Supplementary file 7 [file DataSheet5.ZIP › MDA-MB-231/1-2-100X (3)-1_new.jpg]

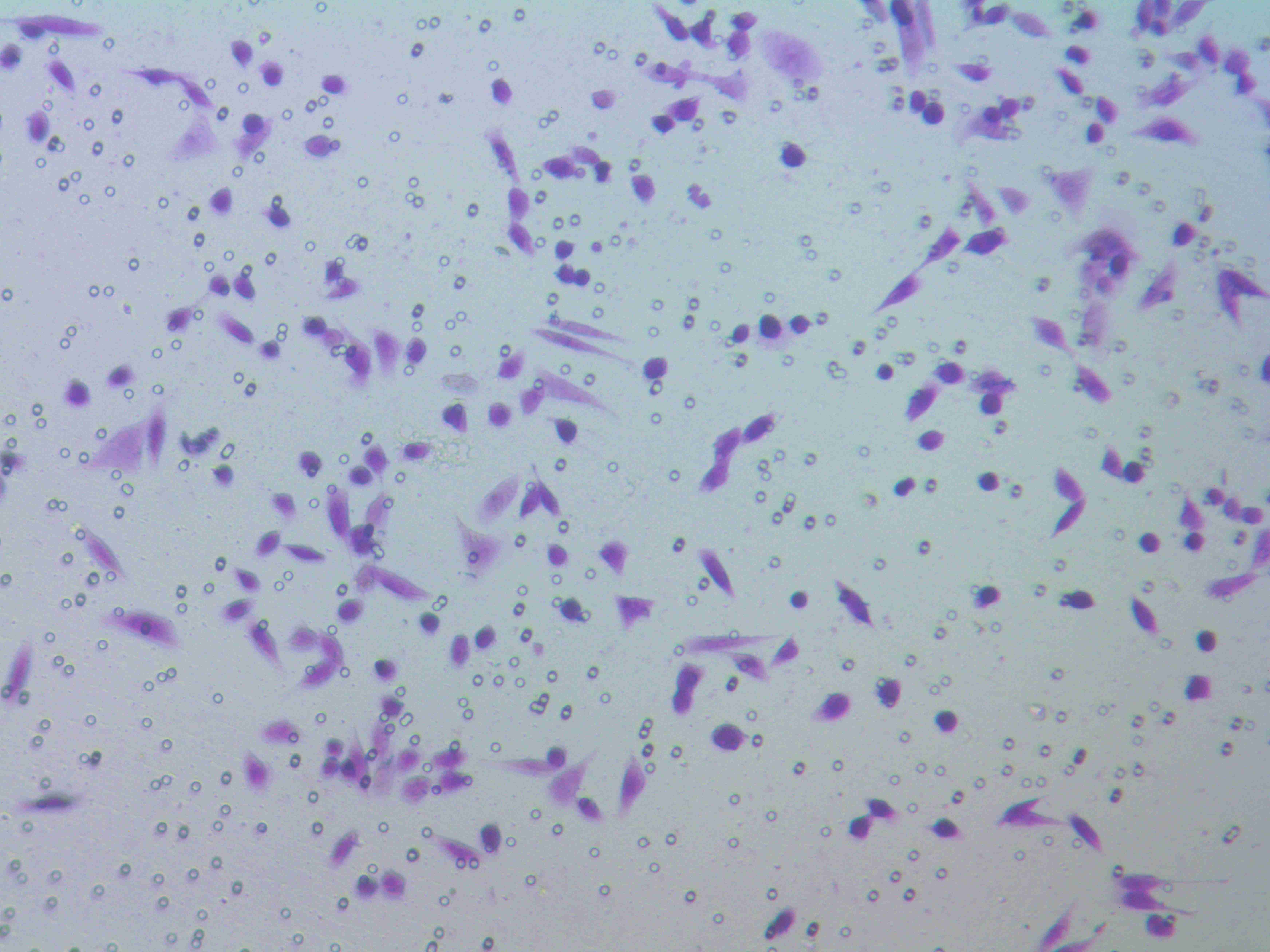

Supplement: Supplementary file 7 [file DataSheet5.ZIP › MDA-MB-231/1-3-100X (1)-1_new.jpg]

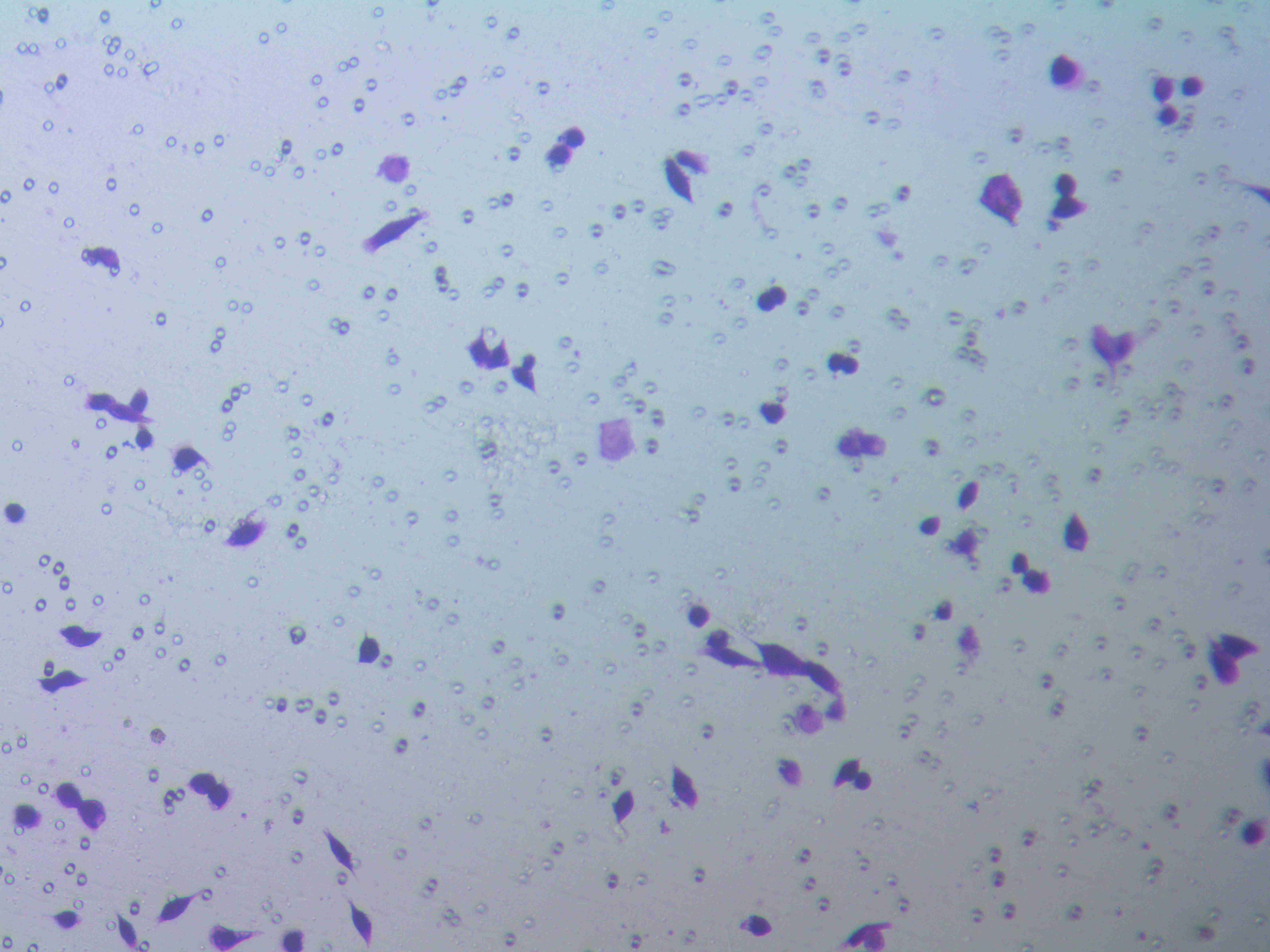

Supplement: Supplementary file 7 [file DataSheet5.ZIP › MDA-MB-231/2-1-100X (3)-1_new.jpg]

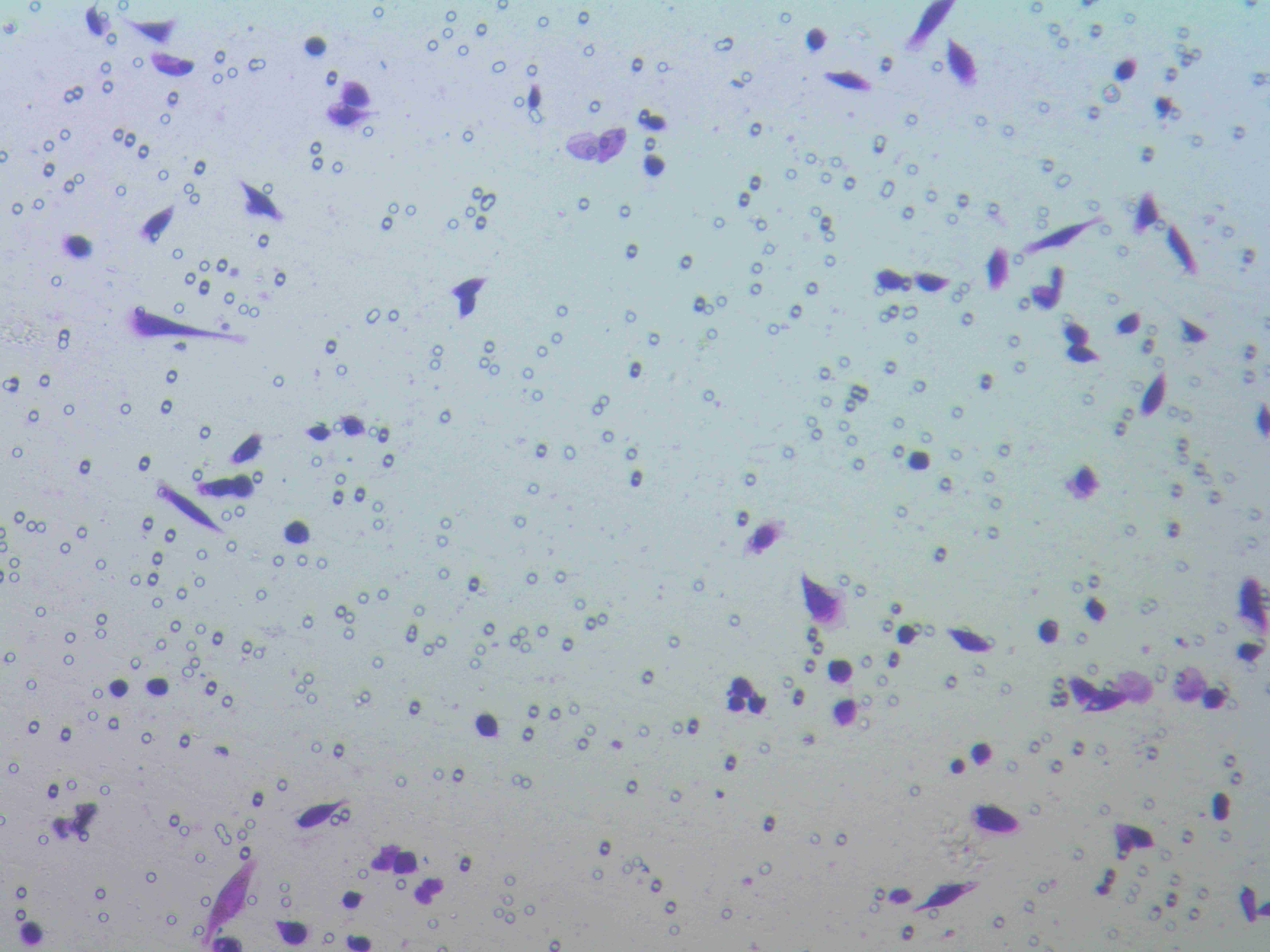

Supplement: Supplementary file 7 [file DataSheet5.ZIP › MDA-MB-231/2-2-100X (2)-1_new.jpg]

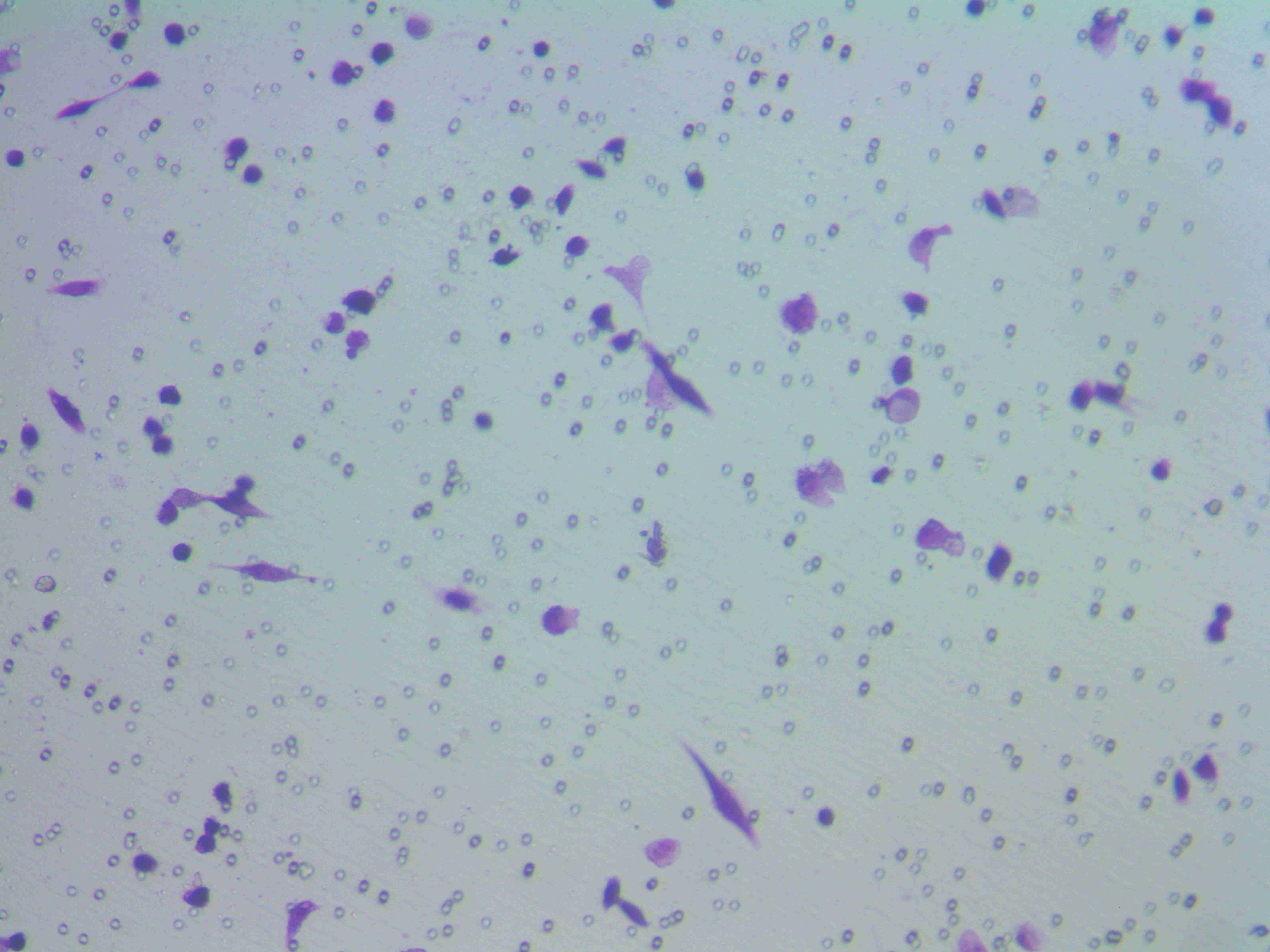

Supplement: Supplementary file 7 [file DataSheet5.ZIP › MDA-MB-231/2-3-100X (2)-1_new.jpg]

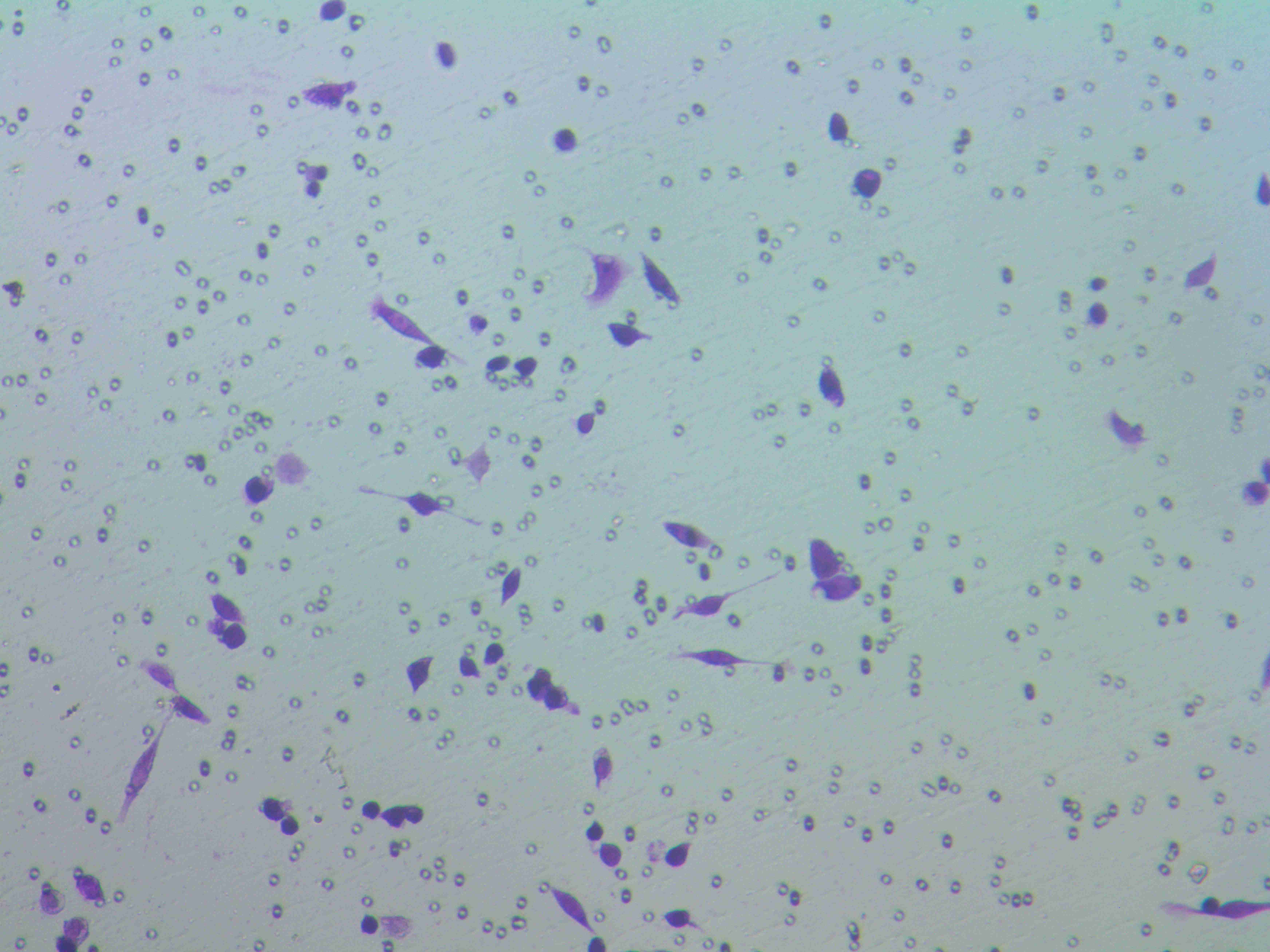

Supplement: Supplementary file 7 [file DataSheet5.ZIP › MDA-MB-231/3-3-100X (1)-1_new.jpg]

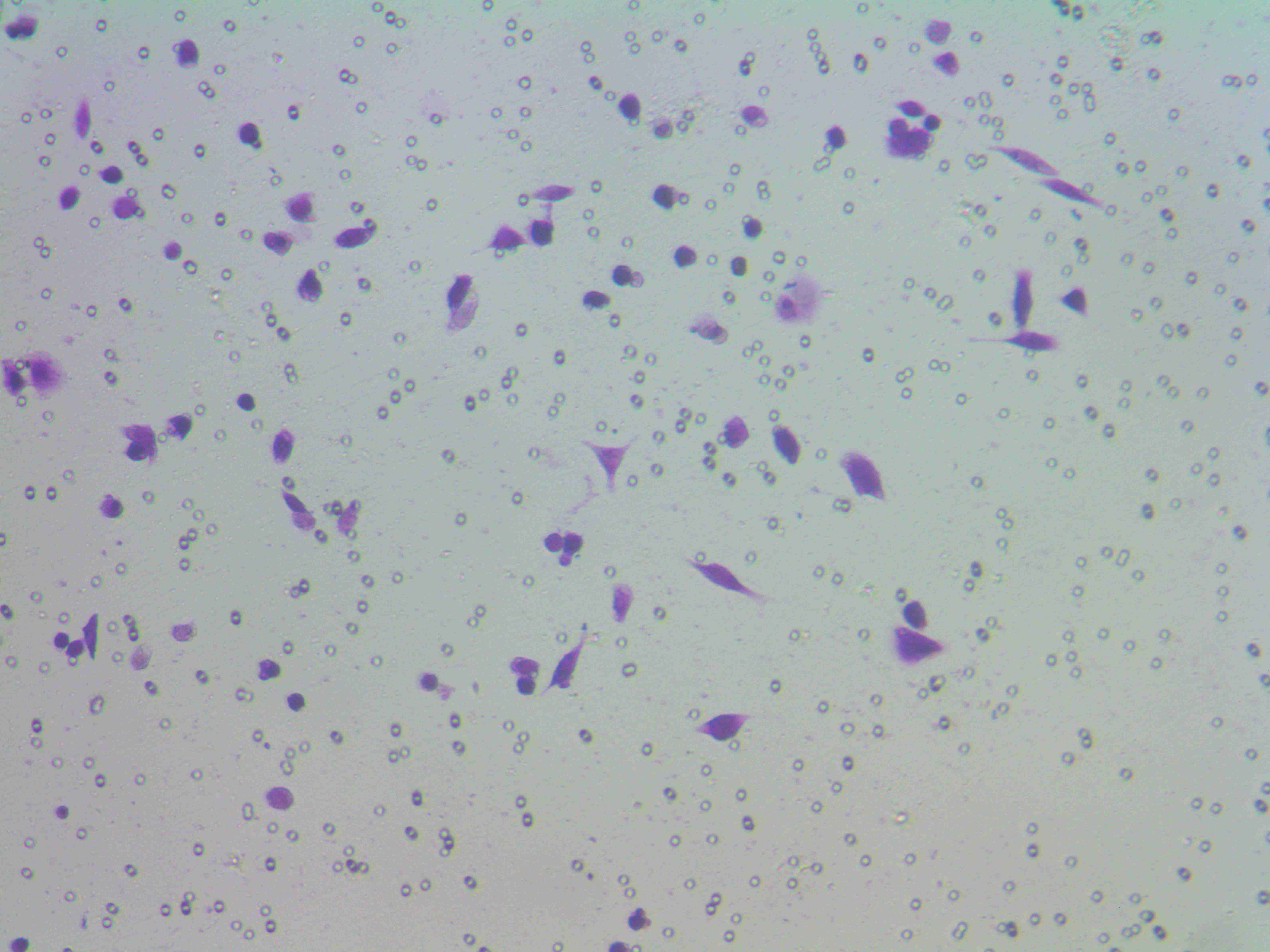

Supplement: Supplementary file 7 [file DataSheet5.ZIP › MDA-MB-231/3-3-100X (3)-1_new.jpg]

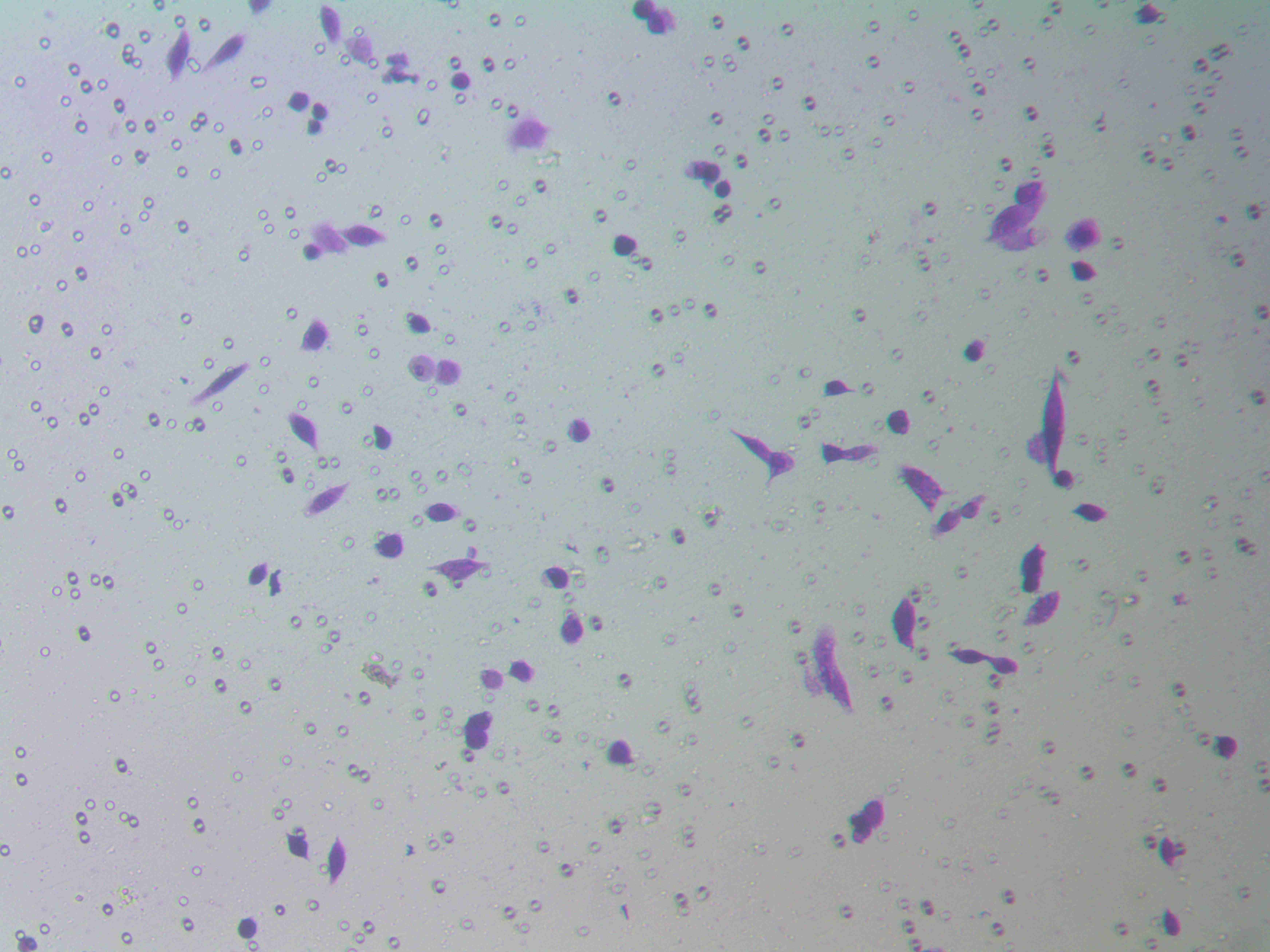

Supplement: Supplementary file 7 [file DataSheet5.ZIP › MDA-MB-231/3-3-100X (4)-1_new.jpg]

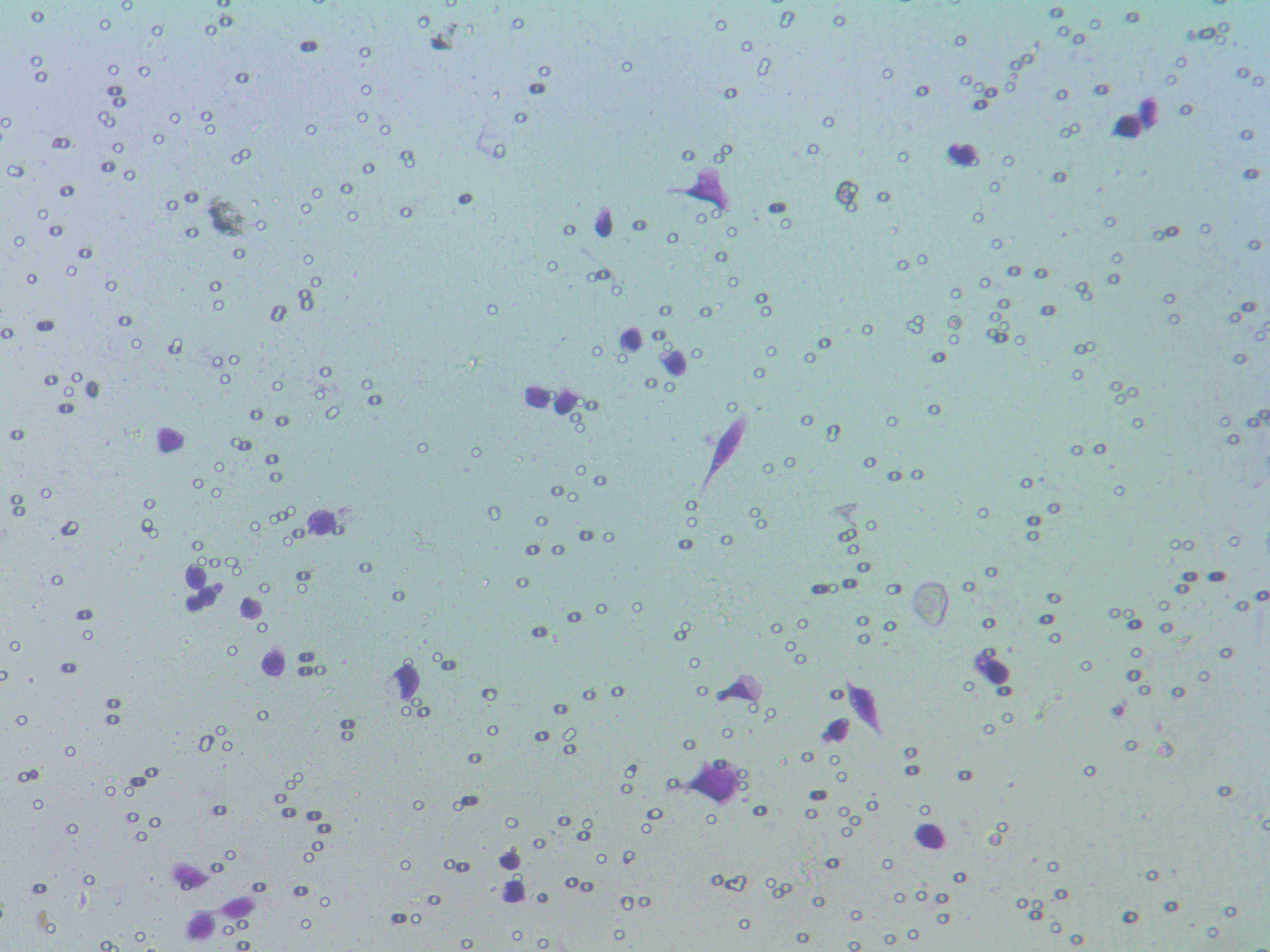

Supplement: Supplementary file 7 [file DataSheet5.ZIP › MDA-MB-231/4-2-100X (3)-1_new.jpg]

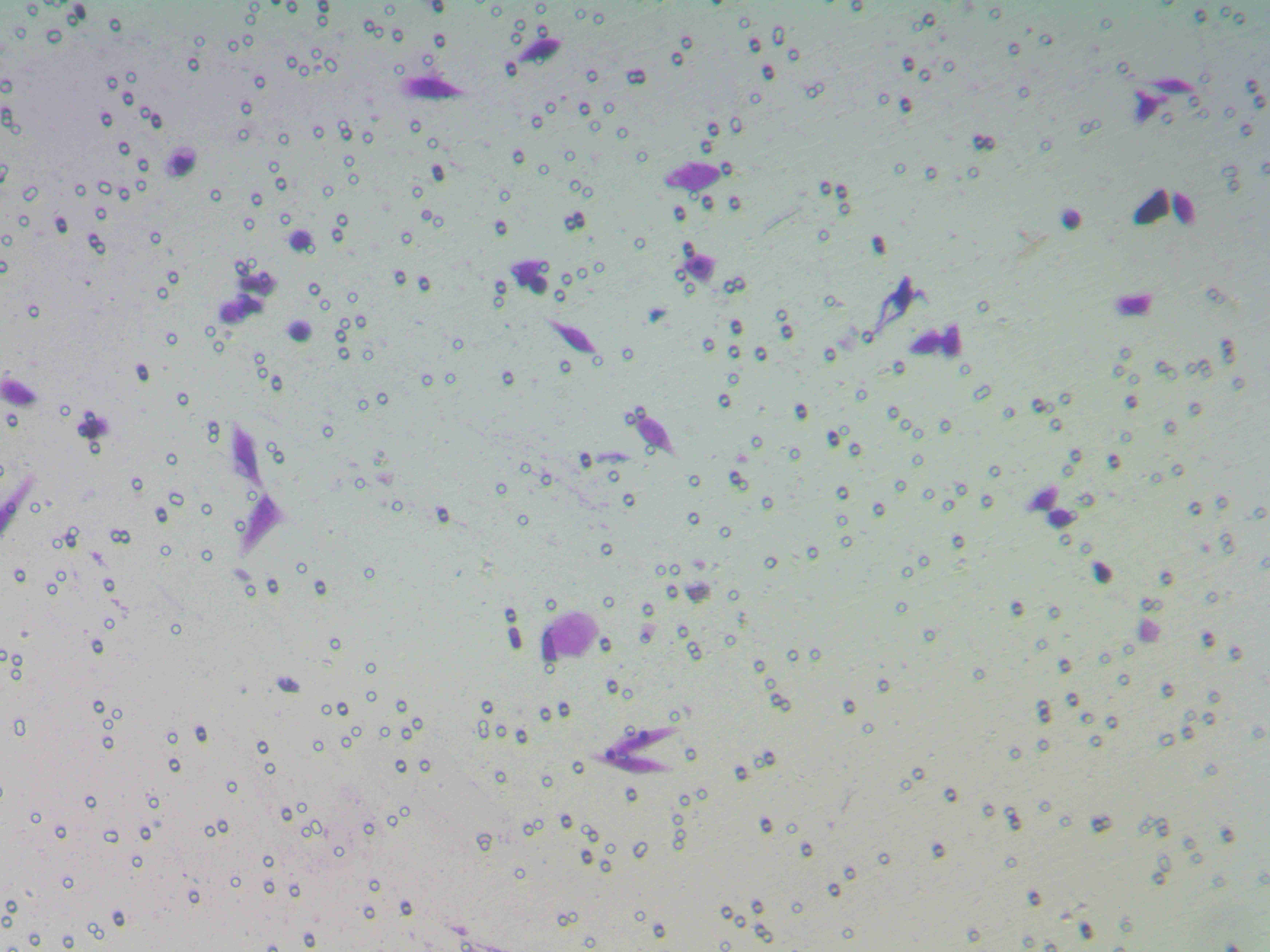

Supplement: Supplementary file 7 [file DataSheet5.ZIP › MDA-MB-231/4-3-100X (2)-1_new.jpg]

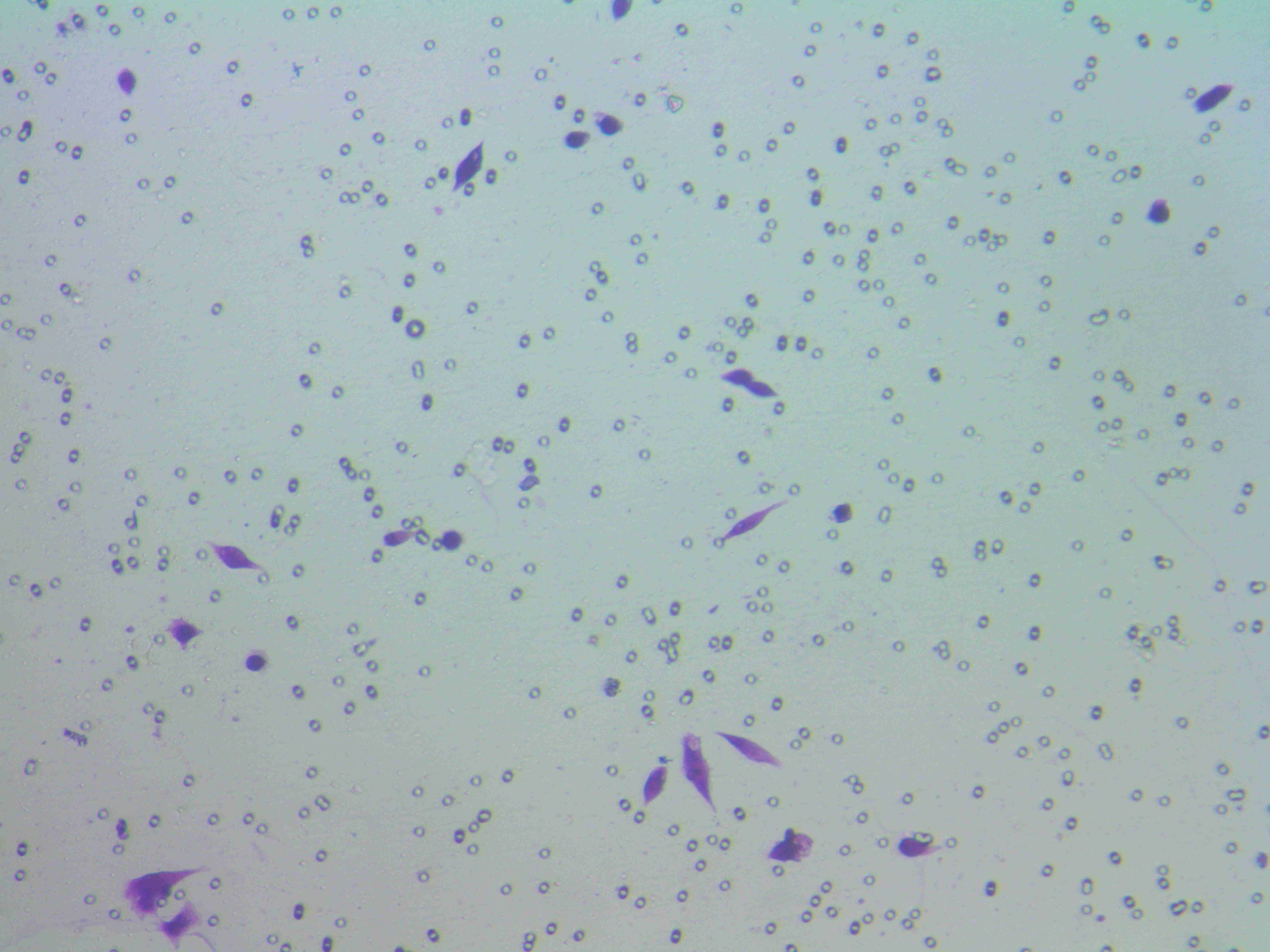

Supplement: Supplementary file 7 [file DataSheet5.ZIP › MDA-MB-231/4-3-100X (5)-1_new.jpg]

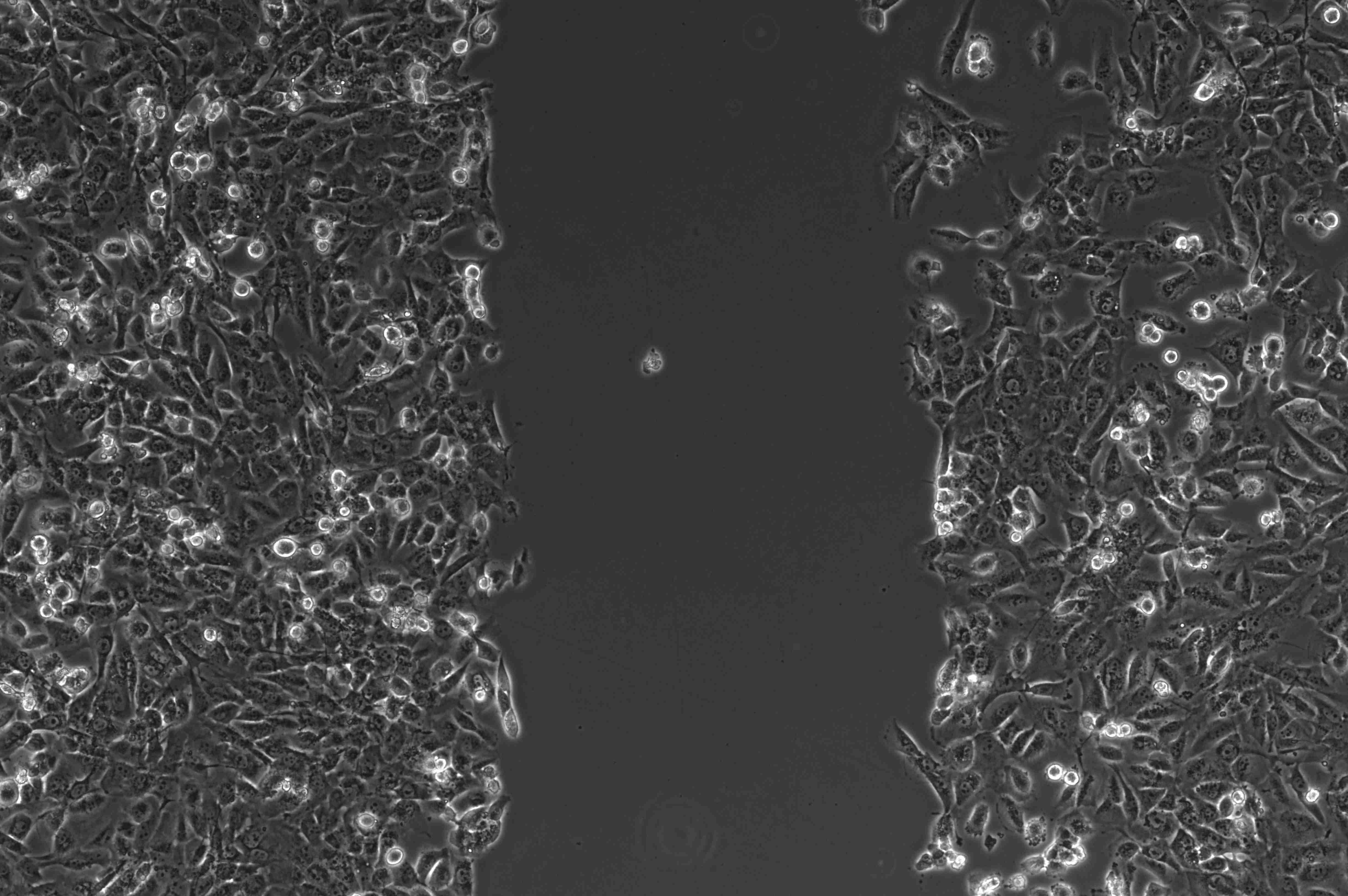

Supplement: Supplementary file 8 [file DataSheet7.ZIP › Wound healing/BT-549/5-0h-100X (1)-1_new.jpg]

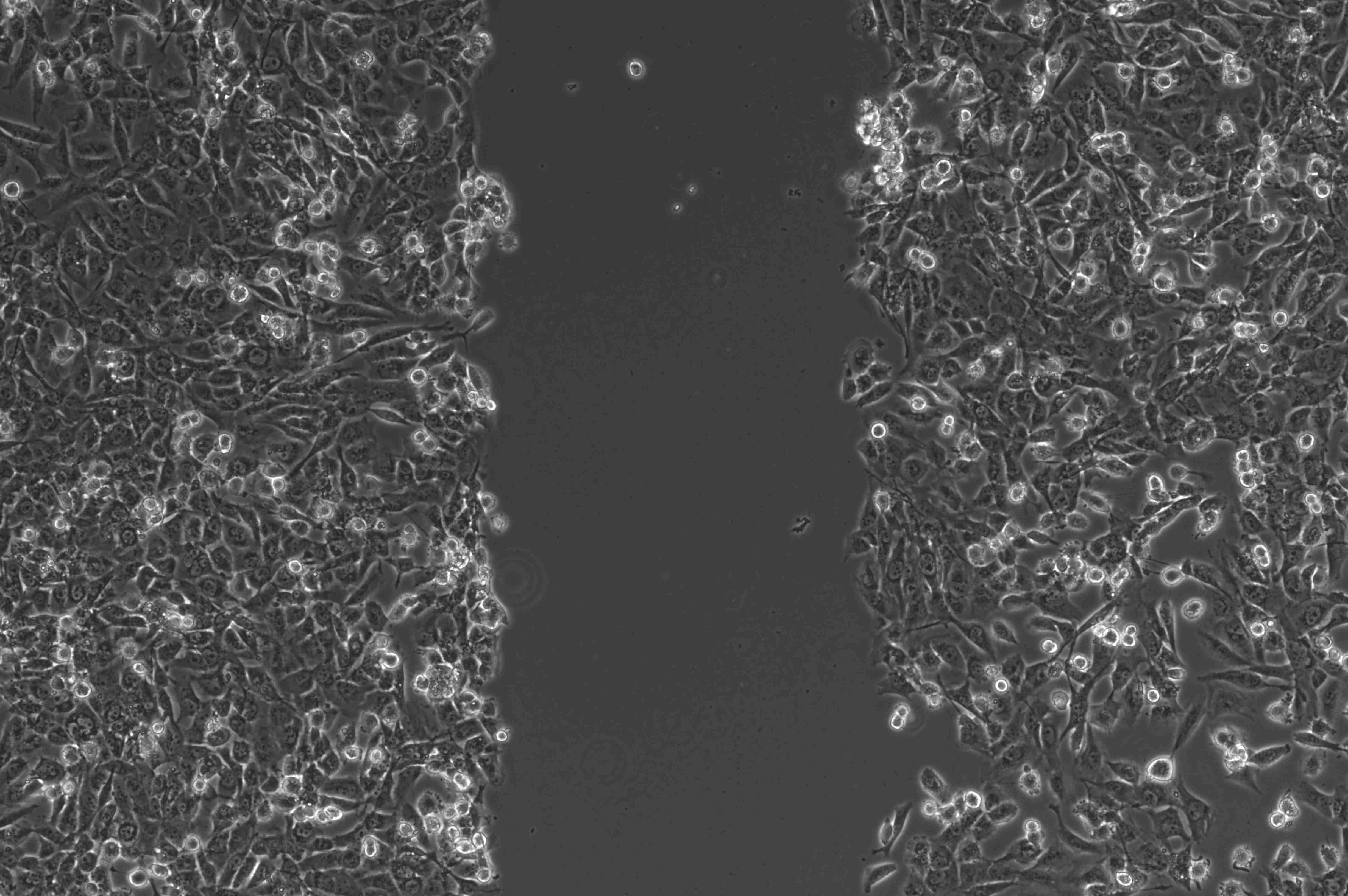

Supplement: Supplementary file 8 [file DataSheet7.ZIP › Wound healing/BT-549/5-0h-100X (2)_new.jpg]

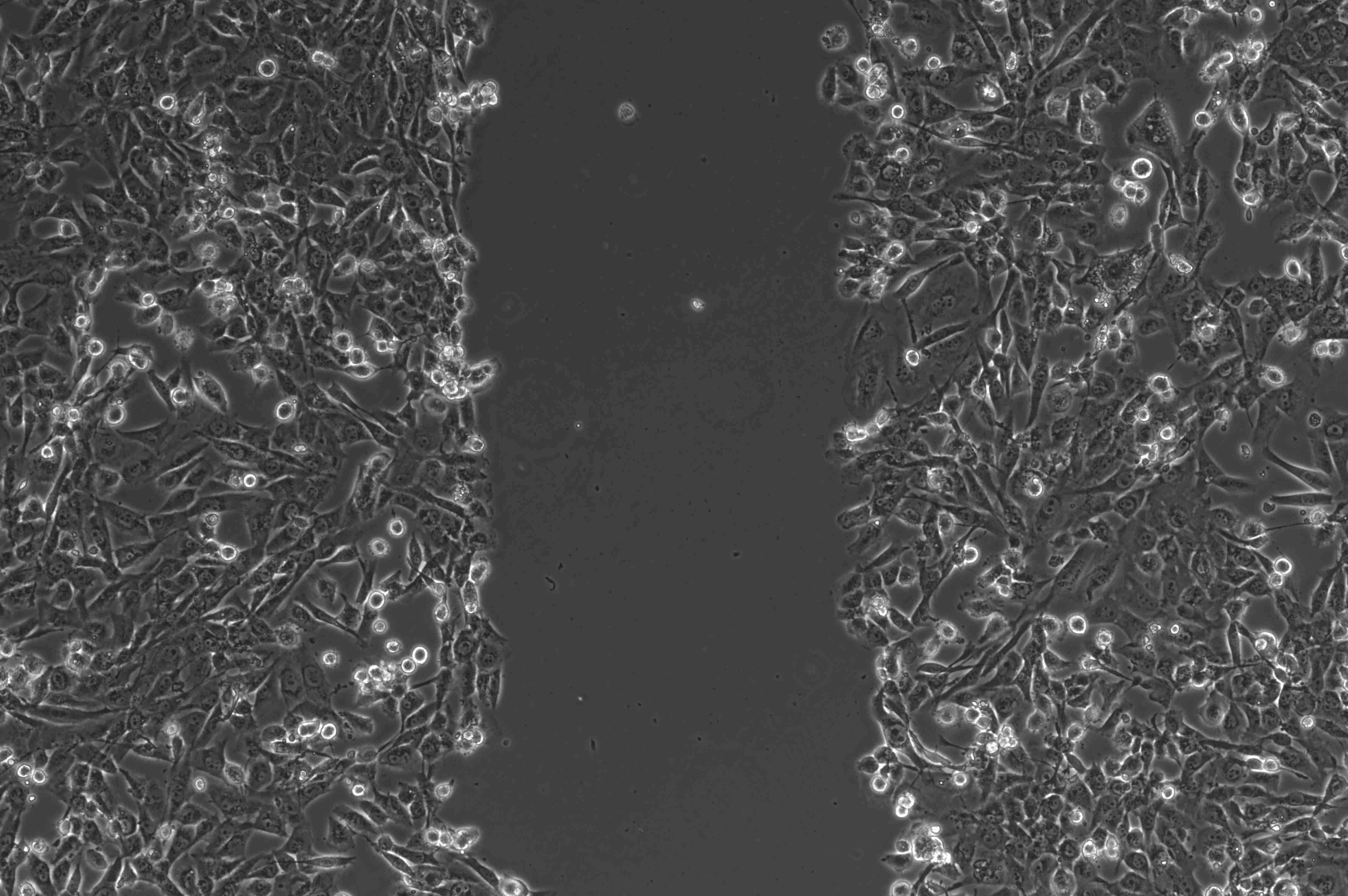

Supplement: Supplementary file 8 [file DataSheet7.ZIP › Wound healing/BT-549/5-0h-100X (3)_new.jpg]

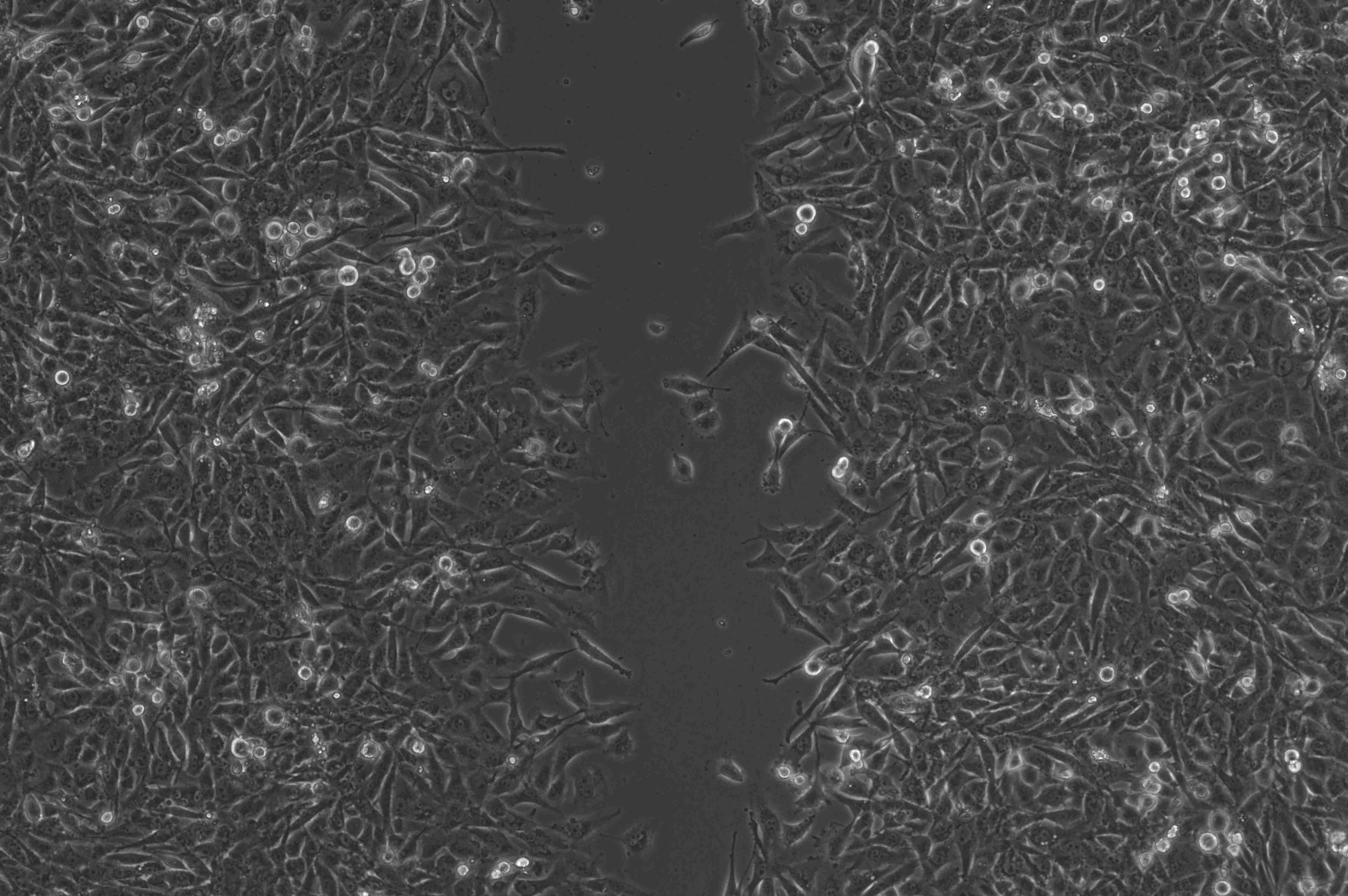

Supplement: Supplementary file 8 [file DataSheet7.ZIP › Wound healing/BT-549/5-24h-100X (1)_new.jpg]

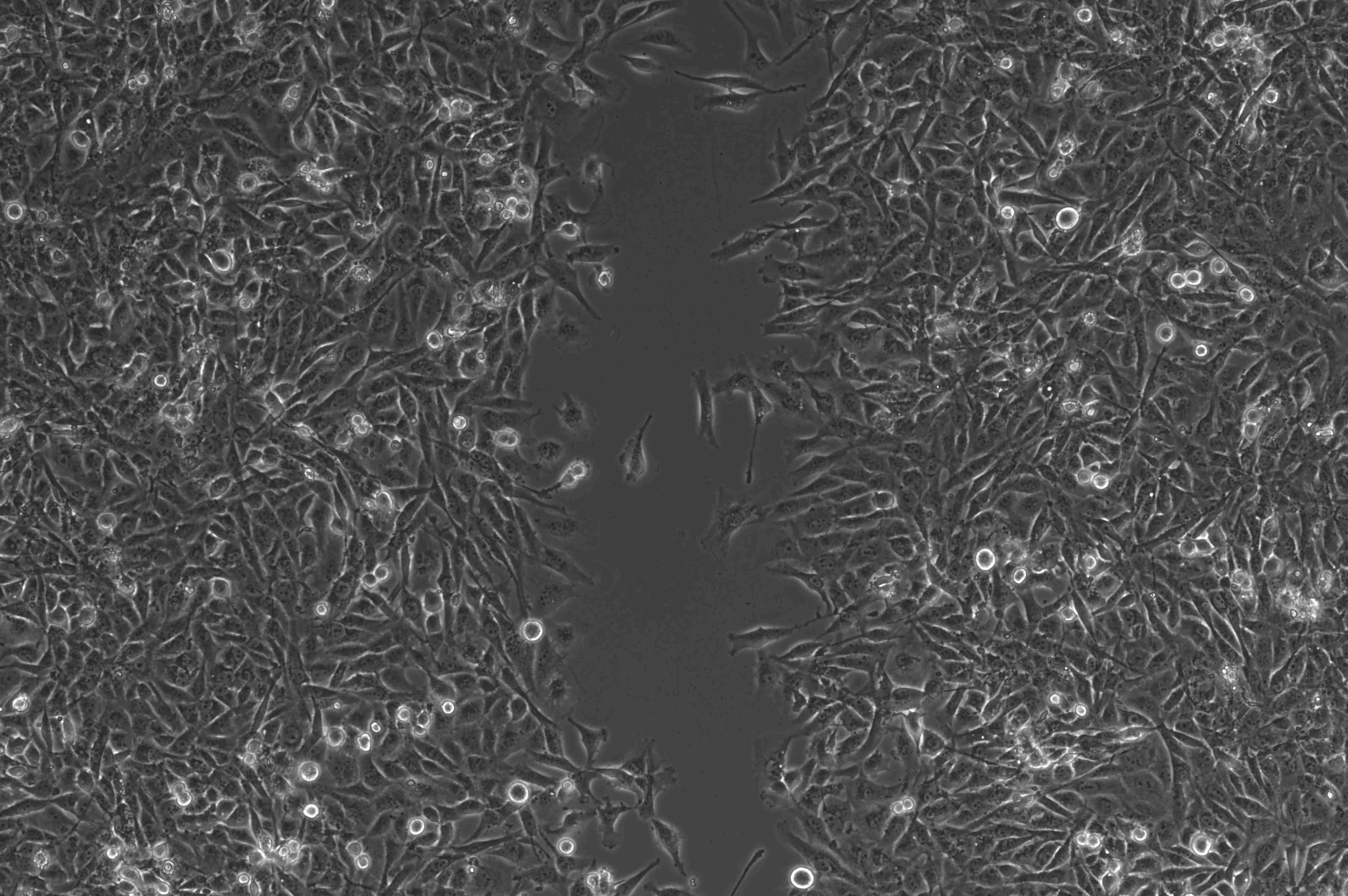

Supplement: Supplementary file 8 [file DataSheet7.ZIP › Wound healing/BT-549/5-24h-100X (2)-1_new.jpg]

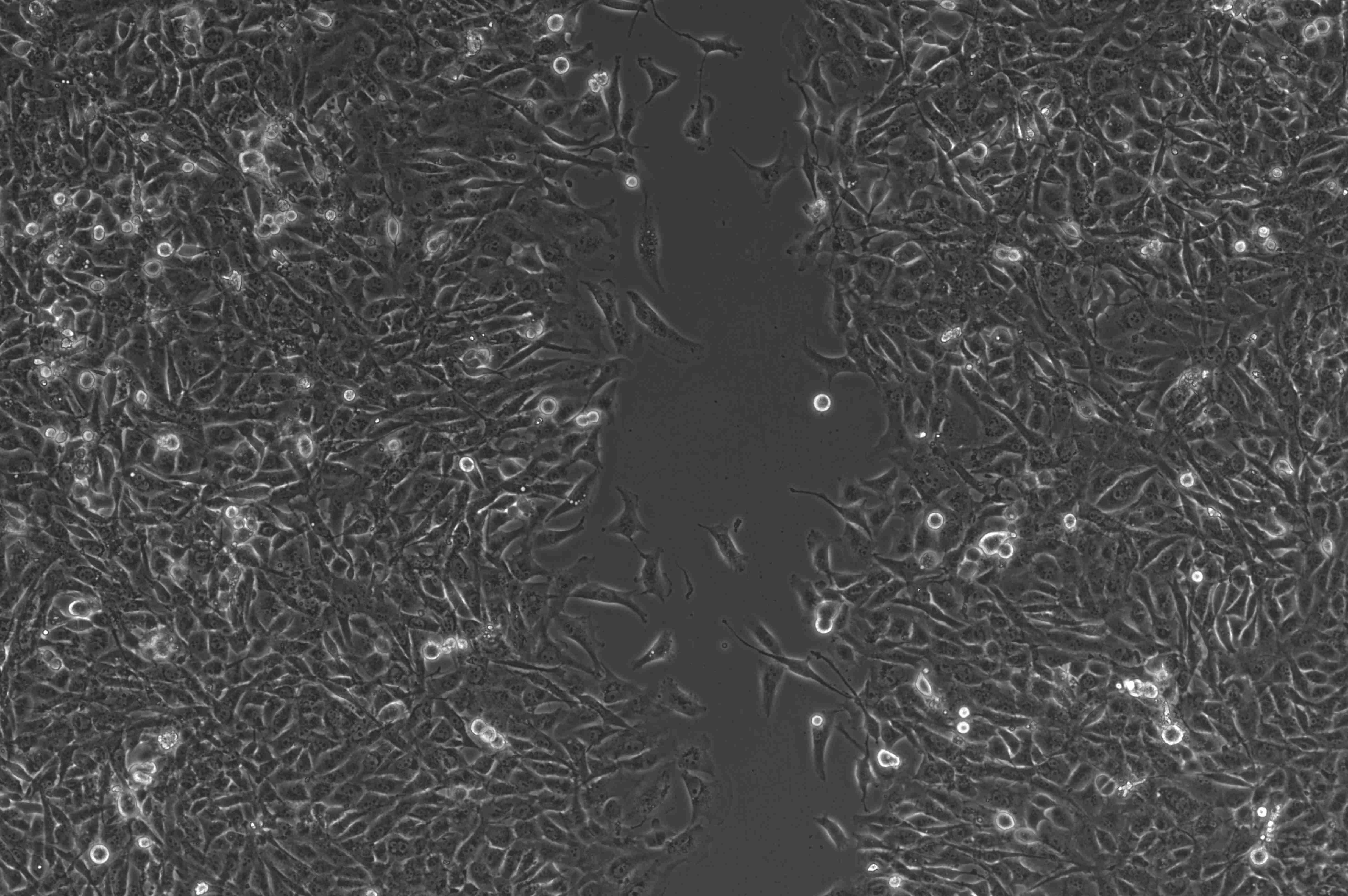

Supplement: Supplementary file 8 [file DataSheet7.ZIP › Wound healing/BT-549/5-24h-100X (3)_new.jpg]

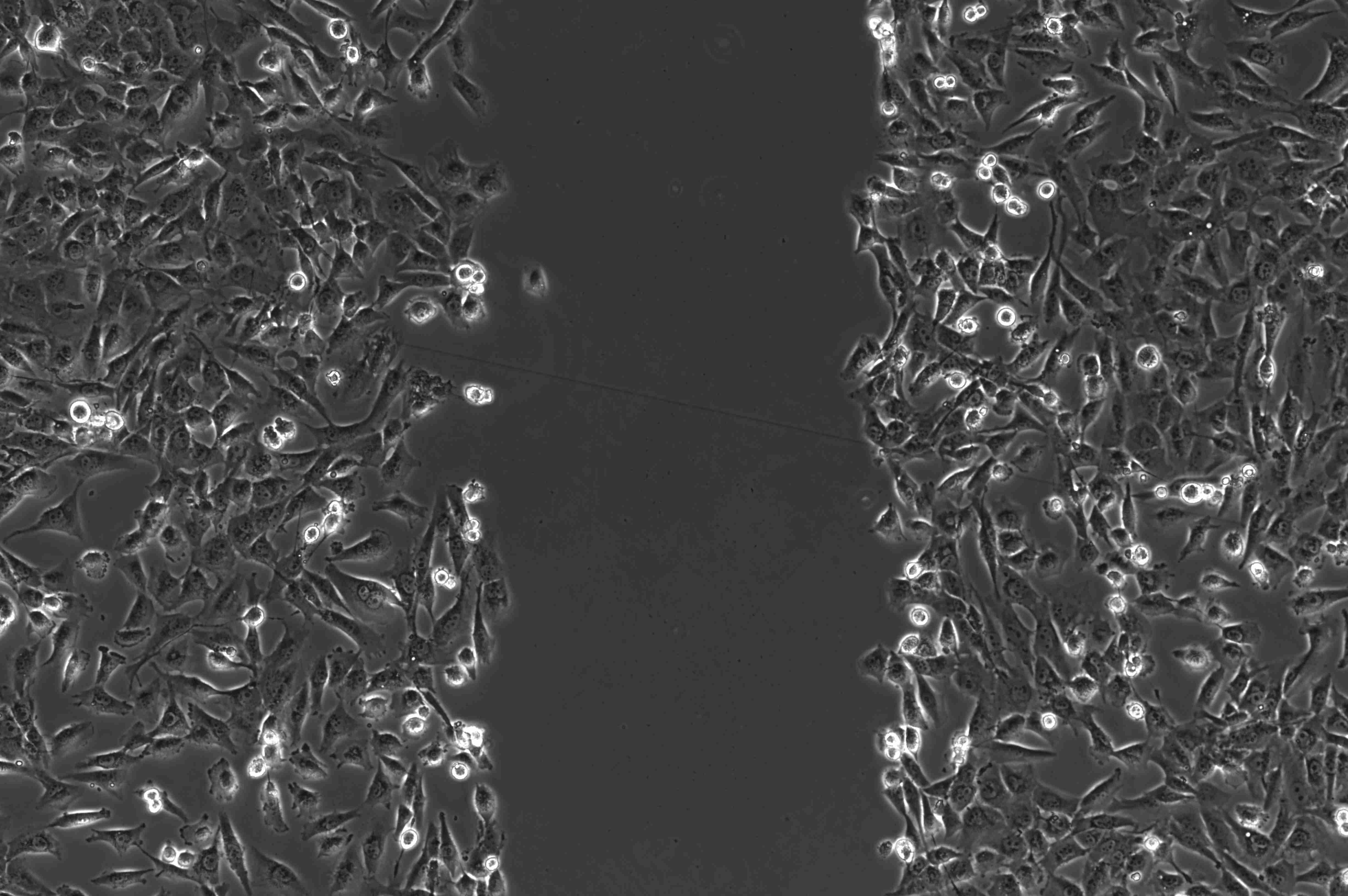

Supplement: Supplementary file 8 [file DataSheet7.ZIP › Wound healing/BT-549/6-0h-100X (1)_new.jpg]

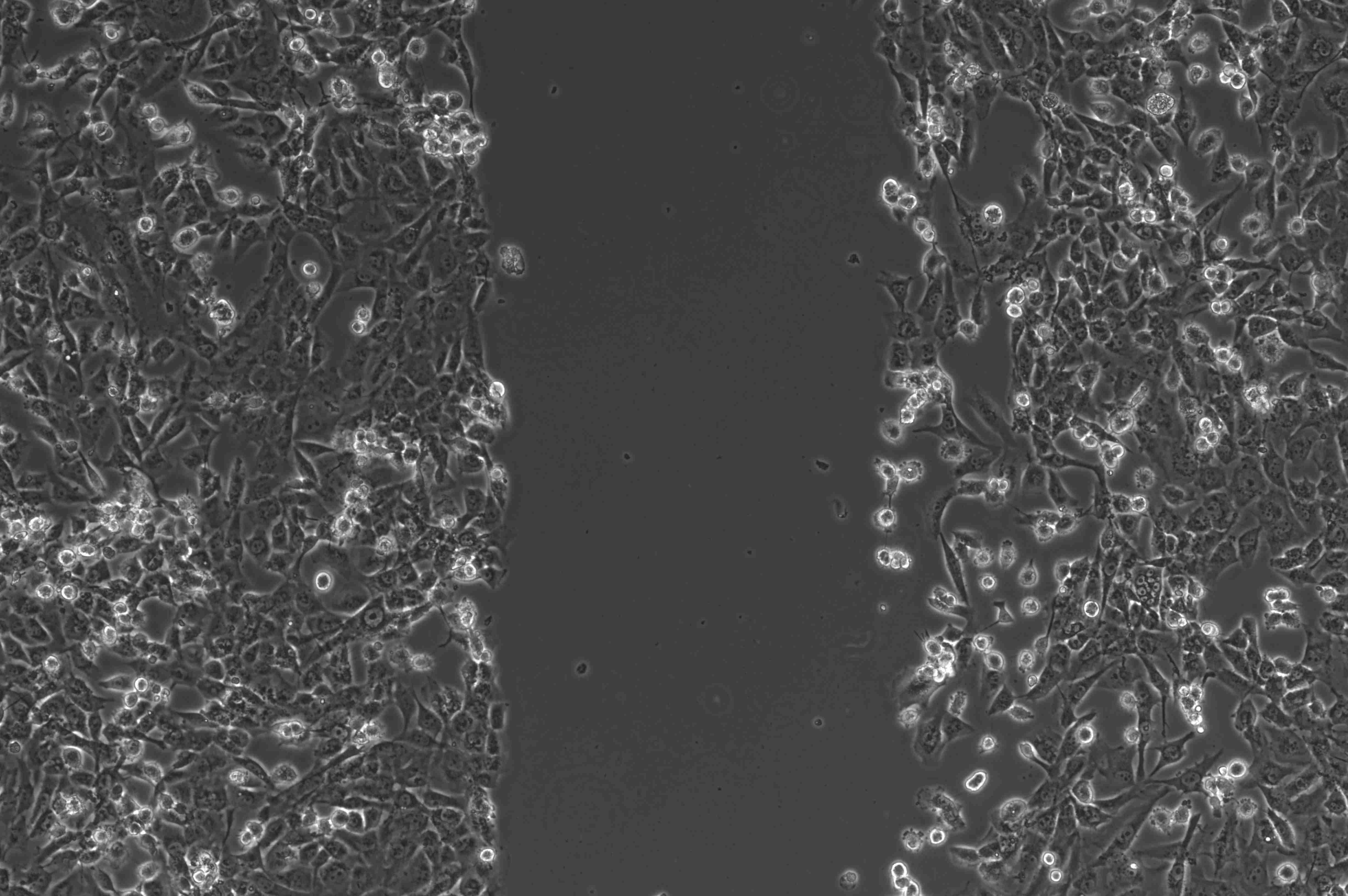

Supplement: Supplementary file 8 [file DataSheet7.ZIP › Wound healing/BT-549/6-0h-100X (2)-1_new.jpg]

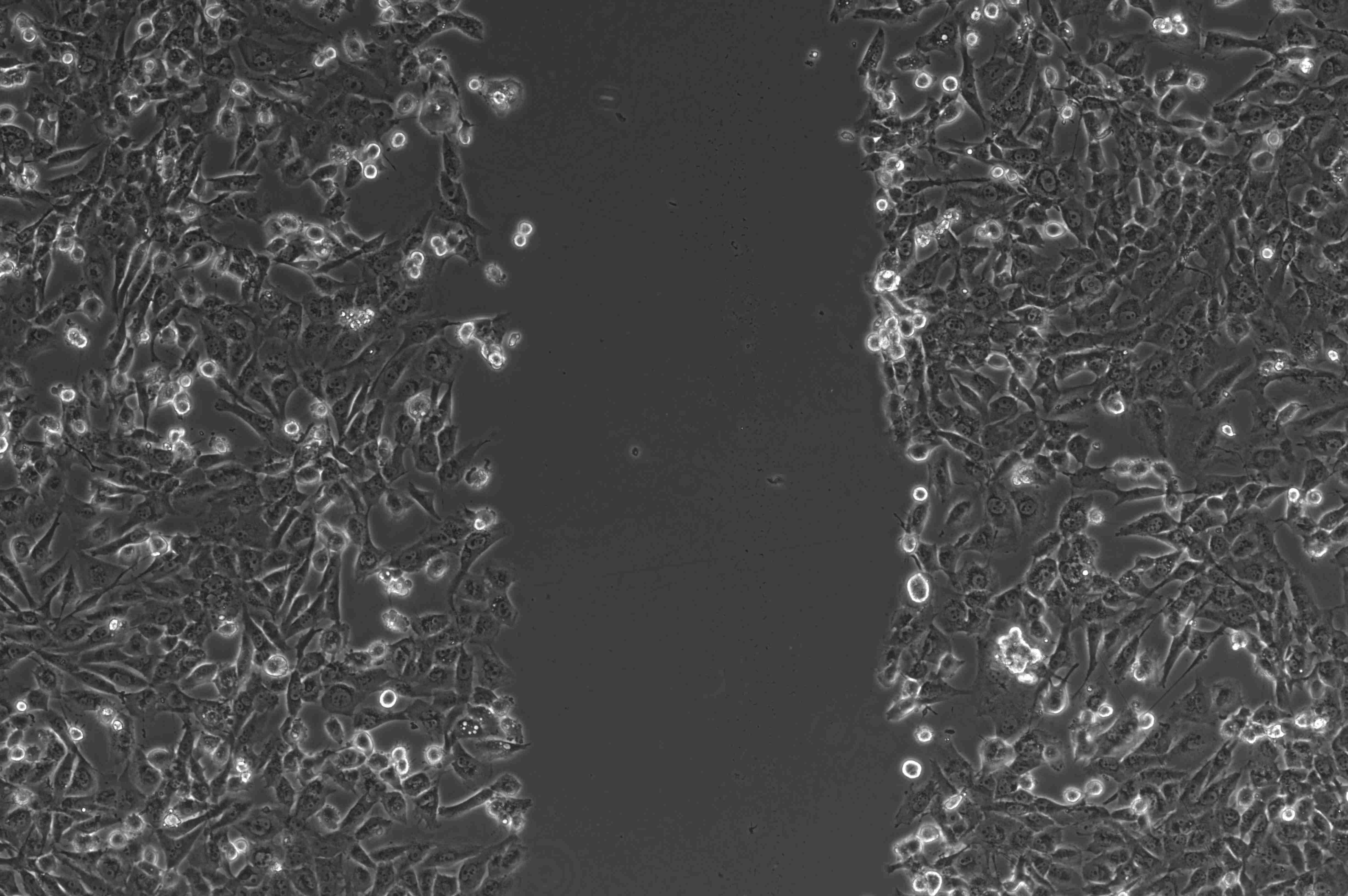

Supplement: Supplementary file 8 [file DataSheet7.ZIP › Wound healing/BT-549/6-0h-100X (3)_new.jpg]

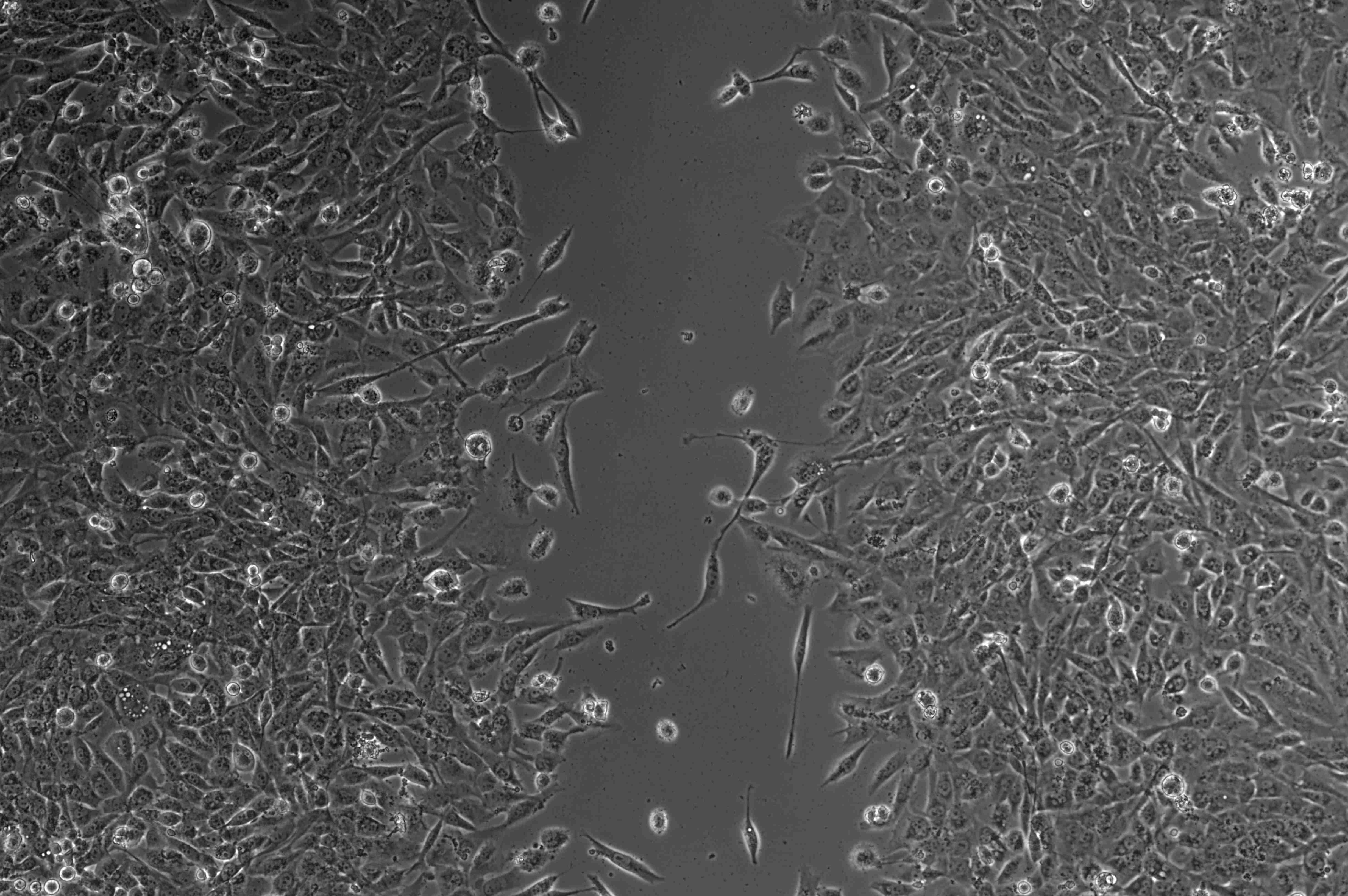

Supplement: Supplementary file 8 [file DataSheet7.ZIP › Wound healing/BT-549/6-24h-100X (1)_new.jpg]

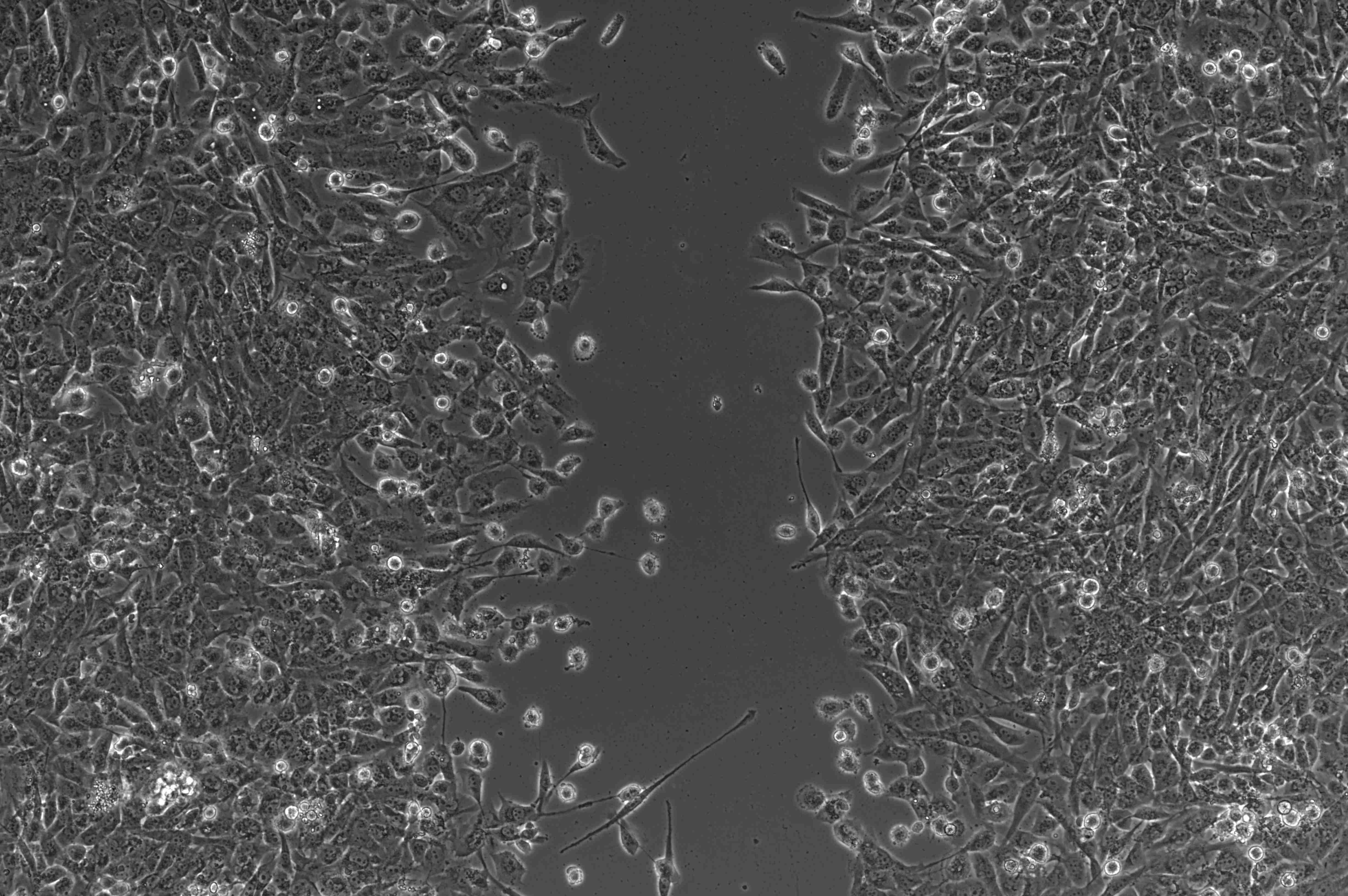

Supplement: Supplementary file 8 [file DataSheet7.ZIP › Wound healing/BT-549/6-24h-100X (2)_new.jpg]

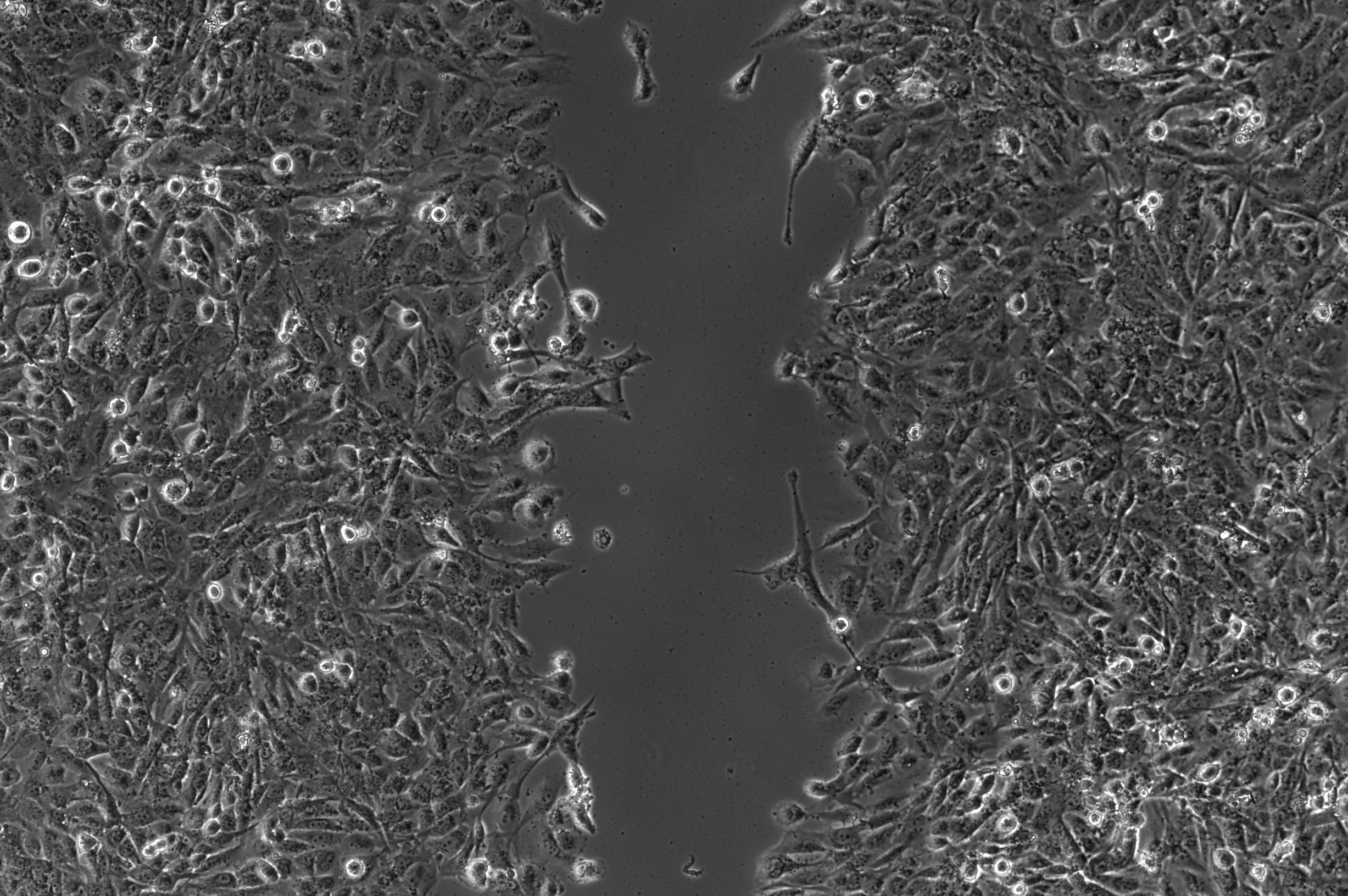

Supplement: Supplementary file 8 [file DataSheet7.ZIP › Wound healing/BT-549/6-24h-100X (3)-1.jpg]

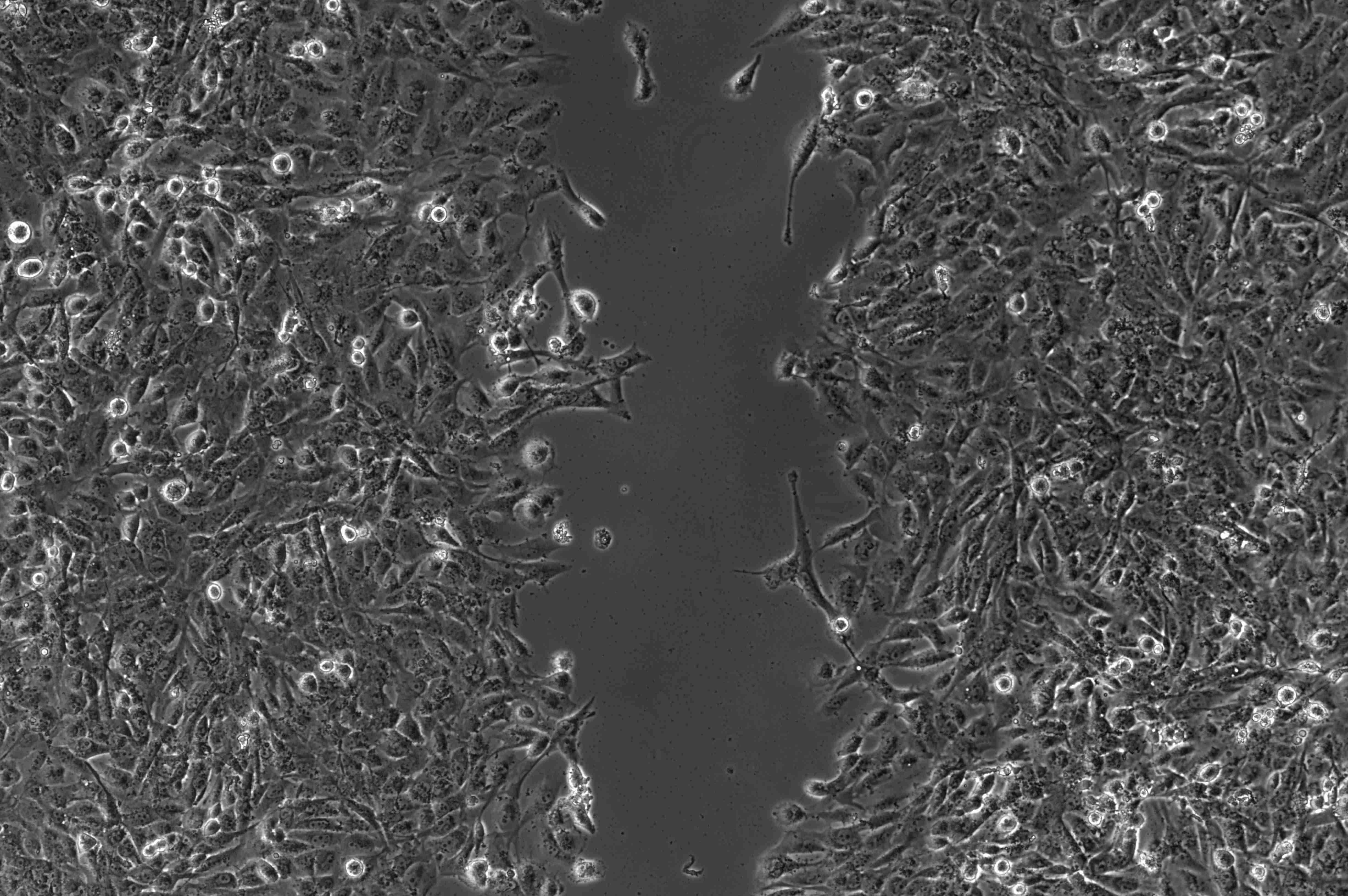

Supplement: Supplementary file 8 [file DataSheet7.ZIP › Wound healing/BT-549/6-24h-100X (3)-1_new.jpg]

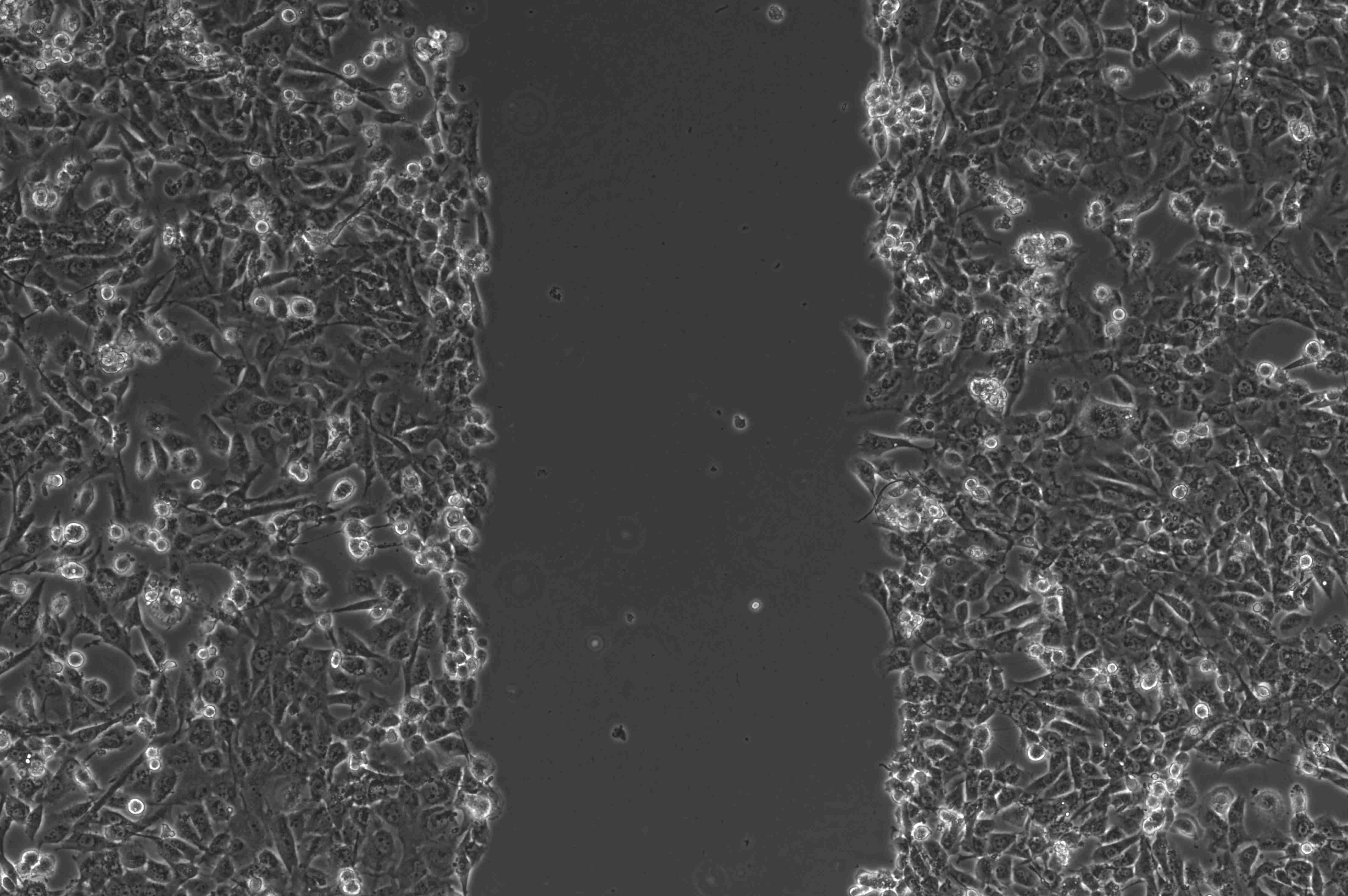

Supplement: Supplementary file 8 [file DataSheet7.ZIP › Wound healing/BT-549/7-0h-100X (1)-1_new.jpg]

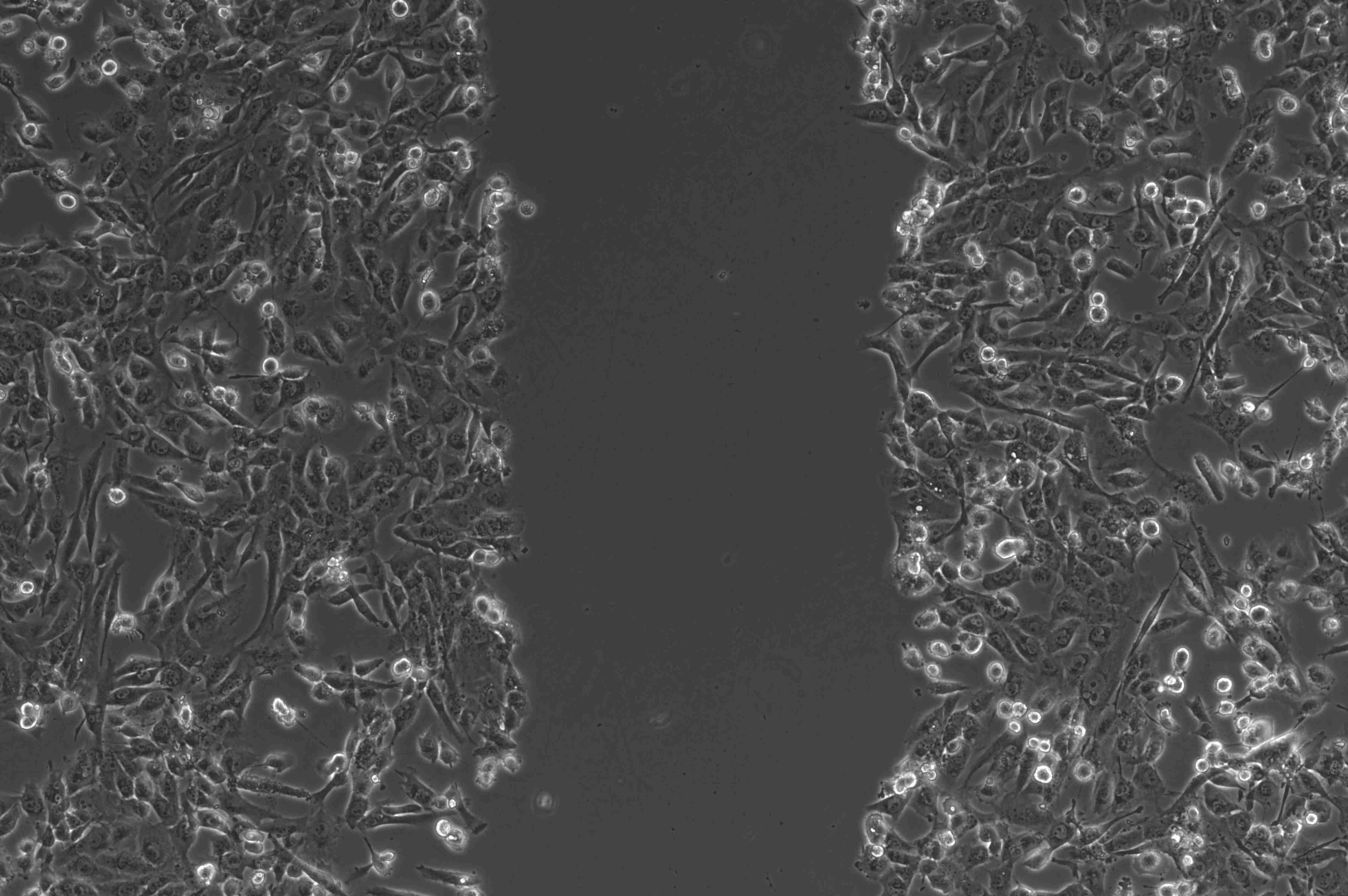

Supplement: Supplementary file 8 [file DataSheet7.ZIP › Wound healing/BT-549/7-0h-100X (2)_new.jpg]

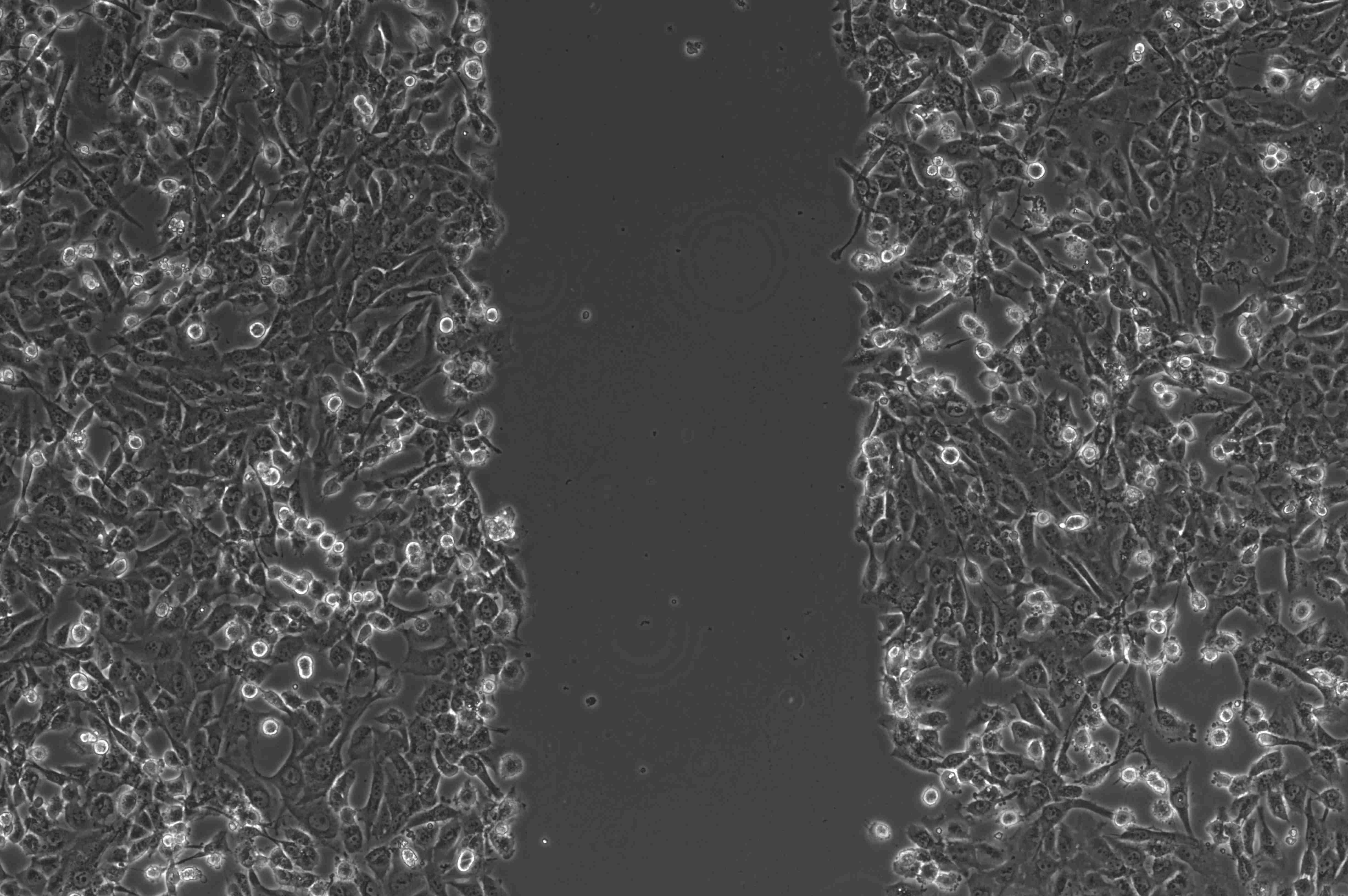

Supplement: Supplementary file 8 [file DataSheet7.ZIP › Wound healing/BT-549/7-0h-100X (3)_new.jpg]

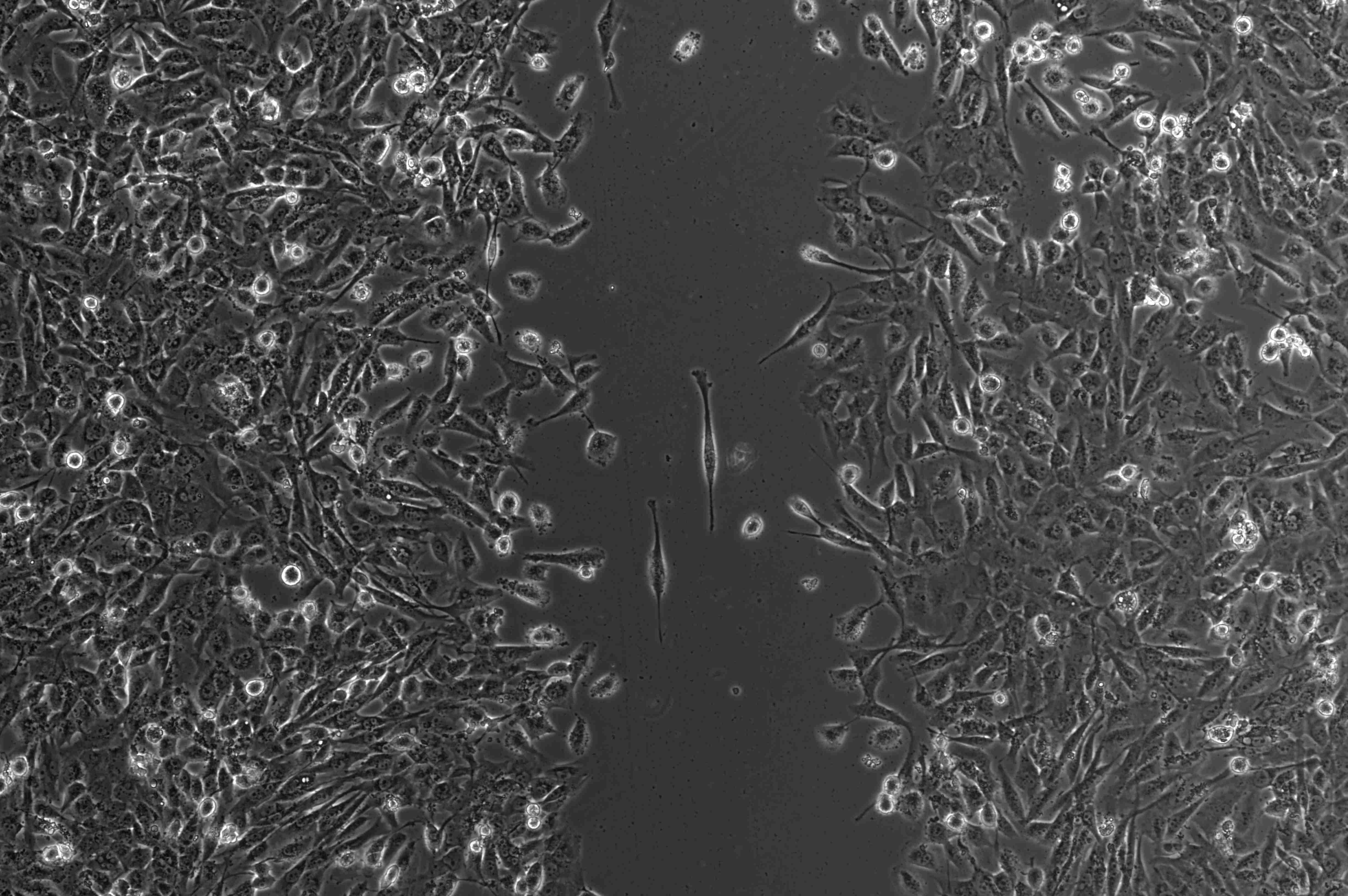

Supplement: Supplementary file 8 [file DataSheet7.ZIP › Wound healing/BT-549/7-24h-100X (1)-1_new.jpg]

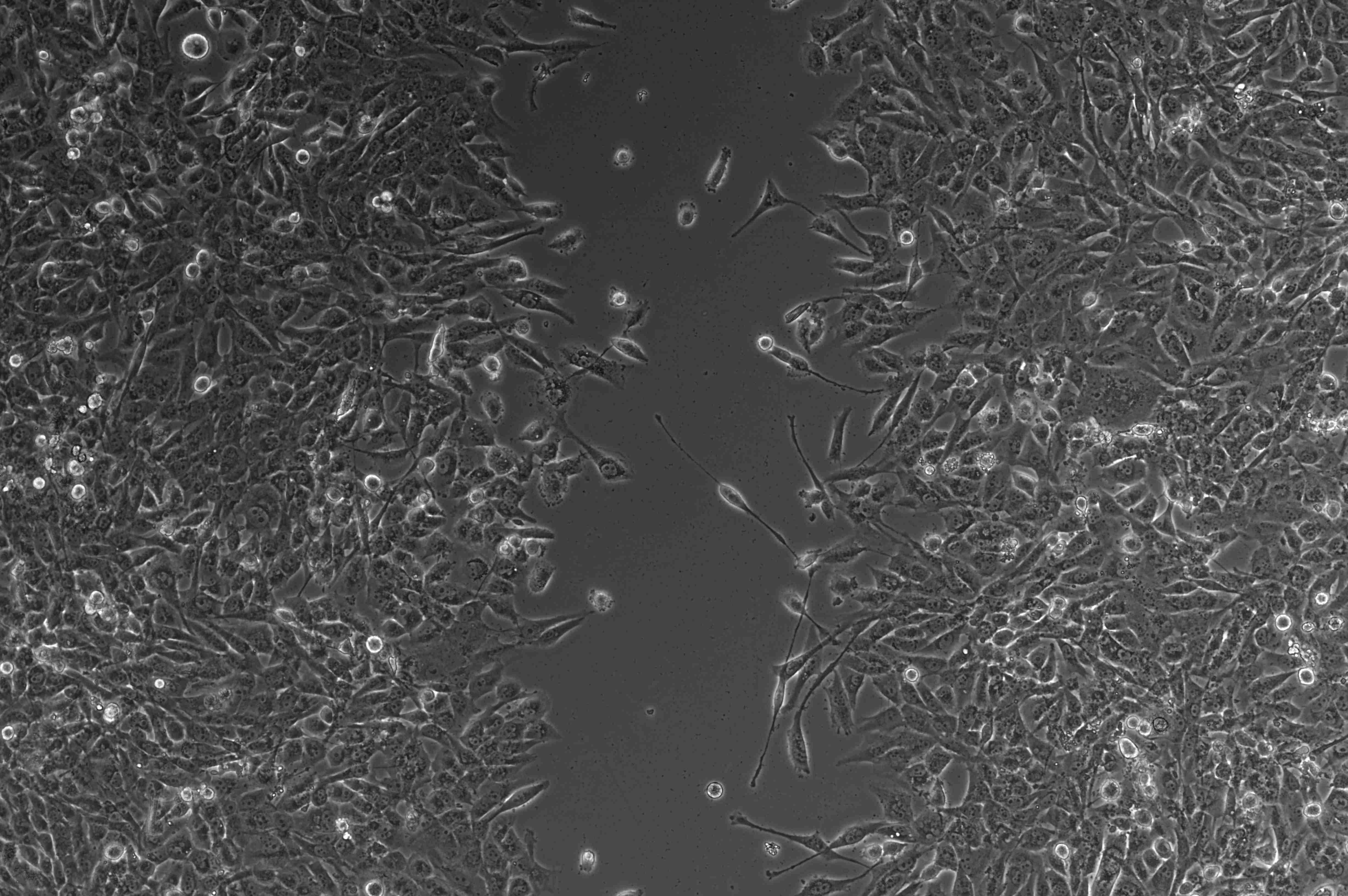

Supplement: Supplementary file 8 [file DataSheet7.ZIP › Wound healing/BT-549/7-24h-100X (2)_new.jpg]

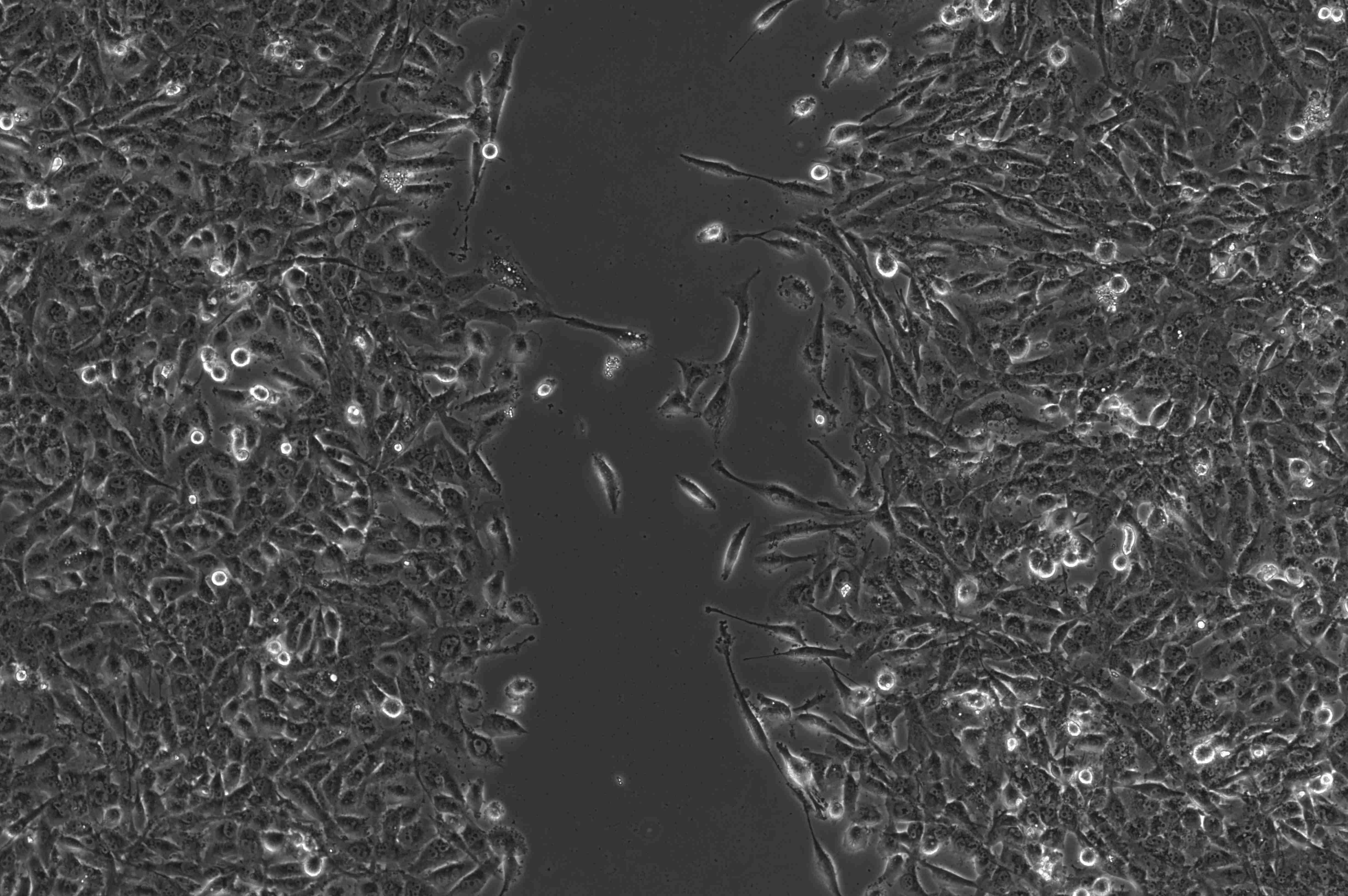

Supplement: Supplementary file 8 [file DataSheet7.ZIP › Wound healing/BT-549/7-24h-100X (3)_new.jpg]

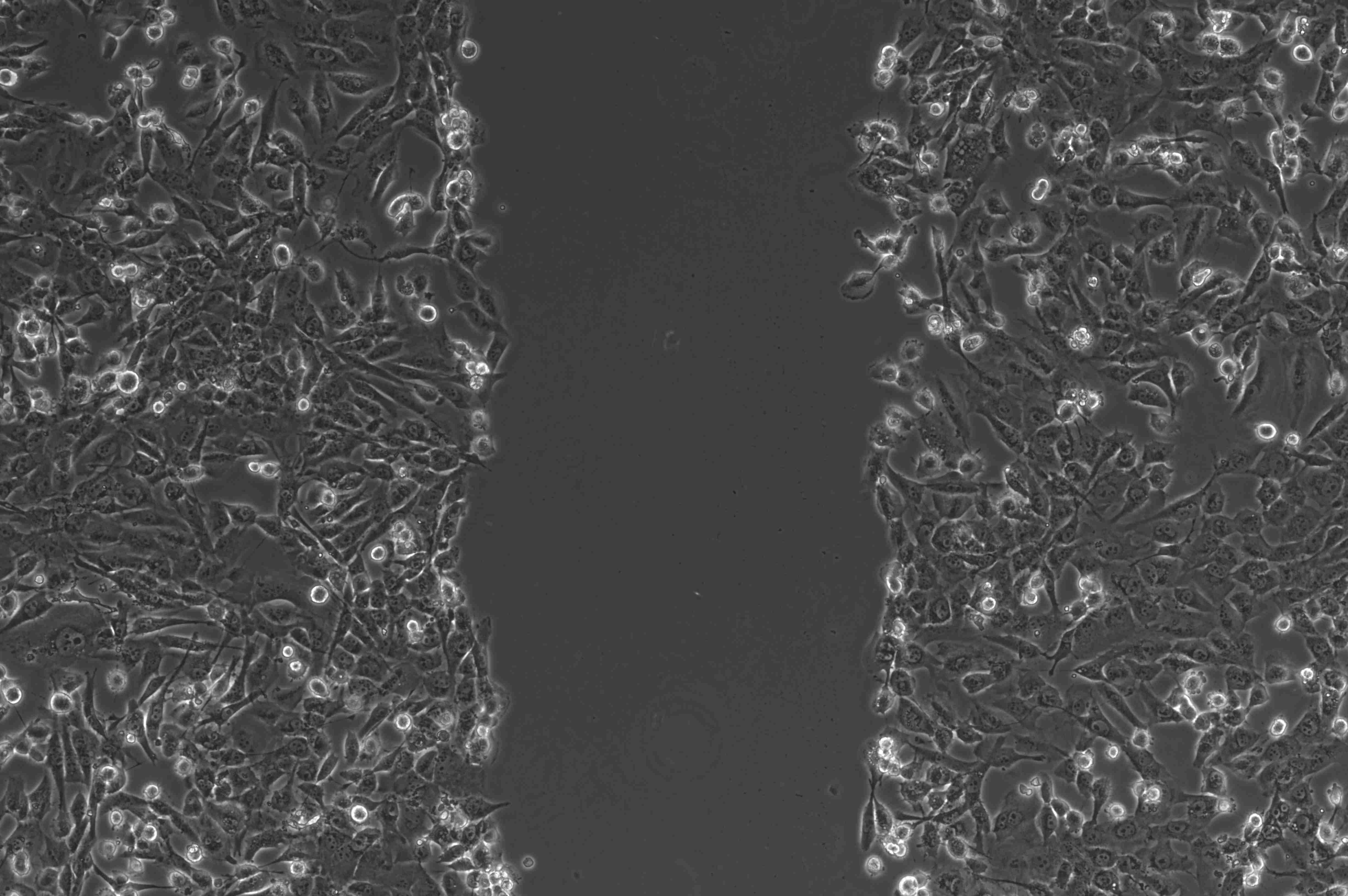

Supplement: Supplementary file 8 [file DataSheet7.ZIP › Wound healing/BT-549/8-0h-100X (1)-1_new.jpg]

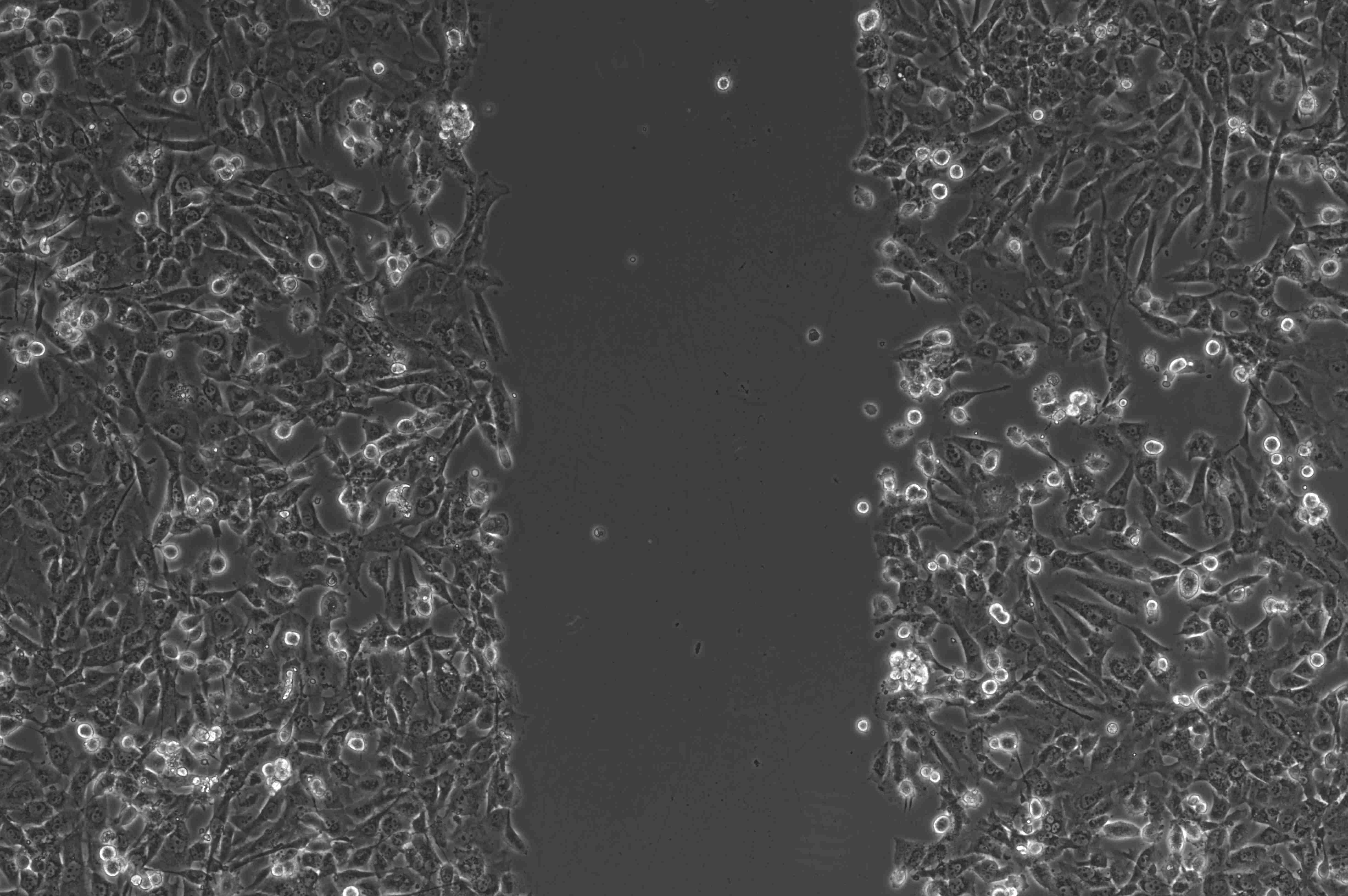

Supplement: Supplementary file 8 [file DataSheet7.ZIP › Wound healing/BT-549/8-0h-100X (2)_new.jpg]

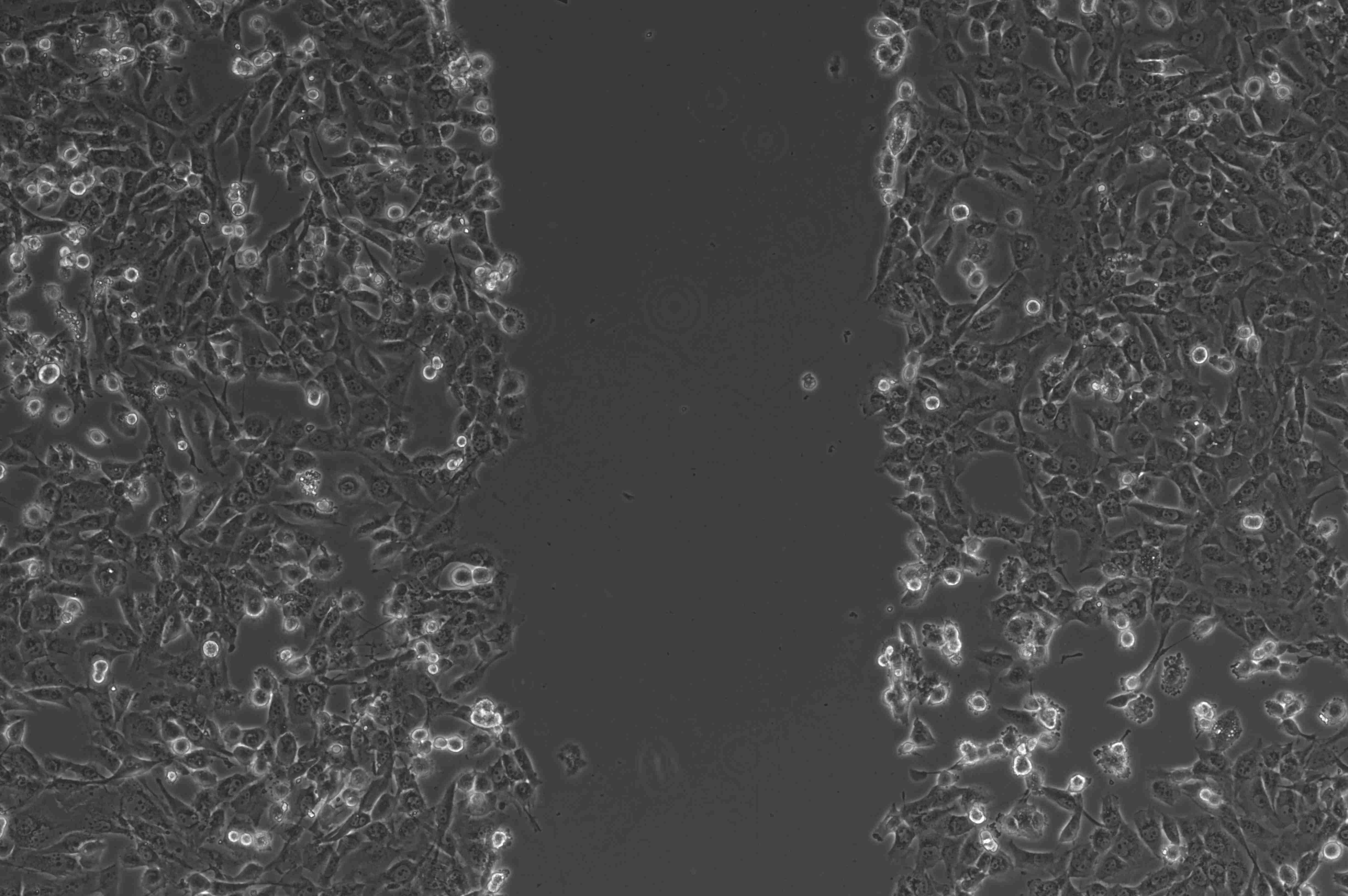

Supplement: Supplementary file 8 [file DataSheet7.ZIP › Wound healing/BT-549/8-0h-100X (3)_new.jpg]

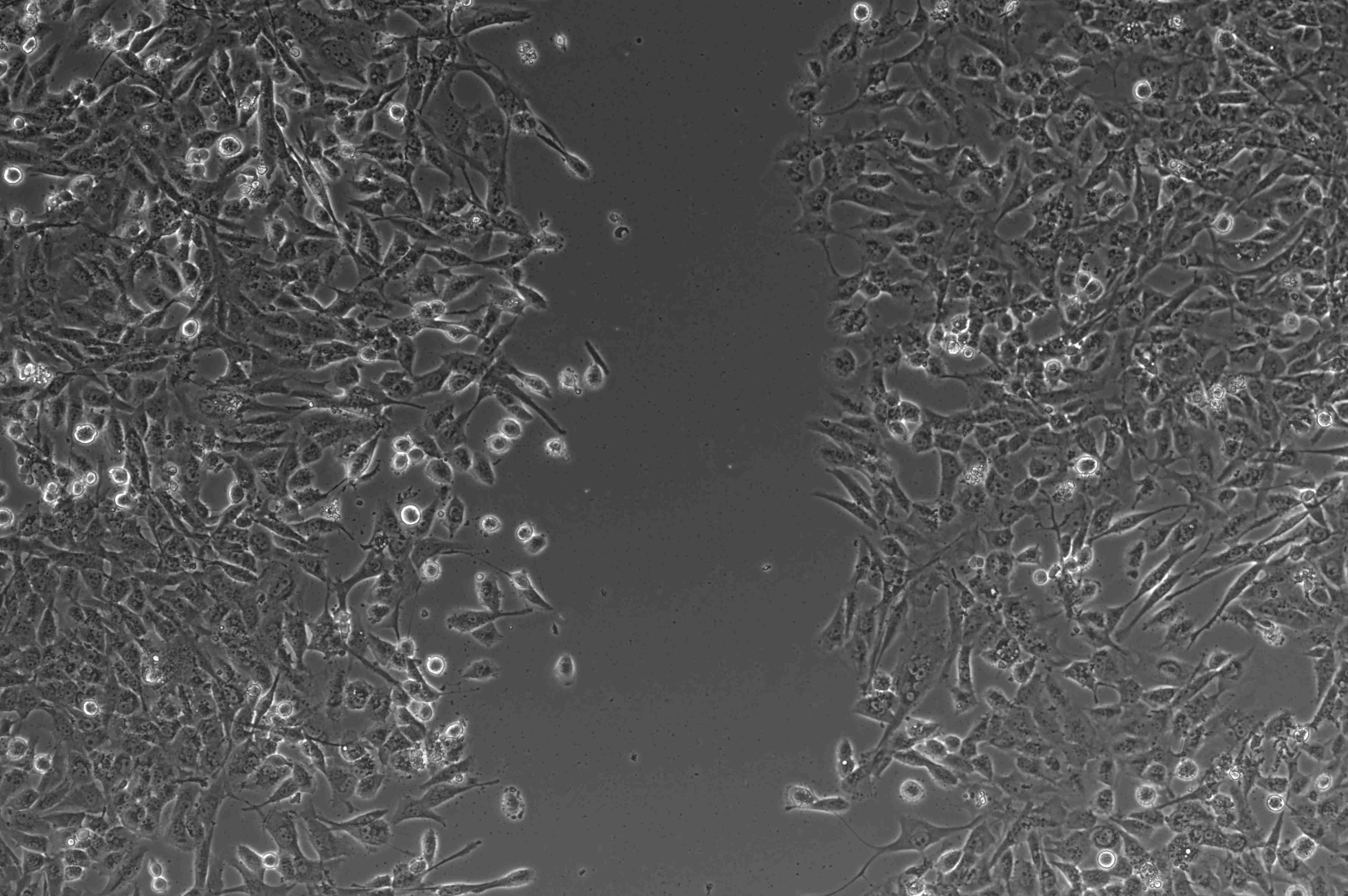

Supplement: Supplementary file 8 [file DataSheet7.ZIP › Wound healing/BT-549/8-24h-100X (1)_new.jpg]

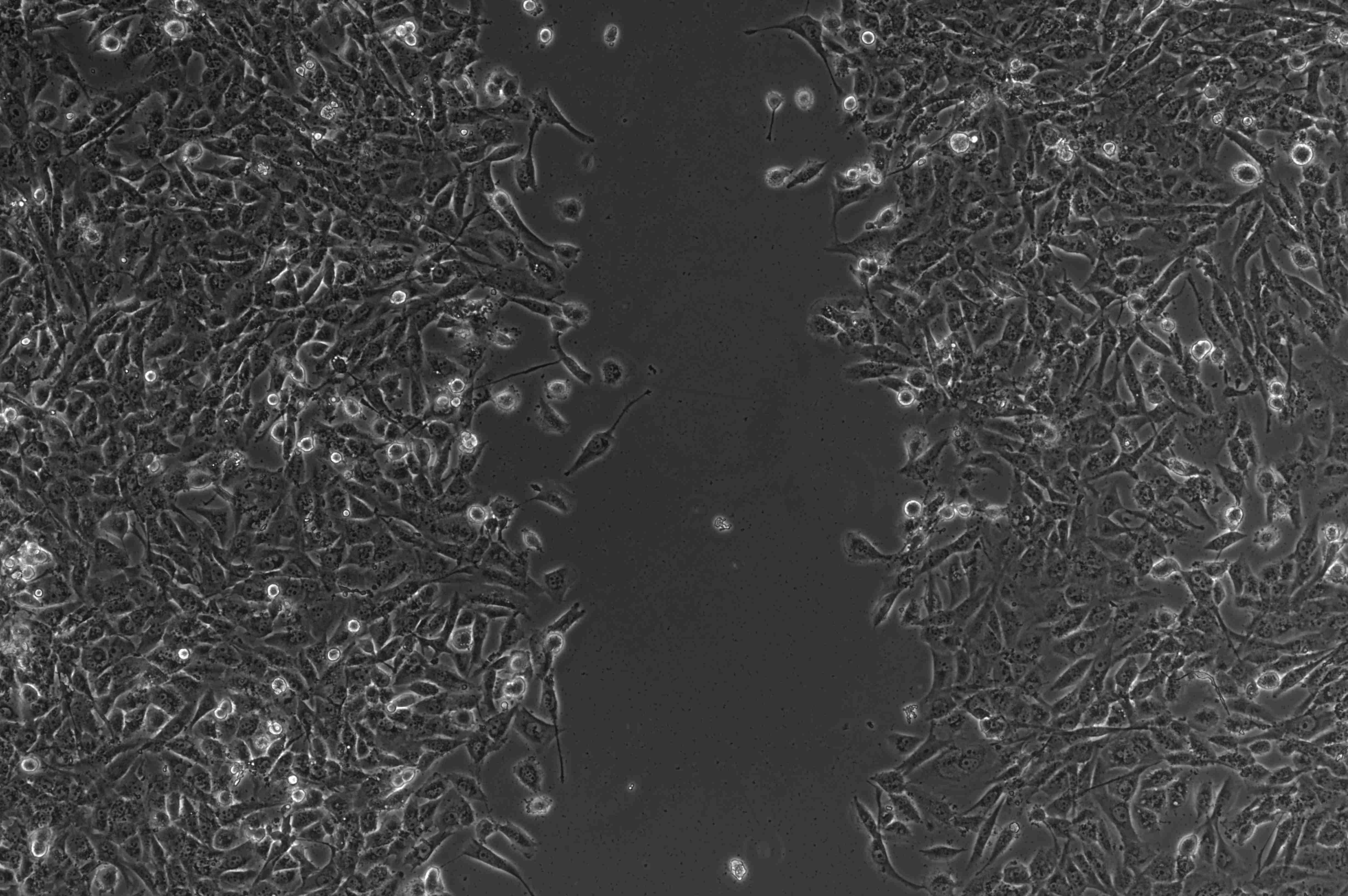

Supplement: Supplementary file 8 [file DataSheet7.ZIP › Wound healing/BT-549/8-24h-100X (2)_new.jpg]

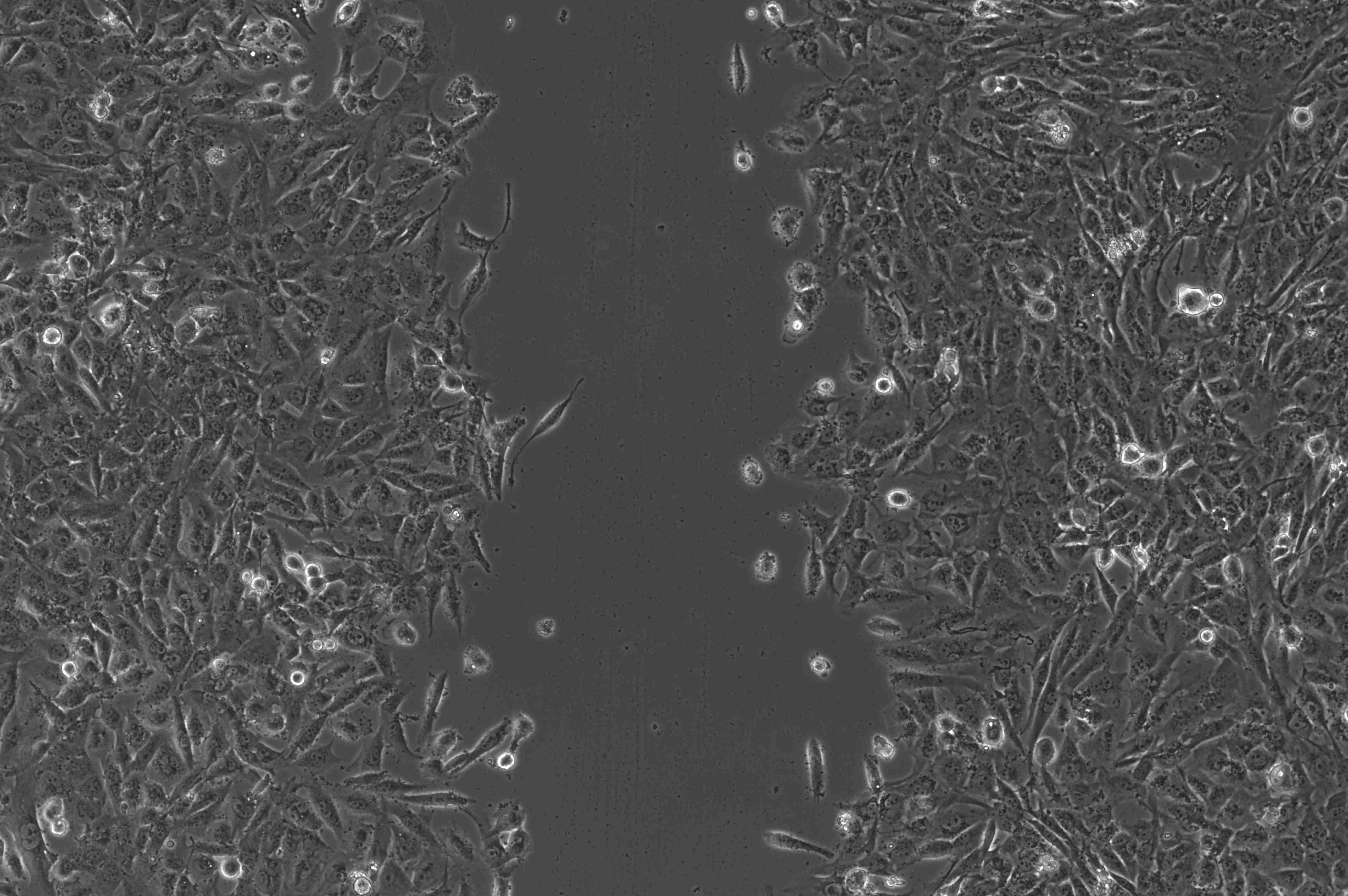

Supplement: Supplementary file 8 [file DataSheet7.ZIP › Wound healing/BT-549/8-24h-100X (3)_new.jpg]

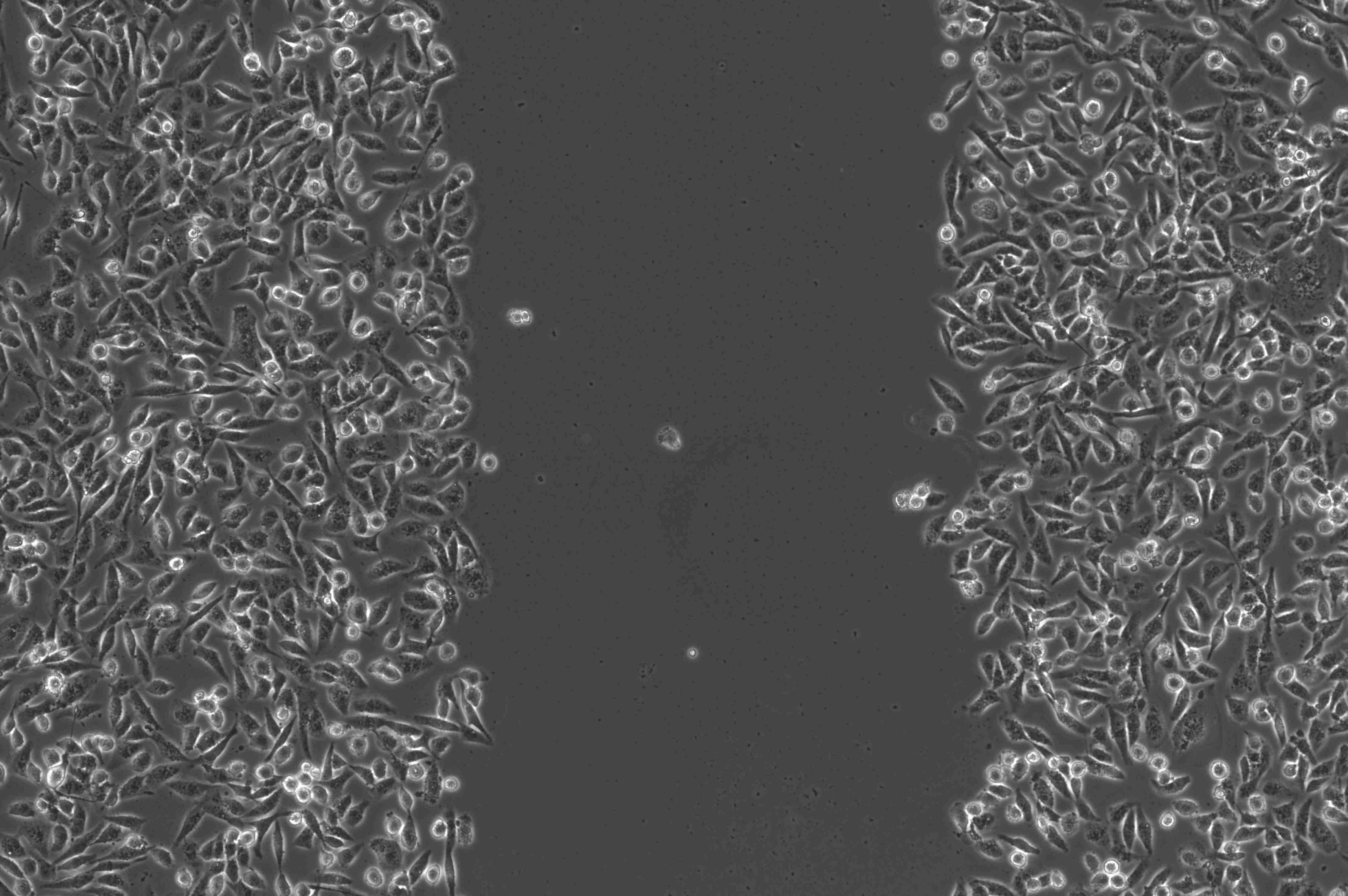

Supplement: Supplementary file 8 [file DataSheet7.ZIP › Wound healing/MDA-MB-231/1-0h-100X (1)-1_new.jpg]

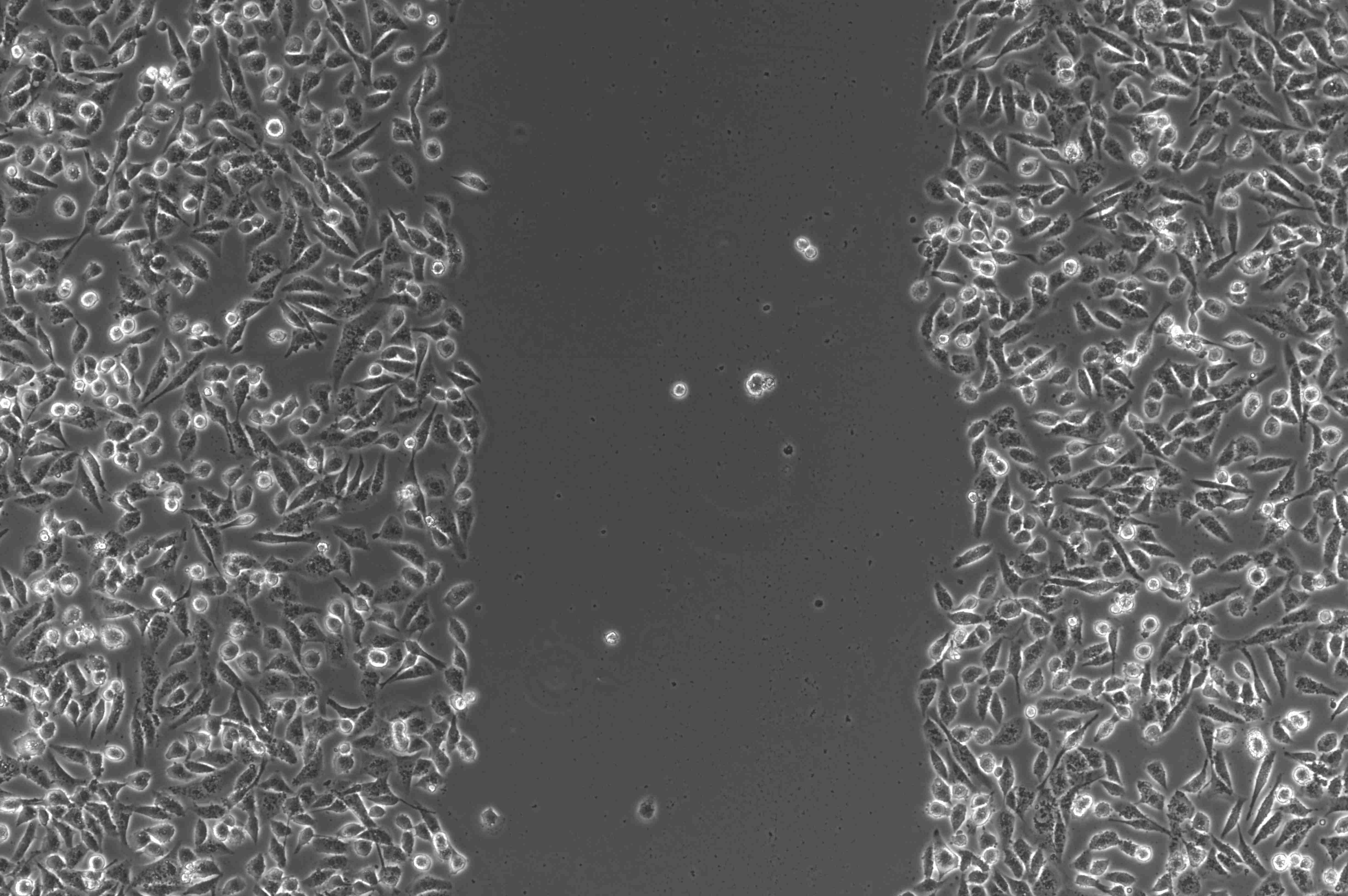

Supplement: Supplementary file 8 [file DataSheet7.ZIP › Wound healing/MDA-MB-231/1-0h-100X (2)_new.jpg]

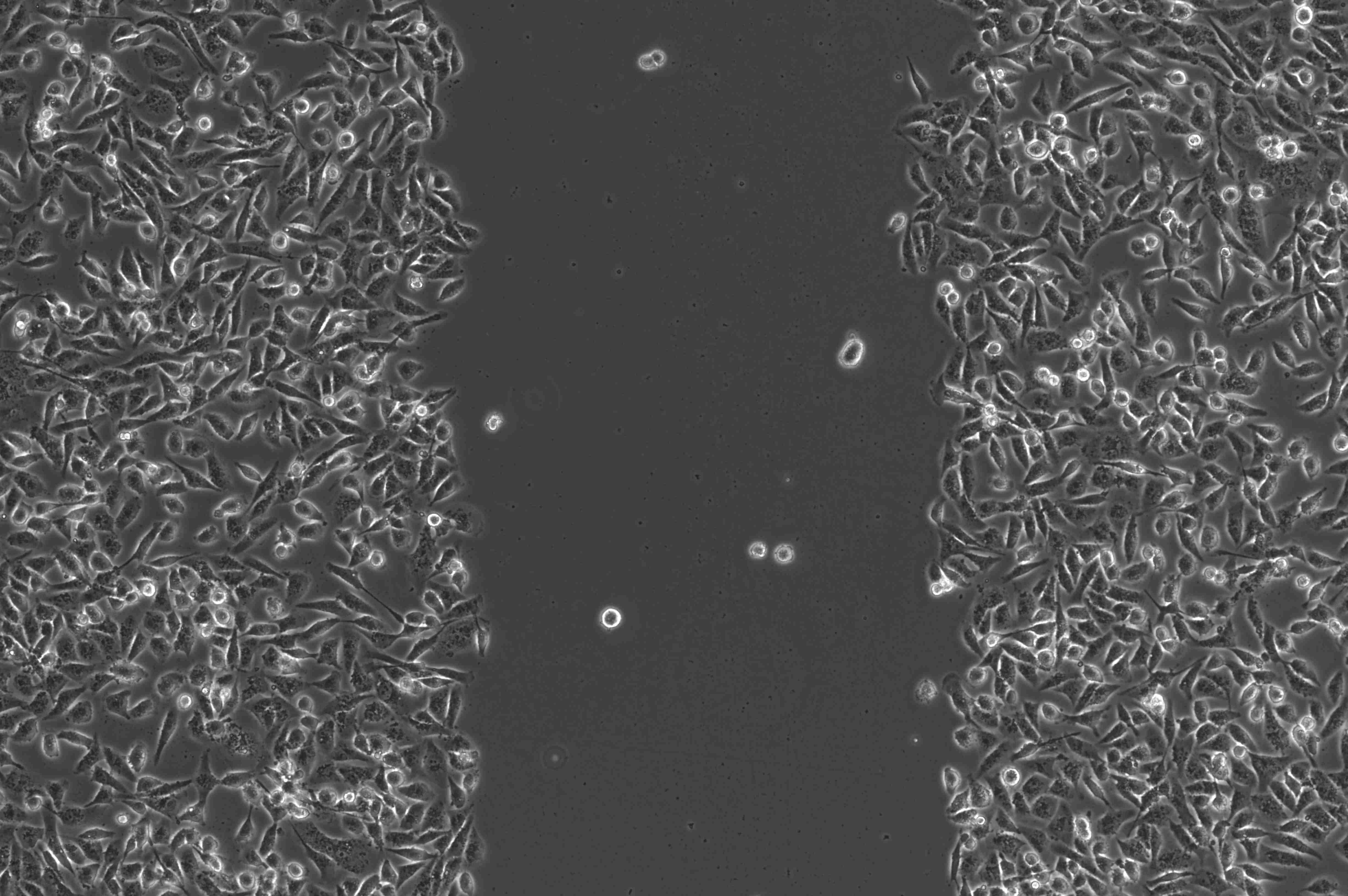

Supplement: Supplementary file 8 [file DataSheet7.ZIP › Wound healing/MDA-MB-231/1-0h-100X (3)_new.jpg]

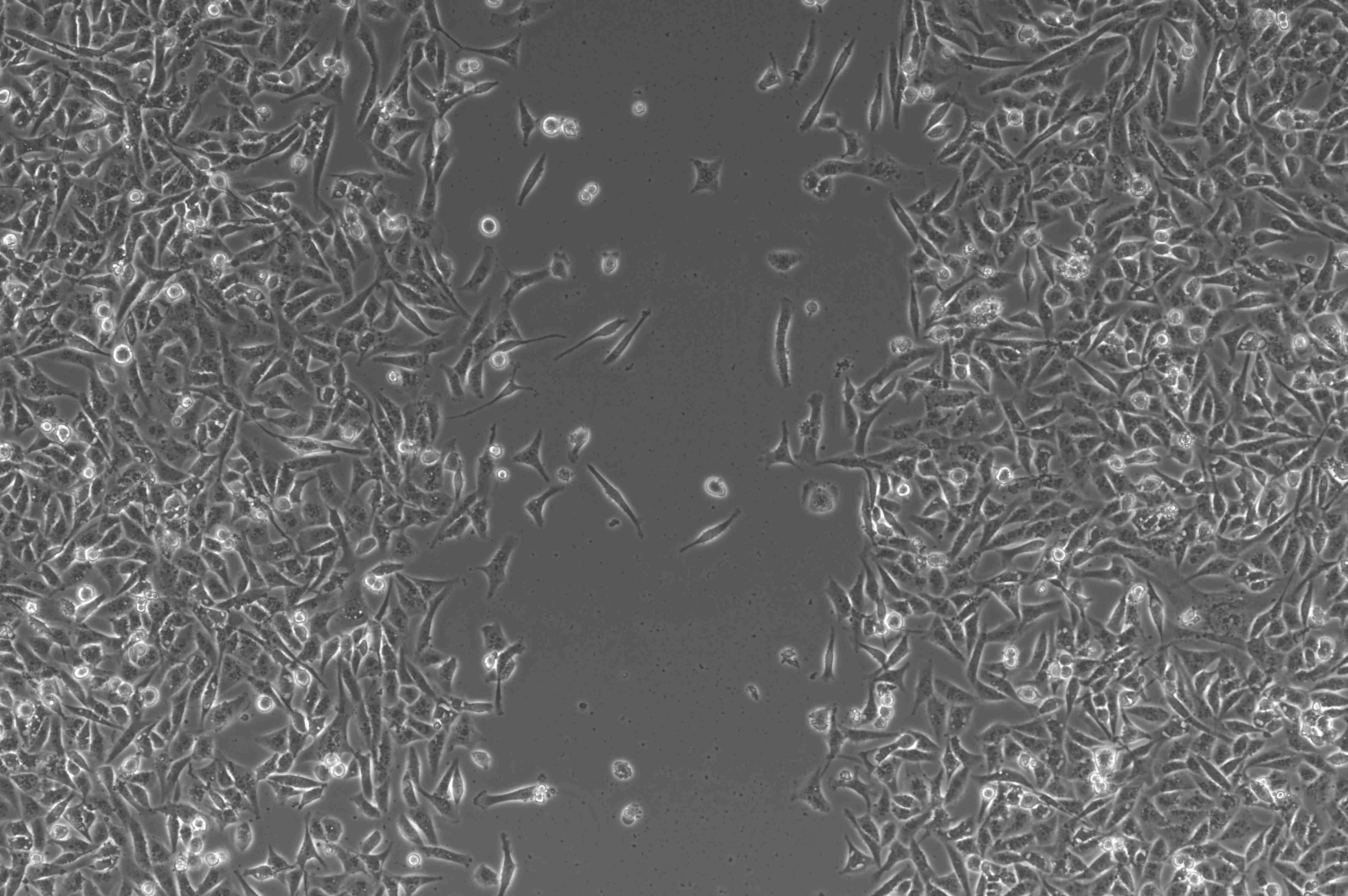

Supplement: Supplementary file 8 [file DataSheet7.ZIP › Wound healing/MDA-MB-231/1-24h-100X (1)_new.jpg]

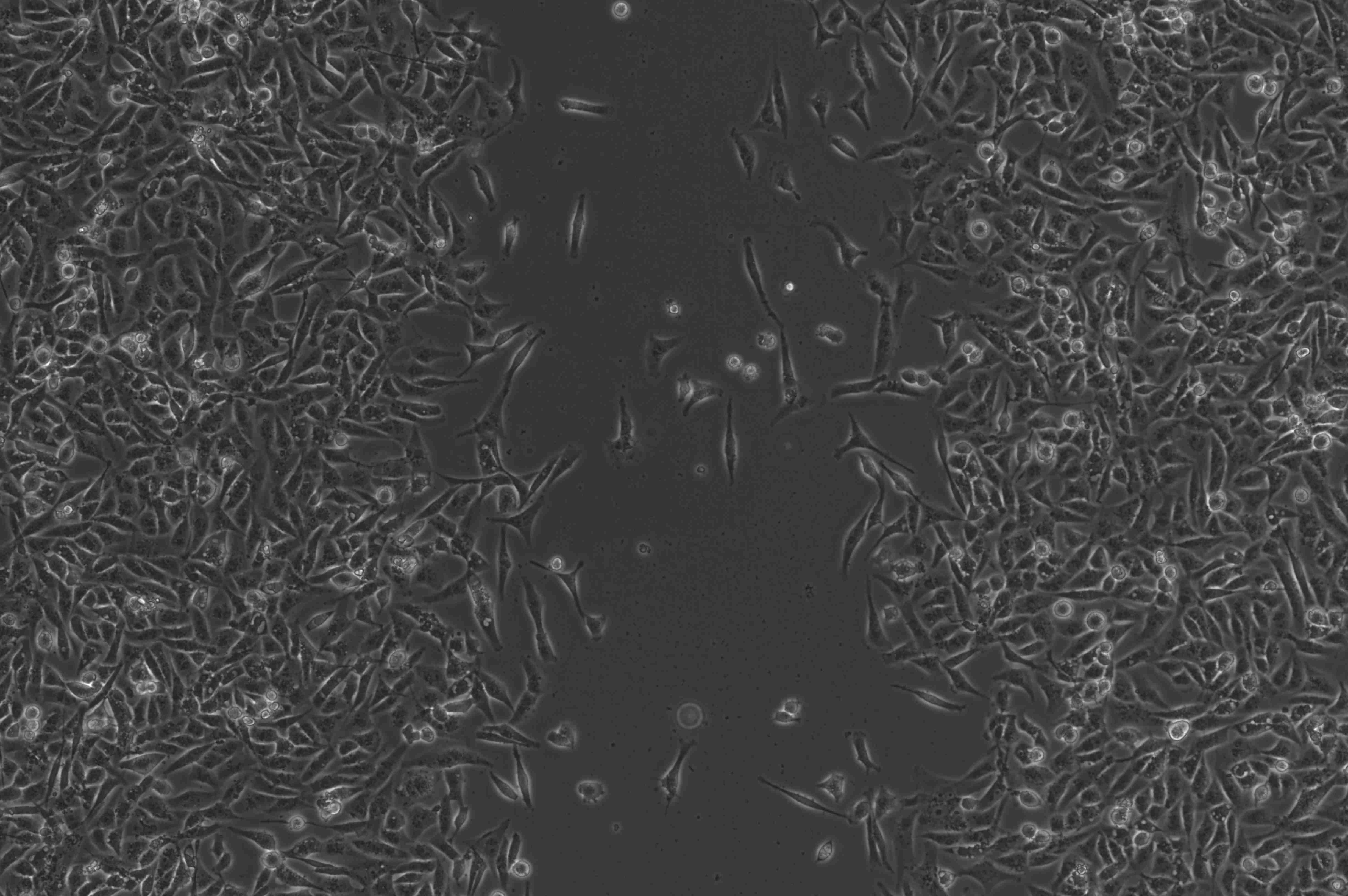

Supplement: Supplementary file 8 [file DataSheet7.ZIP › Wound healing/MDA-MB-231/1-24h-100X (2)-1_new.jpg]

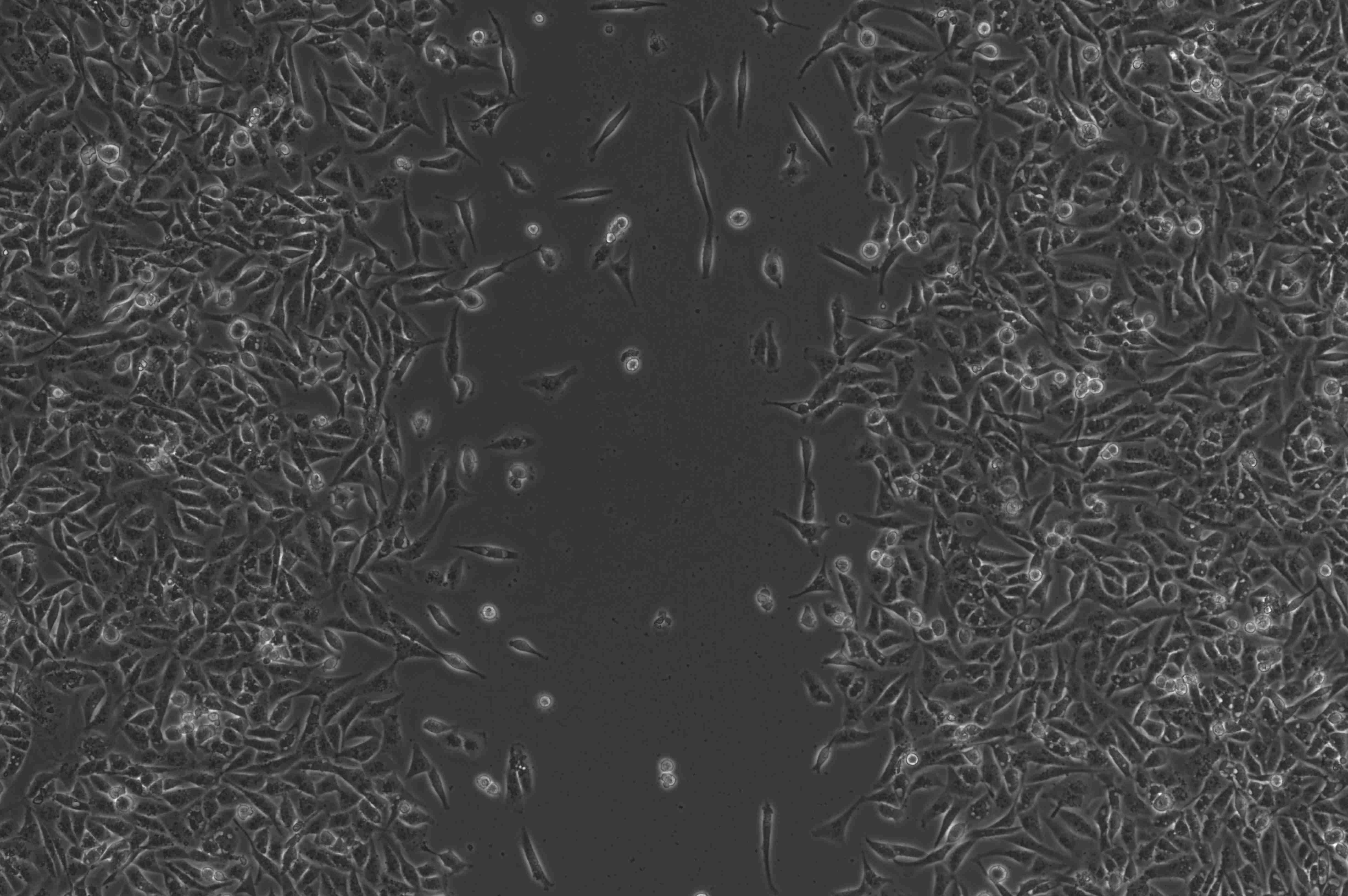

Supplement: Supplementary file 8 [file DataSheet7.ZIP › Wound healing/MDA-MB-231/1-24h-100X (3)_new.jpg]

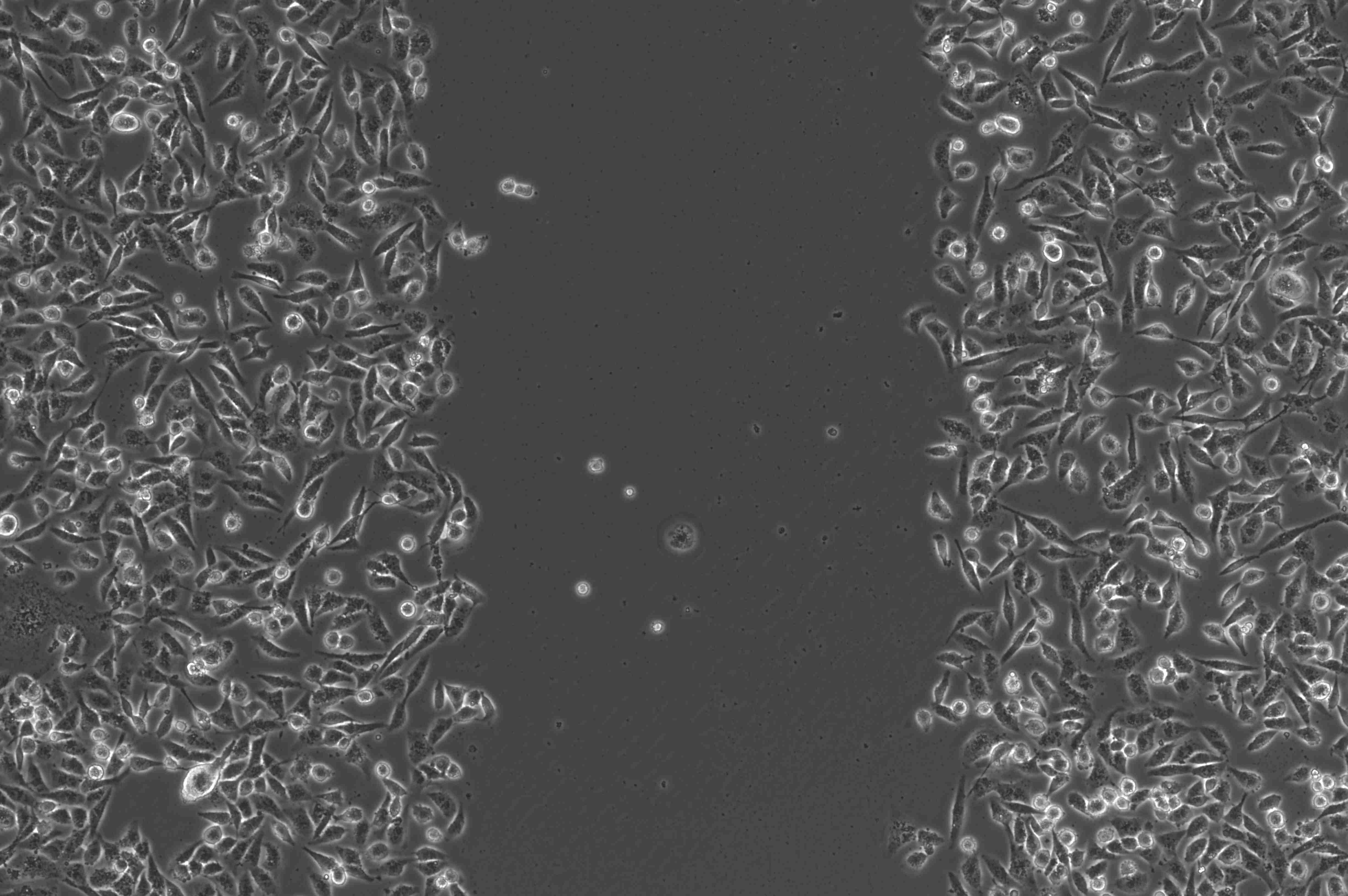

Supplement: Supplementary file 8 [file DataSheet7.ZIP › Wound healing/MDA-MB-231/2-0h-100X (1)_new.jpg]

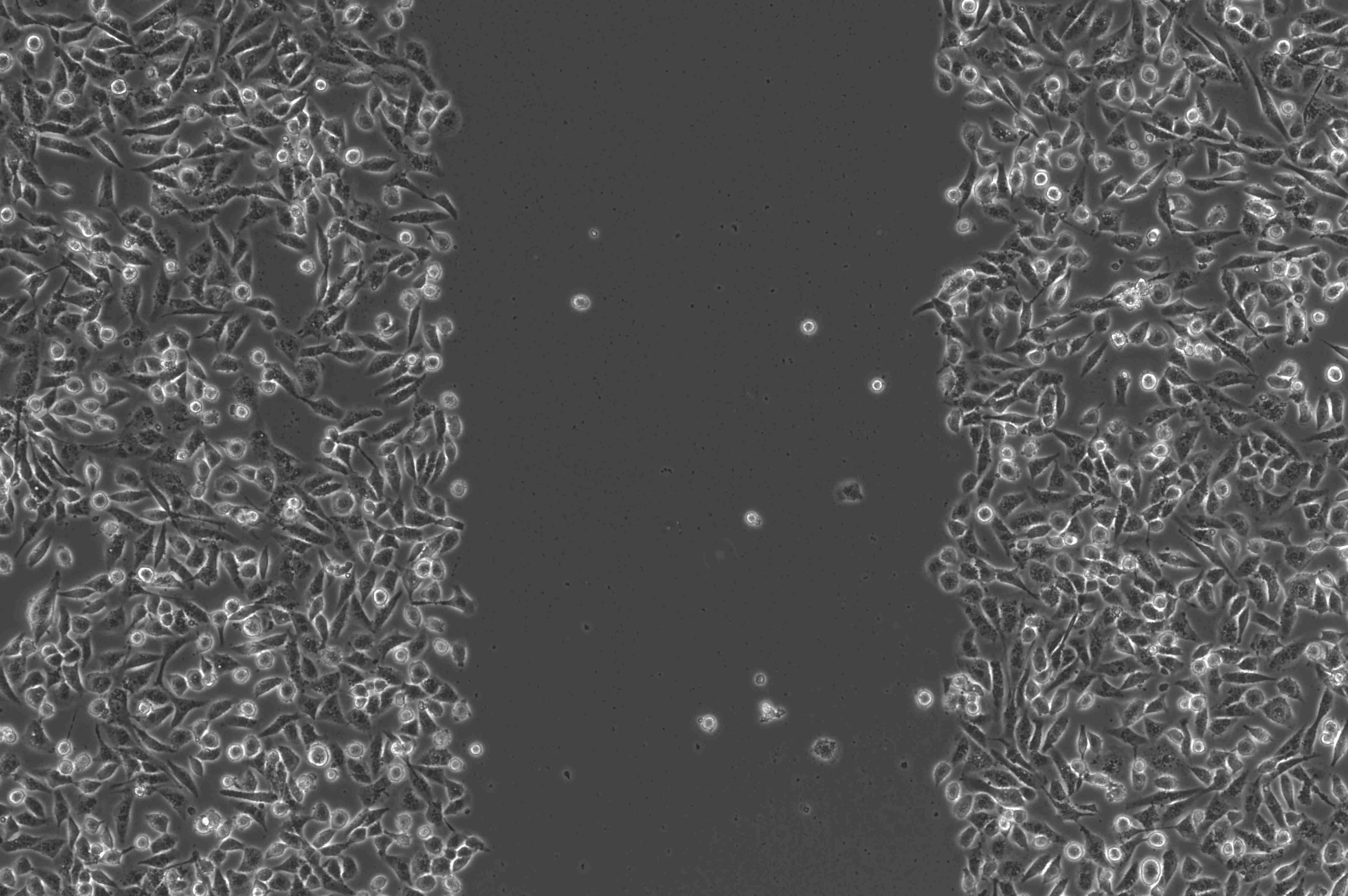

Supplement: Supplementary file 8 [file DataSheet7.ZIP › Wound healing/MDA-MB-231/2-0h-100X (2)_new.jpg]

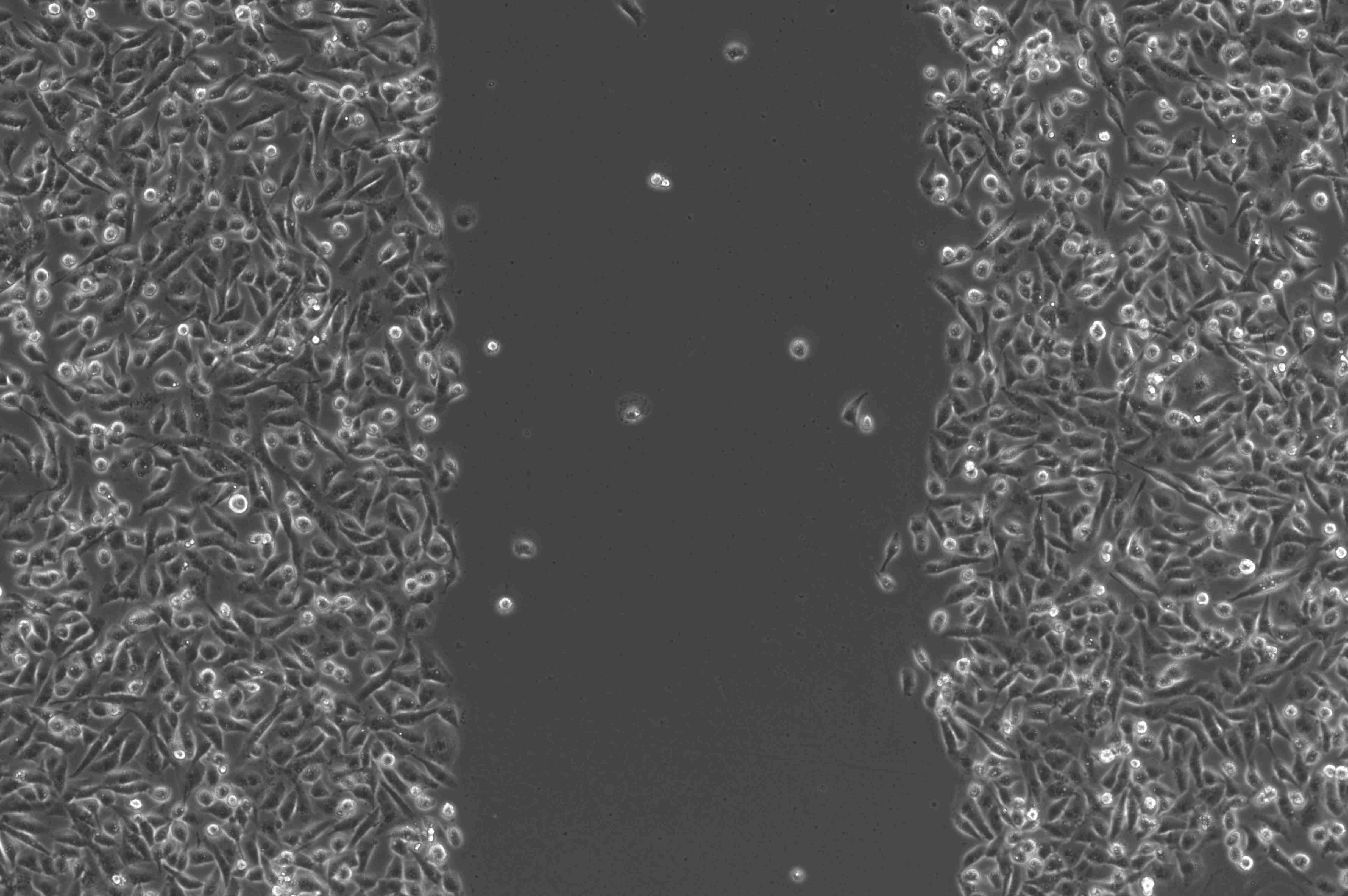

Supplement: Supplementary file 8 [file DataSheet7.ZIP › Wound healing/MDA-MB-231/2-0h-100X (3)_new.jpg]

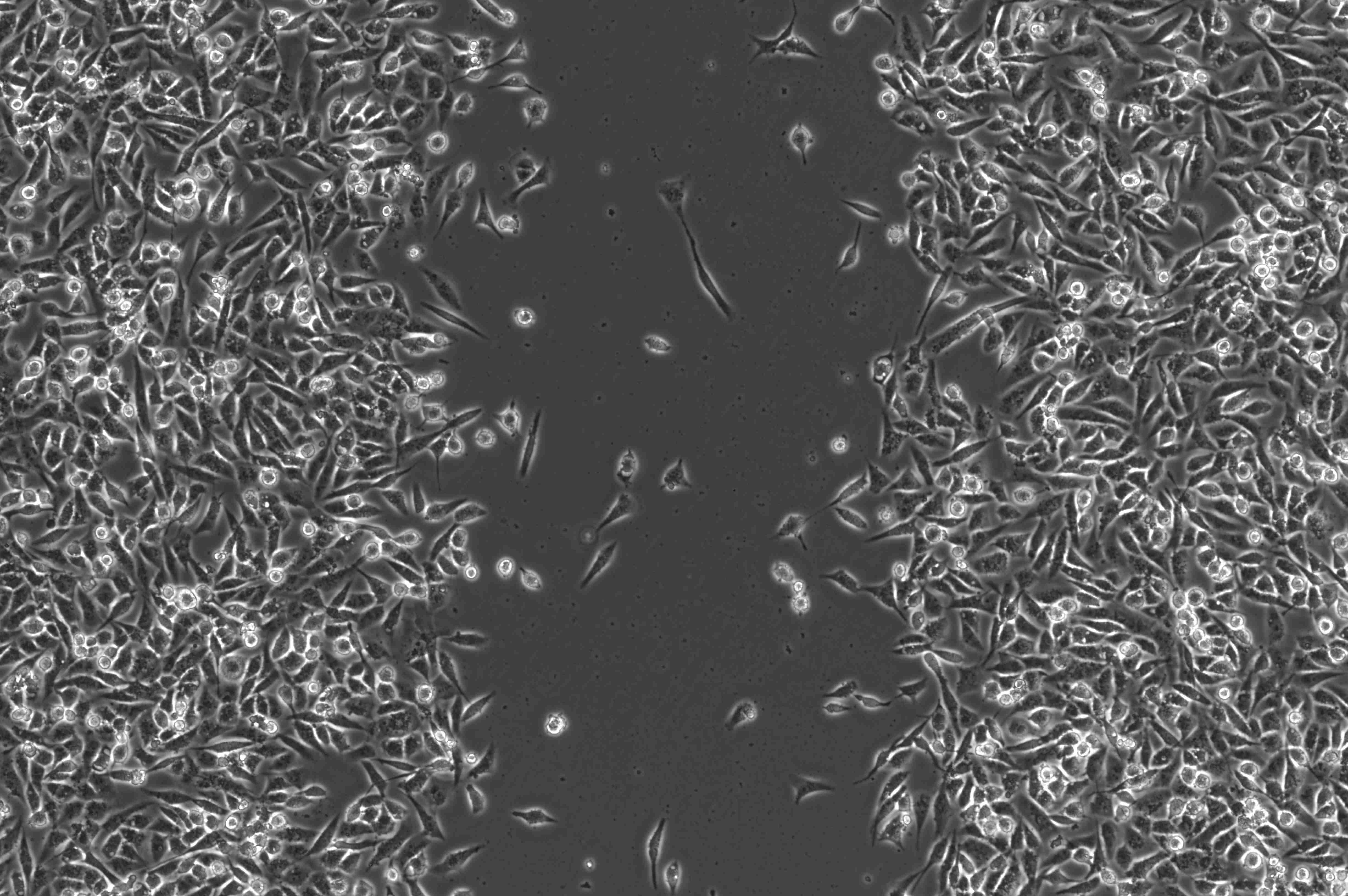

Supplement: Supplementary file 8 [file DataSheet7.ZIP › Wound healing/MDA-MB-231/2-24h-100X (1)_new.jpg]

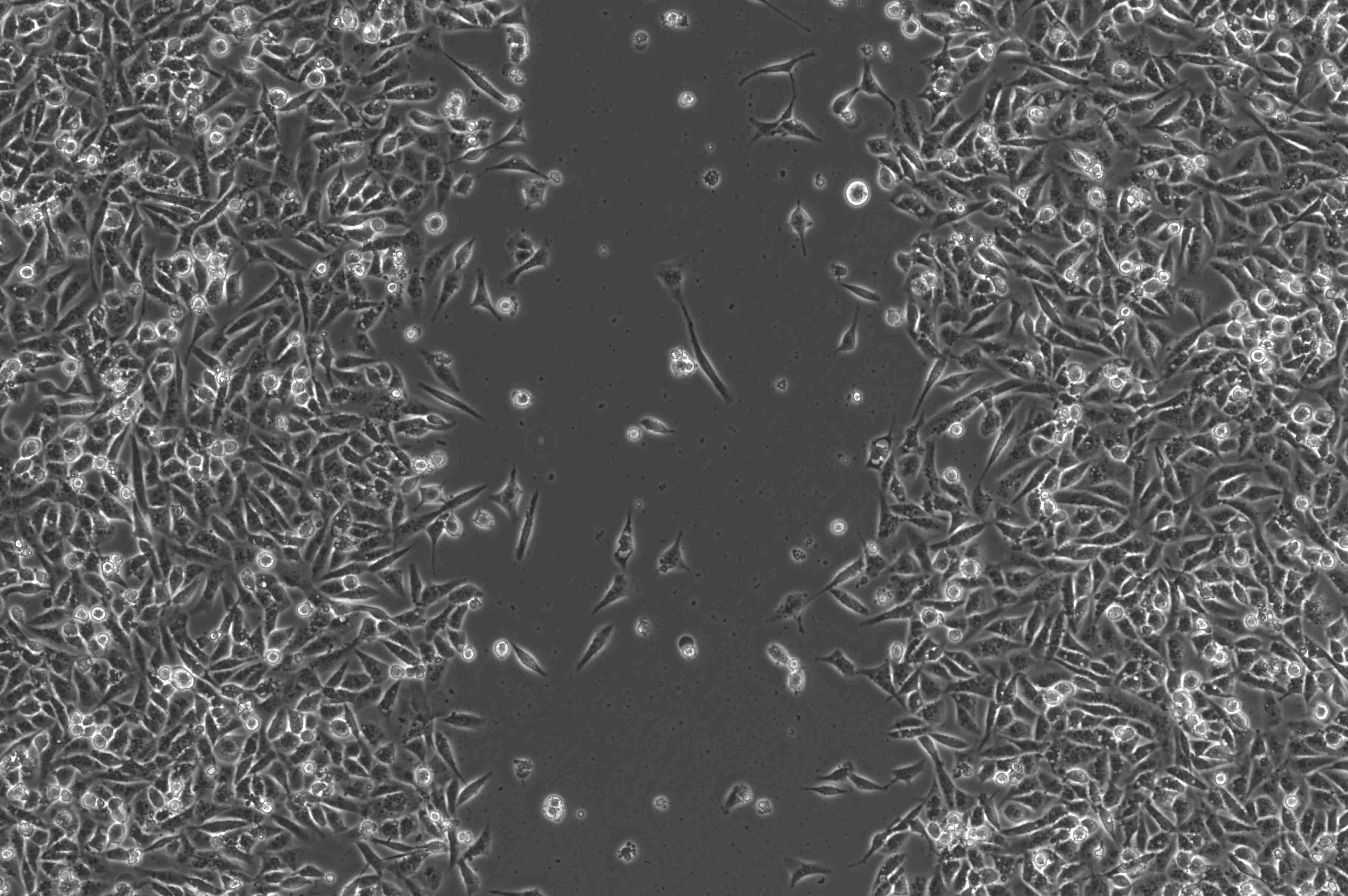

Supplement: Supplementary file 8 [file DataSheet7.ZIP › Wound healing/MDA-MB-231/2-24h-100X (2)-1_new.jpg]

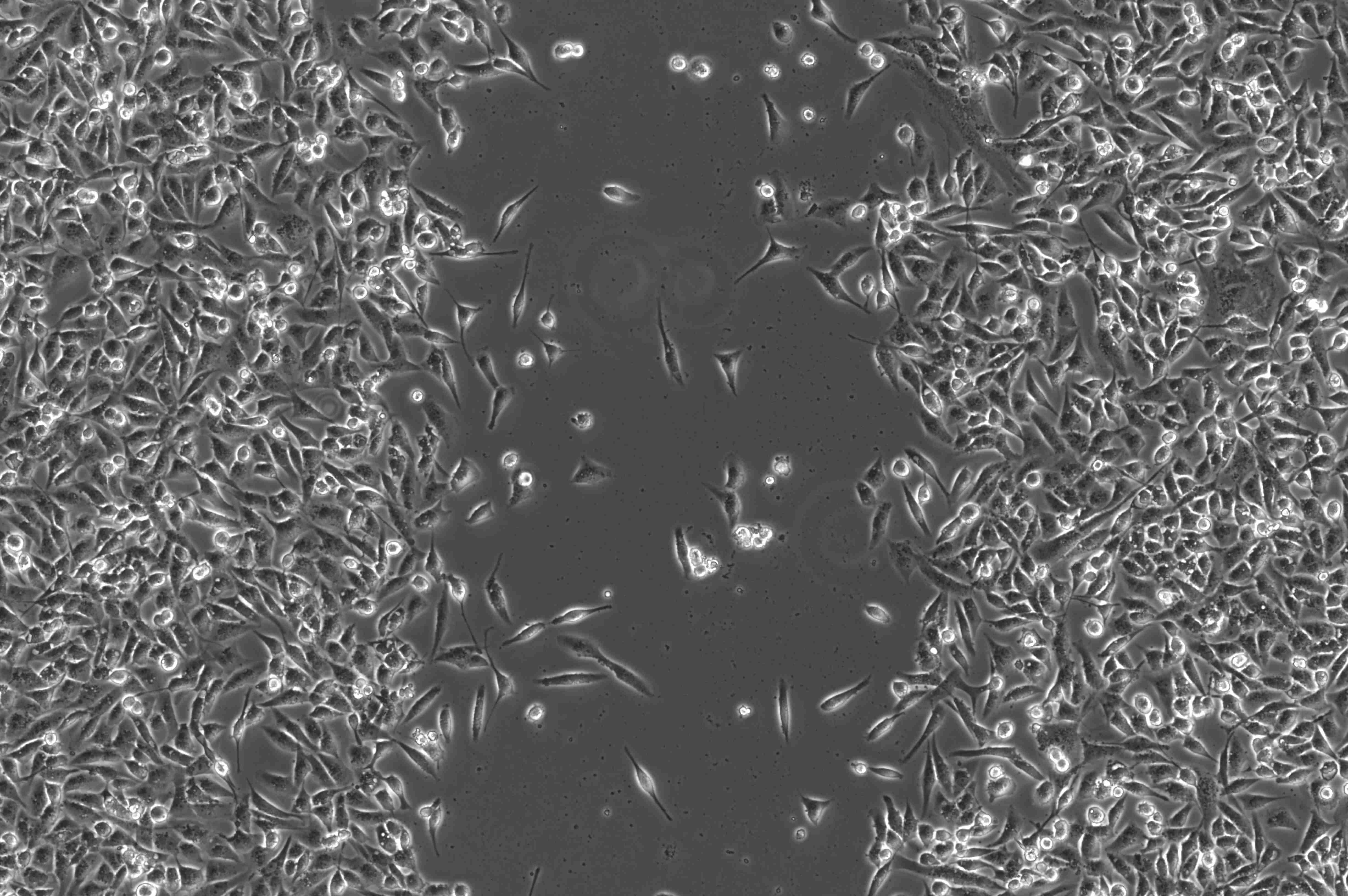

Supplement: Supplementary file 8 [file DataSheet7.ZIP › Wound healing/MDA-MB-231/2-24h-100X (3)_new.jpg]

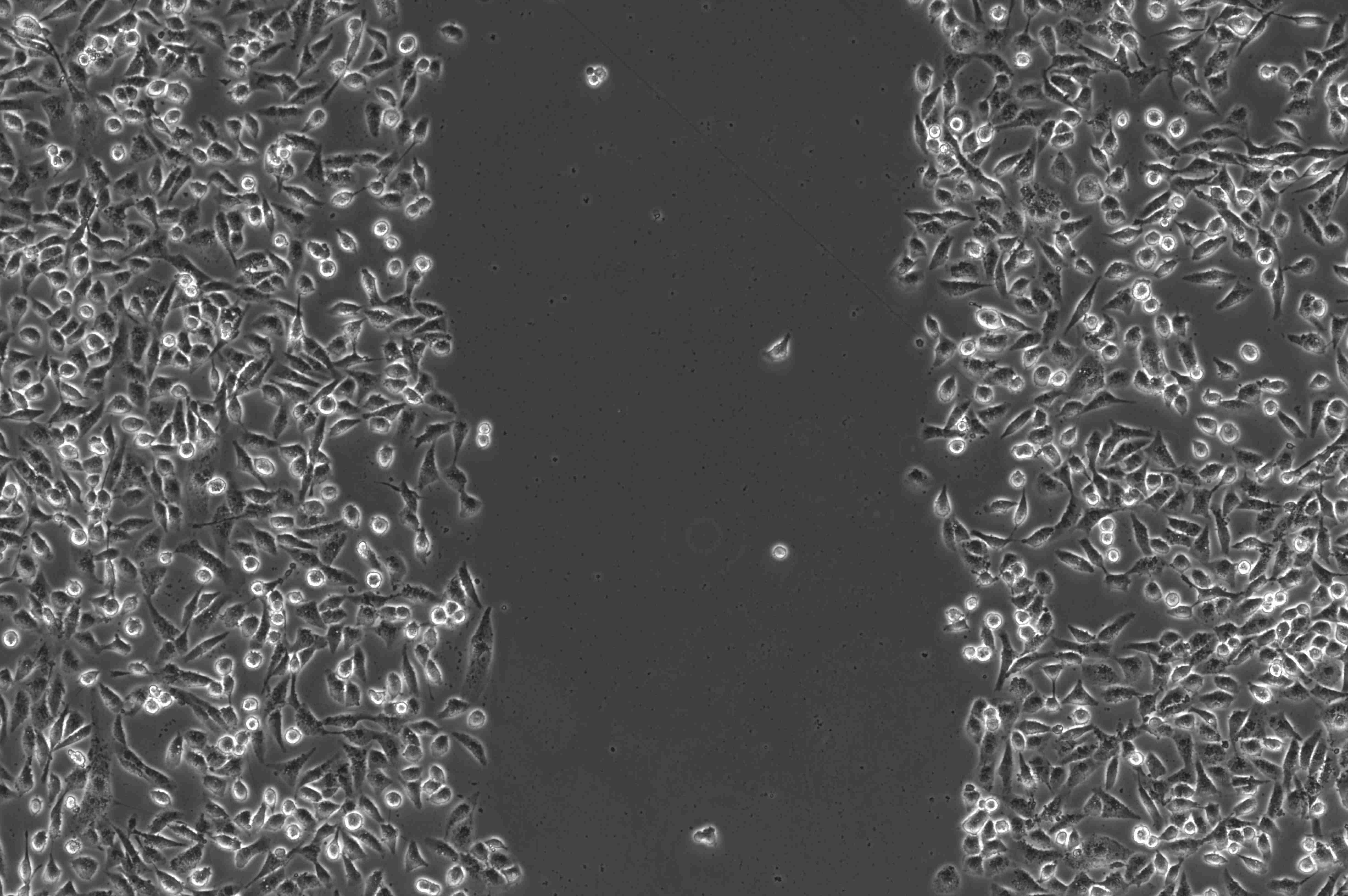

Supplement: Supplementary file 8 [file DataSheet7.ZIP › Wound healing/MDA-MB-231/3-0h-100X (1)-1_new.jpg]

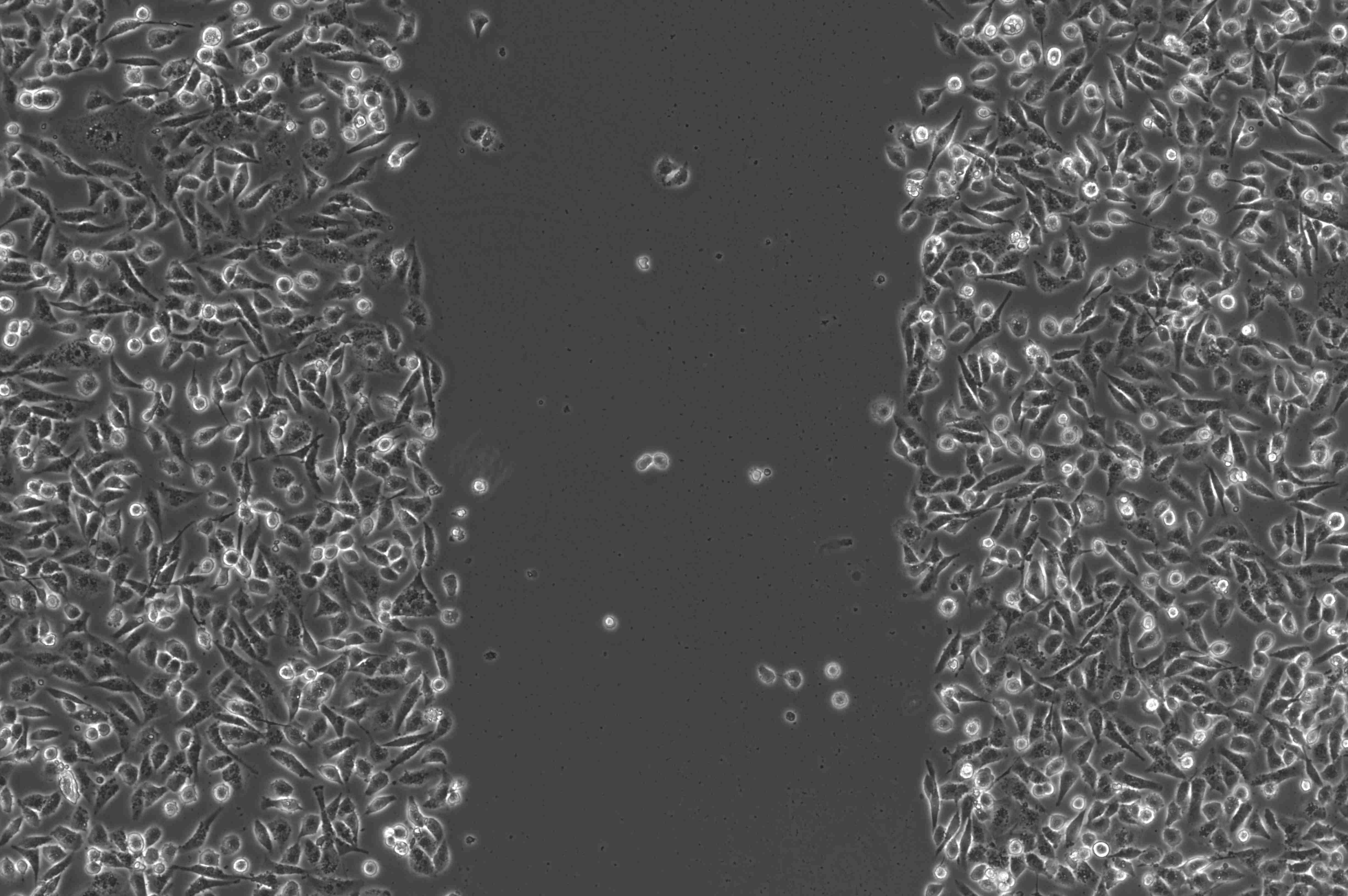

Supplement: Supplementary file 8 [file DataSheet7.ZIP › Wound healing/MDA-MB-231/3-0h-100X (3)_new.jpg]

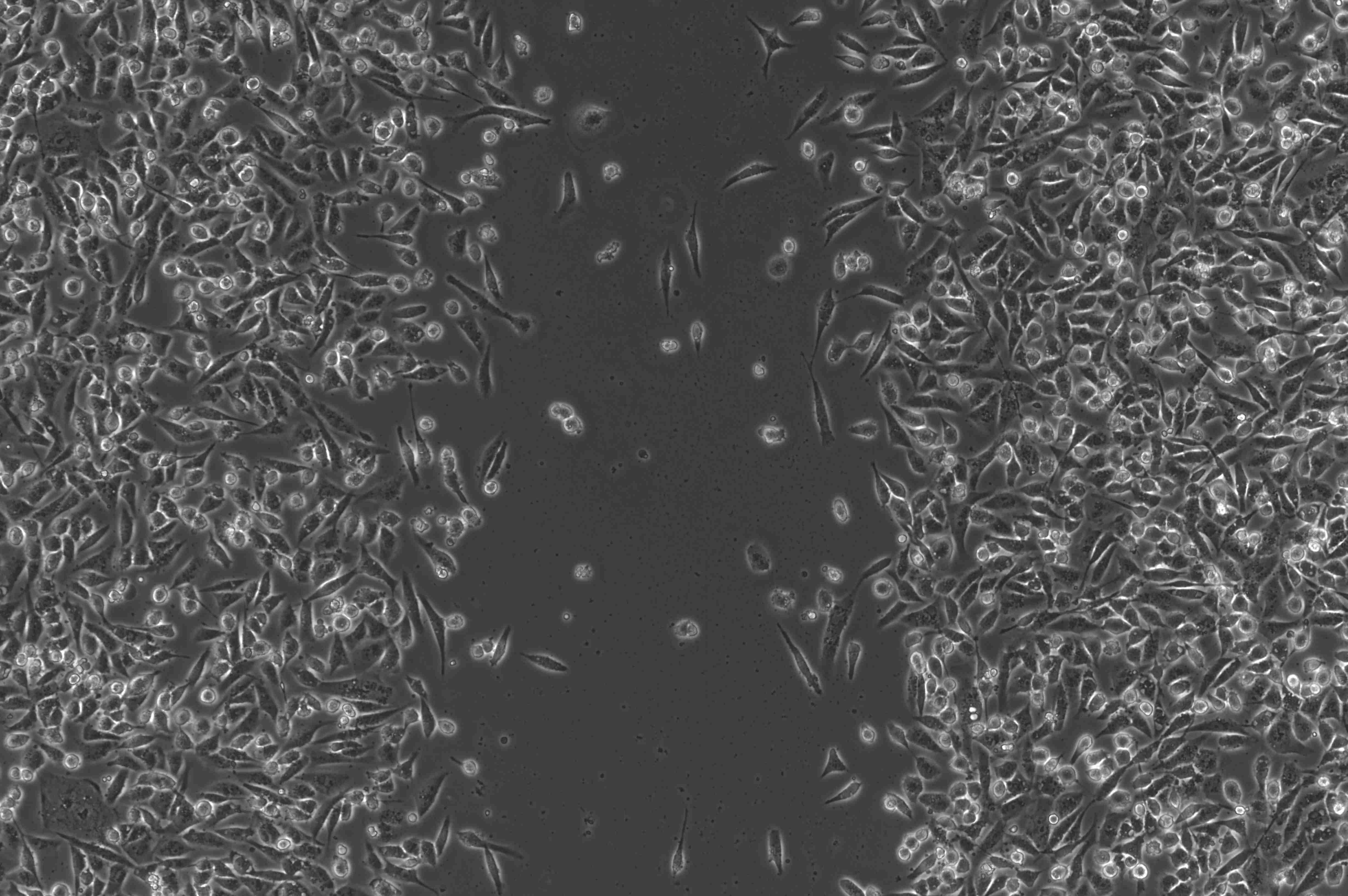

Supplement: Supplementary file 8 [file DataSheet7.ZIP › Wound healing/MDA-MB-231/3-24h-100X (1)-1_new.jpg]

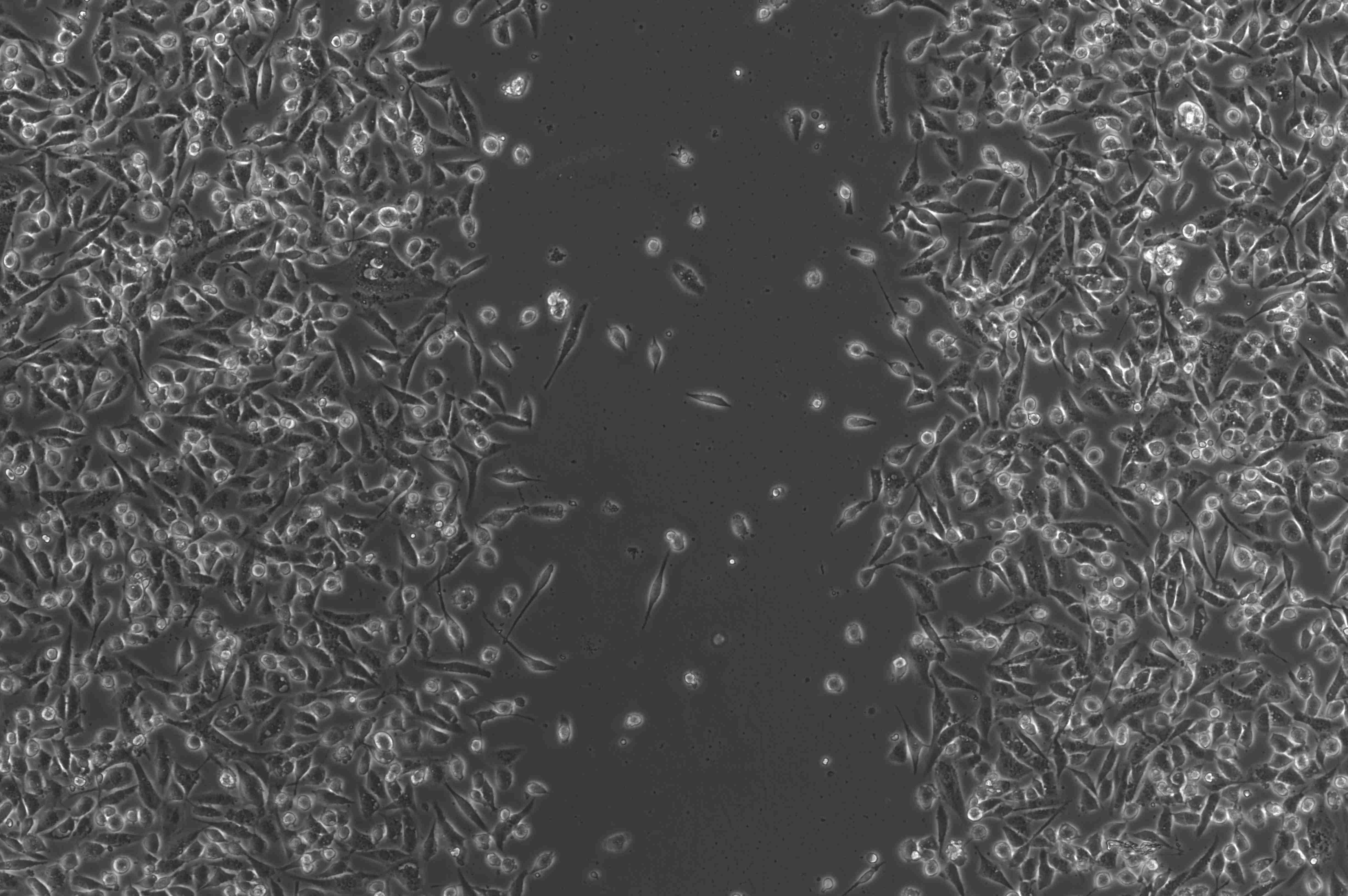

Supplement: Supplementary file 8 [file DataSheet7.ZIP › Wound healing/MDA-MB-231/3-24h-100X (2)_new.jpg]

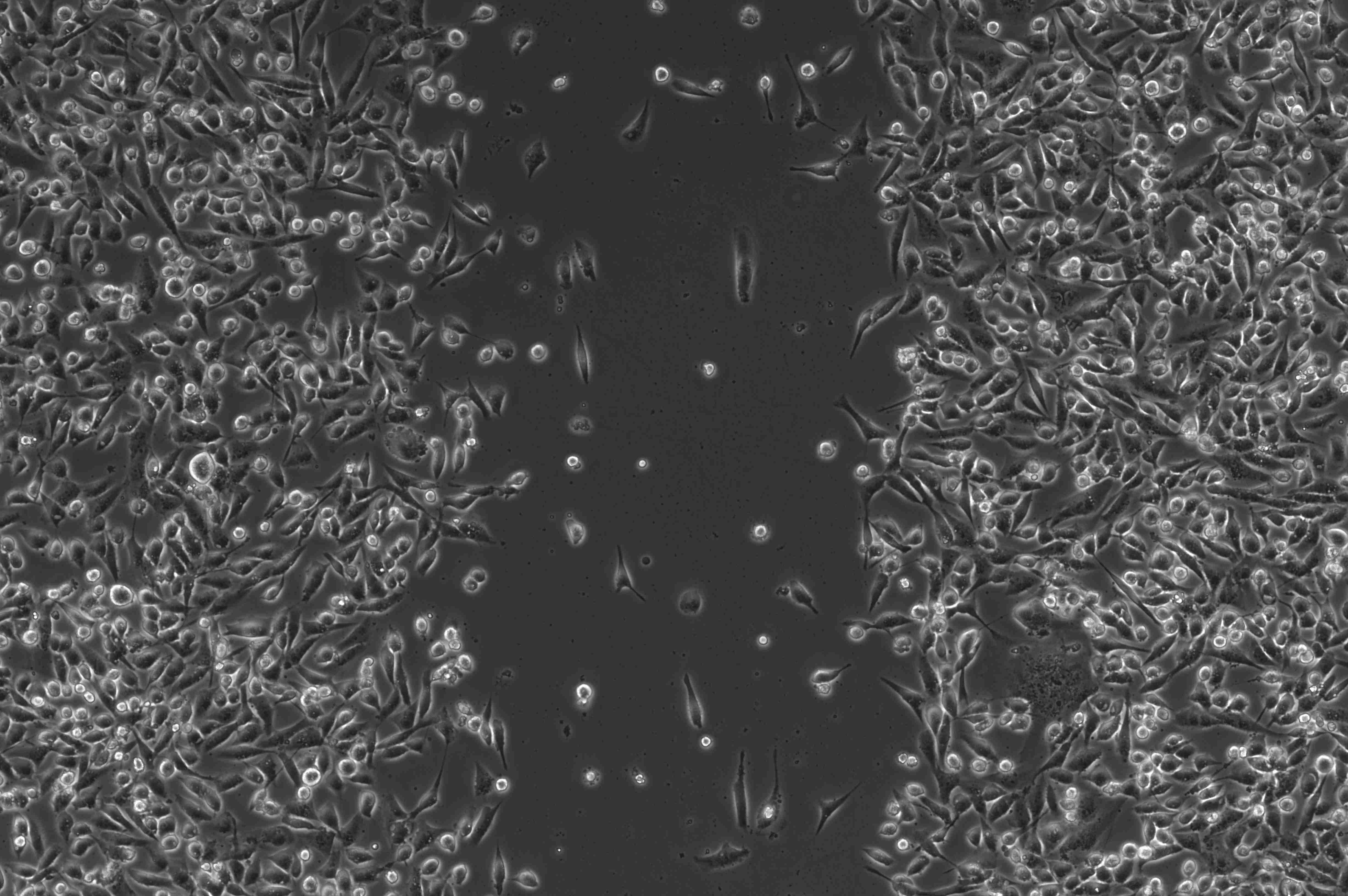

Supplement: Supplementary file 8 [file DataSheet7.ZIP › Wound healing/MDA-MB-231/3-24h-100X (3)_new.jpg]

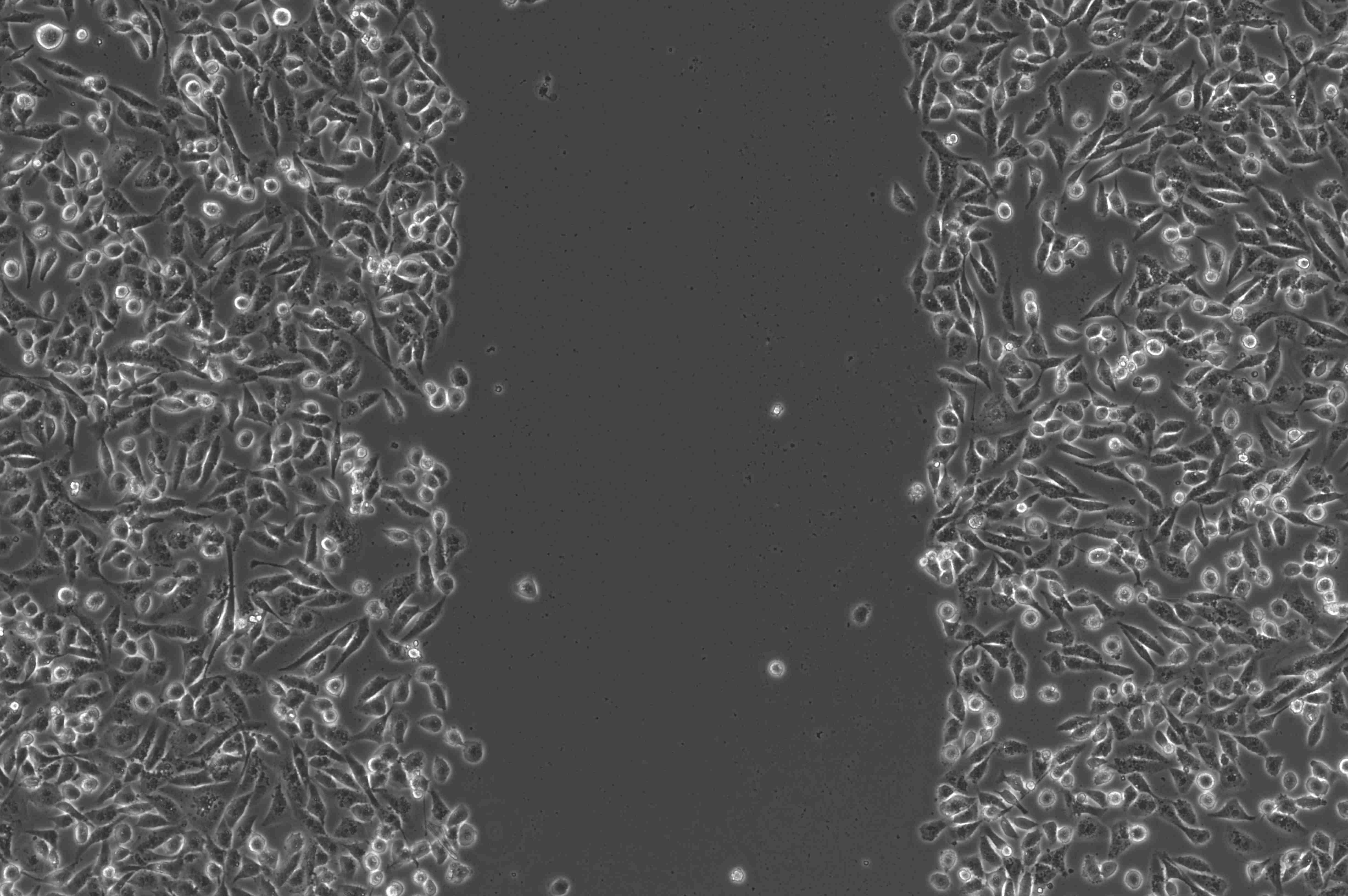

Supplement: Supplementary file 8 [file DataSheet7.ZIP › Wound healing/MDA-MB-231/4-0h-100X (1)-1_new.jpg]

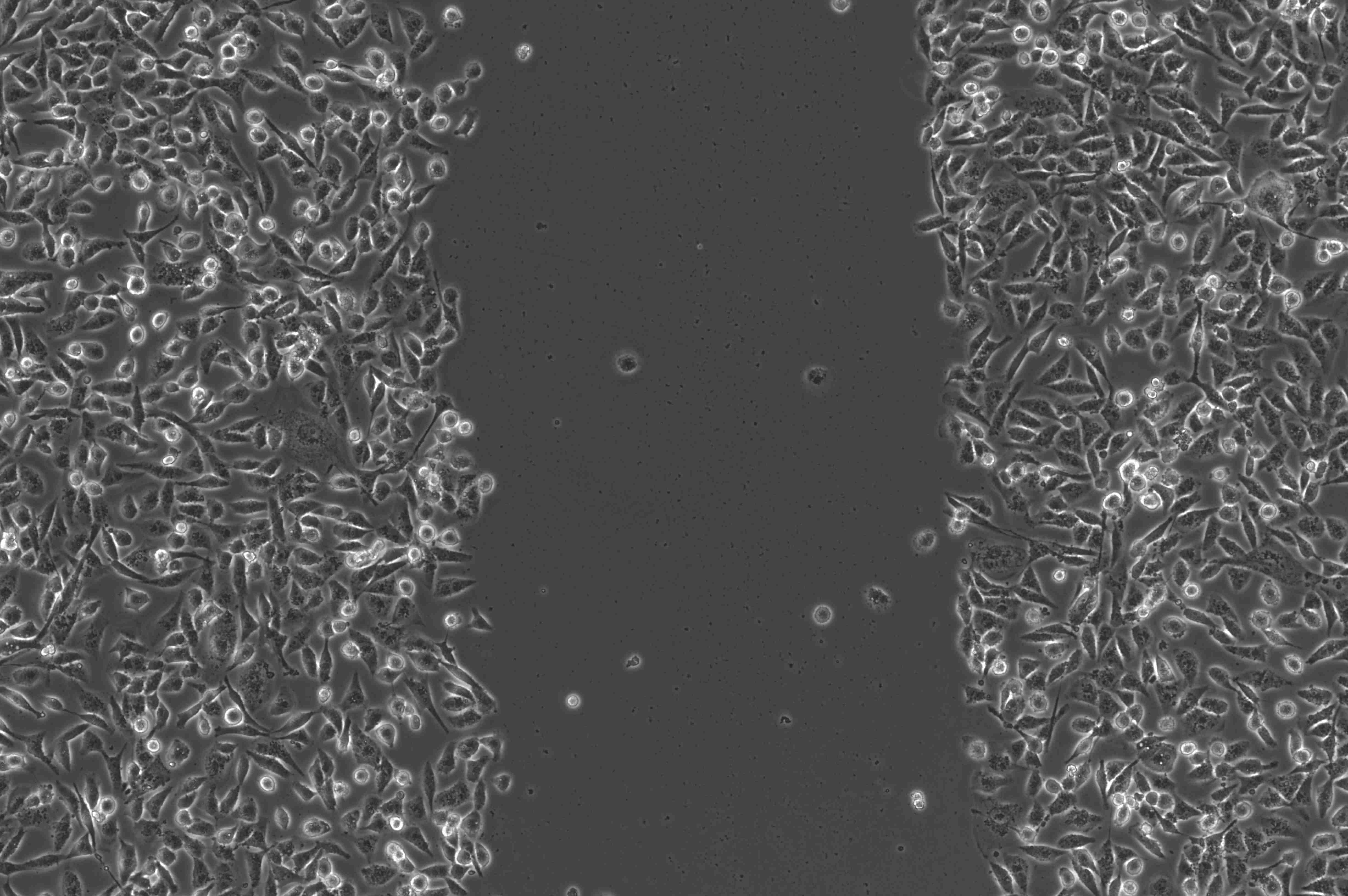

Supplement: Supplementary file 8 [file DataSheet7.ZIP › Wound healing/MDA-MB-231/4-0h-100X (2)_new.jpg]

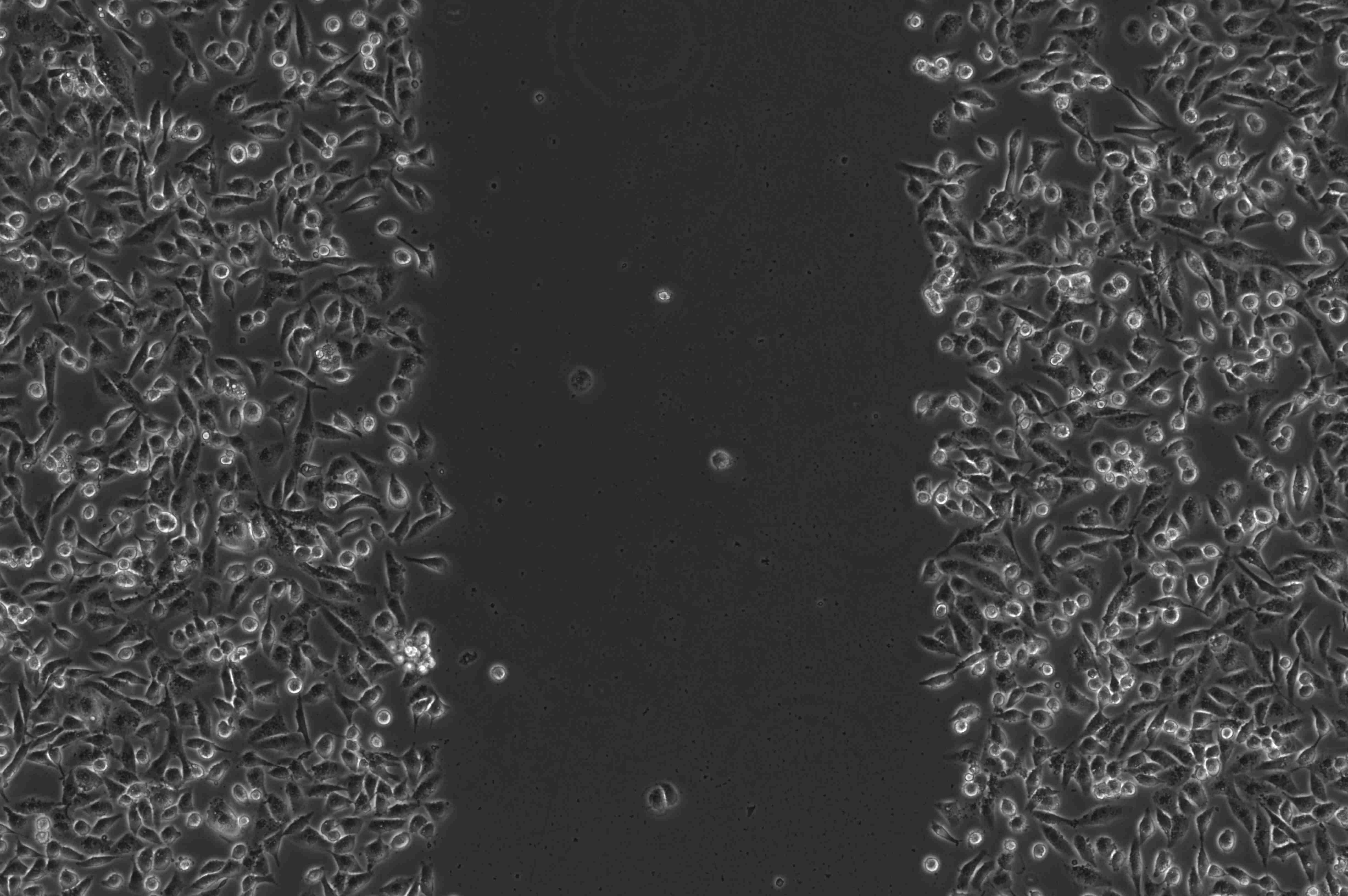

Supplement: Supplementary file 8 [file DataSheet7.ZIP › Wound healing/MDA-MB-231/4-0h-100X (3)_new.jpg]

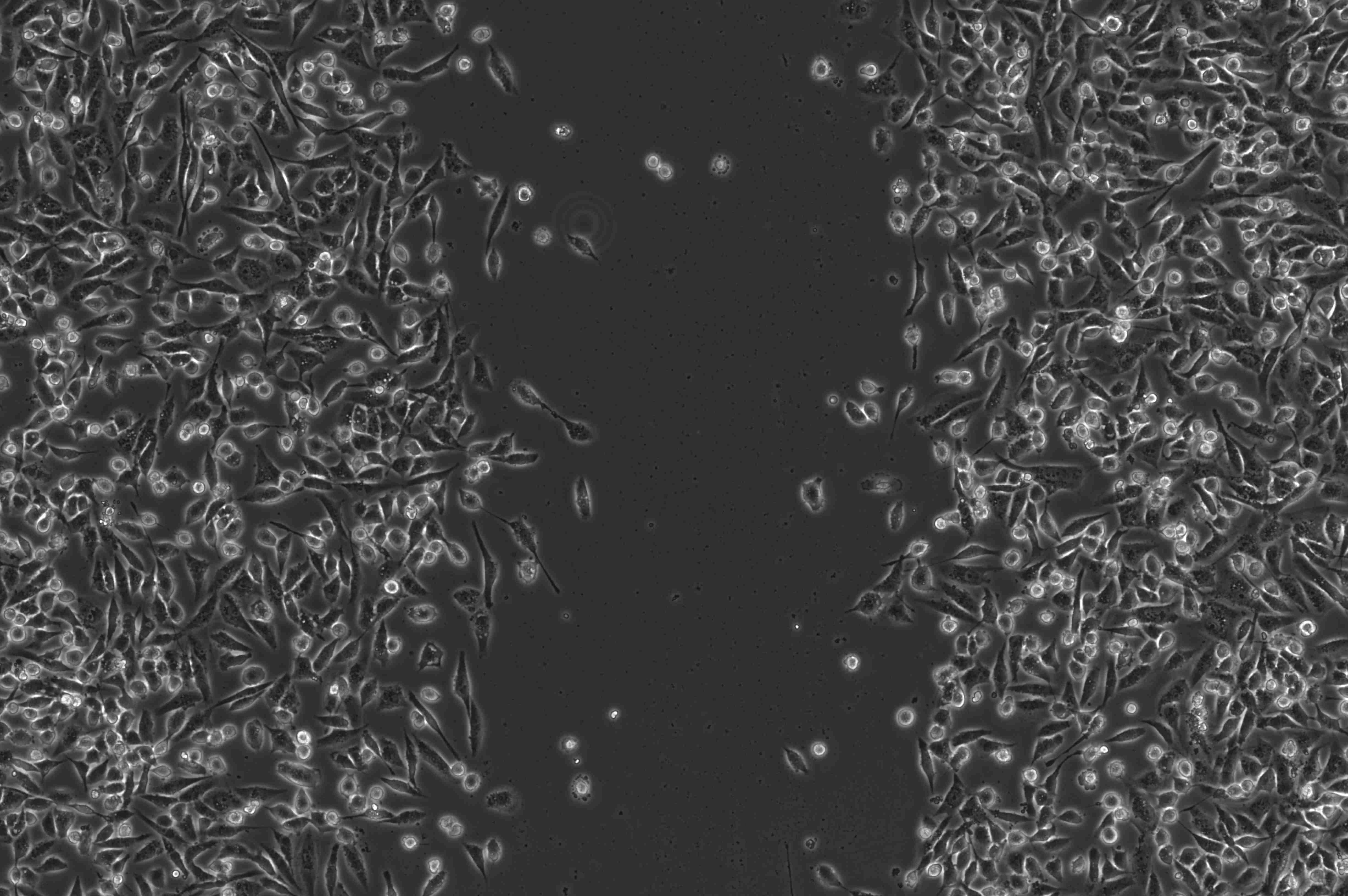

Supplement: Supplementary file 8 [file DataSheet7.ZIP › Wound healing/MDA-MB-231/4-24h-100X (1)-1_new.jpg]

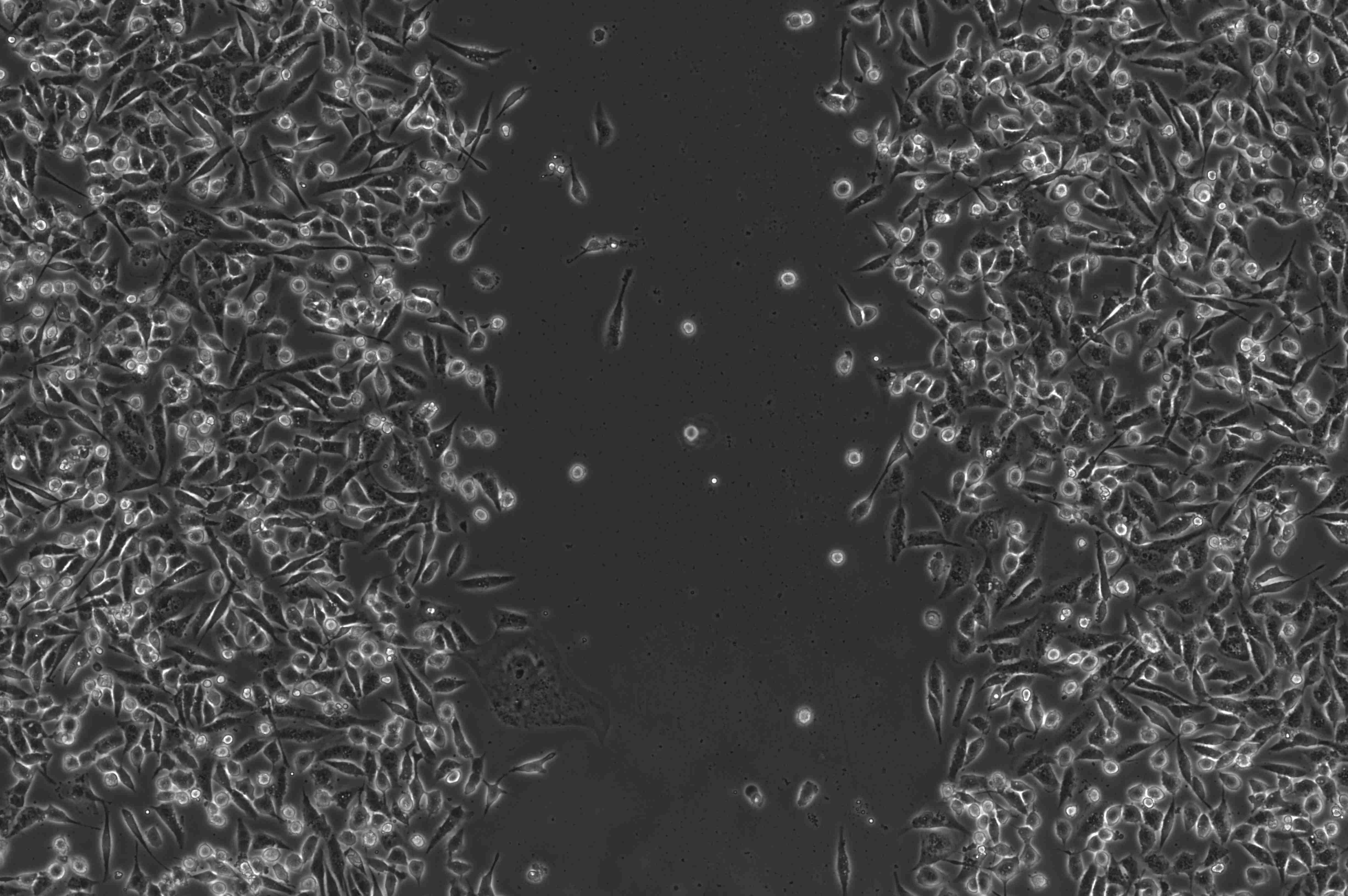

Supplement: Supplementary file 8 [file DataSheet7.ZIP › Wound healing/MDA-MB-231/4-24h-100X (2)_new.jpg]

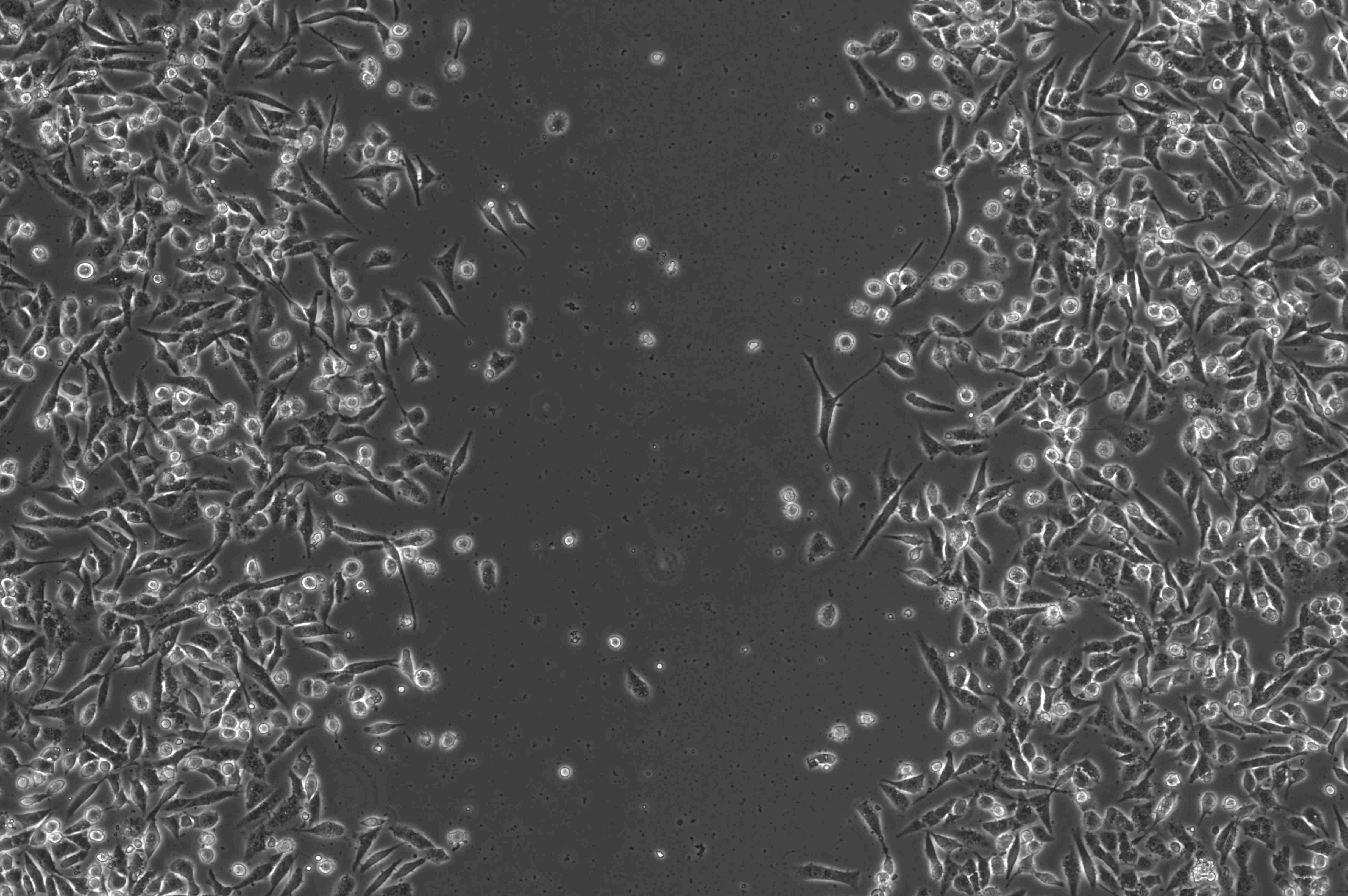

Supplement: Supplementary file 8 [file DataSheet7.ZIP › Wound healing/MDA-MB-231/4-24h-100X (3)_new.jpg]
